# Regulation of microtubule nucleation in mouse bone marrow-derived mast cells by ARF GTPase-activating protein GIT2

**DOI:** 10.3389/fimmu.2024.1321321

**Published:** 2024-02-02

**Authors:** Vadym Sulimenko, Vladimíra Sládková, Tetyana Sulimenko, Eduarda Dráberová, Věra Vosecká, Lubica Dráberová, Omar Skalli, Pavel Dráber

**Affiliations:** ^1^ Laboratory of Biology of Cytoskeleton, Institute of Molecular Genetics of the Czech Academy of Sciences, Prague, Czechia; ^2^ Laboratory of Signal Transduction, Institute of Molecular Genetics of the Czech Academy of Sciences, Prague, Czechia; ^3^ Department of Biological Sciences, The University of Memphis, Memphis, TN, United States

**Keywords:** mast cells, centrosome, G protein-coupled receptor kinase-interacting protein 2 (GIT2), microtubule nucleation, protein kinase C (PKC)

## Abstract

Aggregation of high-affinity IgE receptors (FcϵRIs) on granulated mast cells triggers signaling pathways leading to a calcium response and release of inflammatory mediators from secretory granules. While microtubules play a role in the degranulation process, the complex molecular mechanisms regulating microtubule remodeling in activated mast cells are only partially understood. Here, we demonstrate that the activation of bone marrow mast cells induced by FcϵRI aggregation increases centrosomal microtubule nucleation, with G protein-coupled receptor kinase-interacting protein 2 (GIT2) playing a vital role in this process. Both endogenous and exogenous GIT2 were associated with centrosomes and γ-tubulin complex proteins. Depletion of GIT2 enhanced centrosomal microtubule nucleation, and phenotypic rescue experiments revealed that GIT2, unlike GIT1, acts as a negative regulator of microtubule nucleation in mast cells. GIT2 also participated in the regulation of antigen-induced degranulation and chemotaxis. Further experiments showed that phosphorylation affected the centrosomal localization of GIT2 and that during antigen-induced activation, GIT2 was phosphorylated by conventional protein kinase C, which promoted microtubule nucleation. We propose that GIT2 is a novel regulator of microtubule organization in activated mast cells by modulating centrosomal microtubule nucleation.

## Introduction

Granulated mast cells play a crucial role in allergies and innate and adaptive immune responses. They possess high-affinity IgE receptors (FcϵRIs) on their plasma membrane, and crosslinking FcϵRI-bound IgE with a multivalent antigen (Ag) activates mast cells, leading to their degranulation. This process results in the release of inflammatory mediators from secretory granules, including enzymes like proteases and β-hexosaminidase, vasoactive amines such as histamine, and proteoglycans. Subsequently, the synthesis of lipid mediators, such as prostaglandins and leukotrienes, occurs, and various cytokines and chemokines are produced (1, 2). The initial signaling event induced by FcϵRI crosslinking involves the phosphorylation of the cytoplasmic tails of the FcϵRI β- and γ-subunits, as well as the activation of non-receptor protein tyrosine kinases Lyn, Fyn, and Syk. These kinases phosphorylate intracellular and transmembrane adaptors, which in turn organize and coordinate subsequent signaling events, including Ca^2+^ influx and activation of conventional serine/threonine protein kinase C (cPKC), which is dependent both on Ca^2+^ and diacylglycerol. This further enhances the propagation of signal transduction (3, 4).

Mast cell activation triggers alterations in cell morphology, driven by a significant reorganization of the cytoskeleton, which facilitates mast cell degranulation (5, 6). Microtubules, composed of αβ-tubulin dimers, play an important role in this process, as the movement of secretory granules depends on the integrity of the microtubule network (7–9). Several studies have shown that mast cell activation results in an enhanced generation of microtubules (9–11), as well as the temporary formation of protrusions containing microtubules (12, 13). Furthermore, it has been demonstrated that the influx of Ca^2+^ is crucial for the remodeling of microtubules (12, 14). The significance of microtubule motors for both anterograde (13, 15, 16) and retrograde (17) transport of granules along microtubules has also been demonstrated. Although these findings emphasize the critical function of microtubules in degranulation, the intricate molecular processes governing microtubule remodeling in activated mast cells are not fully understood.

In mast cells, centrosomes serve as dominant microtubule nucleation sites. Centrosomal microtubules are nucleated with the help of γ-tubulin ring complexes (γTuRCs) that are composed of highly conserved but less abundant γ-tubulin (18), together with γ-tubulin complex proteins (GCPs) 2-6 (19, 20). γTuRC-activating proteins, responsible for recruiting γTuRCs to centrosomes, play a vital role in centrosomal microtubule nucleation (21, 22). Other proteins that exert a more indirect influence on microtubule nucleation are also important for regulating microtubule nucleation (23). The activity of regulatory proteins that affect centrosomal nucleation can be finely tuned through phosphorylation (24).

G protein-coupled receptor kinase-interacting proteins (GITs) represent GTPase-activating proteins (GAPs) for ADP-ribosylation factor (Arf) small GTPases (25). GITs are structurally conserved multidomain scaffold/adaptor proteins (26) that link Arf GTPases with other intracellular signaling events (27). Mice express two GIT proteins: GIT1 and GIT2. A major difference among these GITs is that GIT1 has two splice variants of comparable size, while GIT2 undergoes extensive alternative splicing, resulting in both a full-length version, GIT2-long, and a truncated version lacking the C-terminal domain, known as GIT2-short (26). GITs have been associated with the control of cell adhesion and migration, relying on the actin cytoskeleton. GITs interact with p21-activated kinase interacting exchange factors (PIXs), which serve as guanine nucleotide exchange factors (GEFs) for Rac/Cdc42 GTPases, regulating microfilaments (28). However, it has also been demonstrated that GIT1 is associated with centrosomes, where it helps in the activation of PAK1 kinase through a GTPase-independent mechanism (29). In earlier studies, we established that GIT1 serves as a positive regulator of centrosomal microtubule nucleation in mouse bone marrow-derived mast cells (30) as well as in various other cell types (31). Despite their similar domain structures, GIT1 and GIT2 may have distinct cellular functions (27). The involvement of GIT2 in microtubule organization during mast cell activation remains unexplored.

In this study, we investigated the role of GIT2 in controlling microtubule organization in activated mast cells. Our findings indicate that the activation of a stable cell line of mouse bone marrow-derived mast cells by Ag results in increased centrosomal nucleation, and GIT2 plays an important role in this process. It interacts with γTuRCs, associates with centrosomes, and, in contrast to GIT1, acts as a negative regulator of centrosomal microtubule nucleation. Phosphorylation regulates the centrosomal localization of GIT2 and during activation, GIT2 is phosphorylated by cPKCs. Our findings suggest the existence of a new signaling pathway regulating microtubule organization and degranulation in activated mast cells.

## Materials and methods

### Reagents

Calyculin A, Cytocholasin B, DMSO, DNAse I, dinitrophenyl-albumin (DNP-albumin), fibronectin, Ficoll 400, Histopaque-1077, LY-333531, 4-nitrophenyl N-acetyl-β-D-glucosaminide, nocodazole, PP2, phorbol 12-myristate 13-acetate (PMA), puromycin, and neuropeptide Substance P (SP) were procured from Sigma-Aldrich (St. Louis, MO, USA). Hygromycin B and Fura-2-acetoxymethyl ester (Fura-2-AM) were obtained from Invitrogen (Carlsbad, CA, USA), while polyethylenimine was procured from Polysciences, Inc. (Warrington, PA, USA). GST-tagged active human PKCβII (P63-10G; specific activity 423nmol/min/mg) was supplied by SignalChem Biotech (Vanier Place Richmond, BC, Canada). Protein A Sepharose CL-4B and Glutathione Sepharose 4 Fast Flow were purchased from GE Healthcare Life Sciences (Chicago, IL, USA). SuperSignal WestPico Chemiluminescent reagents were obtained from Pierce (Rockford, IL, USA). Protease-inhibitor mixture tablets (Complete EDTA-free) were acquired from Roche Molecular Biochemicals (Mannheim, Germany). Restriction enzymes were purchased from New England Biolabs (Ipswich, MA, USA). Oligonucleotides were synthesized by Merck Life Science (Prague, Czech Republic). Stocks of Calyculin A (10 mM), LY-333531 (1 mM), and PP2 (10 mM) were prepared in DMSO.

### Antibodies

The catalog numbers for primary commercial antibodies (Abs) are provided in parentheses. The previously described mouse monoclonal Abs (mAbs) TU-30 (IgG1) and TU-31 (IgG2b), which specifically target the γ-tubulin sequence 434-449, have been documented in previous studies (32, 33). Mouse mAb GTU-88 (IgG1; T6557) directed against the γ-tubulin sequence 38-53 was acquired from Sigma-Aldrich. Mouse mAbs to GCP4 (IgG1; sc-271876) and nucleolin (IgG1; sc-56640), along with rabbit Abs to fibrillarin (sc-25397) and GIT1 (sc-13961) were purchased from Santa Cruz Biotechnology (Dallas, TX, USA). Mouse mAbs SPE-7 (IgE, D8406) specific for DNP, TUB2.1 (IgG1, T4026) to β-tubulin, and rabbit Abs to actin (A2066), β-PIX (HPA004744), and GAPDH were procured from Sigma-Aldrich. The mouse mAb to ODF2 (ab43840) and the rabbit Ab to pericentrin for immunofluorescence (ab4448) were acquired from Abcam (Cambridge, UK). Rabbit Abs to calcineurin (2614), PKCβII (25453), P-(Ser/Thr)-Phe (9631), and P-Ser in PKC substrate motif (6967) were obtained from Cell Signaling (Danvers, MA, USA), while the rabbit Ab to GIT2 (GTX133285) came from Genetex (Irvine, CA, USA). The rabbit Ab to pericentrin for immunoblotting (ABT59) was sourced from Millipore (Temecula, CA, USA), and the rabbit Ab to tRFP (AB233) was provided by Evrogen (Moscow, Russia). The mouse mAb to mNeonGreen (IgG2c; 32F6) was acquired from ChromoTek (Planegg-Martinsried, Germany), and the mAb to P-Tyr (IgG2b; 05-1050) was obtained from Millipore. The mouse mAb GCP2-02 (IgG1) targeting GCP2, was characterized previously (34). For the immunoprecipitation experiments, a rabbit Ab to non-muscle myosin heavy chain (BT-561; Biomedical Technologies., Stoughton, MA, USA) and the mAb MT-03 (IgG2b), directed at microtubule-associated protein (MAP) 2ab (35), were used as negative controls. Anti-mouse (W402B) and anti-rabbit (W401B) Abs, conjugated with horseradish peroxidase, were obtained from Promega Biotec (Madison, WI, USA). Anti-mouse Ab conjugated with DyLight 549 (115-505-146) and anti-rabbit Ab conjugated with AF488 (115-545-146) were sourced from Jackson Immunoresearch Laboratories (West Grove, PA, USA).

The GST (glutathione transferase)-tagged fragment encoding the human GCP3 polypeptide (aa 1-310; UniProtKB - Q96CW5) was used as an immunogen to produce mouse mAbs against GCP3. The GST-fusion protein was prepared as described (36). The fragment encoding human GCP3 (GenBank^®^ nucleotide sequence database, accession number NM_006322.6) was amplified via PCR using forward (5’-ATATGAATTCATGGCGACCCCGGAC-3’) (*Eco*RI restriction site) and reverse (5’-ATTGTCGACTTACTGGTCCGTGTATCTTCT-3’) (*Sal*I restriction site underlined) primers with a Myc-DDK-tagged human TUBGCP3 plasmid (RC207395; OriGene Technologies, Rockville, MD, USA) serving as a template. The resulting fragment was ligated into pGEX-6P-1 (Amersham Biosciences, Uppsala, Sweden) to generate a GST-tagged fusion protein. The procedures for immunization of BALB/c mice, fusion of immune cells with mouse myeloma cells Sp2/0, screening by enzyme-linked immunosorbent assay, and subsequent cloning of hybridoma cells have been described previously (37, 38). The class and subclass of the generated mAbs were determined using IsoStrip (Cat. No. 11493027001; Roche Diagnostics, Mannheim, Germany). The selected mAb GCP3-01 (IgG1, kappa) exhibited reactivity in whole cell lysates of both human and mouse cell lines with proteins of relative molecular weights corresponding to that of GCP3 (**Supplementary Figure S1A**). To pinpoint the location of the corresponding epitope, GST-tagged GCP3 fragments were generated from the Myc-DDK-tagged human TUBGCP3 plasmid using primers summarized in **Supplementary Table S1**. Immunoblot analysis disclosed the location of the epitope in the N-terminal domain of GCP3, aa 1-25 (**Supplementary Figure S1B**). The exact position of the epitope was determined by epitope mapping (33). The 16 synthetic overlapping peptides (10-mer peptides, with an overlap of 9 aa and an offset of 1 aa) covering the aa region 1-25 were prepared by SPOT synthesis and covalently bound to the membrane (JPT Peptide Technologies, Berlin, Germany). The epitope sequence recognized by GCP3-01 is located at aa position 14-23 of human GCP3 (**Supplementary Figure S1C**). The identical aa sequence is also present in GCP3 of other species, including monkeys, mice, rats, rabbits, dogs, and horses. During the screening of hybridoma clones, we selected mouse mAb GST-01 (IgG2b, kappa), which targets GST and is suitable for the immunodetection of GST-tagged proteins (**Supplementary Figure S1B**).

### Cell cultures and transfection

A stable cell line of mouse bone marrow-derived mast cells (BMMCL) was generously provided by Dr. M. Hibbs (39). The cells were cultivated in freshly prepared RPMI-1640 culture medium, which was supplemented with 100 U/ml of penicillin, 100 µg/ml of streptomycin, MEM nonessential amino acids, 1 mM sodium pyruvate, 10% fetal calf serum (FCS), and 10% WEHI-3 cell supernatant as a source of interleukin-3 (IL-3). Cells were grown at 37°C in 5% CO_2_ and passaged every 2-3 days. BMMCL were transfected with DNA constructs through nucleofection using a Mouse Macrophage Kit and the Amaxa Nucleofector II (program Y-001; Lonza Cologne AG, Cologne, Germany), following the manufacturer’s instructions. Subsequently, the cells were transferred to culture media supplemented with IL-3 and cultured for 24-48 hours before analysis. In some cases, cells were treated for 30 min with 100 nM Calyculin A to inhibit Ser/Thr phosphatases. Alternatively, cells were pretreated for 30 min with 10 μM LY-333531 to inhibit cPKC before Calyculin A treatment. Cells were also treated for 15 min with freshly prepared pervanadate as described previously (10) to inhibit Tyr phosphatases. To suppress the activity of Src family kinases, cells were incubated for 60 min with 20 μM PP2 before pervanadate treatment. To activate PKCs, cells were incubated for 15 min with 1 μM PMA.

The HEK 293FT packaging cells, derived from human embryonic kidney tissue, were sourced from Promega Biotec. Cells were grown at 37°C in 5% CO_2_ in DMEM supplemented with 10% FCS and antibiotics. For lentivirus production, HEK 293FT cells were used at passages 4-15. Cells were transfected with DNA constructs using polyethylenimine as described previously (30).

### DNA constructs

To prepare the lentiviral vector for the expression of C-terminally TagRFP-tagged fusion proteins, the coding sequence of TagRFP-T was amplified from the pCI-TagRFP plasmid (40) using primers containing sites recognized by *BamH*I*/Not*I: forward 5’-ATAGGATCCATGGTGTCTAAGGGCGAAGA-3’ and reverse 5’-ATGCGGCCGCTTACTTGTACAGCTCGT-3’. The PCR product was ligated to the pCDH-CMV-MCS-EF1-hygro vector (System Biosciences, Palo Alto, CA), resulting in the pTagRFP-hygro vector. To prepare C-terminally TagRFP-tagged mouse GIT2, transcript variant 1 (tv1) (mGIT2, gene *Git2*; RefSeq ID: NM 019834), the coding sequence was amplified from Myc-DDK-tagged ORF clone Git2 (Origene, MR222164) using primers containing sites recognized by *NheI/BamHI*: forward 5’-ATAGCTAGCATGTCGAAGCGGCTCCG-3’ and reverse 5’-ACAGGATCCGCTGCTGTTCTCTTTGGTG-3’. The PCR product was ligated into pTagRFP-hygro, resulting in the pmGIT2-TagRFP-hygro vector. To prepare C-terminally TagRFP-tagged mouse GIT1 (tv1) (mGIT1, gene *Git1*; RefSeq ID: NM_001004144.1), the RFP coding sequence was amplified from the pCI-TagRFP plasmid using primers containing sites recognized by *Xho*I/*BstB*I: forward 5′- ATTCCTCGAGATGGTGTCTAAGGGCGAA -3′ and reverse 5′- CGGCTTCGAATTACTTGTACAGCTCGTCC -3′. The PCR product was ligated to pmGIT1-GFP-hygro (see below) cleaved by *Xho*I/*BstB*I (the GFP tag was digested out from the vector), resulting in the pmGIT1-TagRFP-hygro vector. To prepare the pmGIT1-GFP-hygro vector, the cassette encoding mouse GIT1-GFP was digested out from the mGIT1-GFP-puro vector (30) by *Xba*I/*BstB*I and inserted into the pCDH-CMV-MCS-EF1-hygro vector.

To prepare the lentiviral vector for the expression of C-terminally mNeonGreen-tagged mouse GIT2, the TagRFP tag was digested out from pmGIT2-TagRFP-hygro with *BamHI/NotI*, resulting in the pmGIT2-hygro vector and the mNeonGreen coding sequence was amplified from the mNeonGreen-EB3-7 plasmid (Allele Biotechnology, San Diego, CA, USA) using forward 5’-ATTAGGATCCATACCGGTCGCCACCATG-3’ and reverse 5’-ATGCGGCCGCTTACTTGTACAGCTCG-3’ primers. The PCR product was digested by *BamHI/NotI* and ligated into pmGIT2-hygro, resulting in the pmGIT2-mNeonGreen-hygro vector. To prepare the lentiviral vector for the expression of mNeonGreen tag, the coding sequence of mNeonGreen was digested out from the mNeonGreen-EB3-7 plasmid (Allele Biotechnology) by *BamHI/NotI* and ligated to pCDH-CMV-MCS-EF1-hygro vector (System Biosciences), resulting in the pmNeonGreen-hygro vector. To prepare the lentiviral vector for the expression of C-terminally mNeonGreen-tagged mouse γ-tubulin-1 (mTUBG1, gene *Tubg1*; RefSeq ID: NM_134024), cassette encoding mTUBG1-mNeonGreen was digested out from plasmid pβactin-Tubg1-mNeonGreen (41) by *NheI/NotI* and inserted into the pCDH-CMV-MCS-EF1-hygro vector (System Biosciences), resulting in the pmTUBG1-mNeonGreen-hygro vector.

The plasmid pGST-hTUBG1, which encodes N-terminally GST-tagged human γ-tubulin, has been described previously (36). The plasmid mNeonGreen-EB3-7 (Allele Biotechnology) was used for the expression of tagged end-binding protein 3 (EB3).

### RNA interference

A set of five mouse Git2 (Gene ID: 26431) short hairpin RNA (shRNA) constructs, cloned into the lentiviral PLKO.1 vector (TRCN0000088573, TRCN0000088574, TRCN0000088575, TRCN0000088576, and TRCN0000088577) was obtained from Open Biosystems (Huntsville, AL, USA). Additionally, two shRNA constructs (shRNA-A, shRNA-B) cloned into the PLKO.1 were prepared in the laboratory. The corresponding oligonucleotide sequences (**Supplementary Table S2**) were selected from the Broad Institute’s *GPP Web Portal* (https://portals.broadinstitute.org/gpp/public/). Sense and anti-sense oligonucleotides were annealed and inserted into the pLKO.1 puro vector (Addgene, Watertown, MA, USA) after linearization with *Age*I/*Eco*RI. These vectors target all transcript variants of mouse GIT2. Immunoblotting experiments revealed that the most significant reduction in GIT2 protein levels was achieved using vector shRNA-A (GIT2_KD1) and vector shRNA-B (GIT2_KD2).

Cells transduced with the pLKO.1 vector containing non-target shRNA (Sigma) were used as the negative control (pLKO.1-NT).

### Lentiviral infection

Lentiviral infections were conducted as described previously (12), using HEK 293FT packaging cells for virus production. After 3 days, the transfection mixture was replaced with a fresh complete medium containing either 5 μg/ml puromycin or 1mg/ml Hygromycin B. Stable selection was accomplished by culturing cells with antibiotics for 1-2 weeks. In phenotypic rescue experiments, the medium containing puromycin was supplemented with Hygromycin B, and stable selection was achieved by culturing cells for 1-2 weeks.

### Gene editing

We employed CRISPR/Cas9 gene editing (42) to disrupt the expression of mouse *Git2* (Ensembl: ENSMUSG00000041890). Plasmids SpCas9 and pU6-sgRNAnew-III, generously provided by Dr. R. Malík, Institute of Molecular Genetics, CAS, Prague, Czech Republic, were used for optimal Cas 9 production and the generation of single-guide RNAs (sgRNAs), respectively. DNA oligonucleotides required for sgRNA production were designed using CRISPR design software (https://www.deskgen.com) and then cloned into the *Bsm*BI sites of pU6-sgRNAnew-III. To selectively enrich cells with disrupted *Git2* expression, we utilized the pRR-puro plasmid with a multiple cloning site housing a non-functional puromycin resistance cassette (43). Sense and anti-sense oligonucleotides, containing sequences from the region of interest and overhangs with *Aa*tII/*Sac*I restriction sites, were annealed and ligated into pRR-puro, which had been previously digested with *Aat*II/*Sac*I. This led to the creation of the reporter plasmid pRR-mGIT2-puro. Co-transfection of the reporter plasmid with plasmids encoding sgRNAs and Cas9 induced a CRISPR-mediated double-strand break in the reporter plasmid. Upon repair of this double-strand break-through homologous recombination, puromycin resistance was restored.

### Generation of cell lines lacking GIT2

To delete a significant portion of the GIT2 gene, BMMCL were transfected via nucleofection with CRISPR/Cas9 vectors comprising different pairs of sgRNAs and SpCas9, together with reporter plasmid pRR-mGIT2-puro, which was used to enrich cells lacking GIT2 expression. The transfection mixture consisted of 1 µg of each sgRNA, 1 µg of the reporter plasmid, and 2 µg of SpCas9. After 48 hours, the culture medium was replaced, and puromycin was added to a final concentration of 5 µg/ml. Stable selection was accomplished by culturing cells with puromycin for 1 week. Subsequently, a single-cell dilution protocol was employed to obtain cell clones, which were then subjected to PCR and immunoblotting analysis. GIT2_KO cell lines were generated using different pairs of sgRNAs in the transfection mixture: GIT2_KO1 (sgRNAs #1 and #3), GIT2_KO2 (sgRNAs #1 and #5), and GIT2_KO3 (sgRNAs #2 and #4). Single-cell clones were expanded, and genomic DNA was extracted using the QIAamp DNA Mini Kit (QIAGEN, Gilden, Germany). The sgRNA target sites were then amplified by PCR with primers flanking the corresponding sites (**Supplementary Table S3**). The resulting PCR fragments were subcloned into a pCR2.1 vector using a TA cloning kit (Invitrogen), and DNA from individual colonies was sequenced.

In phenotypic rescue experiments, GIT2_KO cells expressing either GIT2-TagRFP, GIT1-TagRFP or TagRFP alone were subjected to sorting using a BD Influx cell sorter (BD Bioscience, San Jose, CA, USA). The emission of TagRFP was triggered by a 561 nm laser, and the resulting fluorescence was detected using a 585/29 band-pass filter.

### Real-time qRT-PCR

Total RNAs were extracted in four separate isolations from both non-activated and activated wild-type Control (pLKO.1-NT) or GIT2_KD1 cells using the RNeasy Mini Kit (QIAGEN), following the manufacturer’s protocols. Subsequently, the RNAs were converted into cDNA using the High-Capacity cDNA Reverse Transcription Kit with random primers (Applied Biosystems, Waltham, MA, USA), according to the manufacturer’s instructions. For quantitative PCRs, gene-specific primers were designed for mouse interleukin 6 (*Il6*, NCBI Ref. Seq.: NM_031168.2, NM_001314054.1; primers anneal to both transcript variants), mouse interleukin 13 (*Il13*, NCBI Ref. Seq.: NM_008355.3), tumor necrosis factor (*Tnf*, NCBI Ref. Seq.: NM_013693.3, NM_001278601.1; primers anneal to both transcript variants), prostaglandin-endoperoxide synthase 2 (*Ptgs2*, NCBI Ref. Seq.: NM_011198.4), and β-actin (*Actb*, NCBI Ref. Seq.: NM_007393.5). In silico testing by NCBI BLAST confirmed that all primers were specific to their intended targets. The primer sequences are listed in **Supplementary Table S4**. Quantitative PCRs were performed using the LightCycler 480 System (Roche, Mannheim, Germany), following established protocols (33). Each sample was analyzed in triplicate, and the identity of PCR products was confirmed through sequencing.

### Chemotaxis assay

Cell chemotaxis was evaluated using a 24-well Transwell system with polycarbonate filters of 8-μm pore size (Corning Inc., Durham, NC, USA), following the previously described protocol (44). DNP-albumin, at a concentration of 250 ng/ml in RPMI 1640, supplemented with 20 mM HEPES and 1% BSA served as the chemoattractant. Three independent experiments were carried out.

### Cell activation

Cells were sensitized with DNP-specific IgE (mouse mAb SPE-7; 1 μg/ml) for 2 hours in a culture medium without 10% WEHI-3 cell supernatant. After sensitization, the cells were activated with Ag (DNP-albumin conjugate; 100 ng/ml; 30-40 mol DNP/mol albumin) for 30 min at 37°C. Activation occurred in a BSS-BSA buffer, which comprised 20 mM HEPES, pH 7.4, 135 mM NaCl, 5 mM KCl, 1.8 mM CaCl_2_, 2 mM MgCl_2_, 5.6 mM glucose, and 0.1% BSA, following the described protocol (12). In certain cases, sensitized cells were treated for 30 min with 10 μM LY-333531 before their activation with the Ag. For experiments involving time-lapse imaging, cells were sensitized while in suspension then applied to fibronectin-coated coverslips, and subsequently activated (12). To depolymerize microtubules, sensitized cells were treated with 10 μM nocodazole for 1 h at 37°C and then activated with Ag in the presence of nocodazole. Alternatively, non-sensitized cells in BSS-BSA buffer were activated with 20 μM SP for 30 min at 37°C.

### Degranulation assay and determination of intracellular Ca^2+^ concentration

The quantification of degranulation involved assessing the release of β-hexosaminidase from Ag-activated cells, utilizing 4-nitrophenyl N-acetyl-β-D-glucosaminide as a substrate (9). Each sample was analyzed in triplicate. To calculate the extent of degranulation, the formula absorbance of the culture supernatant × 100/absorbance of the total cell lysate was applied. The values were subsequently normalized to those of control cells.

Changes in intracellular Ca^2+^ levels were monitored using Fura-2-AM as a cell-permeant calcium reporter, following the established protocol (12). The levels of intracellular free Ca^2+^ were measured with a microplate reader, Infinite M200 (Tecan, Männedorf, Switzerland), as the ratio of Fura emissions at 510 nm after excitation with 340 nm and 380 nm (340/380) lasers at the designated time points. After measuring the baseline Ca^2+^ level, activation was initiated by adding Ag.

### Evaluation of cell growth

Cell proliferation was evaluated by manually counting both control and GIT2_KO cells. A total of 3 x 10^5^ cells, diluted in culture medium, were deposited into each well of a 6-well plate. Cells were counted at daily intervals, from the first to the fourth day. Samples were counted in pairs in at least six independent experiments.

### Preparation of cell extracts

When preparing cell extracts for immunoprecipitation and GST pull-down assays, cells were rinsed twice with cold HEPES buffer (comprising 50 mM HEPES at pH 7.6, 75 mM NaCl, 1 mM MgCl_2_, and 1 mM EGTA). Subsequently, the cells were extracted at a concentration of 1 x 10^7^ cells/ml for 10 min at 4°C with HEPES buffer that was further supplemented with 1% NP-40 and phosphatase (1mM Na_3_VO_4_ and 1 mM NaF) and protease inhibitors. The suspension was then subjected to centrifugation (20,000 x g, 15 min, at 4°C), and the resulting supernatant was collected.

The analysis of microtubule polymer was conducted according to a modified method that was previously described (45). Briefly, 8.0 x 10^6^ cells were rinsed twice in PEM buffer (comprising 50 mM PIPES at pH 6.8, 3 mM MgCl_2_, and 1 mM EGTA) at 37°C. Subsequently, cells were extracted for 2 min at 37°C with 0.4 ml of PEM buffer supplemented with 25% glycerol, 0.2% Triton X-100, and protease and phosphatase inhibitors. The suspension was spun down at 1,000 x g for 2 min at 25°C. The nuclear pellet, which contained microtubules, was re-suspended in 0.4 ml of a cold lysis buffer (comprising 50 mM Tris at pH 7.4, 0.5% Triton X-100, 250 mM NaCl, and 5 mM EDTA), supplemented with protease and phosphatase inhibitors, and the mixture was incubated for 5 min on ice. For immunoblot analysis, the samples were subsequently diluted 1:8 with an SDS-sample buffer.

### Centrosome isolation

Centrosomes were prepared following a modified method as previously described (46). In brief, 6.0 x 10^8^ BMMCL were pelleted and re-suspended in 100 ml of media and treated with nocodazole (10 μg/ml) and Cytochalasin B (5 μg/ml) for 90 minutes at 37°C to depolymerize microtubules and actin filaments. Subsequently, the cells were sedimented by centrifugation (500 x g, 5 min, at 4°C) and washed sequentially with 40 ml of cold PBS, 8% (w/w) sucrose in 0.1x PBS, and 8% (w/w) sucrose in double-distilled water to remove salts. The sedimented cells were lysed in 20 ml of 0.5% NP-40 in a low ionic strength buffer (composed of 1 mM HEPES, 0.5 mM MgCl_2_, 8 mM 2-mercaptoethanol) for 20 min at 4°C. Cell debris was removed by centrifugation (1,500 x g, 5 min, at 4°C), and the supernatant was filtered through a 40-μm nylon mesh (Corning). HEPES and DNAse I were added to the supernatant to reach final concentrations of 10 mM and 2 units/ml, respectively. After a 30-minute incubation, centrosomes were concentrated onto a 20% (w/w) Ficoll 400 cushion by centrifugation (25,000 x g, 20 min, at 4°C). The sample enriched for centrosomes was then layered on a discontinuous gradient consisting of 5 ml of 70%, 3 ml of 50%, and 3 ml of 40% (w/w) sucrose in a single thin-wall Ultra-Clear Beckman tube (Cat. No. 344058, Beckman Coulter, Brea, CA, USA). The gradient was subjected to centrifugation (130,000 x g, 90 minutes, at 4°C) in SW 32 Ti rotor (Beckman) and subsequently fractionated from the bottom, with 0.5 ml fractions collected. The sucrose density of each fraction was measured using a refractometer. Centrosome-containing fractions were stored in aliquots in liquid nitrogen. For immunostaining, centrosomes were pelleted onto glass coverslips (47) and fixed in methanol at -20°C for 5 minutes.

The capability of centrosomes to nucleate microtubules was assessed using a modified protocol previously described (46). Centrosomes were incubated in a suspension with 8 μM polymerization-competent porcine brain tubulin (48) in the presence of 1mM GTP for 20 min at 37°C. Subsequently, they were fixed by 0.25% glutaraldehyde for 3 min at room temperature and then pelleted onto coverslips through a 25% (v/v) glycerol cushion. Samples were thereafter postfixed with methanol at -20°C for 5 min before immunostaining.

### Immunoprecipitation, kinase assay, GST pull-down assay, gel electrophoresis, and immunoblotting

Immunoprecipitation was conducted following the procedure outlined previously (49). Cell extracts were incubated with protein A beads that were saturated with mouse mAbs to (i) γ-tubulin (TU-31; IgG2b), (ii) mNeonGreen (IgG2c), and (iii) MAP2 (MT-03; IgG2b, used as a negative control). Additionally, rabbit Abs to (iv) GIT2, (v) tRFP, and (vi) non-muscle myosin (used as a negative control) were employed, along with (vii) immobilized protein A alone. Antibodies to GIT2, tRFP, and mNeonGreen were used at Ig concentrations of 0.5 μg/ml, 2.5 µg/ml, and 2.5 μg/ml, respectively. Ab to myosin was used at a dilution of 1:300. mAbs TU-31 and MT-03, in the form of hybridoma supernatants, were diluted 1:3 and 1:4, respectively.

Alternatively, immunoprecipitated material bound to beads was used for *in vitro* kinase assays, following the previously outlined procedure (10). In some experiments, we added active PKCβII (1 ng/μl) with or without 10 μM LY-333531 to immunocomplexes before the kinase assay. The ^32^P-labeled immunocomplexes were electrophoretically separated and transferred to membranes. Subsequently, the ^32^P-labeled proteins were visualized using autoradiography with the Amersham Typhoon scanner (GE Healthcare Europe GmbH, Freiburg, Germany). The preparation and purification of GST-tagged fusion proteins were previously described, as were the pull-down assays using whole-cell extracts (49).

Standard procedures, as previously outlined (50, 51), were employed for the preparation of samples for SDS-PAGE, gel electrophoresis, and immunoblotting. For immunoblotting, mAb to γ-tubulin (Sigma, GTU-88) was diluted 1:10,000. mAbs to GCP4, β-tubulin, nucleolin, and mNeonGreen were diluted 1:1,000. mAb to P-Tyr was diluted 1:2,000. mAbs to GST, GCP2, and GCP3, all in the form of hybridoma spent culture supernatant, were diluted 1:5,000, 1:5, and 1:1, respectively. Rabbit Abs to GIT2, calcineurin, pericentrin (Millipore), PKCβII, and ODF2 were diluted 1:1,000. Rabbit Abs to GAPDH, actin, GIT1, fibrillarin, tRFP, β-PIX, P-Ser in phospho-PKC motif, and P-(Ser/Thr)-Phe were diluted 1:500,000, 1:30,000, 1:5,000, 1:5,000, 1:5,000, 1:3,000, 1:2,000, and 1:1000, respectively. The secondary Abs, anti-mouse, and anti-rabbit, conjugated with horseradish peroxidase, were diluted 1:10,000. The signal was detected using SuperSignal WestPico Chemiluminescent reagents and the LAS 3000 imaging system (Fujifilm, Düsseldorf, Germany). Immunoblots were performed in at least three replicates. Signal quantification in the immunoblots was carried out using AIDA image analyzer software (version 5; Raytest, Straubenhardt, Germany).

### Microtubule nucleation visualized by time-lapse imaging

BMMCL transfected with the EB3-mNeonGreen plasmid via nucleofection were cultured for 24 hours. Subsequently, they were layered on a cushion of Histopaque-1077, and viable cells were recovered by centrifugation (400 x g, 40 min, at 25°C) at the interface between the sample and the Histopaque-1077. After a PBS wash, the cells were transferred to culture medium and incubated for 60 minutes in a 35 mm µ-Dish with a polymer coverslip bottom (Ibidi GmbH, Gräfelfing, Germany) that had been pre-treated with fibronectin, as previously described (12). Before imaging, the medium was replaced with FluoroBrite™ DMEM (ThermoFisher Scientific, Waltham, MA, USA) supplemented with 25 mM HEPES and 2% FCS, 30 minutes prior to imaging. Time-lapse sequences were captured with the Andor Dragonfly 503 spinning disc confocal microscope (Oxford Instruments, Abingdon, UK) using the following setup: five optical slices at 0.2 µm steps for 30 seconds at 1-second intervals. The microscope was equipped with a stage top microscopy incubator from Okolab (Ottaviano, Italy), a 488 nm solid-state 150 mW laser, HC PL APO 63x/1.2 water objective, and an iXon Ultra 888 EMCCD camera. Each experiment involved imaging at least 9 cells with the following acquisition parameters: 40 µm pinhole size, 15% laser power, 50 ms exposure time, and a 525/50 nm emission filter. The time-lapse sequences were deconvoluted using Huygens Professional software (version 19.04; Scientific Volume Imaging, Hilversum, the Netherlands), and a maximum intensity projection of the z-stack was generated for each time point with the Fiji Processing program (52). The identification of newly nucleated microtubules was carried out by manually counting EB3 comets emanating from the centrosomes.

To visualize microtubule nucleation during the activation event, viable cells expressing EB3-mNeonGreen were sensitized with DNP-specific IgE while in suspension. They were then placed on a fibronectin-coated coverslip at the bottom of a 35-mm µ-Dish and subsequently activated with Ag in FluoroBrite™ DMEM, supplemented with 25 mM HEPES and 2% FCS. Alternatively, non-sensitized cells in this medium were activated with 20 μM SP. To monitor changes in microtubule nucleation within the same cells during activation, sensitized cells were placed on a 35 mm glass-bottom dish (Cellvis, Mountain View, CA, USA; D35-14-1.5-N) coated with fibronectin, and a perfusion insert RC-37F (Warner Instruments, Hamden, CT, USA) was positioned within the dish. The medium was exchanged for Ag-containing medium at a controlled flow rate of 7.4 ml/min using a Reglo ICC peristaltic pump (Masterflex LLC, Barrington, IL, USA).

### Immunofluorescence microscopy

Immunofluorescence microscopy was conducted on cells attached to fibronectin-coated coverslips (12) using various fixation methods (53). Samples were extracted in 0.5% Triton X-100 for 5 min at 37°C, fixed with 3% formaldehyde for 20 min at room temperature and postfixed with precooled methanol for 10 min at -20°C (Tx/F/M). Alternatively, samples were fixed in formaldehyde and then extracted in Triton X-100 (F/Tx). Samples were also postfixed in methanol after F/Tx fixation (F/Tx/M). Centrosomes were fixed in methanol (M). Centrosomes nucleating microtubules were fixed in glutaraldehyde and postfixed in methanol (G/A). Rhodamine-conjugated phalloidin (provided by T. Wieland) was used at a concentration of 0.5 μg/ml. mAbs to γ-tubulin (TU-30), in the form of spent culture supernatant, and β-tubulin were diluted 1:10 and 1:500, respectively. Rabbit Ab to pericentrin was diluted 1:200. AF488-conjugated anti-mouse and anti-rabbit Abs were diluted 1:200, while the DY549-conjugated anti-mouse Ab was diluted 1:1,000. The specimens were mounted in MOWIOL 4-88 (Calbiochem, San Diego, CA, USA) and examined with an Olympus AX-70 Provis microscope (Olympus, Hamburg, Germany) using a 60×/1.0 NA water immersion objective.

To quantify cell spreading, cells were incubated on fibronectin-coated coverslips for 45 min. Subsequently, they were fixed and stained with rhodamine-tagged phalloidin. Cell boundaries were delineated manually, and cell areas were computed using image analysis software from Soft Imaging System (Münster, Germany).

### Statistical analysis

We conducted a minimum of three independent experiments for each quantification, with specific data point counts provided in the figure legends. Data are presented as mean ± SD. Statistical significance was determined using either a two-tailed, unpaired Student’s *t*-test or one-way ANOVA, followed by Dunnett’s, Sidak’s, or Tukey’s *post hoc* tests implemented in Prism 8 software (GraphPad Software, San Diego, CA, USA). The tests used are specified in the figure legends. For all analyses, *p*-values are denoted as follows: *, *p* < 0.05; **, *p* < 0.01; ***, *p* < 0.001; ****, *p* < 0.0001.

## Results

### Changes in microtubule nucleation during activation of mast cells by Ag

**Figure 1:**
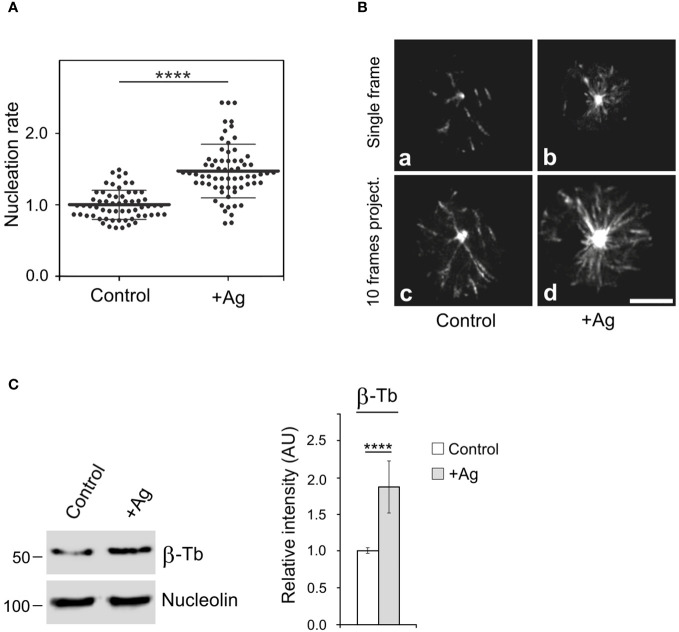
The Ag-induced activation of BMMCL results in increased centrosomal nucleation and microtubule amount. Cells were activated by FcεRI aggregation with Ag at a concentration of 100 ng/ml for 10 min. **(A)** Nucleation of microtubules in cells expressing EB3-mNeonGreen. Microtubule nucleation rate (EB3 comets/min) in activated cells (+Ag) relative to non-activated cells (Control). Three independent experiments were conducted, with a minimum of 17 cells counted in each experiment. The dot plot depicts data for Control (n = 61) and +Ag (n = 66), where bold and thin lines represent mean ± SD. Statistical significance was determined using a two-tailed, unpaired Student’s t-test. **(B)** Time-lapse imaging of control and activated (+Ag) cells. Still images of EB3 (Single frame) and projection tracks of EB3 comets over 10 s (10 frames project.). Scale bar, 5 μm (a-d). **(C)** Resting cells or activated cells (+Ag) were extracted in 0.2% Triton X-100 at 37°C, and detergent-insoluble fractions were subjected to immunoblotting with Abs to β-tubulin (β-Tb) and nucleolin (used as a loading control). The densitometric quantification of immunoblots is presented on the right. Relative intensity of β-tubulin normalized to control cells and to the amount of nucleolin. Values indicate mean ± SD (n = 8). Two-tailed, unpaired Student’s *t*-test was performed to determine statistical significance. ****, p < 0.0001.

It is known that the activation of mast cells by Ag cross-linking of FcεRIs leads to microtubule reorganization (9, 10) and the generation of protrusions containing microtubules (12, 13). To further investigate the mechanism of microtubule reorganization during activation events, we examined whether FcεRI aggregation affects *de novo* microtubule nucleation from centrosomes. Cells expressing EB3-mNeonGreen, which marks the plus ends of growing microtubules, were sensitized by IgE and activated by Ag through FcεRI aggregation. Time-lapse imaging and counting of the number of EB3 comets leaving centrosomes per unit time (nucleation rate) revealed increased centrosomal nucleation in activated cells compared to quiescent cells (**Figure 1A**). This difference was evident in both single-frame and 10-frame projections from control and activated cells (**Figure 1B**). The time-lapse sequence of the same IgE-sensitized cell before and 5 min after the addition of Ag is presented in **Supplementary Video S1**. The increased nucleation also led to a significant rise in microtubule mass in activated cells, as quantified through immunoblotting. To achieve this, cells were initially extracted at 37°C with 0.2% Triton X-100 in a microtubule-stabilizing buffer supplemented with 25% glycerol and inhibitors. Under these conditions, microtubules remained intact and did not depolymerize into αβ-tubulin heterodimers. Subsequently, microtubules with nuclei were rapidly spun down at low speed, and pelleted material was analyzed by immunoblotting with Ab to β-tubulin and Ab to the major nucleolar protein nucleolin (serving as a loading control). The signal corresponding to β-tubulin thus reflected the mass of microtubules (**Figure 1C**). Thus, activation of BMMCL by FcεRI aggregation generates more microtubules, with *de novo* centrosomal microtubule nucleation playing a crucial role in this process. This increase in microtubules could facilitate the transport of granules from the inner cytosolic space to the cell surface.

### GIT2 associates with γTuRC proteins and centrosome

**Figure 2:**
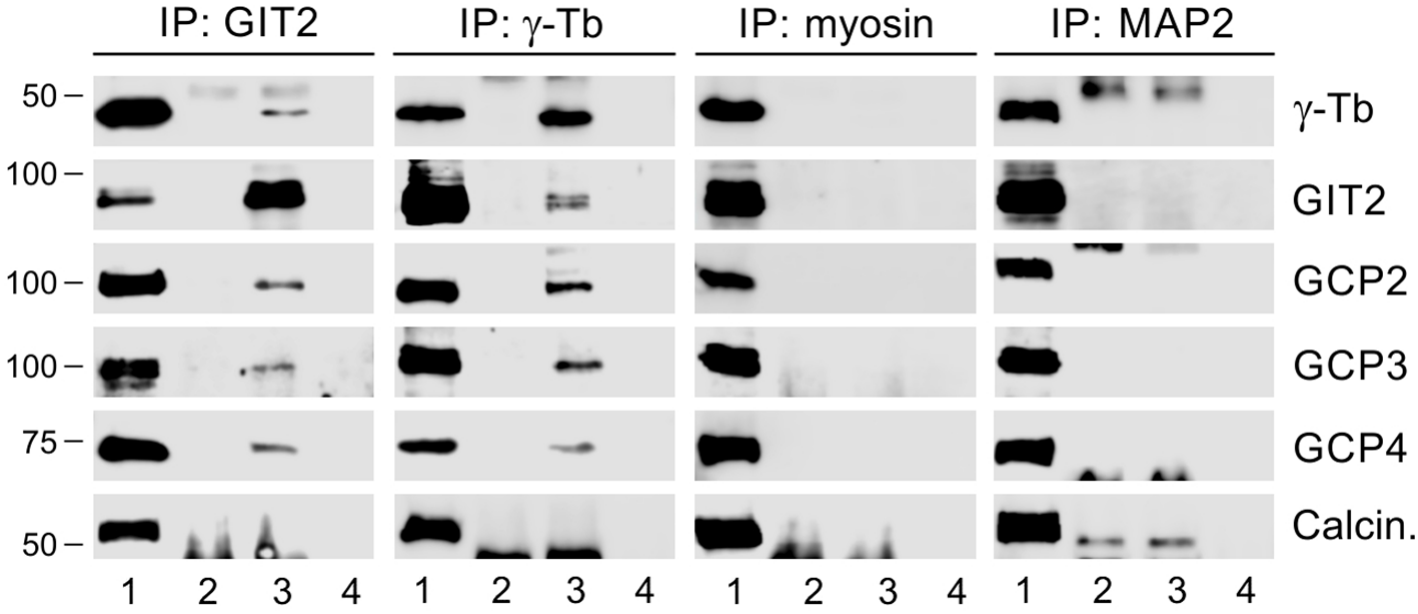
GIT2 interacts with γTuRC proteins of BMMCL. The whole-cell extracts were precipitated with immobilized rabbit Ab to GIT2, mouse mAb TU-31 (IgG2b) to γ-tubulin (γ-Tb), rabbit Ab to myosin (isotype control), or mouse mAb to MAP2 (IgG2b; isotype control). The blots were probed with Abs to γ-tubulin (γ-Tb), GIT2, GCP2, GCP3, GCP4, or calcineurin (Calcin., negative control). Load (*lane 1*), immobilized Abs without cell extracts (*lane 2*), precipitated proteins (*lane 3*), and Ab-free carriers incubated with cell extracts (*lane 4*).

In a previous study, we demonstrated that GIT1 forms complexes with γ-tubulin in BMMCL and acts as a positive regulator of centrosomal microtubule nucleation (30). To assess whether GIT2-long, referred to as GIT2 in the following text, may also play a role in the regulation of microtubule nucleation, we first performed immunoprecipitation experiments with whole-cell extracts from BMMCL. Using rabbit Ab to GIT2 and mouse Ab to γ-tubulin (IgG2b) for reciprocal precipitations, we revealed an association of GIT2 with γ-tubulin and the other γTuRC proteins such as GCP2, GCP3, and GCP4. There was no association with the Ser/Thr protein phosphatase calcineurin, which served as a negative control. Isotype controls for the immunoprecipitation experiments included rabbit anti-myosin and mouse anti-MAP2 (IgG2b) Abs (**Figure 2**).

To independently confirm the interaction between GIT2 and γTuRC proteins, we conducted immunoprecipitation experiments using cells that expressed TagRFP-tagged GIT2, as well as controls expressing only TagRFP. The anti-RFP Ab co-precipitated γ-tubulin, GCP2, GCP3, and GCP4 from cells expressing GIT2-TagRFP (**Supplementary Figure S2A**). In contrast, it did not co-precipitate these proteins from cells expressing TagRFP alone (**Supplementary Figure S2B**). Reciprocal precipitation with the anti-γ-tubulin Ab further validated the interaction of γ-tubulin with GIT2-TagRFP (**Supplementary Figure S2C**). To additionally validate the interaction between GIT2 and γTuRC proteins, we performed pull-down assays using GST-tagged γ-tubulin. Both GIT2 and β-PIX, which interacts with GIT2, as well as GCP2 and GCP4, demonstrated binding to GST-tagged γ-tubulin but not to GST alone. Calcineurin, used as a negative control protein, did not exhibit binding to GST-fusion proteins (**Supplementary Figure S2D**). These results collectively demonstrate that both endogenous and exogenous GIT2 form complexes with γTuRC proteins.

**Figure 3:**
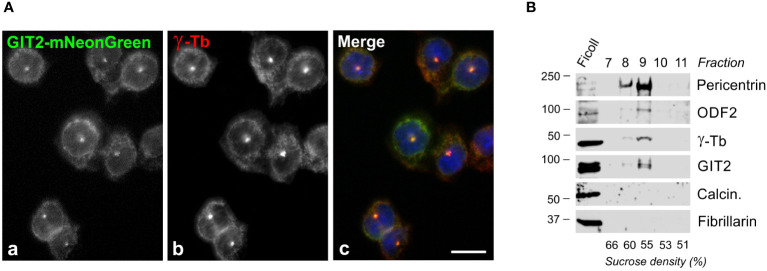
Centrosomal localization of GIT2 in BMMCL. **(A)** Cells expressing mNeonGreen-tagged GIT2 were fixed and stained with Ab to γ-tubulin. GIT2-mNeonGreen (a), γ-tubulin (b; γ-Tb), superposition of images (c; GIT2-mNeonGreen, green; γ-tubulin, red; DAPI, blue). Fixation Tx/F/M. Scale bar, 10 μm (a-c). **(B)** Association of endogenous proteins with purified centrosomes. Centrosomes concentrated by centrifugation onto a Ficoll cushion underwent purification through sucrose gradient centrifugation. The gradient was fractionated from the bottom. Individual fractions are marked on the top, and the sucrose density in each fraction is presented at the bottom. Blots were probed with Abs to pericentrin, ODF2, γ-tubulin (γ-Tb), GIT2, calcineurin (Calcin.), and fibrillarin.

Since γTuRCs play a crucial role in centrosomal microtubule nucleation, we conducted experiments to determine whether GIT2 localizes to the centrosome. Nevertheless, despite applying immunofluorescence microscopy with a limited selection of commercially available Abs directed towards GIT2, we were unable to detect GIT2 at the centrosomes. We, therefore, opted to express mNeonGreen-tagged GIT2 (GIT2-mNeonGreen) and mNeonGreen alone (used as a negative control) in BMMCL. Both proteins were expressed in cells at comparable levels (**Supplementary Figures S3A, C**). On fixed cells, GIT2-mNeonGreen was observed to localize at the interphase centrosome (**Figure 3A**) as well as at spindle poles in cells in metaphase and anaphase (**Supplementary Figure S3B**). In contrast, mNeonGreen alone did not exhibit detectable centrosomal localization (**Supplementary Figure S3D**). To independently verify the association of GIT2 with centrosomes, we isolated microtubule nucleation-competent centrosomes from BMMCL through gradient centrifugation. Upon visualization by double-label immunofluorescence microscopy, the isolated centrosomes appeared as distinct, brightly stained dots when labeled with Abs to γ-tubulin and pericentrin (**Supplementary Figure S4A**). Isolated centrosomes facilitated microtubule nucleation upon the addition of 8 μM porcine brain tubulin and 1 mM GTP (**Supplementary Figure S4B**). Immunoblot analysis of the isolated centrosomes revealed the presence of GIT2 in fractions containing centrosomal marker proteins, including pericentrin, outer dense fiber 2 (ODF2), and γ-tubulin. Conversely, in the same fractions, calcineurin and fibrillarin, representing cytosolic and nuclear markers, respectively, were not detected (**Figure 3B**). These findings collectively confirm that both endogenous and exogenous GIT2 is associated with centrosomes in BMMCL.

### Role of GIT2 in centrosomal microtubule nucleation and degranulation

**Figure 4:**
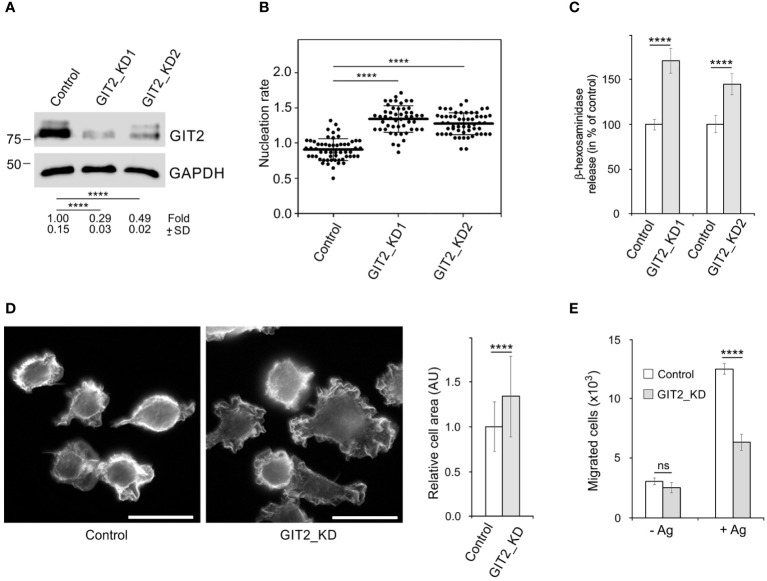
The effect of decreased expression of GIT2 on microtubule nucleation, Ag-induced degranulation and chemotaxis, and cell spreading. **(A)** Blots of whole-cell lysates from BMMCL probed with Abs to GIT2 and GAPDH (loading control). Control cells infected with the pLKO.1 vector containing non-target shRNA (Control, pLKO.1-NT), cells selected after depletion of GIT2 by shRNA-A (GIT2_KD1), or shRNA-B (GIT2_KD2). Numbers under the blot indicate relative amounts of GIT2 normalized to control cells and the amount of GAPDH in individual samples (fold). Values indicate mean ± SD (n = 5). **(B)** Comparison of microtubule nucleation rate (EB3 comets/min) in GIT2_KD1 and GIT2_KD2 cells, respectively, relative to control cells. Three independent experiments were conducted, with a minimum of 12 cells counted in each experiment. The dot plot depicts data for Control (pLKO.1-NT) (n = 57), GIT2_KD1 (n = 50), and GIT2_KD2 (n = 56), where bold and thin lines represent mean ± SD. **(C)** The degranulation in the controls (pLKO.1-NT; n = 3 or 4), GIT2_KD1 (n = 3), and GIT2_KD2 (n = 4) cells. The IgE-sensitized cells were activated by Ag (DNP-albumin; 100 ng/ml), and the degranulation was measured by β-hexosaminidase release. The data represent mean ± SD. Measured values (%): 24.41 ± 2.32 (Control 1), 42.18 ± 6.85 (GIT2_KD1), 21.19 ± 3.43 (Control 2), 30.64 ± 4.68 (GIT2_KD2). **(D)** Comparison of cell spreading in control (pLKO.1-NT) and GIT2_KD cells. Cells were incubated on fibronectin-coated coverslips for 45 min and thereafter fixed (F/Tx) and stained with rhodamine-phalloidin. Scale bar, 20 μm. Quantification of cell area is shown on the right. Three independent experiments (at least 81 cells counted in each experiment). Control (pLKO.1-NT) (n = 309), GIT2_KD (n = 319). Values indicate mean ± SD. **(E)** The chemotaxis in the control (pLKO.1-NT) and GIT2_KD cells. The cells were exposed to control medium (-Ag) or medium supplemented with Ag (+Ag; DNP-albumin; 250 ng/ml). The number of cells migrating into the lower well was determined 6 h after initiation of the experiment. The data represent mean ± SD from three experiments. **(B)** Statistical significance was assessed using one-way ANOVA with Dunnett’s multiple comparison test. **(A, C–E)** Two-tailed, unpaired Student’s t-test was conducted to determine statistical significance. ns, p > 0.05; ****, p < 0.0001.

To assess the role of GIT2 in centrosomal microtubule nucleation and degranulation, we generated GIT2-deficient cells, referred to as GIT2_KD1 and GIT2_KD2, using lentiviral vectors. As a control, we utilized cells containing the pLKO.1 vector with non-targeting shRNA (referred to as Control, PLKO.1-NT). A representative result of immunoblotting experiments following GIT2 depletion is depicted in **Figure 4A**. Unless specified otherwise, the following results are based on GIT2_KD1 cells, abbreviated as GIT2_KD. Time-lapse imaging of cells with EB3-mNeonGreen and counting the number of EB3 comets leaving centrosomes per time unit (nucleation rate) revealed increased centrosomal nucleation in quiescent GIT2_KD1 and GIT2_KD2 cells compared to the control (pLKO.1-NT) (**Figure 4B**). Upon activation of GIT2_KD cells by FcϵRI aggregation with Ag, centrosomal microtubule nucleation further increased (**Supplementary Figure S5A**). Accordingly, increased microtubule nucleation in GIT2_KD cells led to an increase in microtubule amount, which was further augmented in activated cells (**Supplementary Figure S5B**). Depletion of GIT2 resulted in enhanced degranulation, as assessed by the release of β-hexosaminidase (**Figure 4C**). This enhanced degranulation was inhibited by nocodazole (**Supplementary Figure S5C**). Additionally, GIT2 depletion led to an increased influx of Ca^2+^ (**Supplementary Figure S5D**). A previous study reported that the deletion of GIT2 in bone marrow-derived macrophages results in increased transcription of pro-inflammatory cytokines in stimulated cells (54). To further characterize GIT2_KD cells, we also analyzed the impact of GIT2 deficiency on the expression of cytokines and prostaglandins in activated BMMCL. The mRNA levels of cytokines such as tumor necrosis factor (TNFα; gene *Tnf*), interleukin 6 (IL-6; gene *Il6*), and interleukin-13 (IL-13; gene *Il13*) were elevated in GIT2_KD cells compared to the control, and this trend was mirrored in the mRNA levels of prostaglandin-endoperoxidase synthase 2 (COX-2; gene *Ptgs2*), which is crucial for prostaglandin production (**Supplementary Figure S5E**). These observations illustrate that the depletion of GIT2 leads not only to reported changes in cytokines but also to a significant increase in microtubule nucleation, microtubule amount, and degranulation dependent on microtubules.

### Role of GIT2 in BMMCL cell spreading and chemotaxis

Since GITs play a significant role in cell adhesion (55), we conducted a comparison of cell spreading between control and GIT2-depleted cells. Cells were incubated on fibronectin-coated coverslips for 40 min, fixed, and then stained with rhodamine-labeled phalloidin to visualize microfilaments at the cell periphery. The depletion of GIT2 resulted in a substantial increase in cell spreading. **Figure 4D** presents the typical staining of control and GIT2-depleted cells, along with the quantification of cell areas. This observation aligns with previous findings in human cervical epithelial HeLa cells, where GIT2 was shown to suppress cell spreading (56). We also investigated the impact of GIT2 on Ag-induced chemotaxis. The data presented in **Figure 4E** show that cells with reduced GIT2 displayed a notably diminished chemotactic response to Ag in comparison to the control cells.

### Preparation and characterization of cells without GIT2

To further explore the mechanisms by which GIT2 influences centrosomal microtubule nucleation, we generated GIT2-deficient BMMCL using CRISPR/Cas9 editing. A diagram outlining the mouse *Git2* gene depicts the targeting sites in exons 4 and 14 for the sgRNAs, enabling the efficient deletion of all GIT2 isoforms (**Supplementary Figure S6A**). We successfully established three distinct cell lines referred to as GIT2_KO1, GIT2_KO2, and GIT2_KO3. Genomic sequencing confirmed shifts in reading frames (**Supplementary Figure S6B**). GIT2 isoforms were undetectable by immunoblotting, and the depletion of GIT2 did not affect actin expression (**Supplementary Figure S6C**). Unless specified otherwise, the subsequent results pertain to GIT2_KO1 cells, abbreviated as GIT2_KO. The deletion of GIT2 did not have a profound impact on cell division. In comparison to control cells, there was no significant change in the number of viable cells in GIT2_KO (**Supplementary Figure S7A**). As expected, GIT2 deletion led to increased cell spreading, as evidenced by staining fixed cells with rhodamine-labeled phalloidin and quantifying cell area (**Supplementary Figure S7B**). The deletion of GIT2 did not have a significant impact on the expression of GIT1 (**Supplementary Figure S7C**).

**Figure 5:**
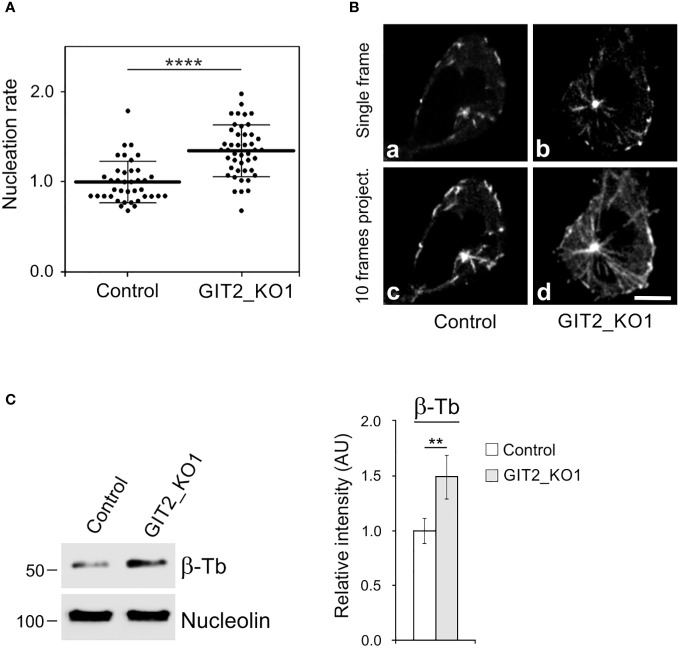
Microtubule nucleation and microtubule amount increases in *Git2* knockout cells. **(A)** Microtubule nucleation rate (EB3 comets/min) in GIT2_KO1 cells relative to controls. Three independent experiments were conducted, with a minimum of 9 cells counted in each experiment. The dot plot displays data for Control (n = 39) and GIT2_KO1 (n = 44), with bold and thin lines representing mean ± SD. Two-tailed, unpaired Student’s *t*-test was performed to determine statistical significance. ****, p < 0.0001. **(B)** Time-lapse imaging of control and GIT2_KO1 cells. Still images of EB3 (Single frame) and projection tracks of EB3 comets over 10 s (10 frames project.). Scale bar, 5 μm (a-d). **(C)** The Control or GIT2_KO1 cells were extracted in 0.2% Triton X-100 at 37°C, and detergent-insoluble fractions were analyzed by immunoblotting with Abs to β-tubulin (β-Tb) and nucleolin (loading control). Densitometric quantification of immunoblots is shown on the right. Relative intensity of β-tubulin normalized to control cells and to the amount of nucleolin. Values indicate mean ± SD (n = 5). Two-tailed, unpaired Student’s *t*-test was performed to determine statistical significance. **, p < 0.01, ****, p < 0.0001.

**Figure 6:**
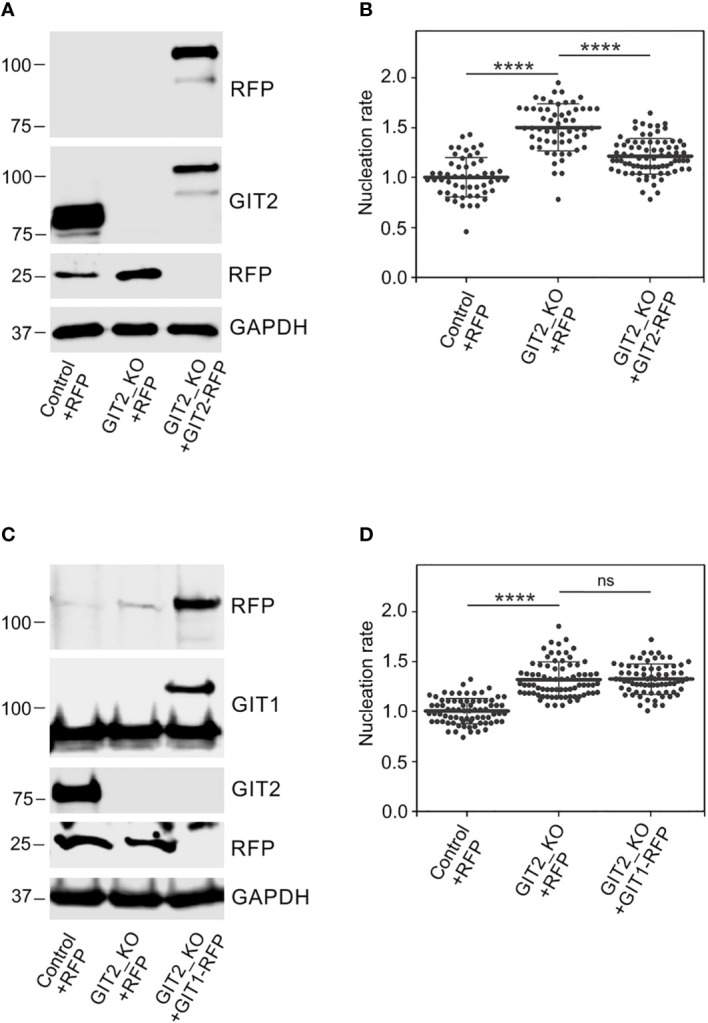
Phenotypic rescue of increased microtubule nucleation in *Git2* knockout cells. **(A)** Immunoblot analysis of GIT2 in whole-cell lysates from control cells expressing TagRFP (Control+RFP), GIT2_KO cells expressing TagRFP (GIT2_KO+RFP), and GIT2_KO cells rescued by GIT2-TagRFP (GIT2_KO+GIT2-RFP). The blots were probed with Abs to RFP and GIT2. GAPDH served as loading control. **(B)** Comparison of microtubule nucleation rate (EB3 comets/min) in GIT2_KO cells expressing TagRFP and GIT2-TagRFP, respectively, relative to control cells expressing TagRFP. Three independent experiments were conducted, with a minimum of 15 cells counted in each experiment. The dot plot illustrates data for Control+RFP (n = 53), GIT2_KO+RFP (n = 58), and GIT2_KO+GIT2-RFP (n = 78), with bold and thin lines indicating mean ± SD. **(C)** Immunoblot analysis of GIT1 in whole-cell lysates from control cells expressing TagRFP (Control+RFP), GIT2_KO cells expressing TagRFP (GIT2_KO+RFP), and GIT2_KO cells rescued by GIT1-TagRFP (GIT2_KO+GIT1-RFP). The blots were probed with Abs to RFP, GIT1, and GIT2. GAPDH served as loading control. **(D)** Comparison of microtubule nucleation rate (EB3 comets/min) in GIT2_KO cells expressing TagRFP and GIT1-TagRFP, respectively, relative to control cells expressing TagRFP. Three independent experiments were conducted, with a minimum of 15 cells counted in each experiment. The dot plot illustrates data for Control+RFP (n = 75), GIT2_KO+RFP (n = 77), and GIT2_KO+GIT1-RFP (n = 71), with bold and thin lines indicating mean ± SD. **(B, D)** Statistical significance was assessed using one-way ANOVA with Sidak’s multiple comparison test. ns, p > 0.05; ****, p < 0.0001.

Time-lapse imaging and counting of the number of EB3 comets showed increased centrosomal nucleation in GIT2_KO1 cells (**Figures 5A, B**), consistent with the findings in cells where GIT2 levels were reduced using shRNAs. This increase in microtubule nucleation was likewise observed in GIT2_KO2 and GIT2_KO3 cells. Consequently, the augmented microtubule nucleation also resulted in an increased microtubule amount (**Figure 5C**). To validate the specificity of the observed alterations in microtubule nucleation, we conducted rescue experiments by introducing GIT2-TagRFP or TagRFP alone into GIT2-KO cells (**Figure 6A**). The introduction of GIT2-TagRFP into GIT2-KO cells led to a decrease in microtubule nucleation, while the expression of TagRFP alone did not have such effect (**Figure 6B**). When GIT1-TagRFP was used for rescue experiments (**Figure 6C**), the extent of microtubule nucleation was unchanged, suggesting that GIT2 and GIT1 have different functions in microtubule nucleation (**Figure 6D**). Taken together, these data demonstrate that GIT2 negatively regulates centrosomal microtubule nucleation.

### Phosphorylation affects GIT2 centrosomal localization

**Figure 7:**
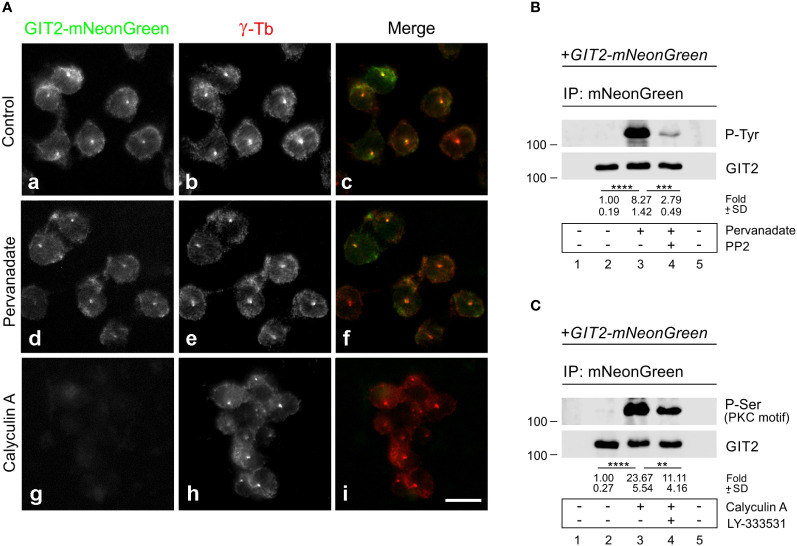
The effect of pervanadate and calyculin A on subcellular localization of GIT2-mNeonGreen. **(A)** GIT2_KO cells expressing mNeonGreen-tagged GIT2 were untreated (Control, a-c) or pretreated with pervanadate (d-f) or Calyculin A (g-i) and then fixed and stained with Ab to γ-tubulin (γ-Tb). GIT2-mNeonGreen (a, d, g), γ-tubulin (b, e, h), superposition of images (c, f, i; GIT2-mNeonGreen, green; γ-tubulin, red). Fixation Tx/F/M. Scale bar, 10 μm (a-i). **(B, C)** Immunoprecipitation experiments with Ab to mNeonGreen and the whole-cell extracts from GIT2_KO cells expressing GIT2-mNeonGreen. Immobilized Ab without cell extracts (*lane 1*), precipitated proteins (*lanes 2-4*), and Ab-free carriers incubated with cell extract (*lane 5*). **(B)** Cells pretreated with DMSO alone (*lane 3*) or with Src kinase family selective inhibitor PP2 (20 μM, 60 min; *lane 4*) before incubation with pervanadate (15 min). The blots were probed with Abs to phosphotyrosine (P-Tyr) and GIT2. Numbers under the blot indicate relative amounts of GIT2-mNeonGreen phosphorylated on tyrosine (P-Tyr) normalized to pervanadate untreated cells and the amount of precipitated GIT2-mNeonGreen in individual samples (fold). Values indicate mean ± SD (n = 4) **(C)** Cells pretreated with DMSO alone (*lane 3*) or with PKCβ selective inhibitor LY-333531 (10 μM, 30 min; *lane 4*) before incubation with Calyculin A (100 nM, 30 min). The blots were probed with Abs to P-Ser in PKC motif and GIT2. Numbers under the blot indicate relative amounts of GIT2-mNeonGreen phosphorylated on serine (P-Ser in PKC motif) normalized to Calyculin A untreated cells and the amount of precipitated GIT2-mNeonGreen in individual samples (fold). Values indicate mean ± SD (n = 5). Two-tailed, unpaired Student’s *t*-test was performed to determine statistical significance. **, p < 0.01, ***, p < 0.001; ****, p < 0.0001.

Previously, we reported that both Src family tyrosine kinases (10) and Ser/Thr conventional PKC kinases (cPKCs) (57) are involved in microtubule reorganization in activated mast cells. To determine whether phosphorylation of GIT2 might play a role in its centrosomal localization, we expressed GIT2-mNeonGreen in GIT2_KO cells and localized the tagged GIT2 in samples extracted with Triton X-100, fixed with formaldehyde, and postfixed by methanol. As expected, exogenous GIT2 was associated with interphase centrosomes (**Figure 7A**a-c). Cells were then treated with pervanadate or Calyculin A, potent inhibitors of tyrosine or Ser/Thr phosphatases, respectively, and examined for the localization of GIT2-mNeonGreen. Pretreatment of cells with pervanadate did not affect the centrosomal localization of exogenous GIT2 (**Figure 7A**d-f). In contrast, pretreating cells with 100 nM Calyculin A for 30 min resulted in cell rounding and the detachment of exogenous GIT2 from detergent-resistant cellular fraction, including centrosomes (**Figure 7A**g-i). The phosphorylation of GIT2-mNeonGreen was verified through immunoprecipitation using whole-cell 1% NP-40 extracts and Ab to mNeonGreen, and the blots were probed with Abs to phosphorylated amino acids. The Ab directed to P-Tyr verified phosphorylation of exogenous GIT2 by tyrosine kinases in pervanadate-treated cells. The level of phosphorylation was strongly suppressed by the Src family kinase-specific inhibitor (PP2) (**Figure 7B**). Likewise, the Ab recognizing P-Ser in PKC motifs confirmed phosphorylation of exogenous GIT2 by PKCs in Calyculin A-treated cells. The level of phosphorylation was partially suppressed by LY-333531, a cPKCs inhibitor that preferentially targets PKCβ isoforms (58) (**Figure 7C**). In control, GIT2_KO cells expressing γ-tubulin-mNeonGreen, tagged γ-tubulins were also observed at centrosomes (**Supplementary Figure S8Aa-c**). This localization remained unchanged after treatment with either pervanadate (**Supplementary Figure S8Ad-f**) or Calyculin A (**Supplementary Figure S8Ag-i**). Immunoblotting indicated that tagged γ-tubulin was phosphorylated by Src family tyrosine kinases (**Supplementary Figure S8B**) but not by PKCs (**Supplementary Figure S8C**; P-Ser, PKC motif). However, phosphorylation of tagged γ-tubulin on Ser/Thr was detected when probing the blot with Ab to P-(Ser/Thr)-Phe (**Supplementary Figure S8C**; P-Ser). Overall, these findings illustrate that phosphorylation of exogenous GIT2 on Ser/Thr, but not on Tyr, affects its subcellular localization. This suggests that centrosomal localization of GIT2 may be regulated by Ser/Thr phosphorylation and that cPKCs may play a role in this process.

### cPKCs phosphorylate GIT2 during activation of mast cells

**Figure 8:**
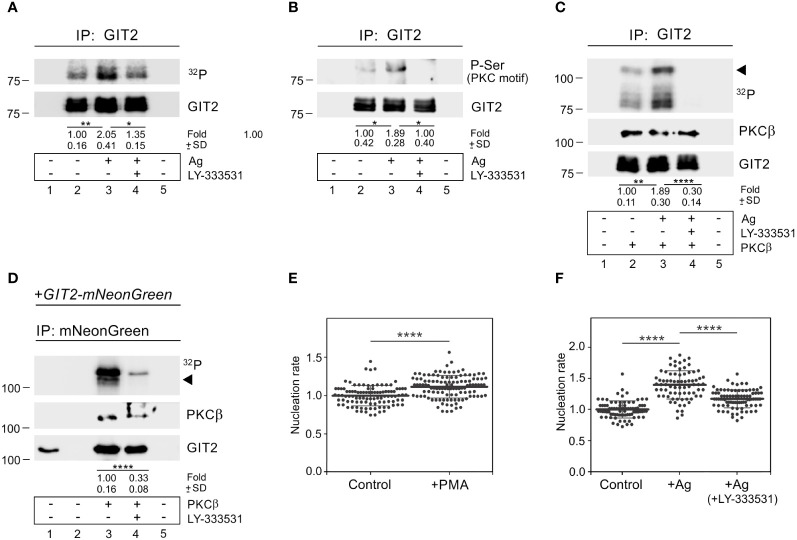
PKCβ phosphorylates GIT2 during Ag-induced activation and modulates microtubule nucleation. **(A–C)** Immunoprecipitation experiments with Ab to GIT2 and the whole-cell extracts from non-activated or Ag-activated cells. Immobilized Abs without cell extracts (*lane 1*), precipitated proteins (*lanes 2-4*), and Ab-free carriers incubated with cell extract (*lane 5*). Cells pretreated with DMSO alone (*lane 3*) or with PKCβ selective inhibitor LY-333531 (10 μM, 30 min; *lane 4*) before incubation with Ag (DNP-albumin; 100 ng/ml) for 5 min. **(A)** *In vitro* kinase assay after immunoprecipitation (^32^P). The blot was probed with Ab to GIT2. Numbers under the blot indicate relative amounts of phosphorylated GIT2 normalized to non-activated cells and the amount of precipitated GIT2 in individual samples (fold). Mean ± SD (n = 4). **(B)** The blots probed with Abs to phosphoserine (P-Ser) in PKC motif and GIT2. Numbers under the blot indicate relative amounts of phosphorylated GIT2 (P-Ser in PKC motif) normalized to non-activated cells and the amount of precipitated GIT2 in individual samples (fold). Mean ± SD (n = 4). **(C)** *In vitro* kinase assay on immunoprecipitated material in the presence of exogenous active PKCβ (^32^P). The blots were probed with Abs to PKCβ and GIT2. The arrowhead point to exogenous PKCβ. Numbers under the blot indicate relative amounts of GIT2 phosphorylated by PKCβ (^32^P) normalized to non-activated cells and the amount of precipitated GIT2 in individual samples (fold). Mean ± SD (n = 4). **(D)** Immunoprecipitation experiments with Ab to mNeonGreen and the whole-cell extracts from GIT2_KO cells expressing GIT2-mNeonGreen, followed by *in vitro* kinase assay in the presence of exogenous active PKCβ (^32^P). Load (*lane 1*), immobilized Ab without cell extract (*lane 2*), precipitated proteins (*lanes 3-4*), and Ab-free carrier incubated with cell extract (*lane 5*). Cells pretreated with DMSO alone (*lane 3*) or with PKCβ selective inhibitor LY-333531 (10 μM, 30 min; *lane 4*). The blots were probed with Abs to PKCβ and GIT2. The arrowhead points to exogenous PKCβ. Numbers under the blot indicate relative amounts of GIT2-mNeonGreen phosphorylated by PKCβ (^32^P) normalized to LY-333531 untreated cells and the amount of precipitated GIT2-mNeonGreen in individual samples (fold). Mean ± SD (n = 5). **(E)** Comparison of microtubule nucleation rate (EB3 comets/min) in cells with activated cPKCs, relative to control cells. Cells were pretreated with DMSO alone (Control) or with PKC activator PMA (1 μM for 30 min). Three independent experiments were conducted, with a minimum of 27 cells counted in each experiment. The dot plot depicts data for Control (n = 102) and cPKCs activated cells (n = 115), where bold and thin lines represent mean ± SD. **(A–E)** Statistical significance was assessed using a two-tailed, unpaired Student’s t-test. **(F)** The effect of PKCβ inhibition on microtubule nucleation in Ag-activated cells. Cells were activated by FcεRI aggregation by Ag at a concentration of 100 ng/ml for 10 min. Microtubule nucleation rate (EB3 comets/min) in activated cells (+Ag) and cells activated in the presence of 10 μM inhibitor (+Ag+LY-333531) relative to control cells (Control). Three independent experiments were conducted, with a minimum of 20 cells counted in each experiment. The dot plot depicts data for Control (n = 80), Ag activated (n = 75), and Ag activated + LY-333531 cells (n = 84), where bold and thin lines represent mean ± SD. One-way ANOVA with Sidak’s multiple comparison test was performed to determine statistical significance. *, p < 0.05; **, p < 0.01; ****, p < 0.0001.

To assess whether GIT2 is phosphorylated during BMMCL activation, we performed an *in vitro* kinase assay using non-activated cells and cells activated by Ag aggregation of FcεRI. These cells were immunoprecipitated with anti-GIT2 Ab. The increased phosphorylation of GIT2 in activated cells was partially suppressed by the cPKCs inhibitor LY-333531 (**Figure 8A**). The augmented phosphorylation of GIT2 in activated cells by PKC was further verified by immunoblotting with the Ab that recognizes P-Ser in PKC motifs. The level of phosphorylation was reduced in the presence of the cPKC inhibitor (**Figure 8B**). When we conducted the *in vitro* kinase assay in the presence of purified active PKCβ, GIT2 was phosphorylated, and its phosphorylation was significantly reduced by LY-333531, which also inhibited the autophosphorylation of exogenous PKCβ (**Figure 8C**). Finally, in the *in vitro* kinase assay with PKCβ, we employed whole-cell extracts of GIT2_KO cells expressing GIT2-mNeonGreen for immunoprecipitation with anti-mNeonGreen Ab. In this case as well, exogenous GIT2 was phosphorylated by PKCβ, and its phosphorylation was inhibited by the cPKC inhibitor (**Figure 8D**). To investigate whether activated PKCs have any effect on microtubule nucleation, we stimulated PKCs using PMA, which mimics the action of diacylglycerol and is thus involved in the activation of conventional and novel PKCs (59). Treating BMMCL with PMA resulted in an increase in *de novo* centrosomal microtubule nucleation (**Figure 8E**). Next, we examined the effect of PKCβ inhibition on microtubule nucleation. While the activation of cells by Ag led to an increase in microtubule nucleation, this effect was significantly attenuated in the presence of LY-333531 (**Figure 8F**). These results collectively demonstrate that GIT2 is phosphorylated during Ag-induced activation of BMMCL, and cPKCs involved in modulating microtubule nucleation may participate in regulating GIT2 phosphorylation levels in this process.

## Discussion

The initiation of signaling pathways occurs when mast cells are activated through the crosslinking of IgE bound to FcϵRI. This leads to Ca^2+^ influx, rapid cytoskeletal reorganization, degranulation, and the synthesis of new mediators. Previous studies have reported that mast cell activation stimulates microtubule formation (9, 10). The increased microtubule formation in activated cells may be attributed to changes in microtubule dynamics and the stabilization of the plus ends of dynamic microtubules. Alternatively, it could result from an increase in centrosomal or non-centrosomal microtubule nucleation (6). It is also possible that a combination of both mechanisms applies. The intricate molecular mechanisms controlling microtubule reorganization have only recently begun to be understood. In this study, we demonstrate, to the best of our knowledge, for the first time, that Ag-induced activation of mast cells increases both the amount of microtubules and centrosomal microtubule nucleation. Furthermore, we reveal that the adaptor signaling protein GIT2 plays an important role in this process. GIT2 associates with γTuRCs and acts as a negative regulator of microtubule nucleation. Its centrosomal localization is influenced by phosphorylation, and during Ag-induced activation, GIT2 undergoes phosphorylation by the Ca^2+^-dependent PKC. Our study proposes a novel regulatory mechanism for microtubule nucleation in activated mast cells.

It is known that mast cells can be stimulated to degranulate not only by the crosslinking of FcεRI-bound IgE but also by activation via various other receptors (2, 60). It has been reported that Ag-induced degranulation is slow and persistent, whereas degranulation via activation of the Mas-related G protein-coupled receptor B2 (MRGPRB2) leads to rapid and transient degranulation (61). Interestingly, the binding of neuropeptide SP to the MRGPRB2 in BMMCL not only triggered degranulation comparable to that in Ag-stimulated cells (**Supplementary Figure S9A**) but also slightly increased microtubule nucleation at 10 min of activation (**Supplementary Figure S9B**). However, the increase was not as pronounced as in Ag-stimulated cells (**Figure 1**), and nucleation was unchanged at 3 min of activation with SP. It is possible that different signaling pathways in activated mast cells converge at centrosomes, where they modulate microtubule nucleation.

Multiple lines of evidence support the involvement of GIT2 in the modulation of centrosomal microtubule nucleation. First, reciprocal immunoprecipitation experiments confirmed an interaction between GIT2 and γTuRC proteins, which are essential for microtubule nucleation. Second, γTuRC proteins are associated with TagRFP-tagged GIT2. Third, pull-down assays with whole-cell extracts showed that GIT2 binds to GST-tagged γ-tubulin. Fourth, mNeonGreen-tagged GIT2 was localized on centrosomes, and endogenous GIT2 was associated with isolated centrosomes. Finally, depletion or deletion of GIT2 enhanced microtubule nucleation, and GIT1 did not rescue the microtubule nucleation phenotype in GIT2_KO cells. These findings strongly suggest that GIT2 serves as a specific negative regulator of centrosomal microtubule nucleation, playing an opposing role to GIT1, which acts as a positive regulator (30, 31). The increased microtubule nucleation observed in GIT2-depleted cells was associated with an augmented microtubule amount and enhanced Ag-induced degranulation depending on microtubules. In contrast, reduced microtubule nucleation in GIT1-depleted cells was linked to a decreased γ-tubulin level in centrosomes and reduced degranulation (30). Thus, GIT1 and GIT2 have distinct and non-redundant roles in centrosomal microtubule nucleation in BMMLC. The centrosomal presence of both GIT1 and GIT2 has been demonstrated in mouse embryonic fibroblasts NIH3T3 (29). Our prior work has revealed that GIT1 is associated with centrosomes in interphase BMMCL (30), and in this study, we establish that GIT2 is also associated with centrosomes in both interphase and mitotic BMMCL. Further investigations are warranted to identify centrosomal proteins that interact with GIT2. Interestingly, the LC-MS/MS analysis of proteins co-immunoprecipitating with GIT2 in mouse pancreatic whole-cell lysates revealed the presence of CDK5RAP2 protein (62), which is known to be involved in both centrosome targeting and the activation of γTuRCs (24). It is worth noting that other GAPs for Arf small GTPases may also play a role in modulating microtubule nucleation (25). For instance, ELMOD2, a GAP for GTPase Arl2 (Arf-like protein 2), has been found to associate with γTuRCs. The deletion of ELMOD2 in cell lines was shown to inhibit the recruitment of γTuRCs to centrosomes and microtubule nucleation (63, 64). Balancing the activities of GITs could potentially regulate centrosomal microtubule nucleation, which, in turn, could impact the number of microtubules available for the transport of granules from the cell interior in Ag-activated mast cells.

The distinct regulatory roles of GIT2 and GIT1 extended beyond microtubule nucleation and were also evident in Ag-induced chemotaxis. We have reported previously that GIT1 depletion enhances chemotaxis (30), while the current study demonstrates that GIT2 depletion leads to the inhibition of chemotaxis to Ag. Interestingly, studies on *Git2* ^-/-^ neutrophils have reported impaired chemotaxis to formyl peptide (fMPL), C5a, and IL-8 (65). Conversely, *Git2* ^-/-^ thymocytes exhibited increased migration to chemokines CXCL12 (SDF-1) and CCL25 (66). Furthermore, the depletion of GIT2 in RBL-2H3 cells did not affect chemotaxis to fMPL (67). These differences observed in various studies may be attributed to variations in the experimental cell models, as it is becoming evident that the effects of GIT1 and GIT2 on cell motility are cell-type-dependent (68). Moreover, differences in the chemoattractants used in the assays and variations in the signaling machinery responsible for sensing direction could also play a role. Microtubules have long been associated with cell motility, although their specific roles in this process can vary among different cell types (69). The precise mechanisms underlying microtubule involvement in mast cell motility remain poorly understood. It has been proposed that microtubules normally act to restrain cell movement and specify directionality (70). Findings from various studies indicate that microtubule destabilization has a pro-migratory effect when amoeboid migration is favored (71). This could potentially explain the differential effects observed in microtubule nucleation and chemotaxis when GIT1 (30) or GIT2 were depleted in mast cells. Considering that both GIT1 and GIT2 influence actin-dependent processes, including cell motility (28), variations in chemotaxis toward Ag could arise from alterations in both microtubules and the actin cytoskeleton. Interestingly, diaphanous-related formin 1 (mDia), with actin-nucleation activity, has been reported to coordinate in mast cells the inhibition of secretion with enhanced chemotaxis (72). Similarly, GITs coordinate microtubule nucleation and microtubule-dependent degranulation with chemotaxis. This indicates that both cytoskeletal systems are simultaneously regulated in mast cells.

The disparities observed between GIT1 and GIT2 in their regulation of microtubule nucleation, degranulation, and chemotaxis in BMMCL suggest that the two GIT isoforms control these cellular processes through distinct mechanisms. Previous studies have provided evidence that, despite sharing a 61% amino acid sequence identity and 70% similarity in mouse GIT1 (tv1) and GIT2 (tv1), as well as possessing the same domain structure (28), GIT isoforms can regulate some cellular functions through different mechanisms. GIT1 and GIT2 have been found to exhibit non-redundant functions in cell spreading and focal adhesion (56). Furthermore, GIT1 and GIT2 have been shown to modulate distinct subsets of signaling pathways during chemotaxis in RBL-2H3 cells (67). Additionally, GIT1 and GIT2 proteins have been demonstrated to affect synaptic strength in neurons by enhancing exocytosis efficiency through distinct mechanisms (73).

Phosphorylation maps of GITs have revealed multiple sites with phosphorylated tyrosine, serine, or threonine; however, in most cases, the corresponding upstream kinases and the functional significance of phosphorylation remain unknown. Analysis of phosphorylation sites on human GIT1, detected by mass spectrometry, predicted two potential phosphorylation sites for PKCs (74). It is known that cPKCs, which are activated by Ca^2+^ and diacylglycerol, play a crucial role in mast cell degranulation and dynamic morphological changes of the actin cytoskeleton (75). There are four isoforms of cPKCs: PKCα, PKCβI, PKCβII, and PKCγ (76). Previously, we found that among cPKCs in BMMLCs, PKCβ isoforms are the most prominent (57). To investigate whether cPKCs might play a role in GIT2 phosphorylation, we conducted *in vitro* kinase assays using recombinant PKCβ and the inhibitor LY-333531, which preferentially suppresses the activities of PKCβ isoforms (58). Our results showed that mouse GIT2 is phosphorylated by PKCβ during Ag-induced activation. Furthermore, inhibiting cPKCs with LY-33351 during activation reduced microtubule nucleation, while activating PKCs with PMA led to increased nucleation. This suggests that cPKCs may also play a role in modulating centrosomal microtubule nucleation. PKCβII has been shown to play an important role in the centrosomes of various cell types, where it interacts with the centrosomal protein pericentrin. Disrupting the pericentrin-PKC interaction results in the disorganization of centrosomal microtubules. However, the specific target of pericentrin-tethered PKCβII has not been identified (77). The importance of PKCβ in microtubule reorganization has also been demonstrated in human T lymphocytes following the crosslinking of the cell surface CD44 receptor. Furthermore, after activation, PKCβ relocates to centrosomes (78). Accumulation of PKCβI in centrosomes has also been reported in peripheral blood T lymphocytes treated with PMA (79). We have previously demonstrated that inhibiting cPKCs in Ag-activated BMMCL led to a reduction in both microtubule formation and degranulation (57). These findings suggest that PKCs in mast cells may play a crucial role not only in regulating the actin cytoskeleton but also in the control of microtubule nucleation and organization. The precise mechanism through which cPKCs modulate microtubule nucleation, whether by phosphorylating GIT2 or other key proteins involved in centrosomal microtubule nucleation, requires further investigation, as does the identification of the target sequence for PKCβ on GIT2 and its role in microtubule nucleation and degranulation. It also remains unclear whether PKCβ-mediated phosphorylation of GIT2 in Ag-stimulated cells leads to dissociation of GIT2 from the centrosome.

Our results also showed that GIT2 is phosphorylated at tyrosine by Src family kinases. The importance of tyrosine phosphorylation in GIT2 for its functions has been documented in several studies. The phosphorylation of GIT2 at tyrosine residues 286, 392, and 592 by Src/FAK has been documented as essential for its interaction with paxillin at focal adhesions (80). Additionally, phosphorylation at Y392 enables GIT2 to associate with the SH2-SH3 adaptor proteins Nck1 and Nck2 (81, 82). Besides, tyrosine phosphorylation of GIT1 is required for its intramolecular conformational changes and release of autoinhibitory interactions (83). Tyrosine phosphorylation of GIT2 could potentially influence both its activation and its interaction with the other signaling molecules.

GIT proteins can be differentially phosphorylated by kinases. It has been reported that p21 protein (Cdc42/Rac)-activated kinase 1 (PAK1) phosphorylates centrosomal GIT1 (Ser 517) in a region where GIT1 and GIT2 are more divergent (29). We have shown that PAK1 acts as a positive regulator of microtubule nucleation, similar to GIT1 (31). Since phosphorylation plays an important role in regulating the functions of GIT proteins (27), one of the reasons for the differential regulation of centrosomal microtubule nucleation by GIT1 and GIT2 during Ag-induced activation of BMMCL might be the differential phosphorylation of GIT isoforms by the concerted action of Tyr and Ser/Thr kinases activated in distinct signaling pathways.

In conclusion, our data indicate that Ag-induced mast cell activation leads to enhanced centrosomal microtubule nucleation, and the GIT2 adaptor protein plays a significant role in this process. GIT2 interacts with γTuRCs, associates with centrosomes, and acts as a negative regulator of microtubule nucleation. It is likely that through these actions, GIT2 is involved in regulating critical processes in mast cell physiology such as Ag-induced degranulation and chemotaxis. Altering microtubule organization via regulators of microtubule nucleation could introduce innovative strategies for treating inflammatory and allergic diseases.

## Data availability statement

The original contributions presented in the study are included in the article/**Supplementary Material**, further inquiries can be directed to the corresponding author/s.

## Ethics statement

Ethical approval was not required for the studies on animals in accordance with the local legislation and institutional requirements because only commercially available established cell lines were used.

## Author contributions

VSu: Conceptualization, Investigation, Methodology, Validation, Visualization, Writing – original draft, Writing – review & editing. VSl: Data curation, Formal Analysis, Investigation, Validation, Visualization, Writing – review & editing. TS: Data curation, Formal Analysis, Investigation, Methodology, Visualization, Writing – review & editing. ED: Investigation, Methodology, Visualization, Writing – review & editing. VV: Investigation, Writing – review & editing. LD: Investigation, Writing – review & editing. OS: Supervision, Writing – review & editing. PD: Conceptualization, Funding acquisition, Investigation, Project administration, Supervision, Writing – original draft, Writing – review & editing.
