# Supplementary material for: Regulation of microtubule nucleation in mouse bone marrow-derived mast cells by ARF GTPase-activating protein GIT2
Source: Front Immunol. 2024 Feb 2;15:1321321. doi: 10.3389/fimmu.2024.1321321 (PMC10870779; doi:10.3389/fimmu.2024.1321321)
Supplement: Supplementary file 1 [file DataSheet_1.zip › Figure S1.pdf]

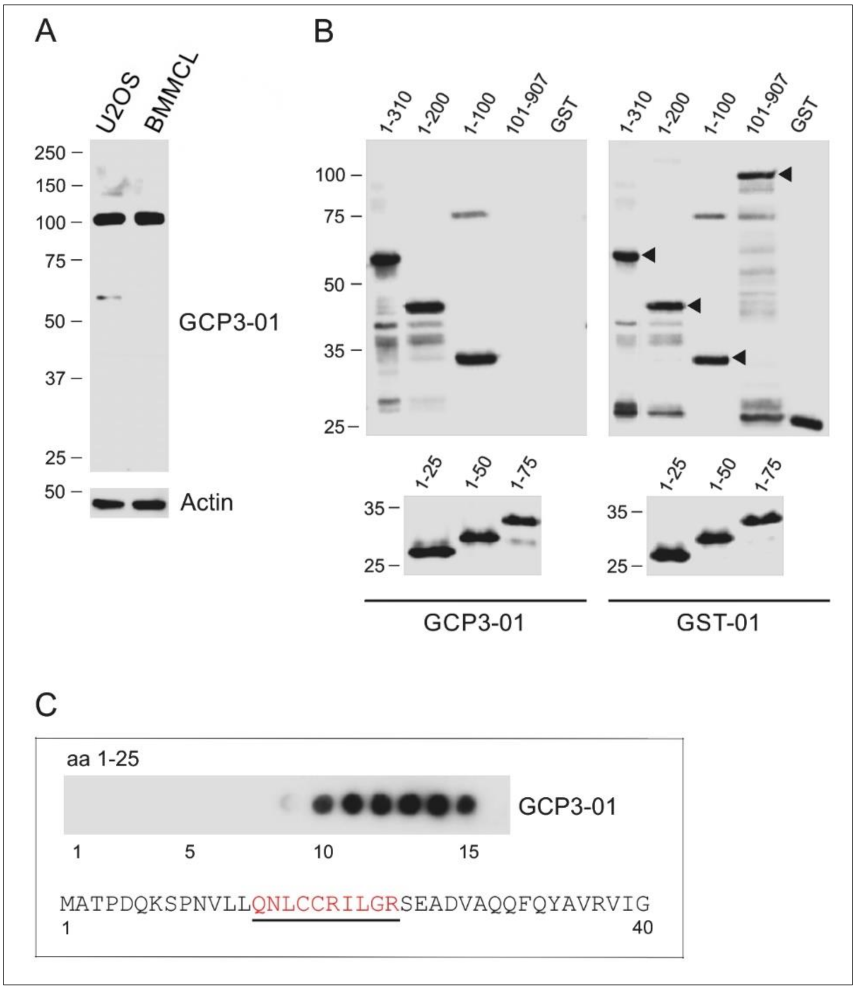

Figure S1. Characterization of the mouse monoclonal Ab GCP3-01 to GCP3. **(A)** Immunoblot analysis of whole cell lysates of human (U2OS) and mouse (BMMCL) cell lines with Abs to GCP3 (GCP3-01) and actin (loading control). **(B)** Reactivity of GCP3-01 with GST-tagged human GCP3 fragments or GST alone (left panels). The positions of full-length GST fusion proteins are marked with the mouse monoclonal Ab GST-01 to GST (black arrowheads in right upper panel). **(C)** Identification of the epitope of GCP3-01 by epitope mapping. Sixteen synthetic overlapping peptides (10-mer peptides, overlap 9 aa) covering the aa sequence 1-25 of human GCP3 were prepared by SPOT synthesis and then covalently bound to the membrane. The position of the epitope is indicated in red.
