# Supplementary material for: Regulation of microtubule nucleation in mouse bone marrow-derived mast cells by ARF GTPase-activating protein GIT2
Source: Front Immunol. 2024 Feb 2;15:1321321. doi: 10.3389/fimmu.2024.1321321 (PMC10870779; doi:10.3389/fimmu.2024.1321321)
Supplement: Supplementary file 1 [file DataSheet_1.zip › Figure S2.pdf]

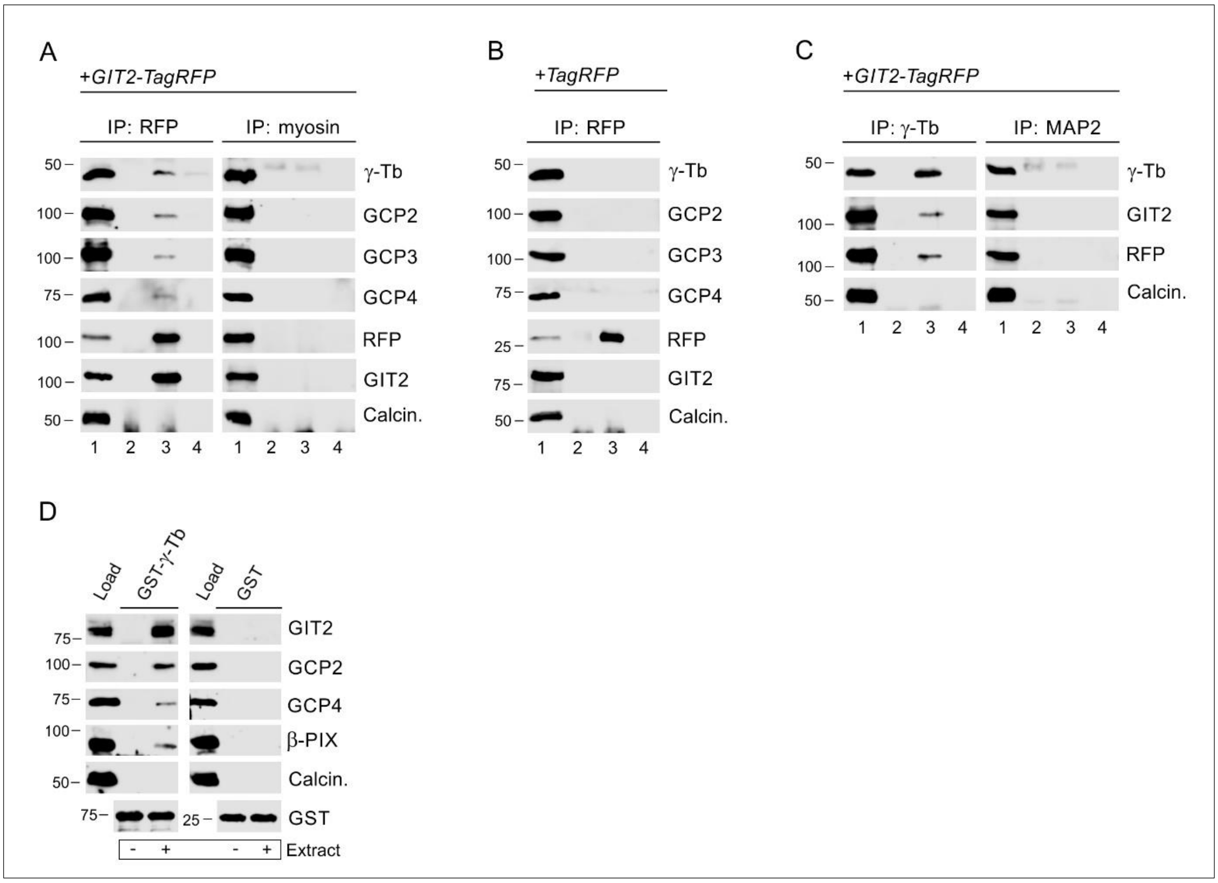

Figure S2. Exogenous GIT2 interacts with γTuRC proteins. **(A-C)** Immunoprecipitation experiments with the whole-cell extracts from BMMCL expressing TagRFP-tagged GIT2 (GIT2-TagRFP) or TagRFP alone. **(A-B)** Precipitation with immobilized rabbit Ab to RFP or rabbit Ab to myosin (isotype control). **(C)** Precipitation with immobilized mouse mAb TU-31 (IgG2b) to γ-tubulin (γ-Tb) or mouse mAb to MAP2 (IgG2b; isotype control). Blots were probed with Abs to γ-tubulin (γ-Tb), GCP2, GCP3, GCP4, RFP, GIT2, and calcineurin (Calcin.; negative control). (**D)** Pull-down assay with GST-tagged γ-tubulin. Immobilized GST-fusion protein or GST alone were incubated with whole-cell extracts from BMMCL (Load). Blots of bound proteins were probed with Abs to GIT2, GCP2, GCP4, β-PIX (positive control), GIT2, calcineurin (Calcin.; negative control), and GST.
