# Supplementary material for: Regulation of microtubule nucleation in mouse bone marrow-derived mast cells by ARF GTPase-activating protein GIT2
Source: Front Immunol. 2024 Feb 2;15:1321321. doi: 10.3389/fimmu.2024.1321321 (PMC10870779; doi:10.3389/fimmu.2024.1321321)
Supplement: Supplementary file 1 [file DataSheet_1.zip › Figure S3.pdf]

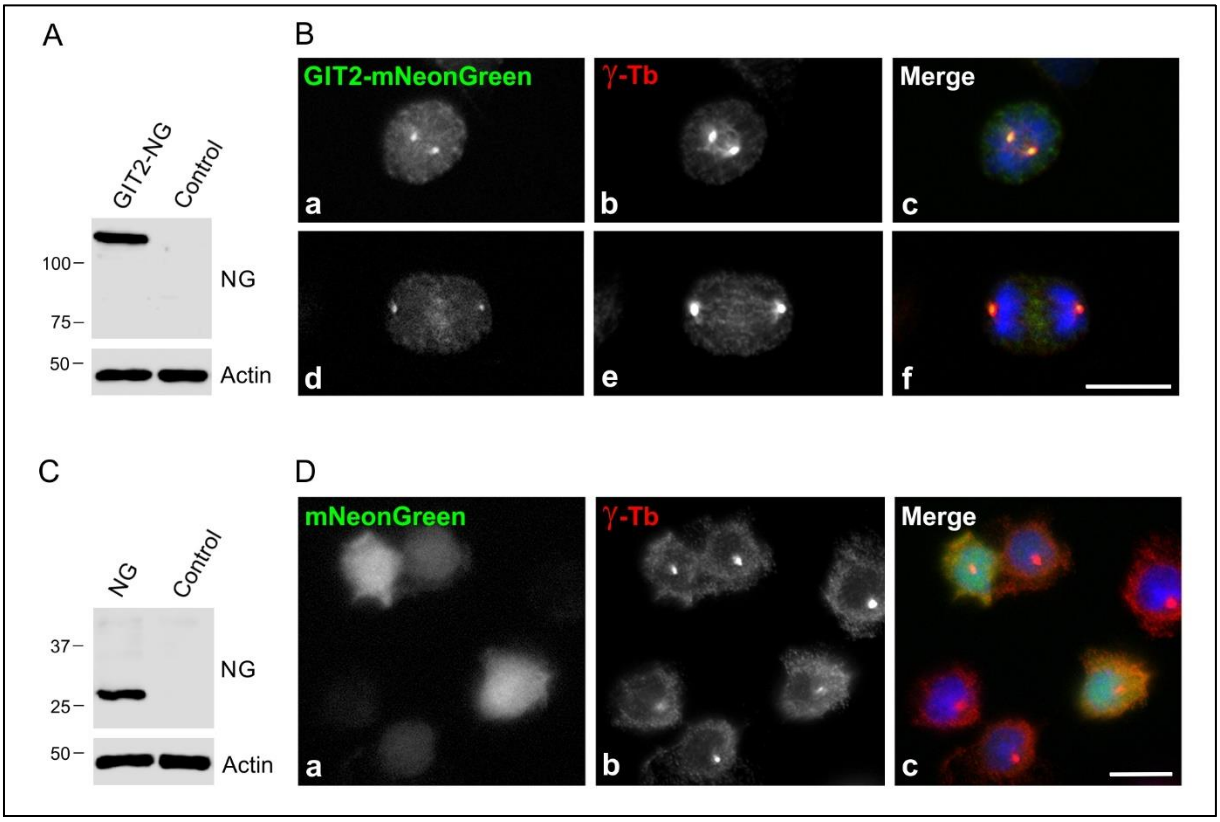

Figure S3. Expression and subcellular distribution of GIT2-mNeonGreen and mNeonGreen in BMMCL. **(A)** Immunoblot analysis of whole-cell lysates from cells expressing GIT2-mNeonGreen (GIT2-NG). As control served non-transfected cells. Blot was probed with Abs to mNeonGreen (NG) and actin (loading control). **(B)** Mitotic cells expressing GIT2-NG mNeonGreen were fixed and stained with Ab to γ-tubulin (γ-Tb). Prometaphase (a-c), anaphase (d-f). mNeonGreen (a, d), γ-tubulin (b, e), superposition of images (c, f; GIT2-mNeonGreen, green; γ-tubulin, red; DAPI, blue). Fixation Tx/F/M. Scale bar, 10 μm (a-f). **(C)** Immunoblot analysis of whole-cell lysates from cells expressing mNeonGreen (NG). As control served non-transfected cells. Blot was probed with Abs to mNeonGreen (NG) and actin (loading control). **(D)** Cells expressing mNeonGreen were fixed and stained with Ab to γ-tubulin. mNeonGreen (a), γ-tubulin (b; γ-Tb), superposition of images (c; mNeonGreen, green; γ-tubulin, red; DAPI, blue). Fixation F/Tx/M. Scale bar, 10 μm (a-c).
