# Supplementary material for: Regulation of microtubule nucleation in mouse bone marrow-derived mast cells by ARF GTPase-activating protein GIT2
Source: Front Immunol. 2024 Feb 2;15:1321321. doi: 10.3389/fimmu.2024.1321321 (PMC10870779; doi:10.3389/fimmu.2024.1321321)
Supplement: Supplementary file 1 [file DataSheet_1.zip › Figure S4.pdf]

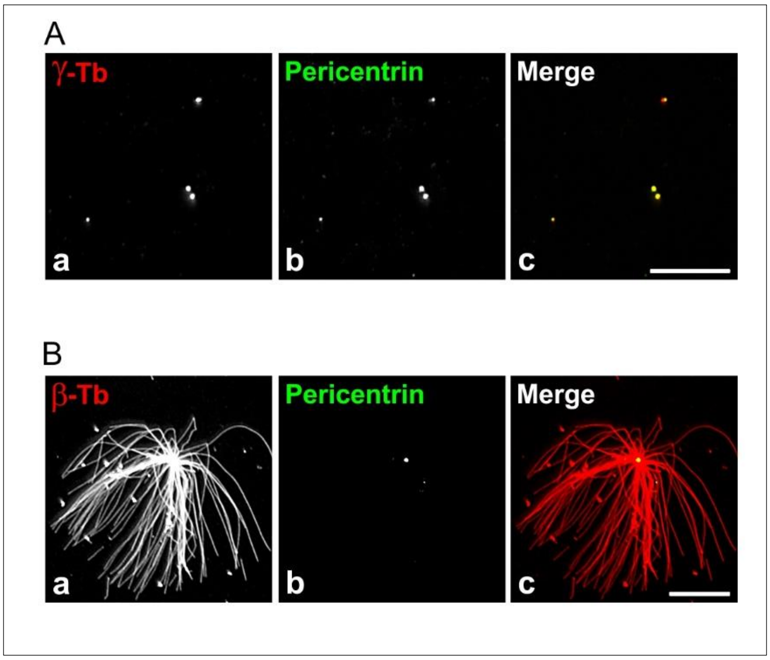

Figure S4. Centrosomes isolated from BMMCL promote microtubule nucleation. **(A)** Immunostaining of centrosomes isolated by sucrose gradient centrifugation. Centrosomes were pelleted on a coverslip, fixed and double-label stained with Abs to to γ-tubulin and pericentrin. γ-Tubulin (a; γ-Tb), pericentrin (b), superposition of images (c, γ-tubulin, red; pericentrin, green). Fixation methanol. Scale bar, 10 μm. (a-c). **(B)** Functional assay of isolated centrosomes. Centrosomes were incubated in suspension with 8 μM tubulin in the presence of 1mM GTP for 20 min at 37°C and thereafter fixed by glutaraldehyde, pelleted on a coverslip, postfixed by methanol (Fixation G/M) and double-label stained with Abs to β-tubulin and pericentrin. β-Tubulin (a; β-Tb), pericentrin (b), superposition of images (c, β-tubulin, red; pericentrin, green). Scale bar, 10 μm (a-c).
