# Supplementary material for: Regulation of microtubule nucleation in mouse bone marrow-derived mast cells by ARF GTPase-activating protein GIT2
Source: Front Immunol. 2024 Feb 2;15:1321321. doi: 10.3389/fimmu.2024.1321321 (PMC10870779; doi:10.3389/fimmu.2024.1321321)
Supplement: Supplementary file 1 [file DataSheet_1.zip › Figure S5.pdf]

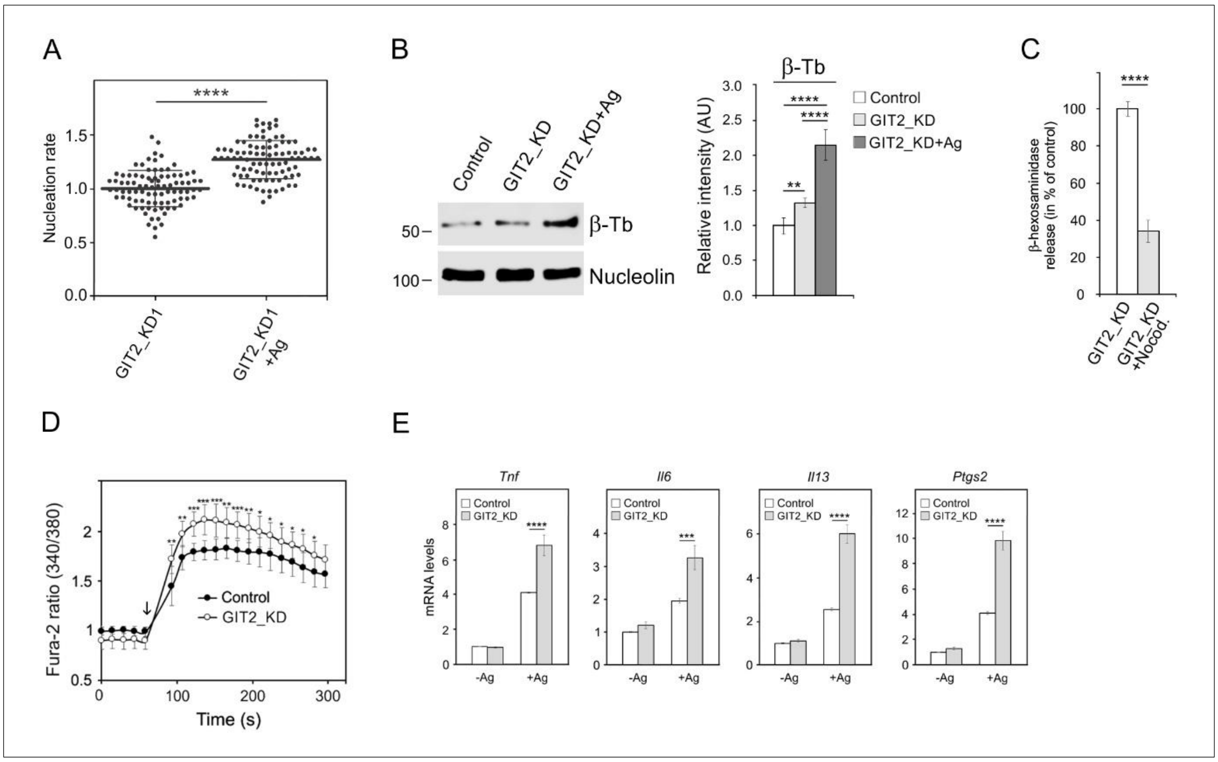


**Figure S5.** Characterization of cells with depleted level of GIT2. **(A)** Microtubule nucleation rate (EB3 comets/min) in activated GIT2_KD1 cells (+Ag) relative to non-activated GIT2_KD1 cells (Control). IgE-sensitized cells were activated by FcεRI aggregation by Ag at concentration 100 ng/ml for 10 min. Three independent experiments (at least 20 cells counted in each experiment). Control (n = 89), +Ag (n = 90). The bold and thin lines within the dot plot represent mean ± SD. **(B)** Comparison of microtubule amount in activated (+Ag) and non-activated GIT2_KD cells relative to the control (pLKO.1-NT). The Control or GIT2_KD cells were extracted in 0.2% Triton X-100 at 37°C, and detergent-insoluble fractions were analyzed by immunoblotting with Abs to β-tubulin (β-Tb) and nucleolin (loading control). Densitometric quantification of immunoblots is shown on the right. Relative intensity of β-tubulin normalized to control cells and to the amount of nucleolin. Values indicate mean ± SD (n = 5). **(C)** Comparison of degranulation in the GIT2_KD cells (n = 3) and GIT2_KD cells pre-treated with nocodazole (n = 3). The IgE sensitized cells were activated by Ag (DNP-albumin; 100 ng/ml), and the degranulation was measured by β-hexosaminidase release. The data represent mean ± SD. Measured values (%): 40.85 ± 2.05 (GIT2_KD), 13.83 ± 2.03 (GIT2_KD + nocodazole). **(D)** The intracellular $Ca^{2+}$ mobilization during cell activation in control and GIT2_KD cells. IgE sensitized cells were loaded with Fura-2-acetoxymethyl ester and activated by high affinity IgE receptor aggregation with Ag (100 ng/ml). Arrow indicates addition of Ag. Data represent the mean ± SD (n=4) from independent experiments performed in duplicates. **(E)** RT-qPCR analysis of cytokine (TNFα, IL-6 and IL-13) and prostaglandin (PTGS2) expression in control (pLKO.1-NT) and GIT2_KD1 cells. Cells were sensitized and either unstimulated or stimulated with Ag (100 ng/ml) for 30 min (TNFα, IL-6, PTGS2) or 60 min (IL-13). The obtained values were normalized with the internal actin control, and fold increases were determined relative to unstimulated cells, arbitrarily set at 1.0. Data represent the mean ± SD (n=4). **(A, C-E)** Two-tailed, unpaired Student's *t*-test was performed to determine statistical significance. **(B)** One-way ANOVA with Tukey's multiple comparison test was performed to determine statistical significance. *, $p < 0.05$; **, $p < 0.01$; ***, $p < 0.001$; ****, $p < 0.0001$.
