# Supplementary material for: Regulation of microtubule nucleation in mouse bone marrow-derived mast cells by ARF GTPase-activating protein GIT2
Source: Front Immunol. 2024 Feb 2;15:1321321. doi: 10.3389/fimmu.2024.1321321 (PMC10870779; doi:10.3389/fimmu.2024.1321321)
Supplement: Supplementary file 1 [file DataSheet_1.zip › Figure S6.pdf]

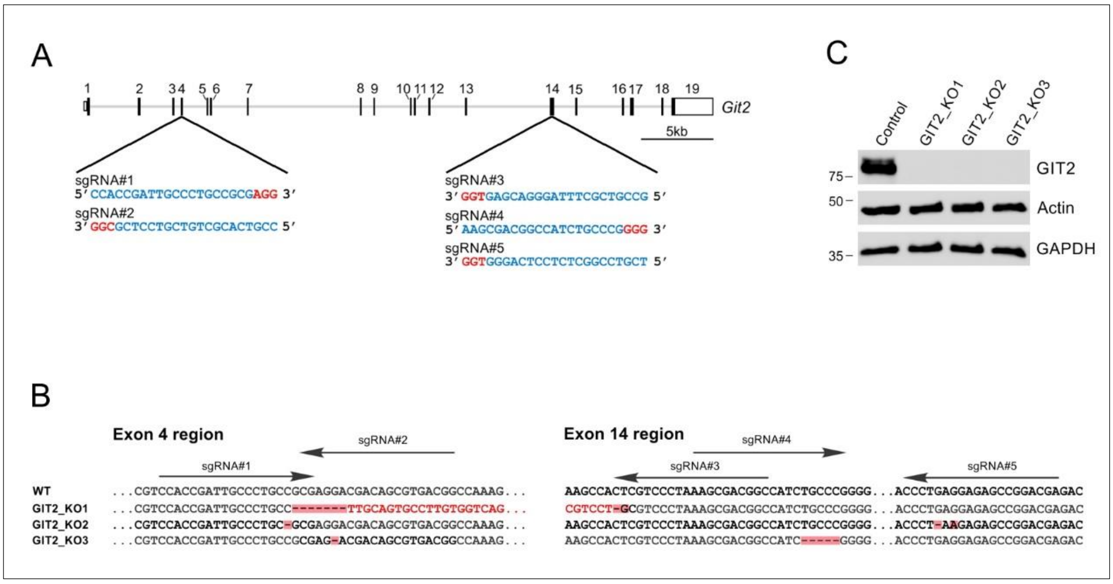


**Figure S6.** Generation of *Git2* knockout cell lines. **(A)** Schematic diagram of the longest transcript of *Git2* gene (47.2 kb) containing 19 exons and sites targeted by single guide RNA (sgRNA) sequences in exon 4 and exon 14. Targeted sites (blue) and protospacer adjacent motifs (PAM; red) are depicted. **(B)** Fragments of the genomic DNA from GIT2_KO1 (sgRNAs #1 and #3), GIT2_KO2 (sgRNAs #1and #5) and GIT2_KO3 (sgRNAs #2 and #4) cell lines were PCR amplified and sequenced. Wild-type *Git2* sequence (WT) and positions of sgRNA sequences are shown on the top. The deletions and insertions are marked in red. Sequencing of KO clones revealed inversion of genomic region between the sgRNA#1 and sgRNA#3 (GIT2_KO1) or small indels (GIT2_KO2, GIT2_KO3). **(C)** GIT2 protein levels in control and GIT2-deficient BMMCL. Immunoblot analysis of whole-cell lysates with Abs to GIT2, actin and GAPDH (loading control).
