# Supplementary material for: Regulation of microtubule nucleation in mouse bone marrow-derived mast cells by ARF GTPase-activating protein GIT2
Source: Front Immunol. 2024 Feb 2;15:1321321. doi: 10.3389/fimmu.2024.1321321 (PMC10870779; doi:10.3389/fimmu.2024.1321321)
Supplement: Supplementary file 1 [file DataSheet_1.zip › Figure S7.pdf]

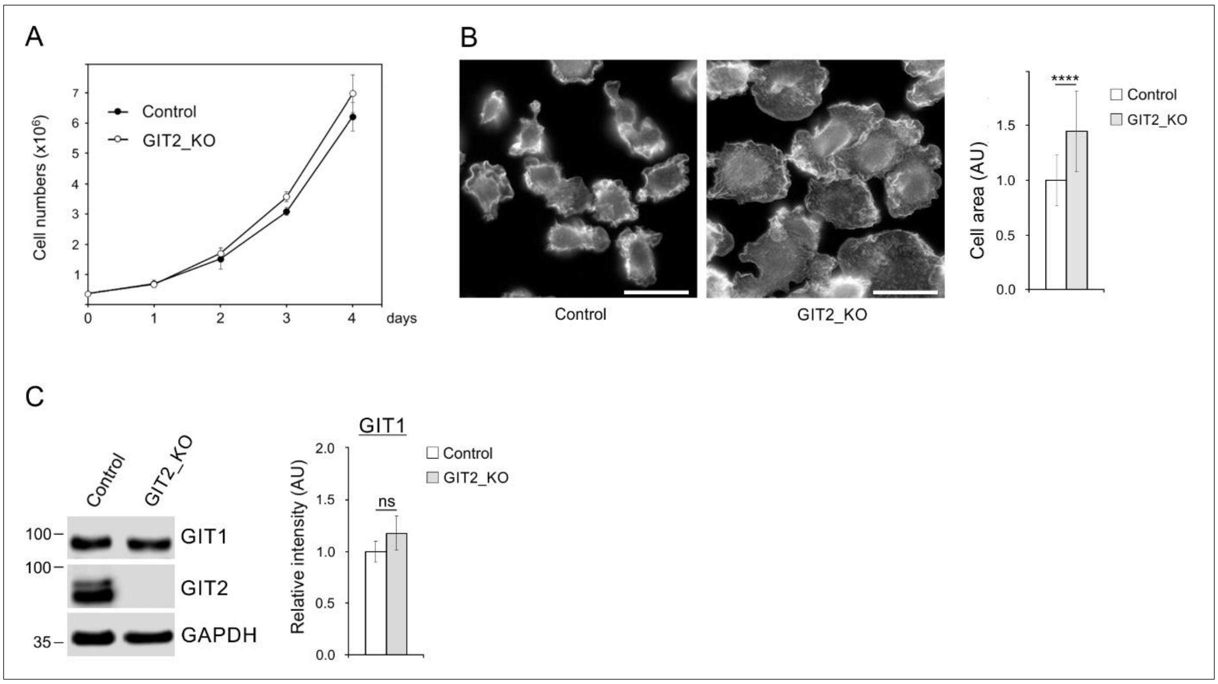

Figure S7. Characterization of cells lacking GIT2. **(A)** Growth curves in control and GIT2_KO1 cells. A total of 3 x 10^5 cells were plated in both cell lines. Values indicate mean ± SD (n = 6, control; n = 8, GIT2_KO). **(B)** Comparison of cell spreading in control and GIT2_KO cells. Cells were incubated on fibronectin-coated coverslips for 45 min, and thereafter fixed (F/Tx) and stained with rhodamine-phalloidin. Scale bar, 20 µm. Quantification of cell area is shown on the right. Three independent experiments (at least 89 cells counted in each experiment). Control (n = 596), GIT2_KO (n = 563). Values indicate mean ± SD. **(C)** GIT1 protein levels in control and GIT2_KO cells. Immunoblot analysis of whole-cell lysates with Abs to GIT1, GIT2 and GAPDH (loading control). Densitometric quantification of immunoblots is shown on the right. Relative intensity of GIT1 normalized to control cells and to the amount of GAPDH. Values indicate mean ± SD (n = 5). **(B-C)** Two-tailed, unpaired Student's *t*-test was performed to determine statistical significance. ns, $p > 0.05$; ****, $p < 0.0001$.
