# Supplementary material for: Regulation of microtubule nucleation in mouse bone marrow-derived mast cells by ARF GTPase-activating protein GIT2
Source: Front Immunol. 2024 Feb 2;15:1321321. doi: 10.3389/fimmu.2024.1321321 (PMC10870779; doi:10.3389/fimmu.2024.1321321)
Supplement: Supplementary file 1 [file DataSheet_1.zip › Figure S8.pdf]

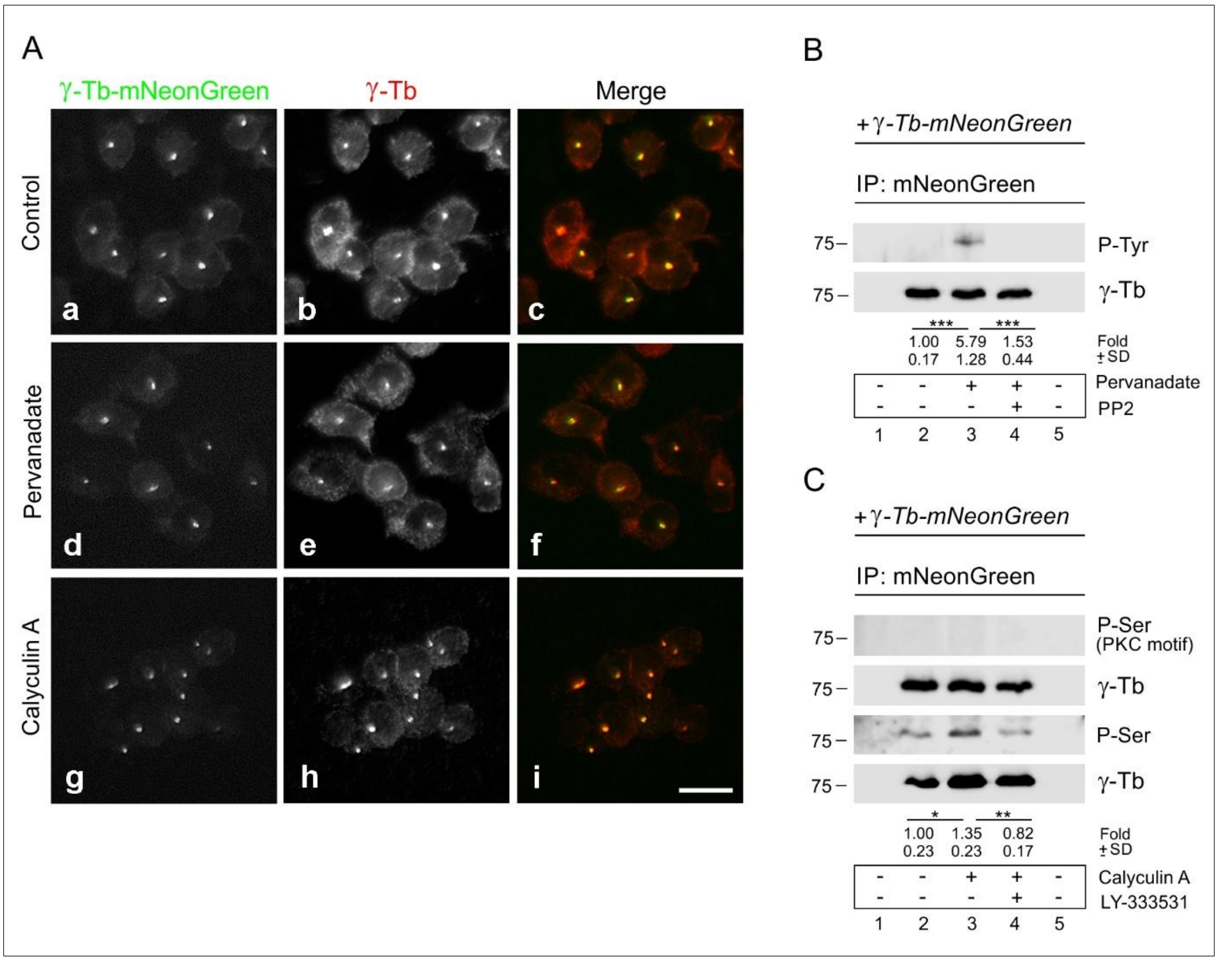

Figure S8. The effect of pervanadate and calyculin A on subcellular localization of γ-tubulin-mNeonGreen. **(A)** GIT2_KO cells expressing mNeonGreen-tagged γ-tubulin were untreated (Control, a-c) or pretreated with pervanadate (d-f) or Calyculin A (g-i), and then fixed and stained with Ab to γ-tubulin (γ-Tb). γ-Tubulin-1-mNeonGreen (a, d, g), γ-tubulin (b, e, h), superposition of images (c, f, i; GIT2-mNeonGreen, green; γ-tubulin, red). Fixation Tx/F/M. Scale bar, 10 μm (a-i). **(B-C)** Immunoprecipitation experiments with the whole-cell extracts from GIT2_KO cells expressing γ-Tubulin-1-mNeonGreen and Ab to mNeonGreen. Immobilized Abs without cell extracts (*lane 1*), precipitated proteins (*lanes 2-4*), and Ab-free carriers incubated with cell extract (*lane 5*). **(B)** Cells pretreated with DMSO alone (*lane 3*) or with Src kinase family selective inhibitor PP2 (20 μM, 60 min; *lane 4*) before incubation with pervanadate (15min). The blots were probed with Abs to P-Tyr and γ-tubulin. Numbers under the blot indicate relative amounts of γ-Tubulin-1-mNeonGreen phosphorylated on tyrosine (P-Tyr) normalized to pervanadate untreated cells and the amount of precipitated γ-Tubulin-1-mNeonGreen in individual samples (fold). Mean ± SD (n = 4). (**C**) Cells pretreated with DMSO alone (*lane 3*) or with PKCβ selective inhibitor LY-333531 (10 μM, 30 min; *lane 4*) before incubation with Calyculin A (100 nM, 30 min). The blots were first probed with Abs to P-Ser in PKC motif or P-Ser and then re-probed with Ab to γ-tubulin. Numbers under the blot indicate relative amounts of γ-Tubulin-1-mNeonGreen phosphorylated on serine (P-Ser) normalized to Calyculin A untreated cells and the amount of precipitated γ-Tubulin-1-mNeonGreen in individual samples (fold). Mean ± SD (n = 5). Two-tailed, unpaired Student's *t*-test was performed to determine statistical significance. *, p < 0.05; **, p < 0.01; ***, p < 0.001.
