# Supplementary material for: Regulation of microtubule nucleation in mouse bone marrow-derived mast cells by ARF GTPase-activating protein GIT2
Source: Front Immunol. 2024 Feb 2;15:1321321. doi: 10.3389/fimmu.2024.1321321 (PMC10870779; doi:10.3389/fimmu.2024.1321321)
Supplement: Supplementary file 1 [file DataSheet_1.zip › Figure S9.pdf]

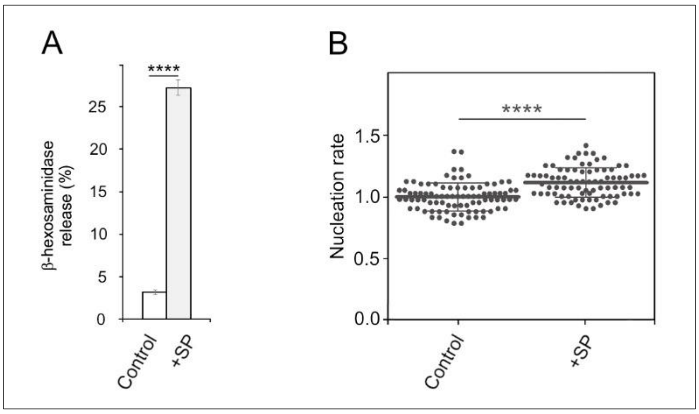

Figure S9. The effect of SP-induced activation of BMMCL on degranulation and microtubule nucleation. (**A**) Comparison of degranulation in control cells (n = 3) and SP-activated cells (+SP; n = 3). Cells were activated with 20 μM SP, and degranulation was measured by release of β-hexosaminidase. Data represent mean ± SD. (**B**) Microtubule nucleation rate (EB3 comets/min) in activated cells (+SP) relative to non-activated cells (Control). Cells were activated with 20 μM SP for 10 min. Three independent experiments (at least 21 cells counted in each experiment). Control (n = 88), +SP (n = 82). The bold and thin lines within the dot plot represent mean ± SD. Two-tailed, unpaired Student's *t*-test was performed to determine statistical significance. ****, $p < 0.0001$.
