# Supplementary material for: Regulation of microtubule nucleation in mouse bone marrow-derived mast cells by ARF GTPase-activating protein GIT2
Source: Front Immunol. 2024 Feb 2;15:1321321. doi: 10.3389/fimmu.2024.1321321 (PMC10870779; doi:10.3389/fimmu.2024.1321321)
Supplement: Supplementary file 1 [file DataSheet_1.zip › Tables S1-S4.pdf]

Supplementary Material

TABLE S1 Sequences of primers used for preparation of GST-tagged GCP3 constructs

Fragment length	Sequence	Amplicon length
1-25aa, fwd	5’-ATATGAATTCATGGCGACCCCGGAC -3’	97 bp
1-25aa, rev	5’-ATTGTCGACTTATTCGCTCCTGCCCAGGA -3’	
1-50aa, fwd	5’-ATATGAATTCATGGCGACCCCGGAC -3’	172 bp
1-50aa, rev	5’-GCCGTCGACTTAATCTCTTTCAACAGTTGGG -3’	
1-75aa, fwd	5’-ATATGAATTCATGGCGACCCCGGAC -3’	247 bp
1-75aa, rev	5’-GCCGTCGACTTATGAAAATAATGCAGCATCTG -3’	
1-100aa, fwd	5’-ATATGAATTCATGGCGACCCCGGAC -3’	322 bp
1-100aa, rev	5’-ATCGTCGACTTAGAGGCTCAGCAAGAGGTAG -3’	
1-200aa, fwd	5’-ATATGAATTCATGGCGACCCCGGAC -3’	622 bp
1-200aa, rev	5’-ATTGTCGACTTACCCCAACTGCTGTCGA -3’	
1-310aa, fwd	5’-ATATGAATTCATGGCGACCCCGGAC -3’	952 bp
1-310aa, rev	5’-ATTGTCGACTTACTGGTCCGTGTATCTTCT -3’	
101-907aa, fwd	5’-ATTAGGATCCAGTGAGGACCCACGCA -3’	2443 bp
101-907aa, rev	5’- ATTGTCGACTCACGTGTGGGAGCTGC -3’	

Underlined are restriction sites for *Eco*RI and *Sal*I.

TABLE S2 Sequences of oligonucleotides for the preparation of shRNA constructs for the depletion of mouse GIT2

shRNA-A

Oligonucleotide	Sequence
Sense	5’-CCGGGGCCGGAAACCAGATCATAAACTCGAGTTTATGATCTGGTTTCCGGCCTTTTTG-3’
Antisense	5’-AATTCAAAAAGGCCGGAAACCAGATCATAAACTCGAGTTTATGATCTGGTTTCCGGCC-3’

shRNA-B

Oligonucleotide	Sequence
Sense	5’-CCGGTCAATCTCTGAGTAATCATTTCTCGAGAAATGATTACTCAGAGATTGATTTTTG-3’
Antisense	5’-AATTCAAAAATCAATCTCTGAGTAATCATTTCTCGAGAAATGATTACTCAGAGATTGA-3’

Targeted sequences are underlined.

TABLE S3 Sequences of primers used to determine gene alterations in GIT2_KO clones

Clone	Sequence	Amplicon length
**GIT2_KO1**		
sgRNA#1 region:		
Fwd:	5‘-GTCACTTCCTCTTCTTACAGCACA-3‘	296 bp
Inv_rev:	5‘-GCCTGGTGTGCAAAAACACA-3‘	
sgRNA#3 region:		
Inv_fwd:	5‘-GCGCCCACCTTACTGAGATC-3‘	470 bp
Rev:	5‘-CAGCCTCCGCCTATGATGAA-3‘	
**GIT2_KO2**		
sgRNA#1 region:		
Fwd:	5‘-CTTGACTCCGGGTGATGAGG-3‘	971 bp
Rev:	5‘-GCACACGCTGTCTTGAGTGA-3‘	
sgRNA#5 region:		
Fwd:	5‘-AGCCCCGCTAGCTTTATGTC-3‘	475 bp
Rev:	5‘-AGCCAGAACGCCCAACTTTA-3‘	
**GIT2_KO3**		
sgRNA#2 region:		
Fwd:	5‘-CTTGACTCCGGGTGATGAGG-3‘	971 bp
Rev:	5‘-GCACACGCTGTCTTGAGTGA-3‘	
sgRNA#4 region:		
Fwd:	5‘-AGCCCCGCTAGCTTTATGTC-3‘	475 bp
Rev:	5‘-AGCCAGAACGCCCAACTTTA-3‘	

TABLE S4 Sequences of primers used for qRT-PCR analysis of mouse genes

Name	Sequence	Amplicon length
Il6, fwd	5‘-GAGGATACCACTCCCAACAGACC-3‘	140 bp
Il6, rev	5‘-AAGTGCATCATCGTTGTTCATACA-3‘	
Il13, fwd	5’-CTTAAGGAGCTTATTGAGGAG-3’	144 bp
Il13, rev	5’-CATTGCAATTGGAGATGTTG-3’	
Tnf, fwd	5’-CTATGTCTCAGCCTCTTCTC-3’	108 bp
Tnf, rev	5’-CATTTGGGAACTTCTCATCC-3’	
Ptgs2, fwd	5’-ACTCATAGGAGAGACTATCAAG-3’	147 bp
Ptgs2, rev	5’-GAGTGTGTTGAATTCAGAGG-3’	
Actb, fwd	5‘-GGACCTGACAGACTACCTCATG-3‘	93 bp
Actb, rev	5‘-TCTTTGATGTCACGCACGATTT-3‘	
