# Supplementary material for: Regulation of microtubule nucleation in mouse bone marrow-derived mast cells by ARF GTPase-activating protein GIT2
Source: Front Immunol. 2024 Feb 2;15:1321321. doi: 10.3389/fimmu.2024.1321321 (PMC10870779; doi:10.3389/fimmu.2024.1321321)
Supplement: Supplementary file 1 [file DataSheet_1.zip › Video S1 caption.pdf]

Supplementary Material

Video S1 Time-lapse sequences of non-activated and activated cell. Cell was activated by FcεRI aggregation by Ag at 100 ng/ml concentration for 5 min. Time-lapse sequences of the same cell before and after activation were collected in five optical slices (0.2 μm steps) for 30 sec at 1 s intervals with the Andor Dragonfly 503 spinning disc confocal microscope. The time-lapse sequences were deconvoluted with Huygens Professional software v. 19.04, and maximum intensity projection of z stack was made for each time point in Fiji.
