# Supplementary material for: Regulation of microtubule nucleation in mouse bone marrow-derived mast cells by ARF GTPase-activating protein GIT2
Source: Front Immunol. 2024 Feb 2;15:1321321. doi: 10.3389/fimmu.2024.1321321 (PMC10870779; doi:10.3389/fimmu.2024.1321321)
Supplement: Supplementary file 2 [file Figure 2-originals.pdf › Data Sheet 7/Figure 2-originals.pdf]

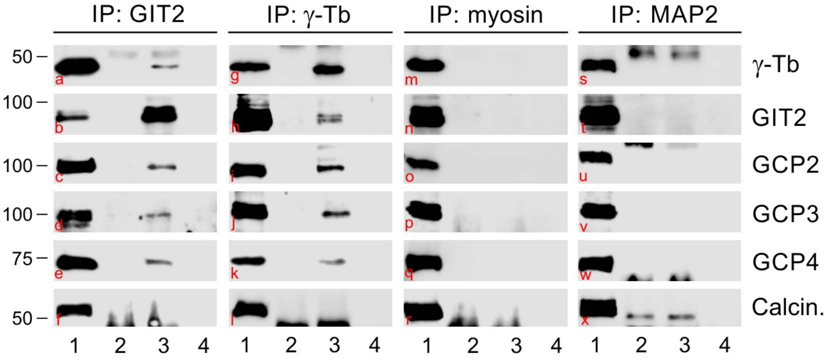

IP: GIT2
IP: γ-Tb
IP: myosin
IP: MAP2
50 –
100 –
100 –
100 –
75 –
50 –
γ-Tb
GIT2
GCP2
GCP3
GCP4
Calcin.
a
b
c
d
e
f
g
h
i
j
k
l
m
n
o
p
q
r
s
t
u
v
w
x
1 2 3 4
1 2 3 4
1 2 3 4
1 2 3 4

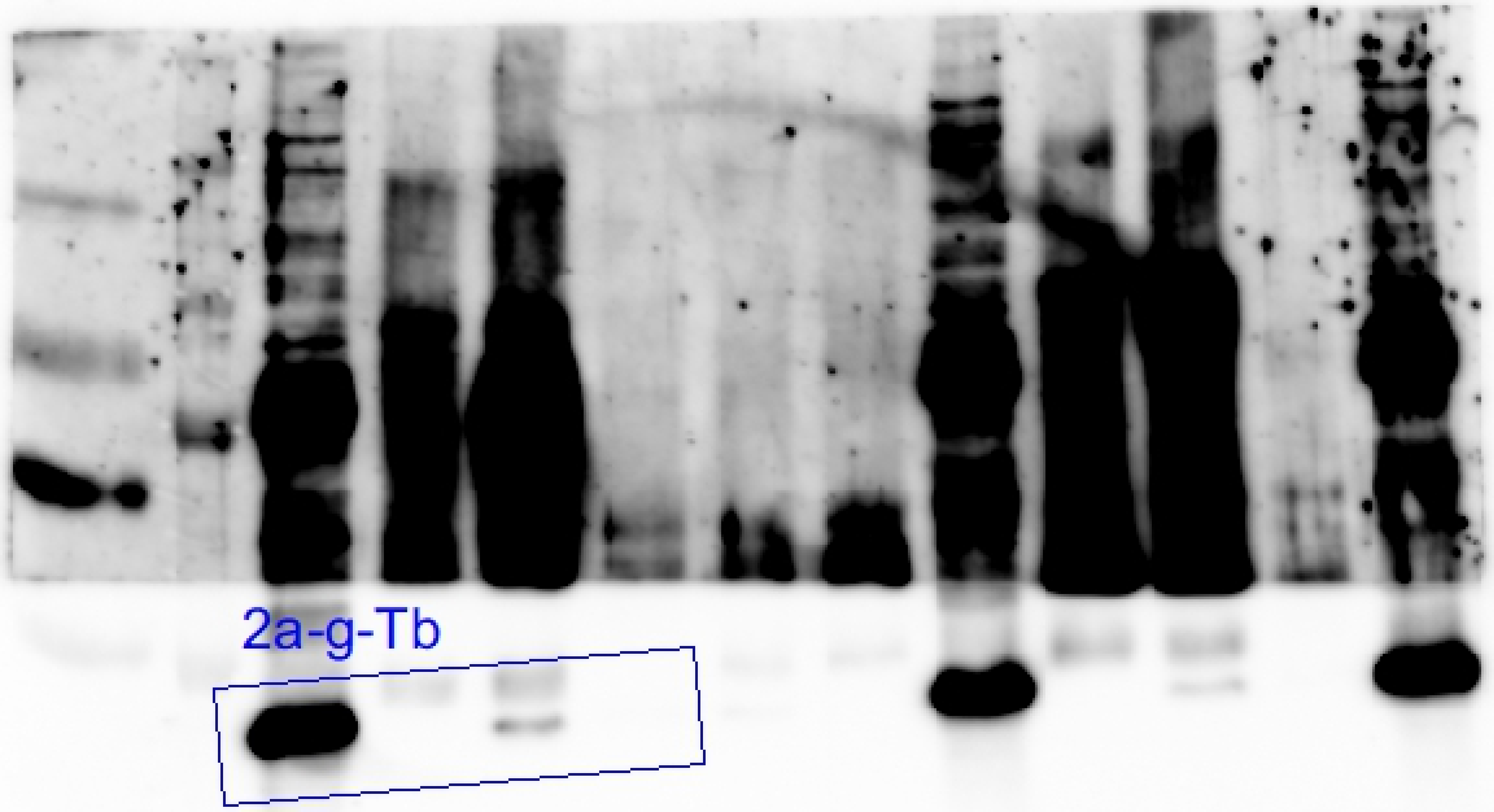
2a-g-Tb

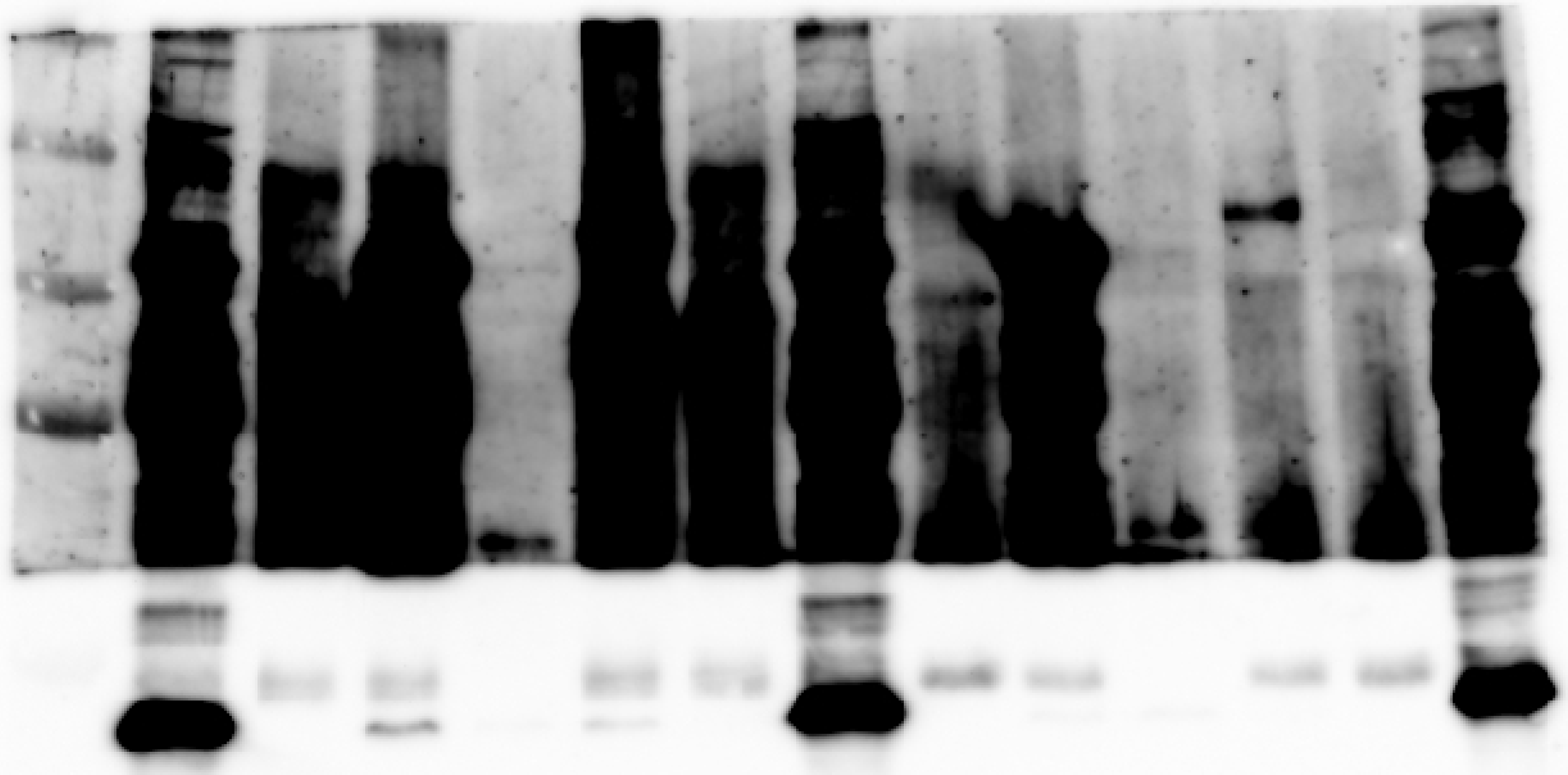

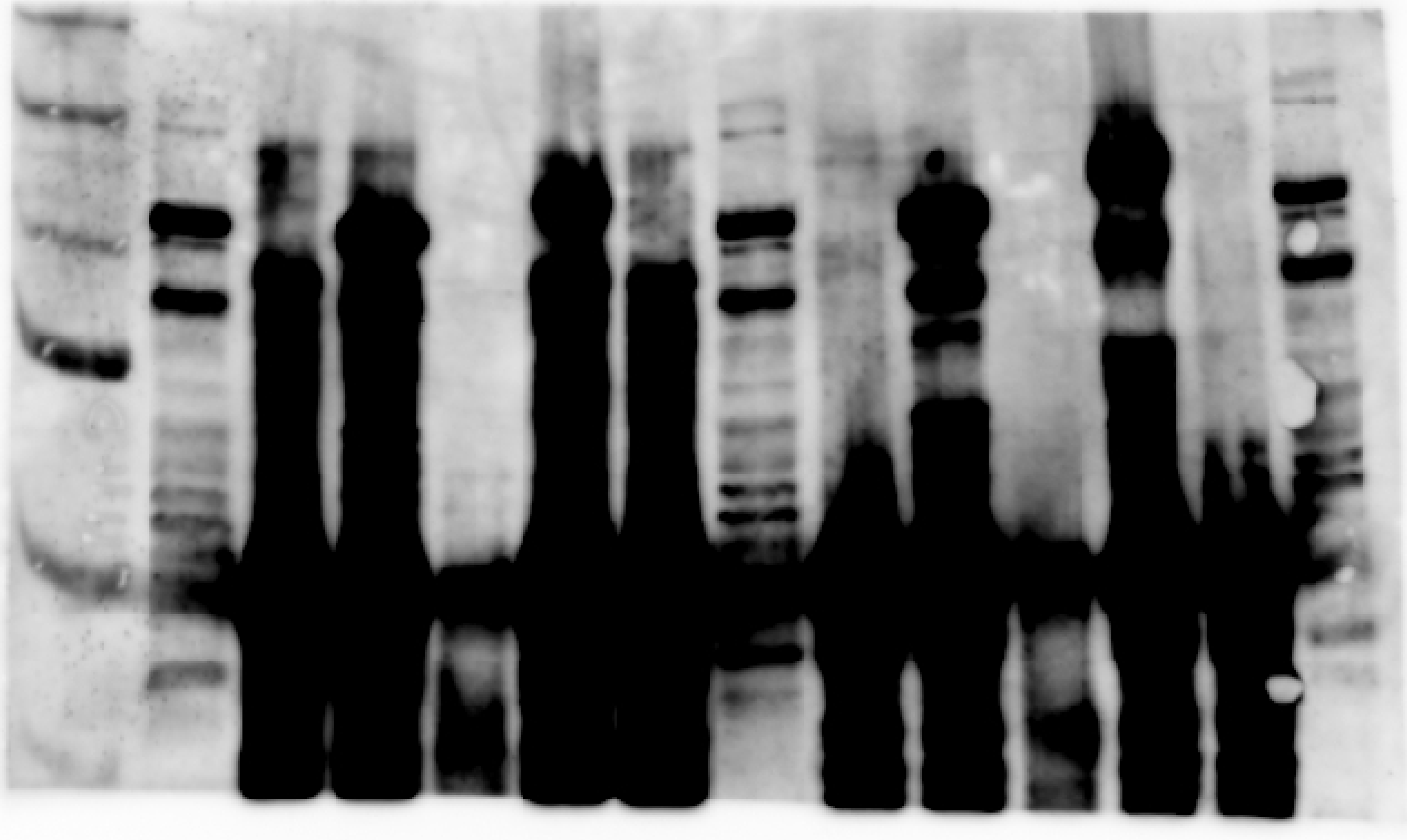

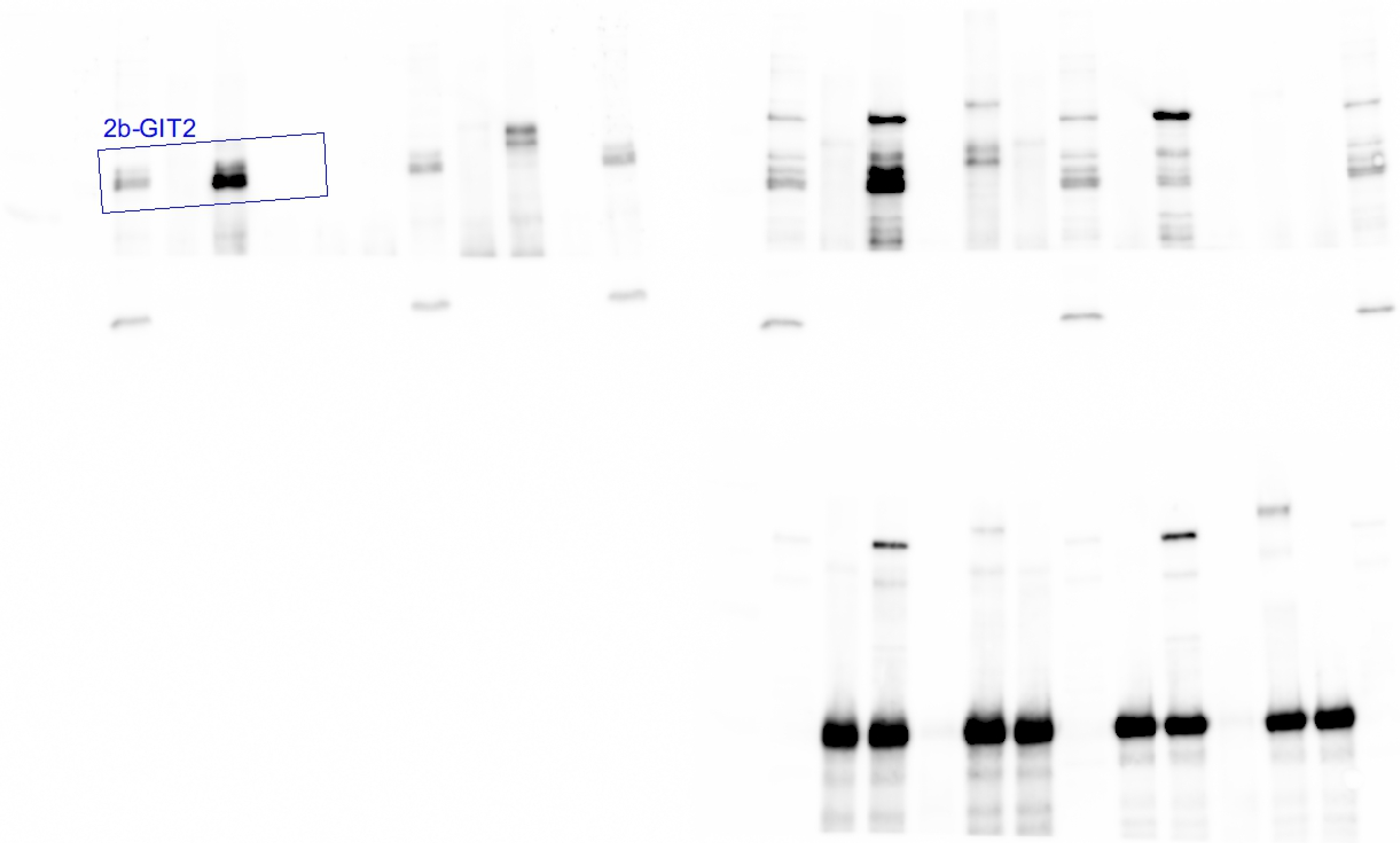
2b-GIT2

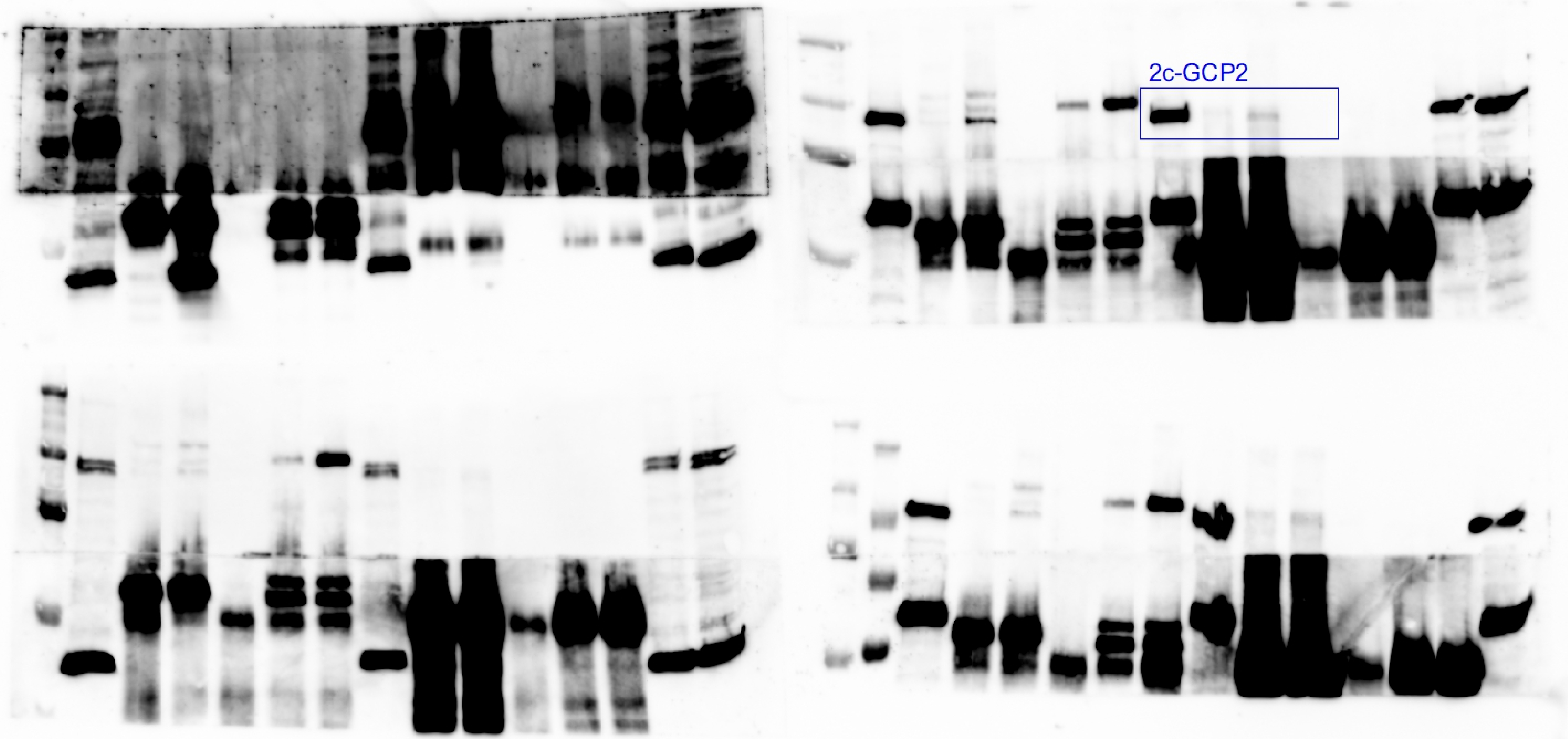
2c-GCP2

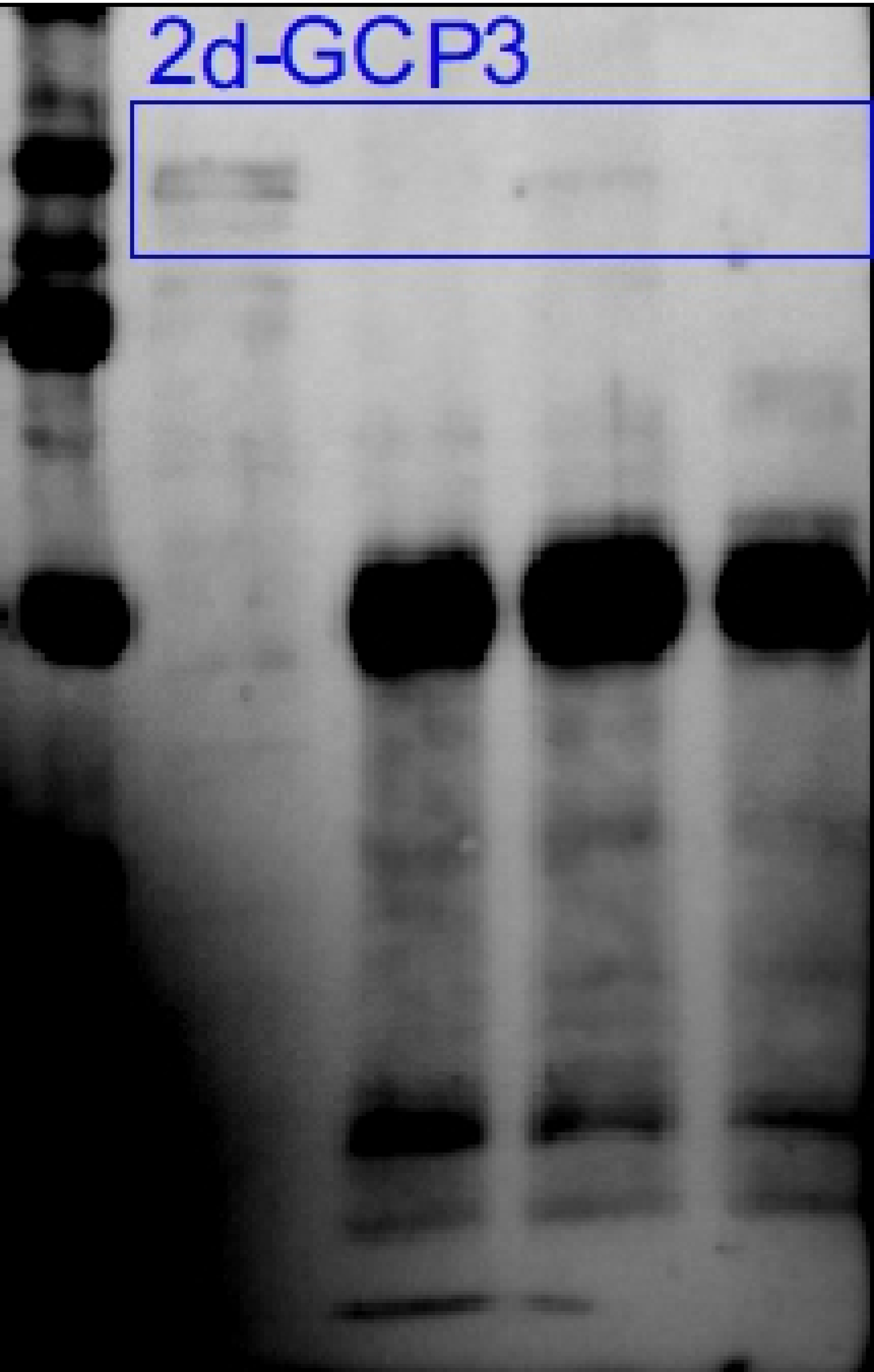
2d-GCP3

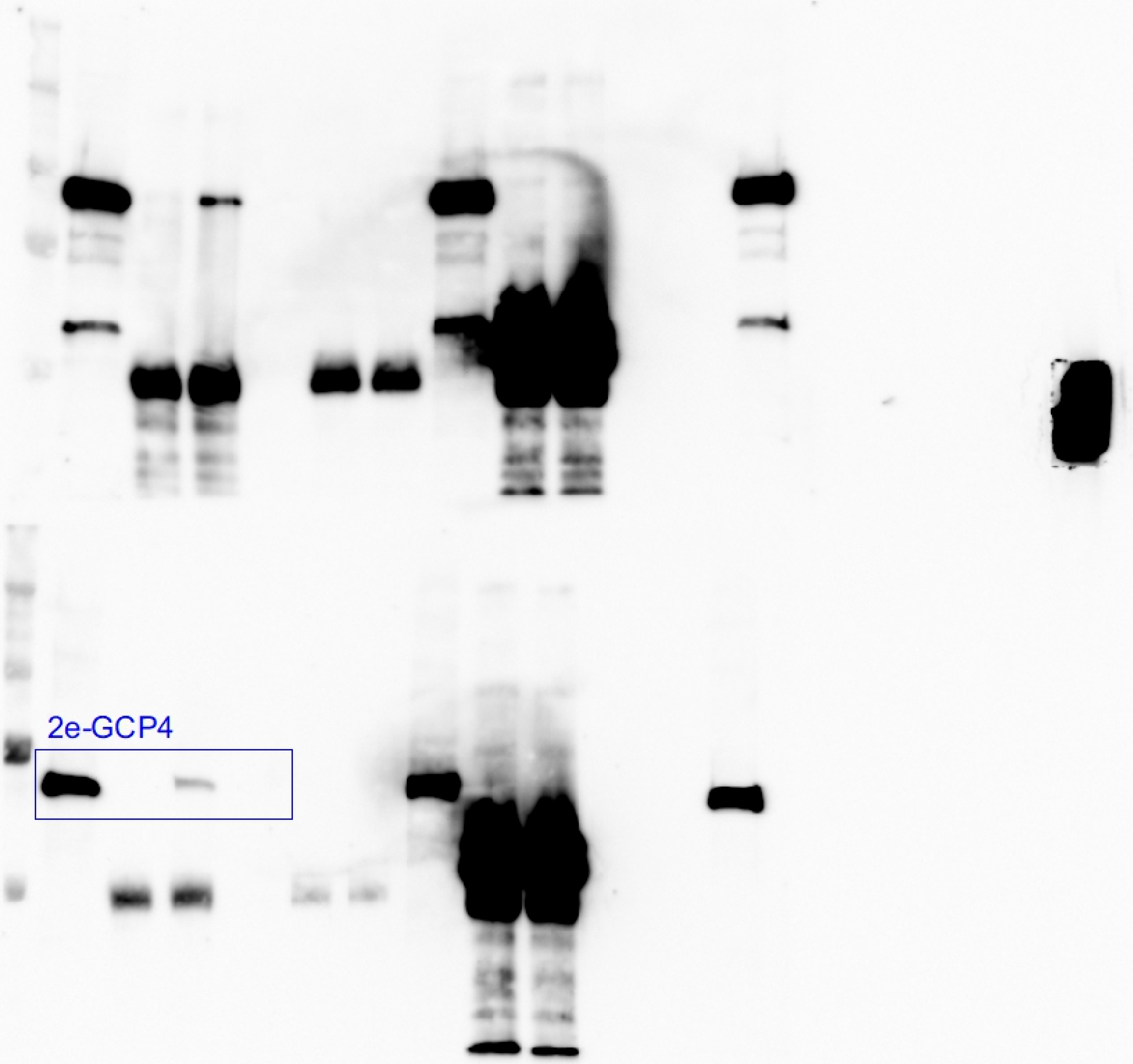
2e-GCP4

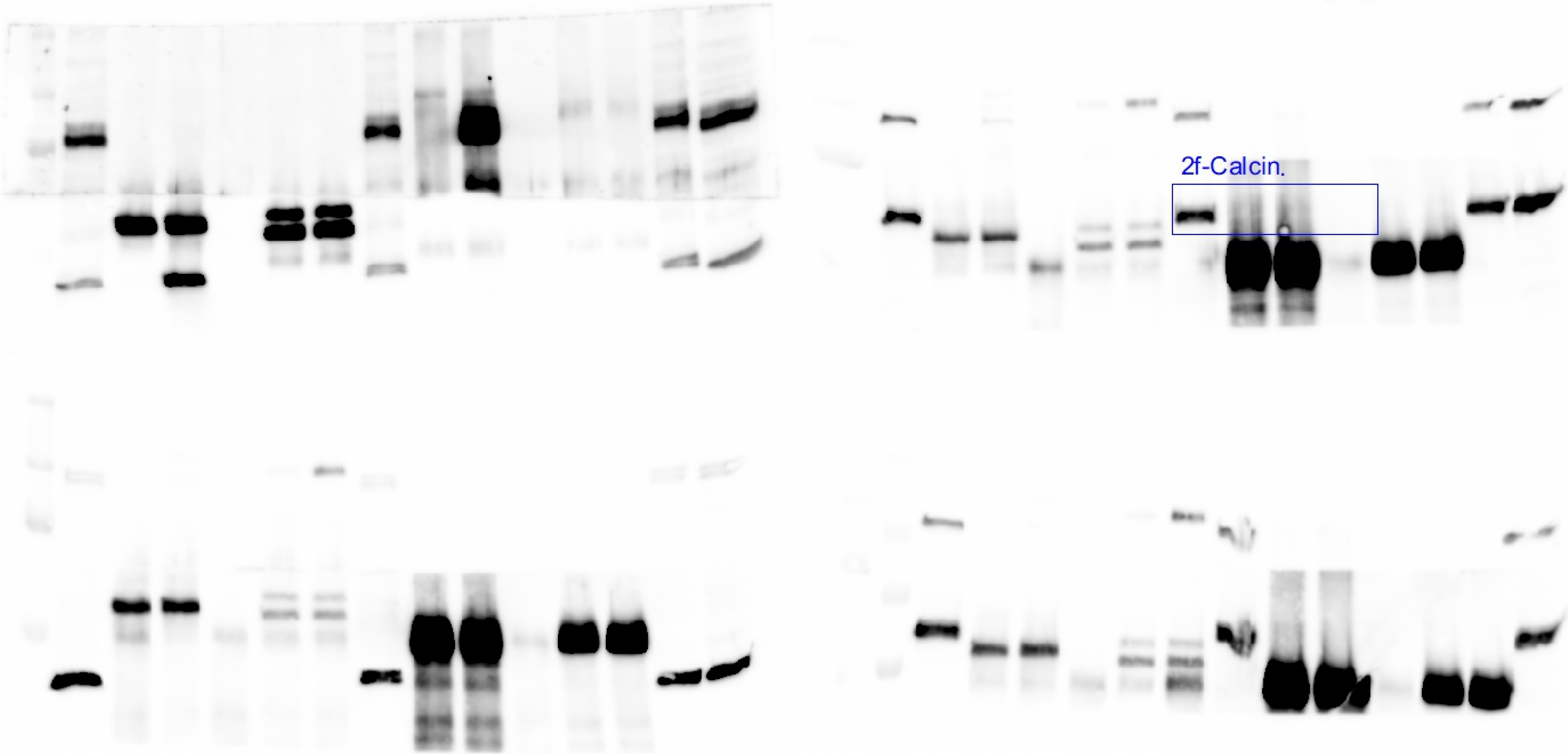
2f-Calcin.

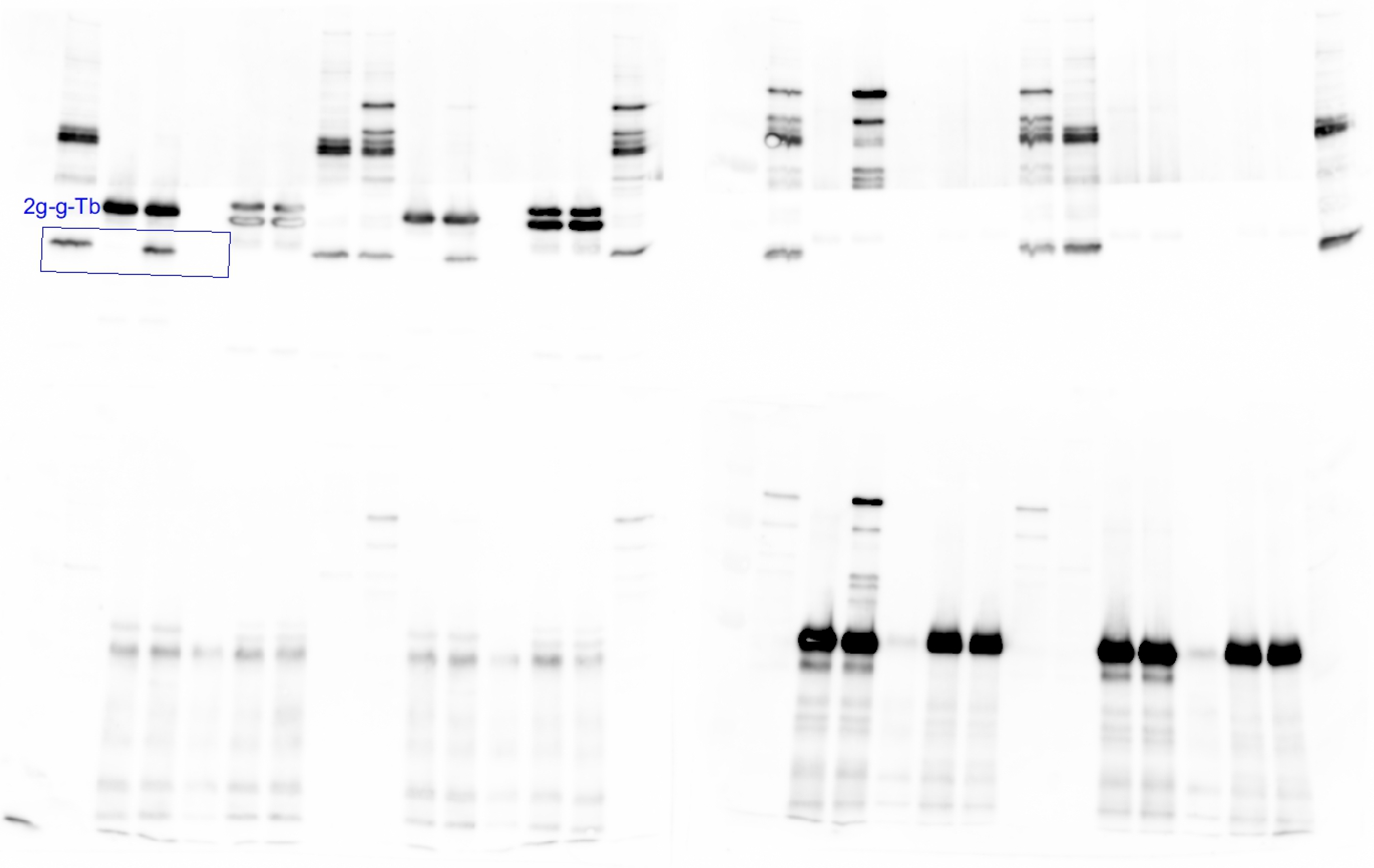
2g-g-Tb

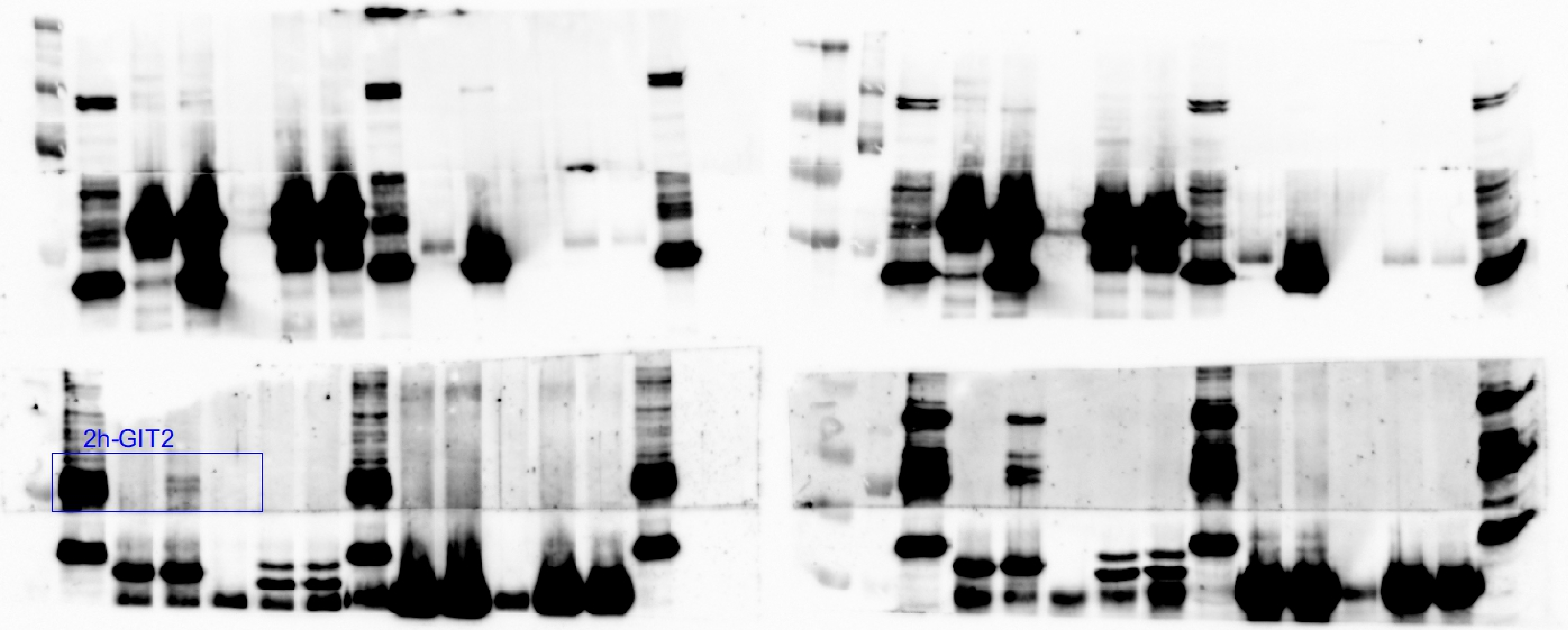
2h-GIT2

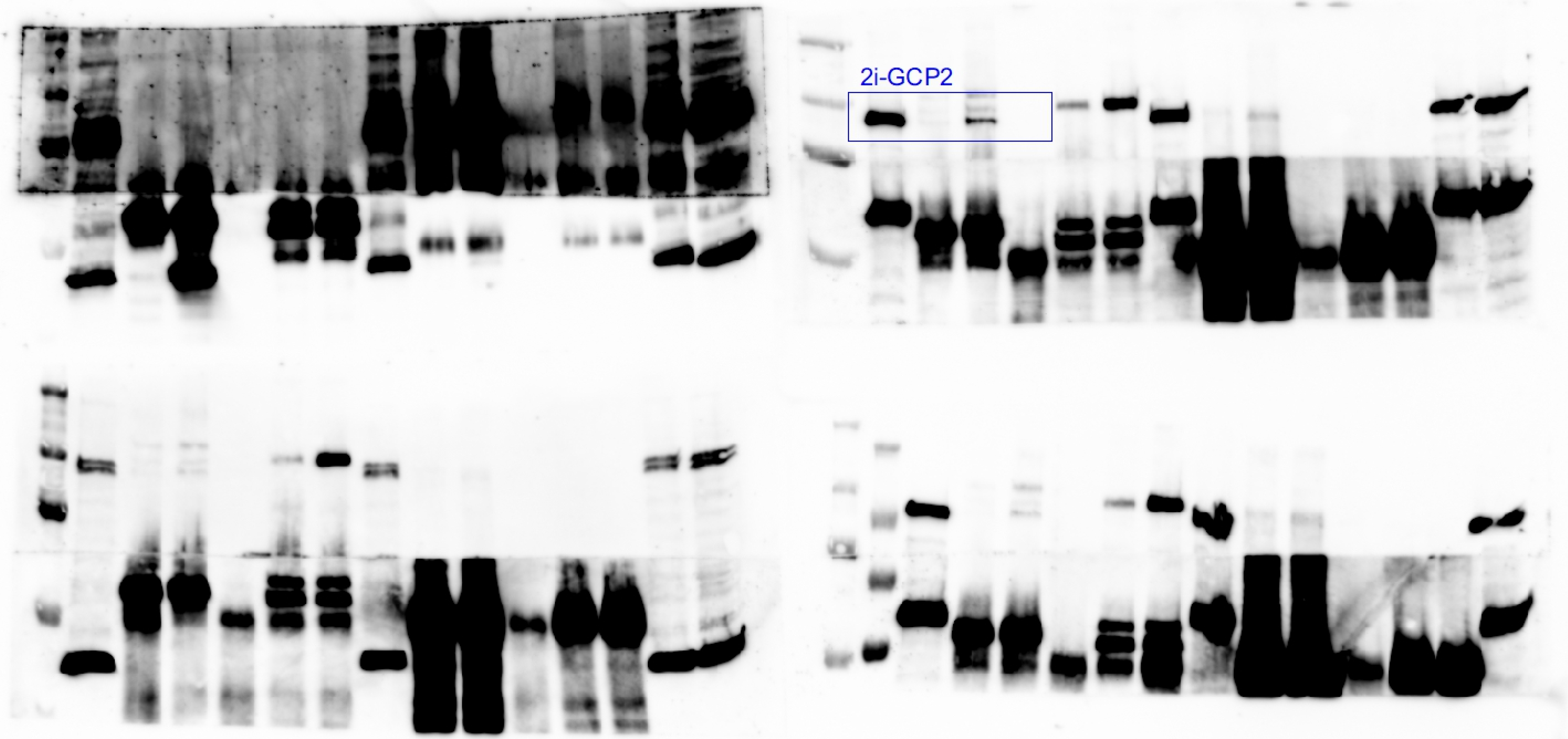
2i-GCP2

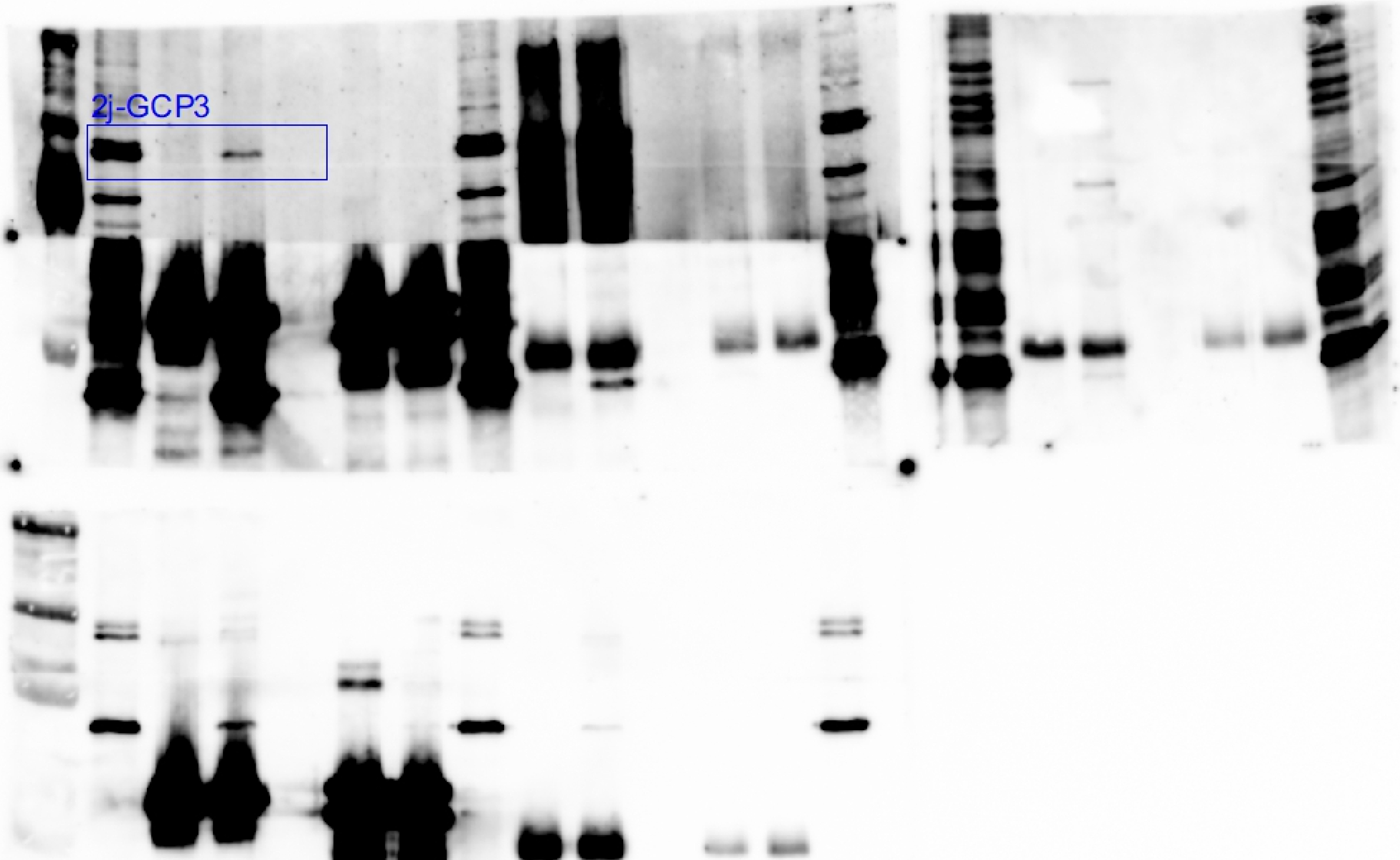
2j-GCP3

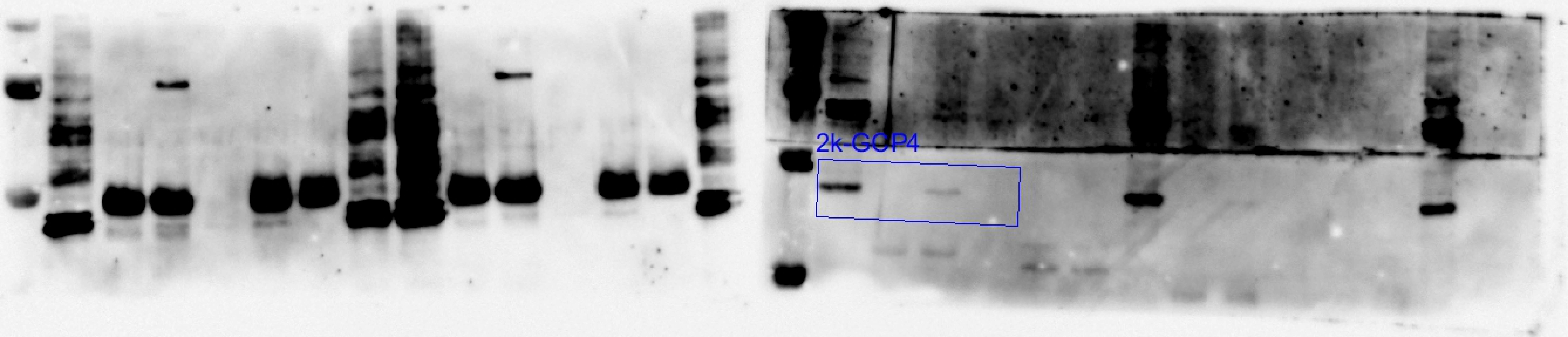
2k-GCP4

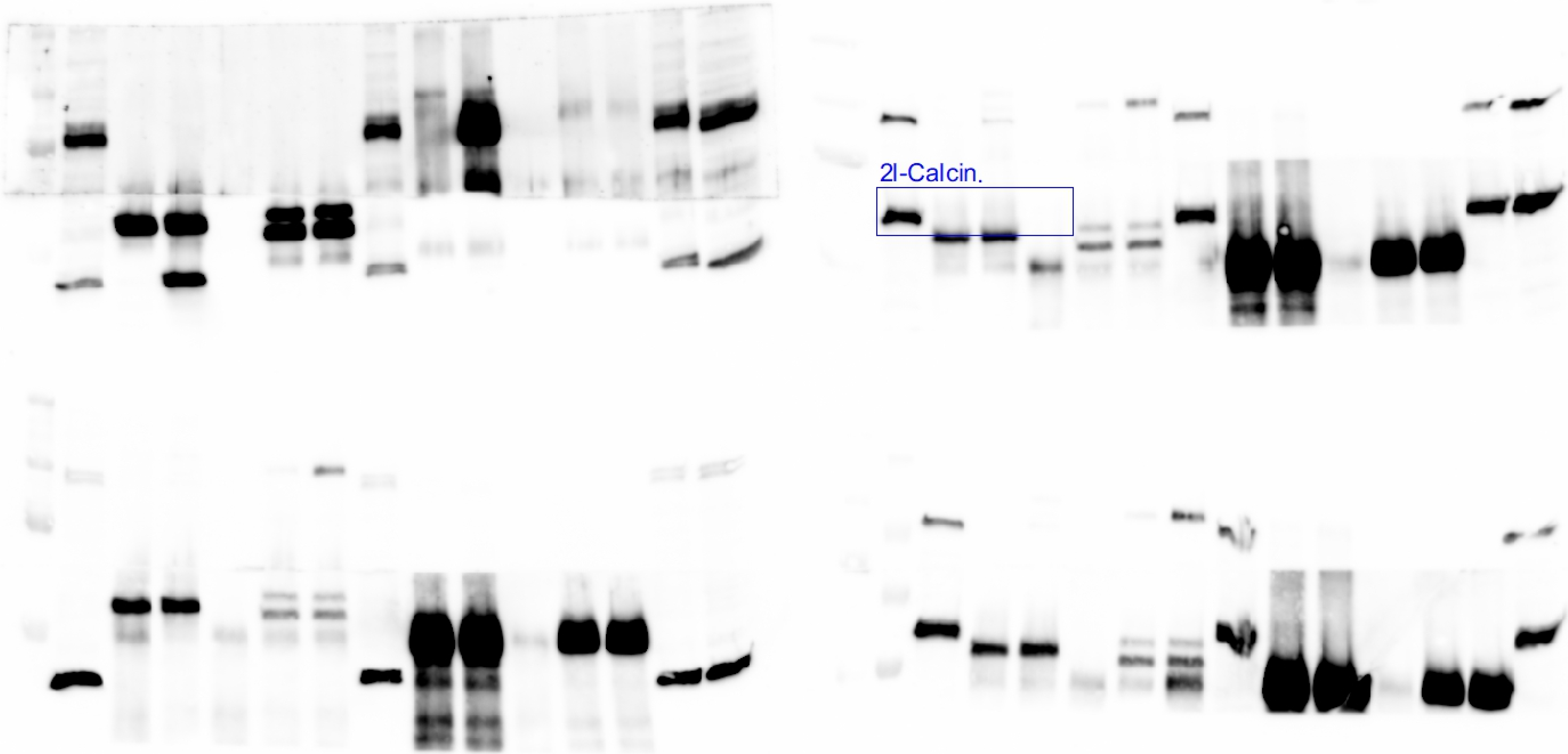
2I-Calcin.

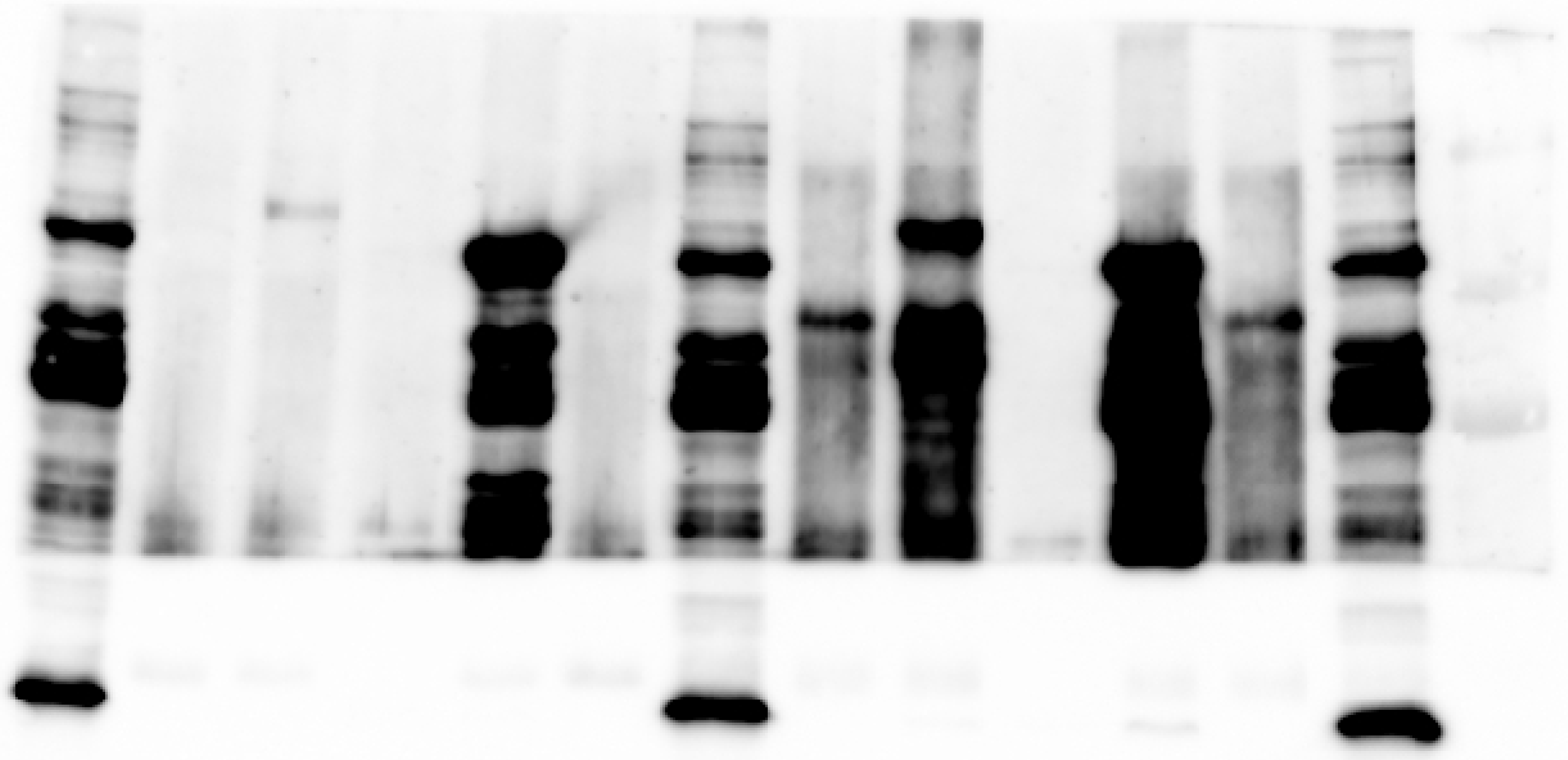

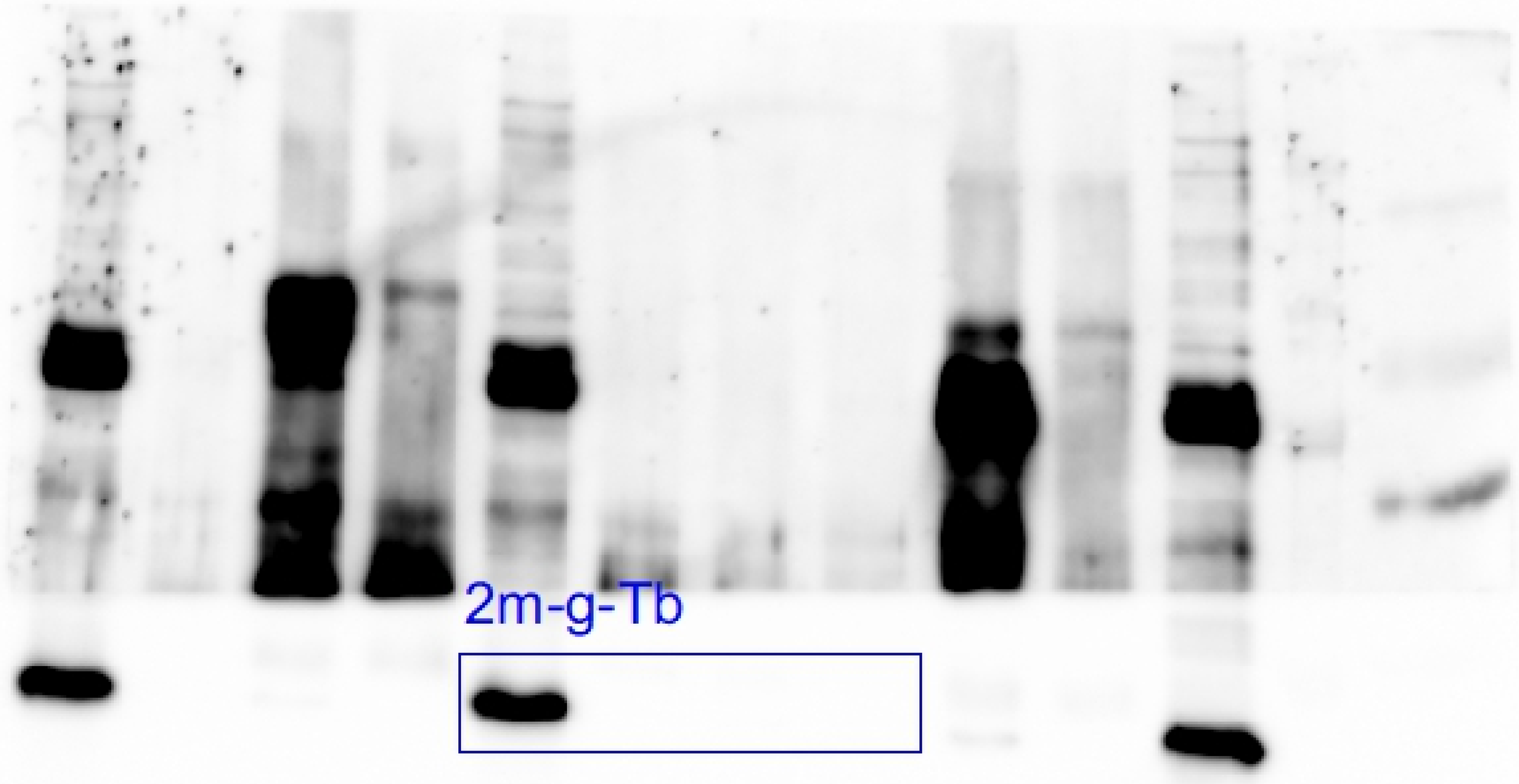
2m-g-Tb

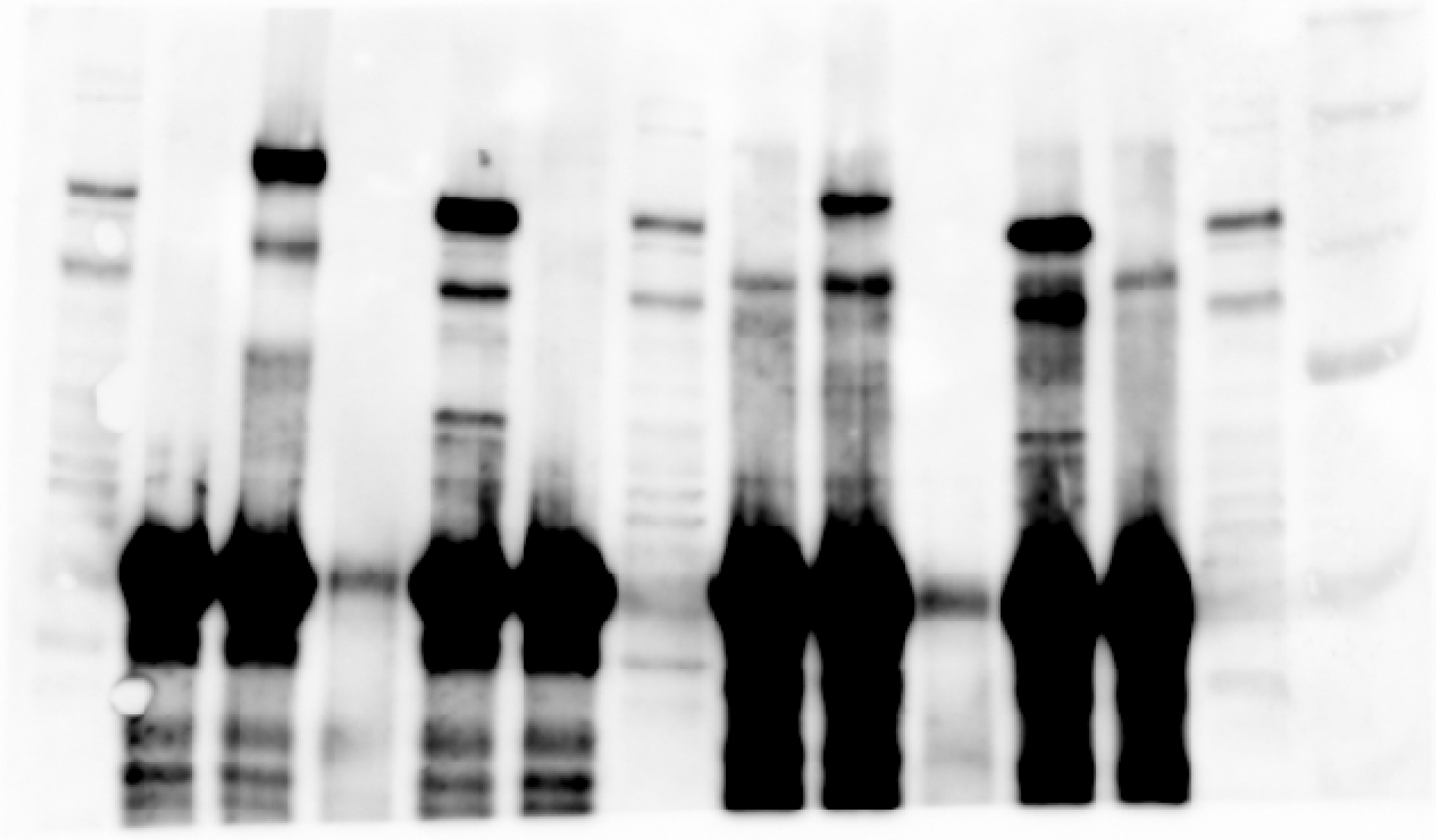

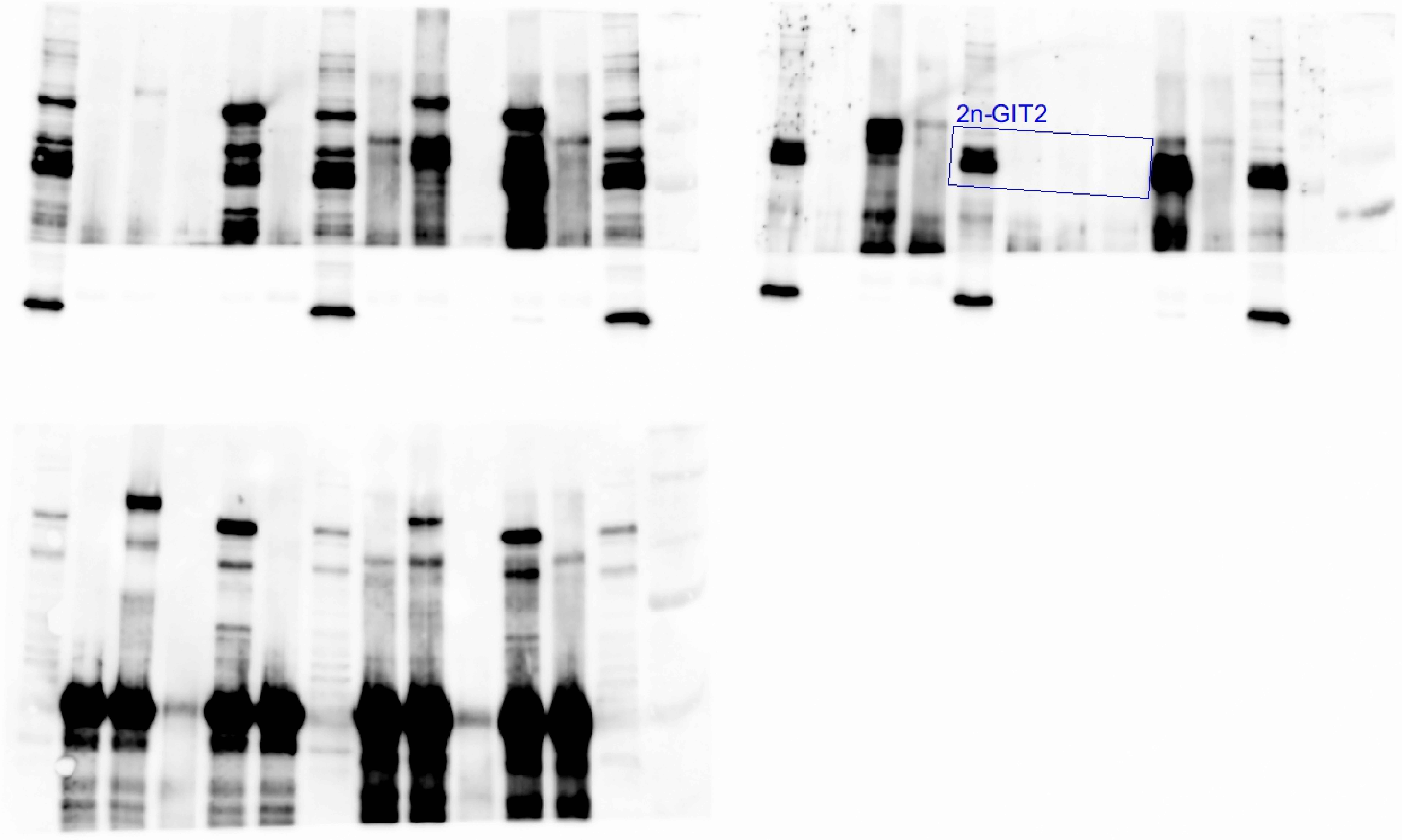
2n-GIT2

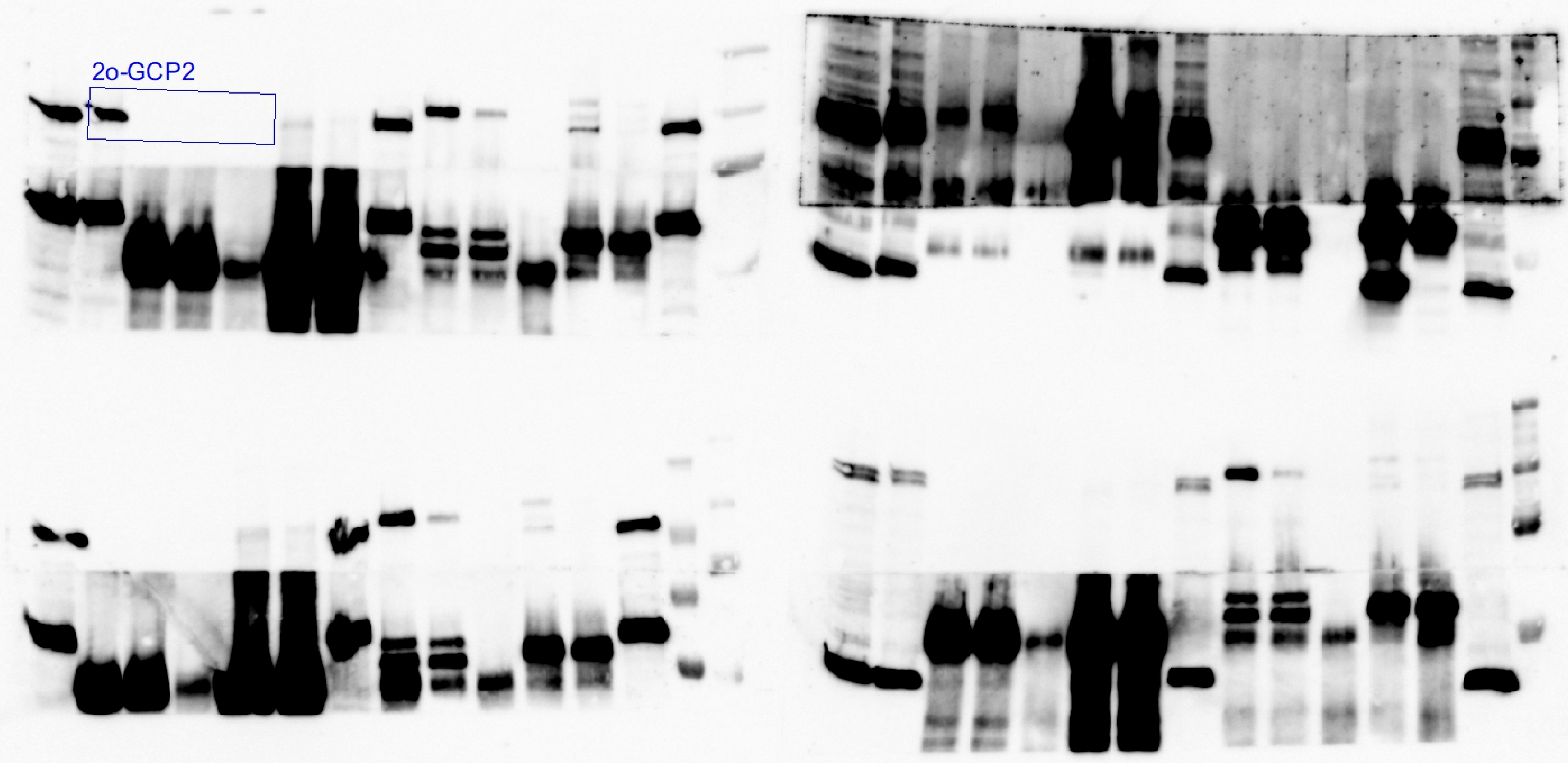
2o-GCP2

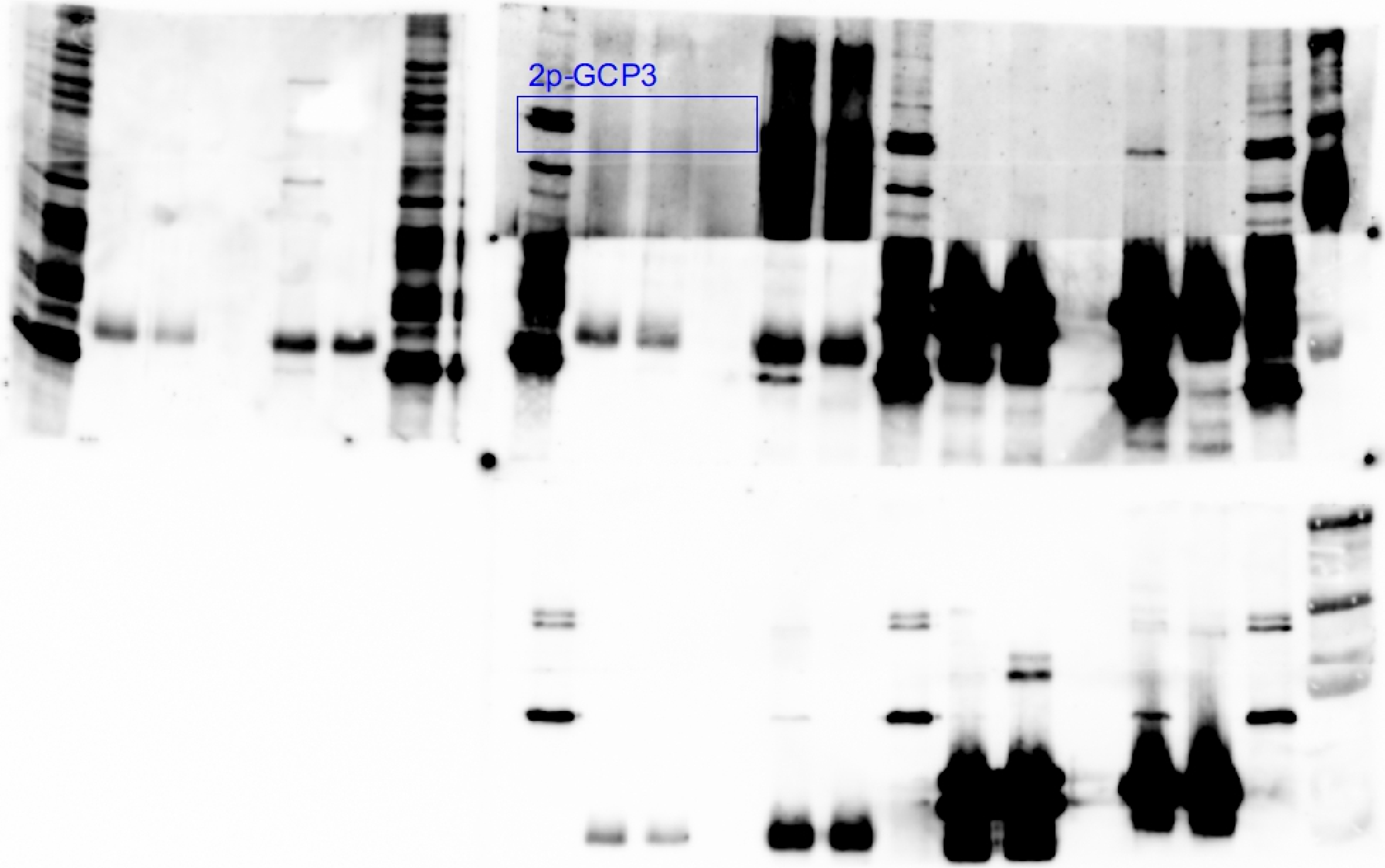

2p-GCP3

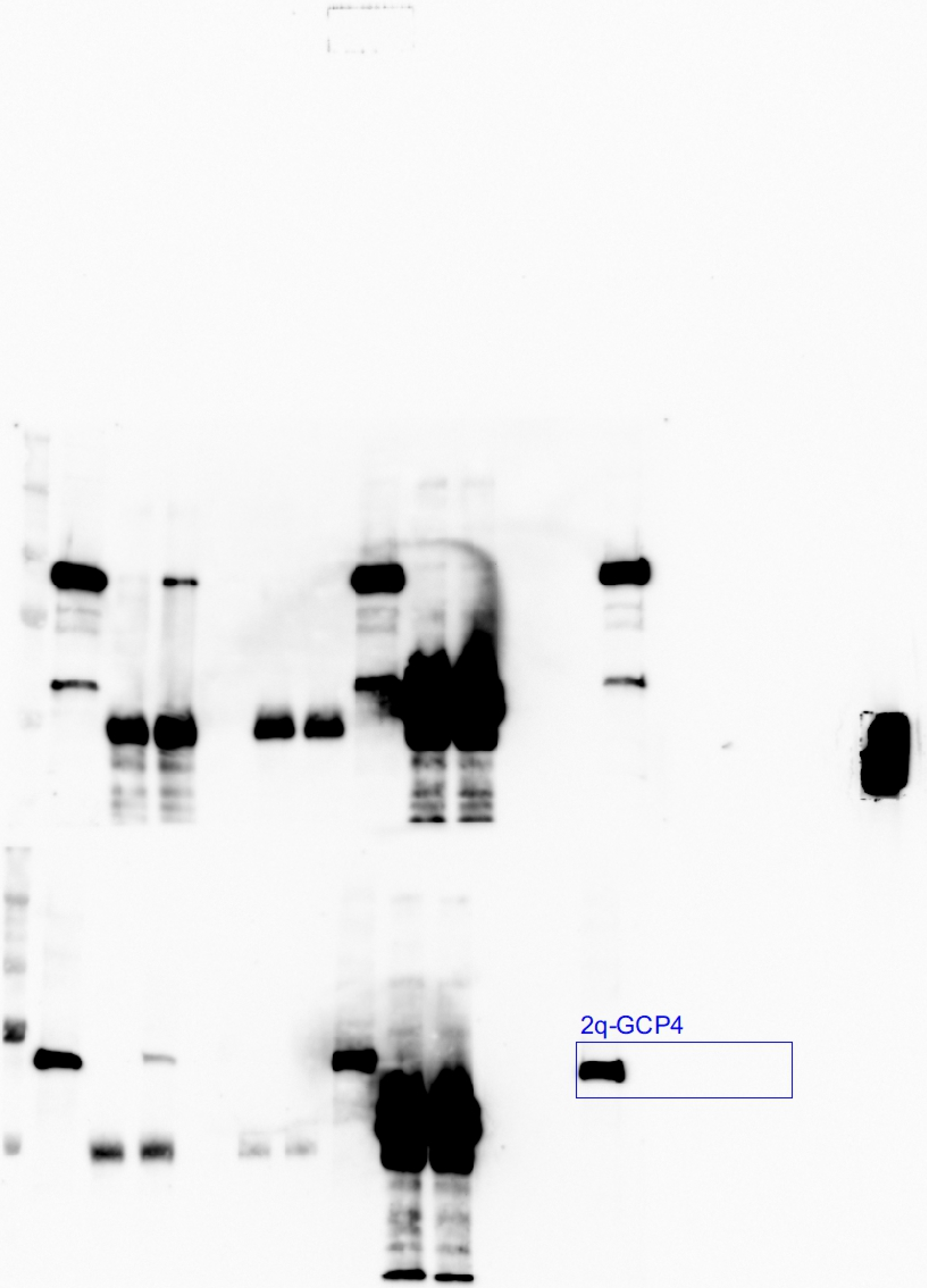
2q-GCP4

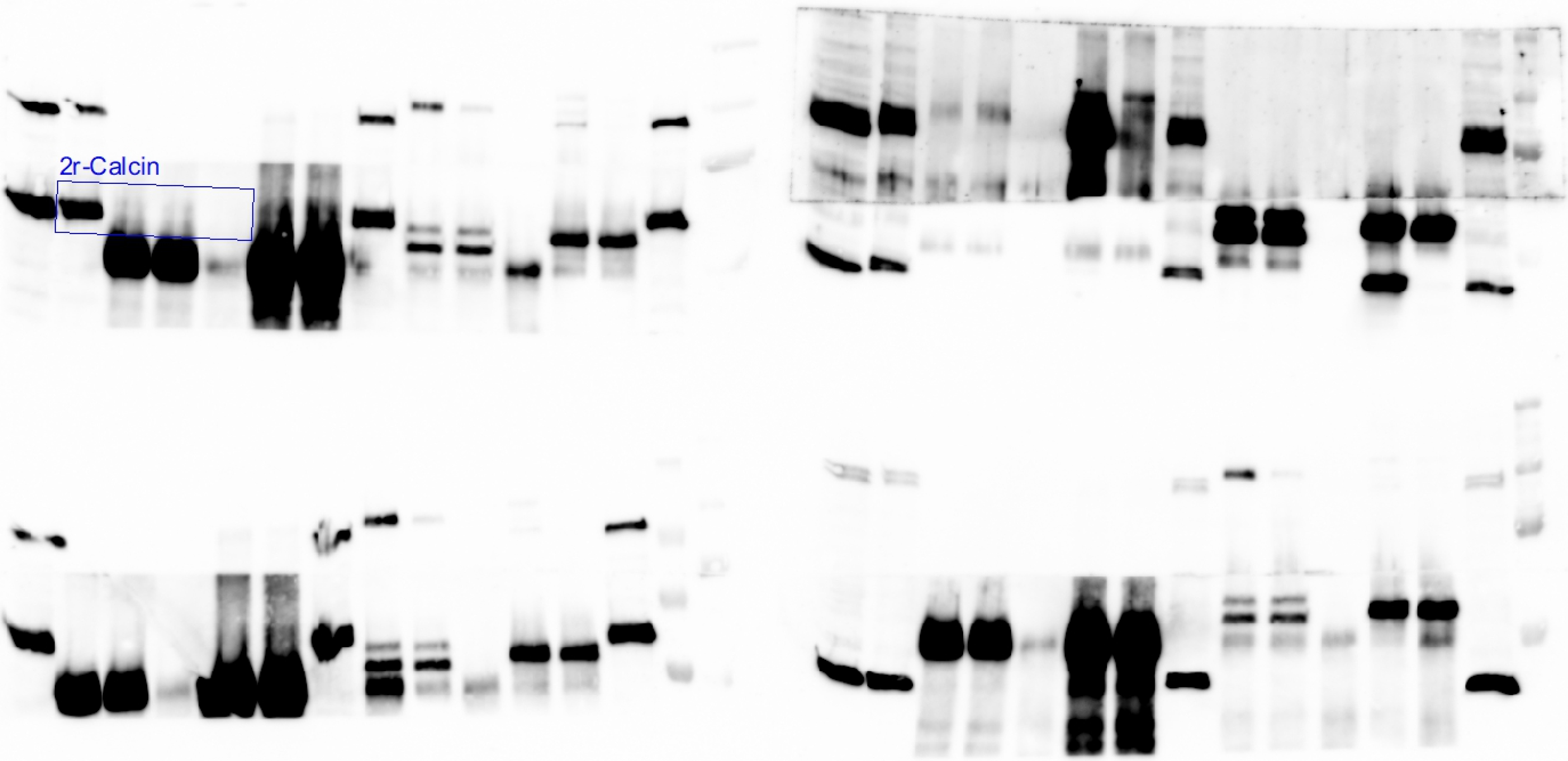
2r-Calcin

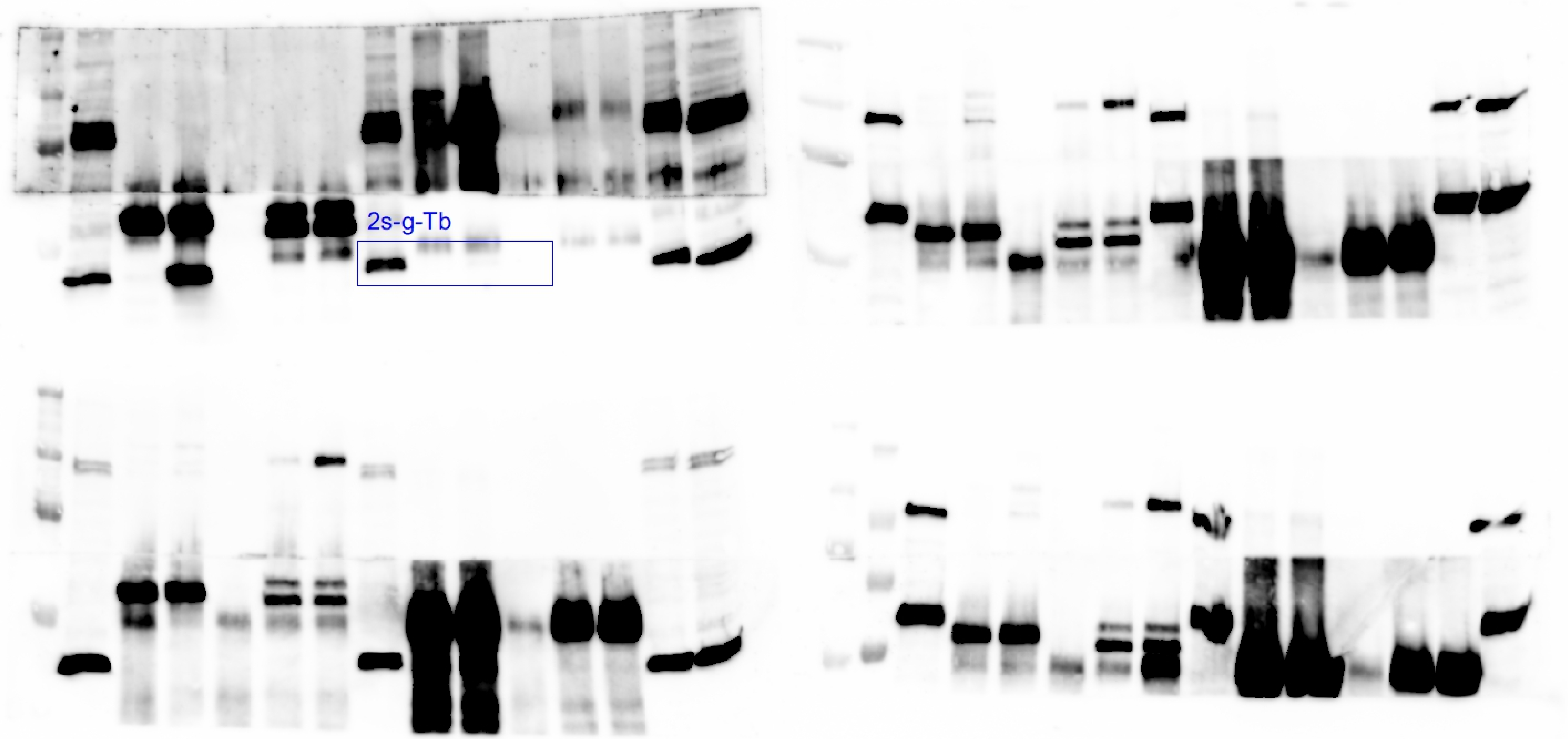

2s-g-Tb

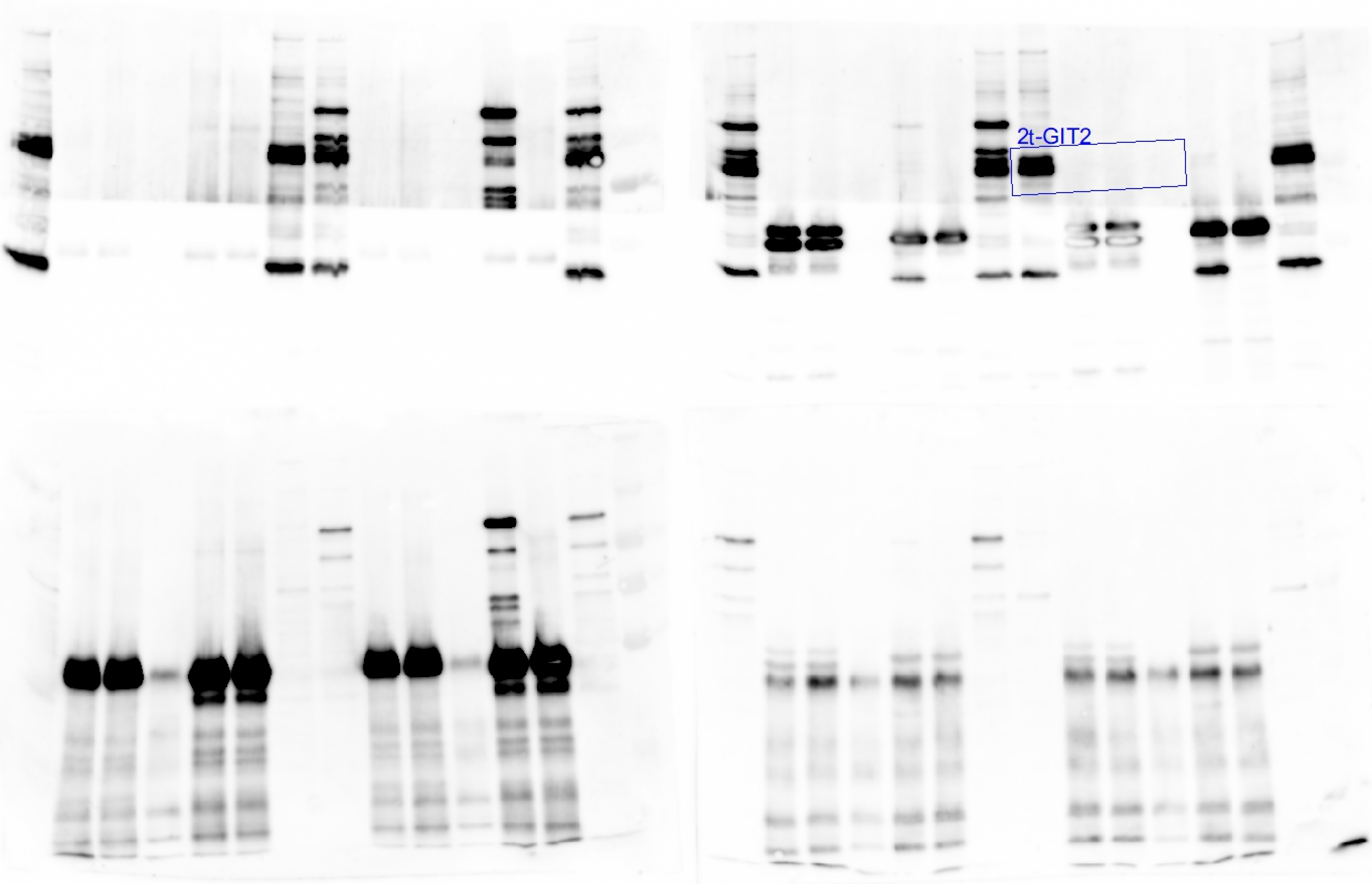
2t-GIT2

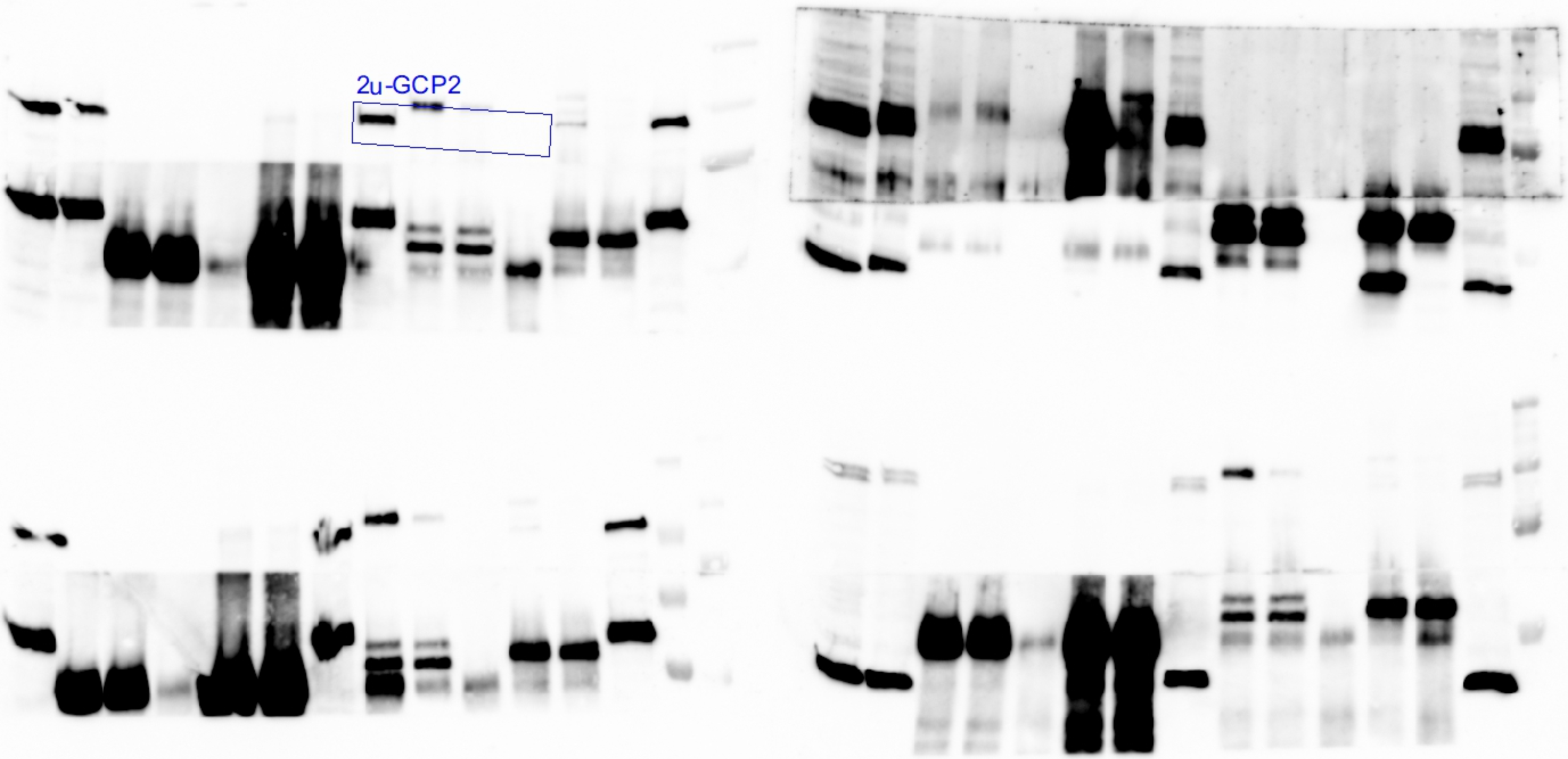
2u-GCP2

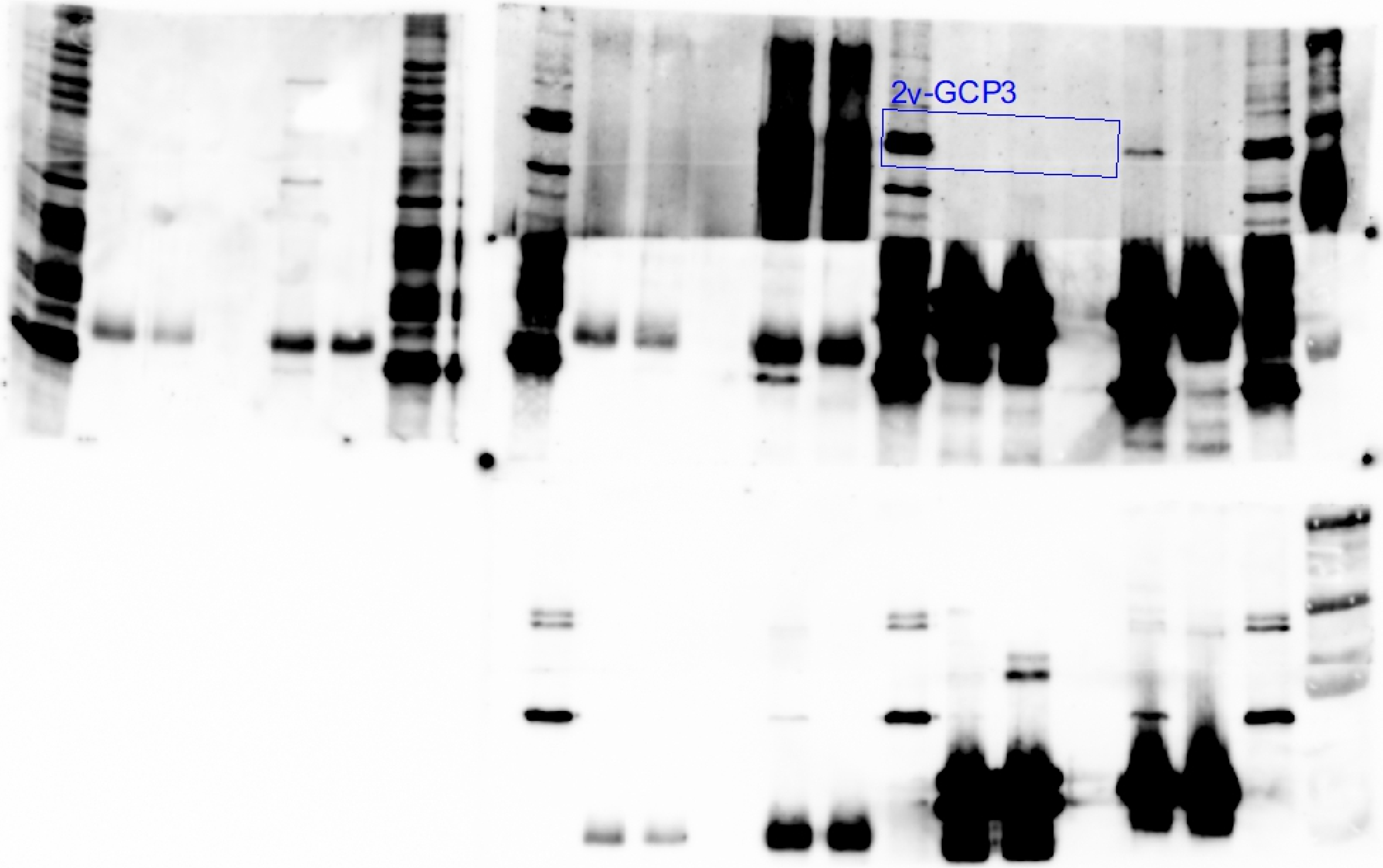
2v-GCP3

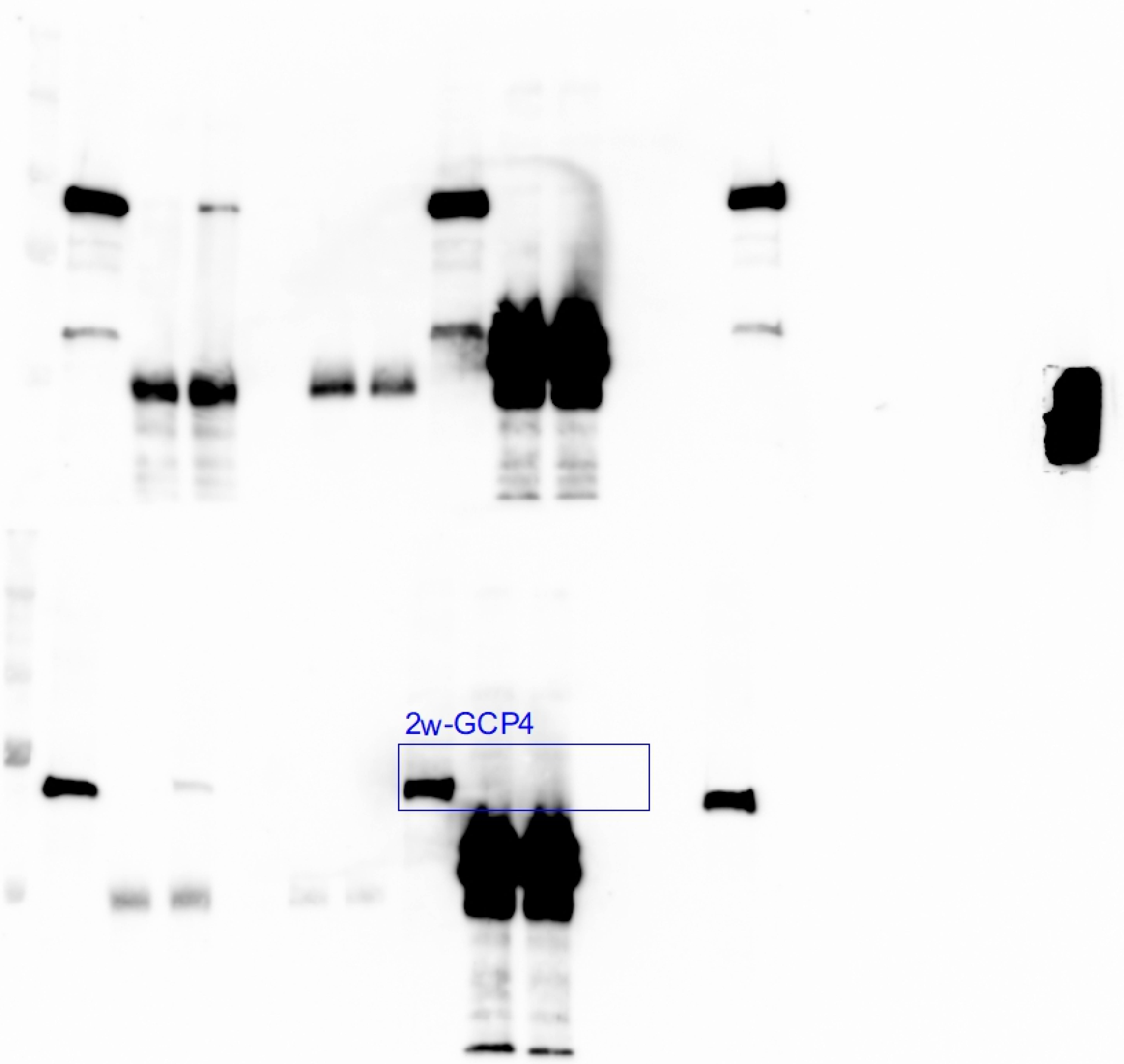
2w-GCP4

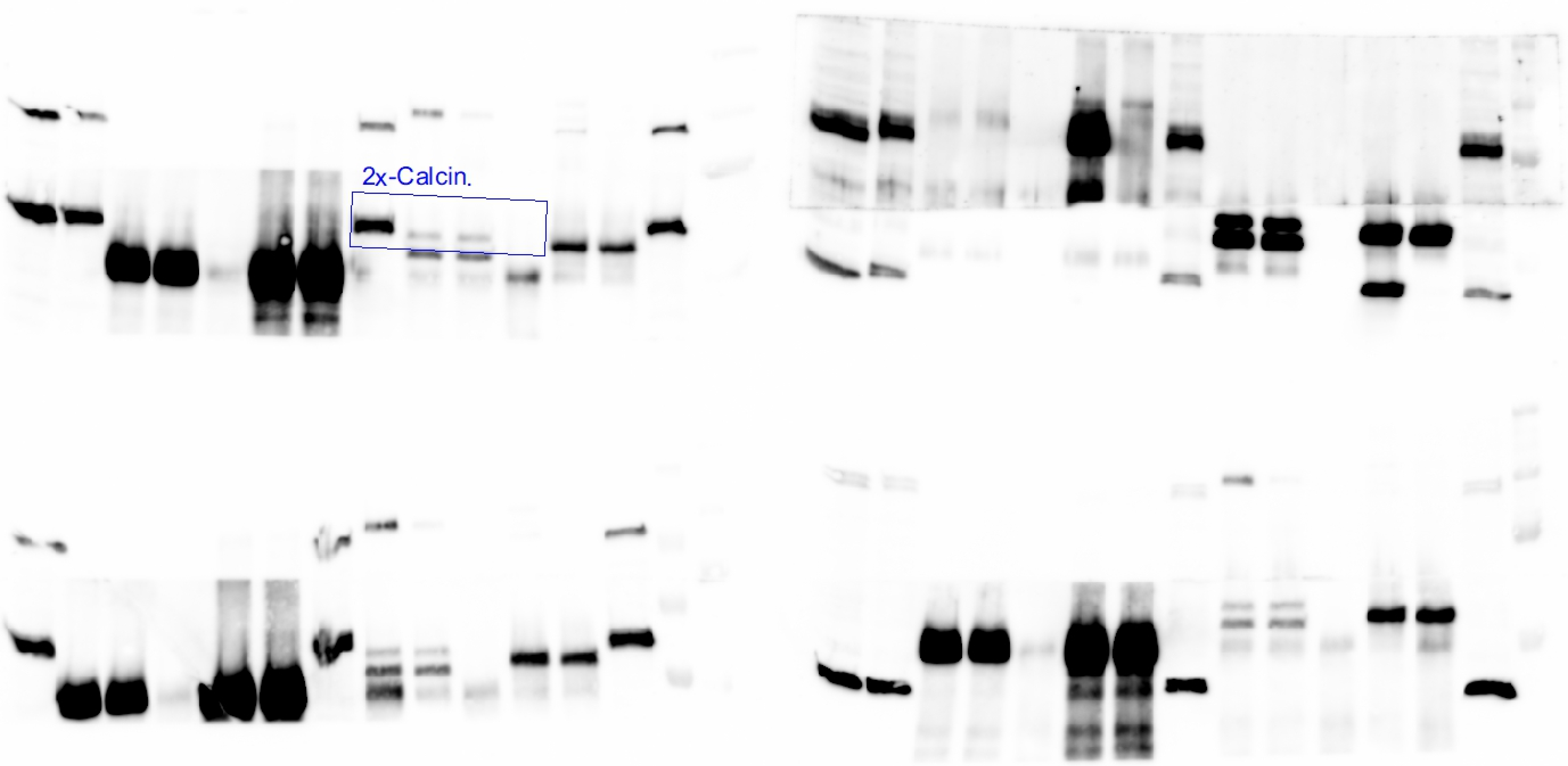
2x-Calcin.
