# Supplementary material for: Regulation of microtubule nucleation in mouse bone marrow-derived mast cells by ARF GTPase-activating protein GIT2
Source: Front Immunol. 2024 Feb 2;15:1321321. doi: 10.3389/fimmu.2024.1321321 (PMC10870779; doi:10.3389/fimmu.2024.1321321)
Supplement: Supplementary file 2 [file Figure 3-originals.pdf › Data Sheet 7/Figure 3-originals.pdf]

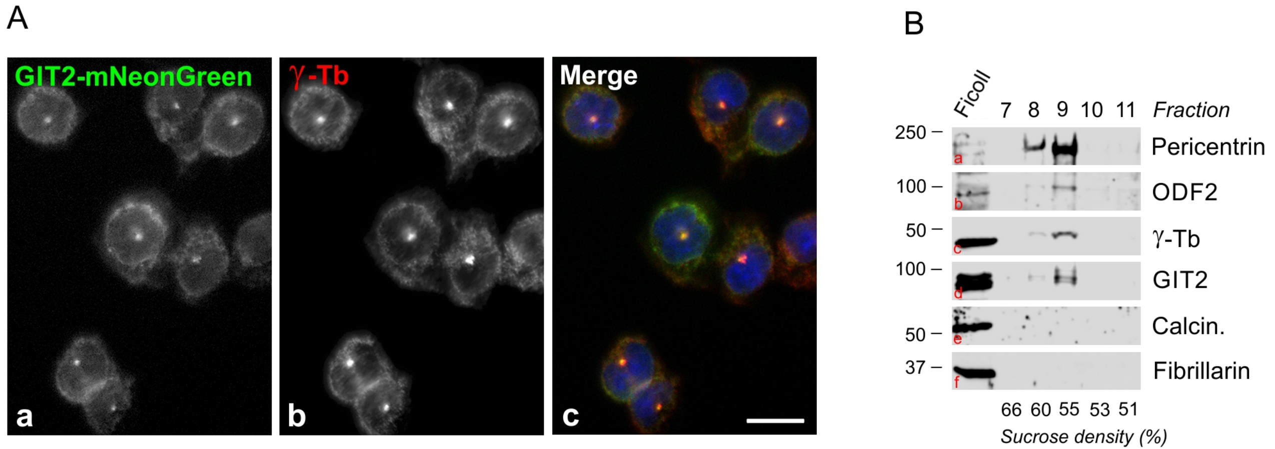
A
GIT2-mNeonGreen
γ-Tb
Merge
a
b
c
B
Ficoll
7
8
9
10
11
Fraction
250
100
50
100
50
37
Pericentrin
ODF2
γ-Tb
GIT2
Calcin.
Fibrillarin
66
60
55
53
51
Sucrose density (%)

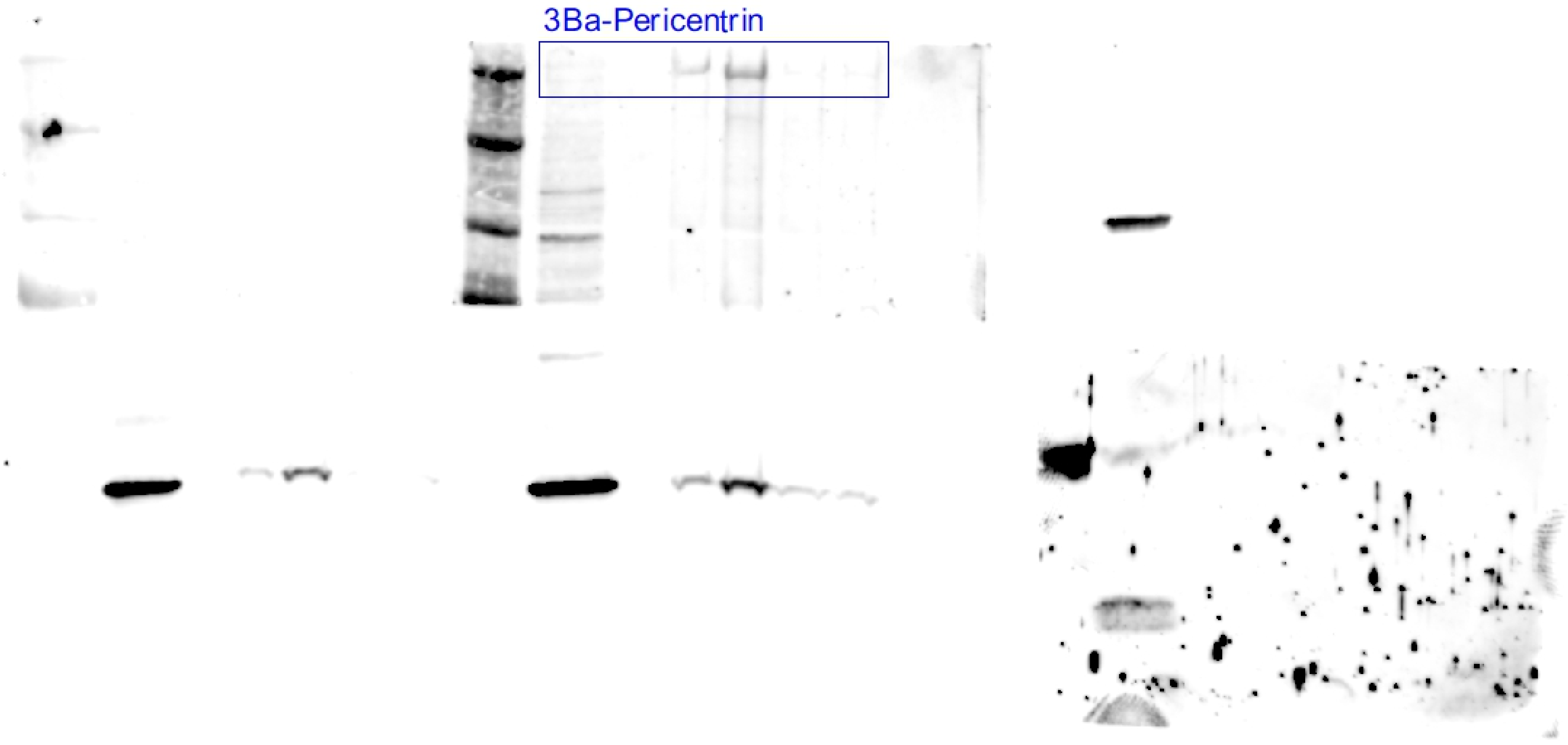
3Ba-Pericentrin

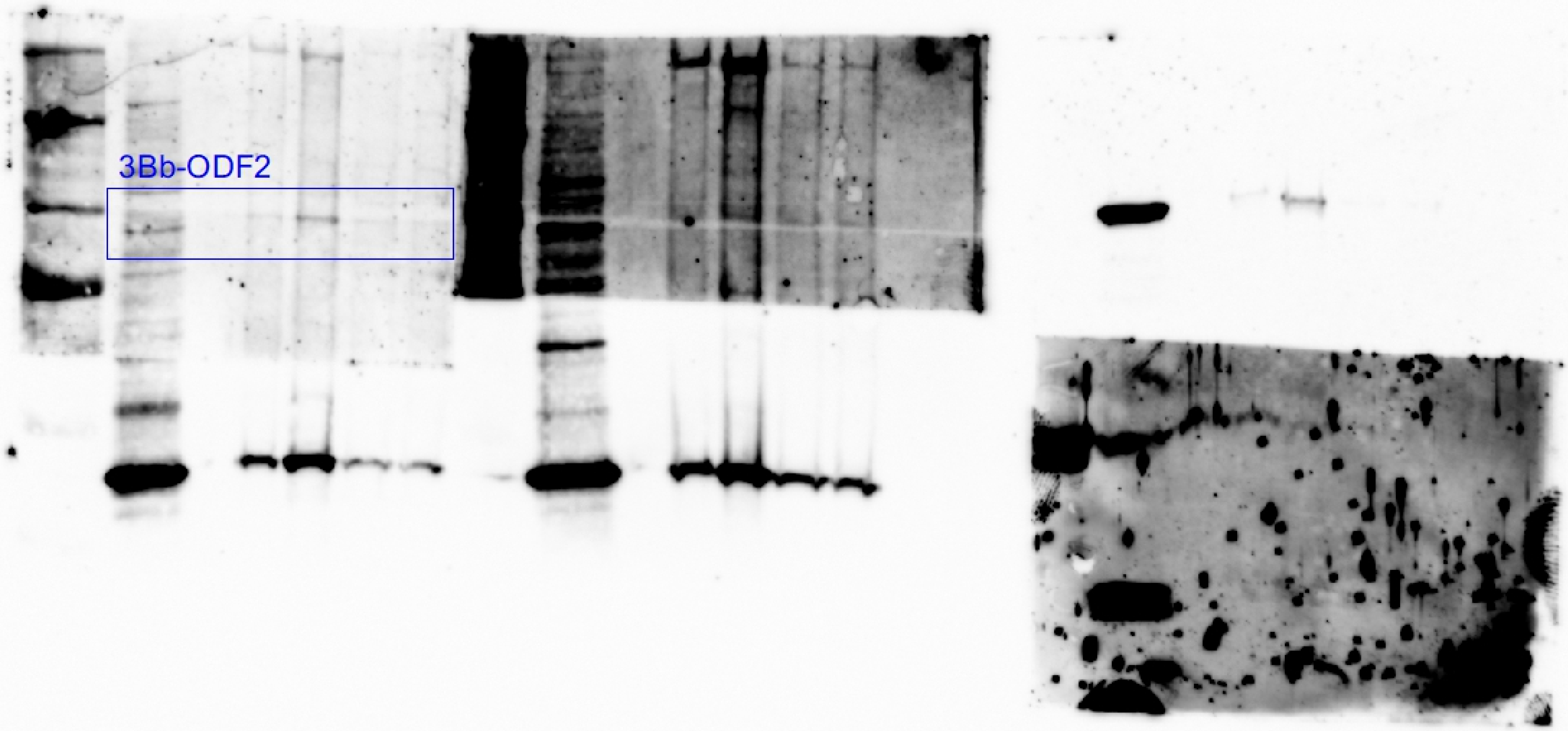
3Bb-ODF2

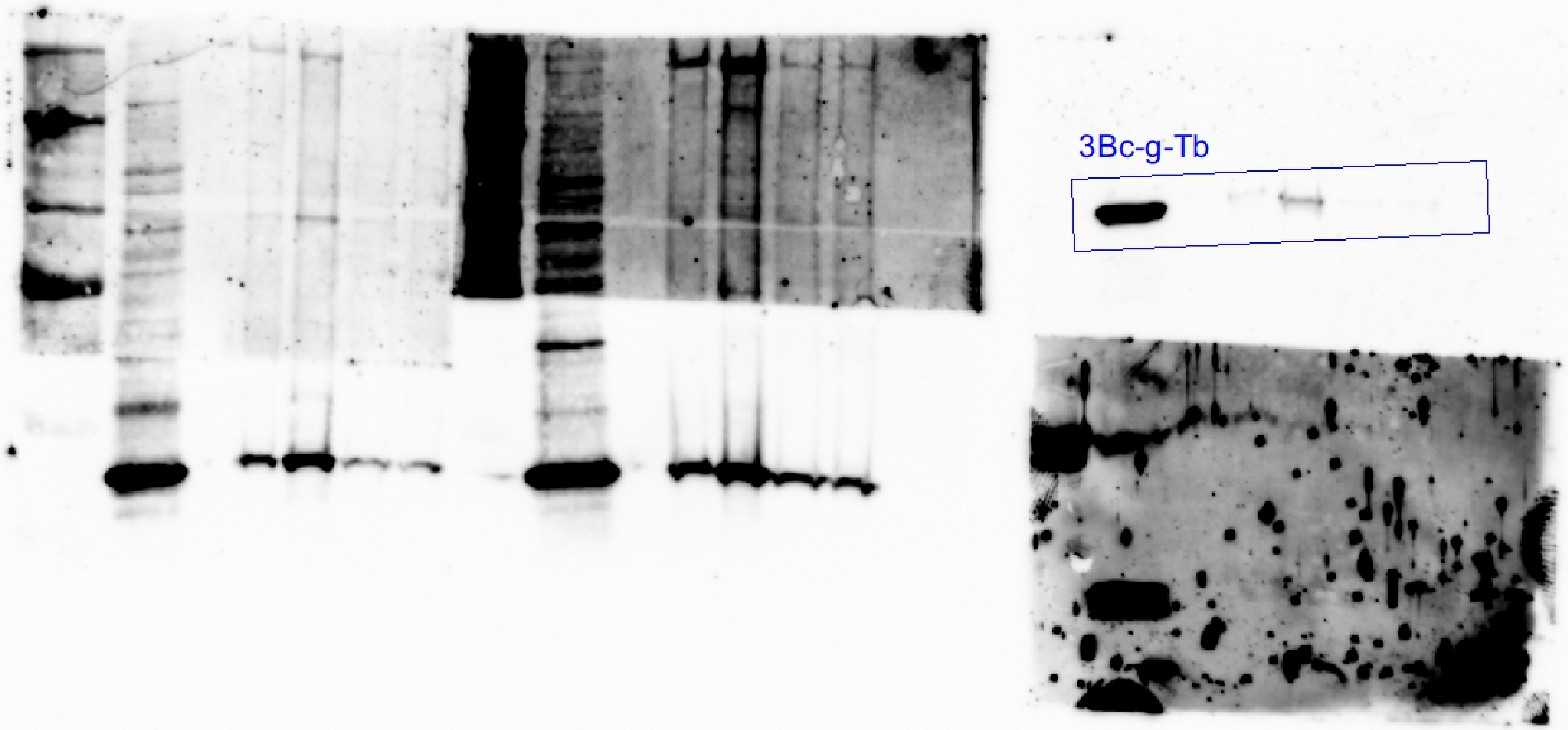
3Bc-g-Tb

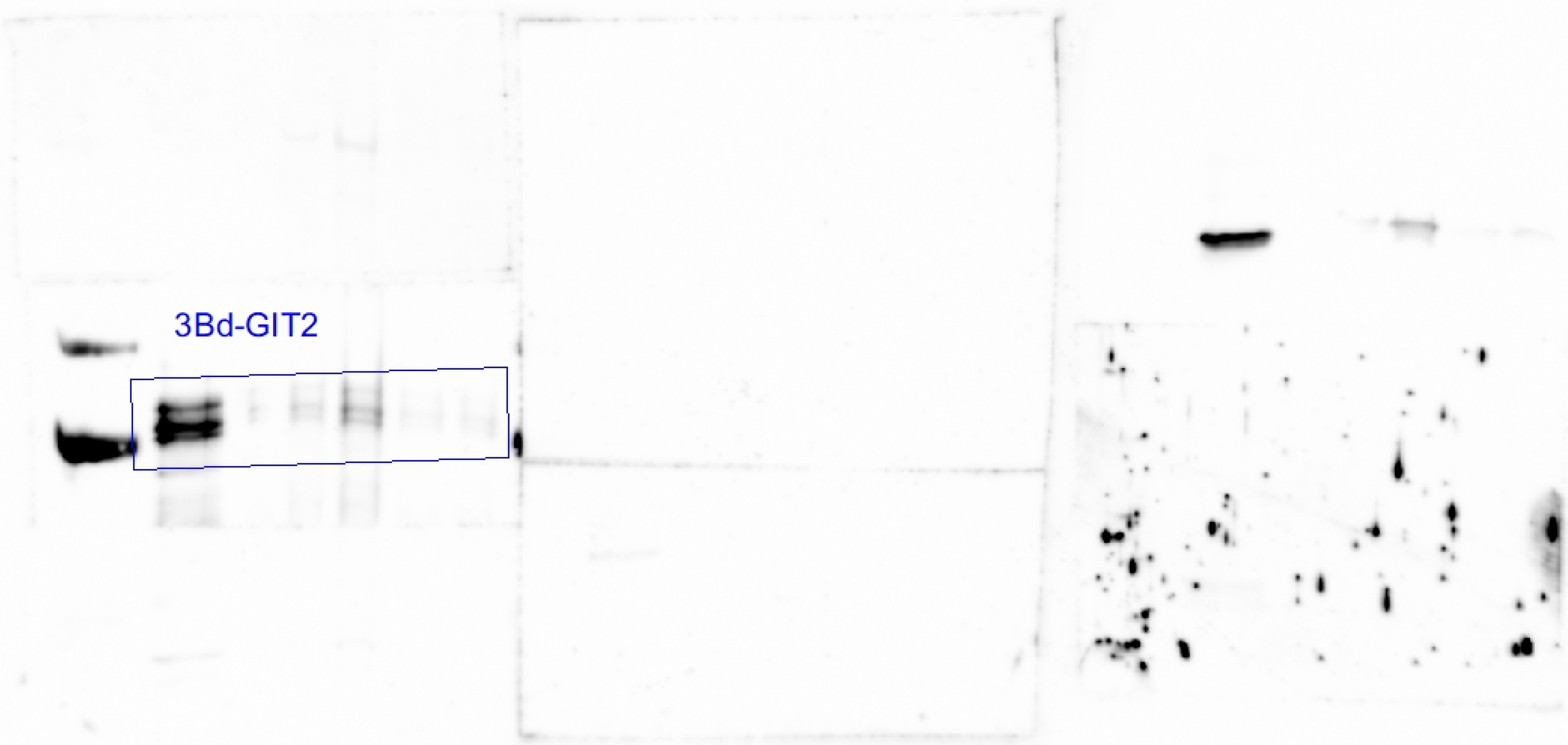
3Bd-GIT2

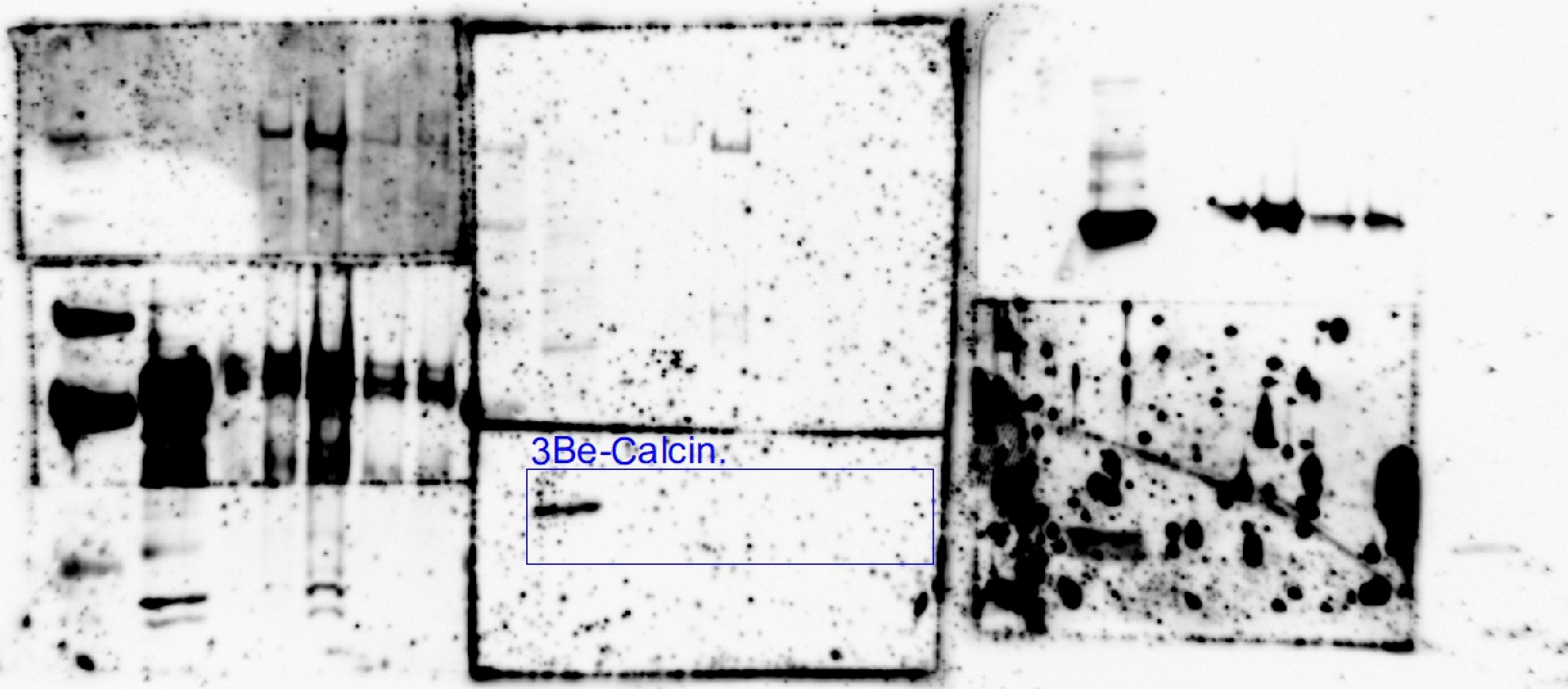
3Be-Calcin.

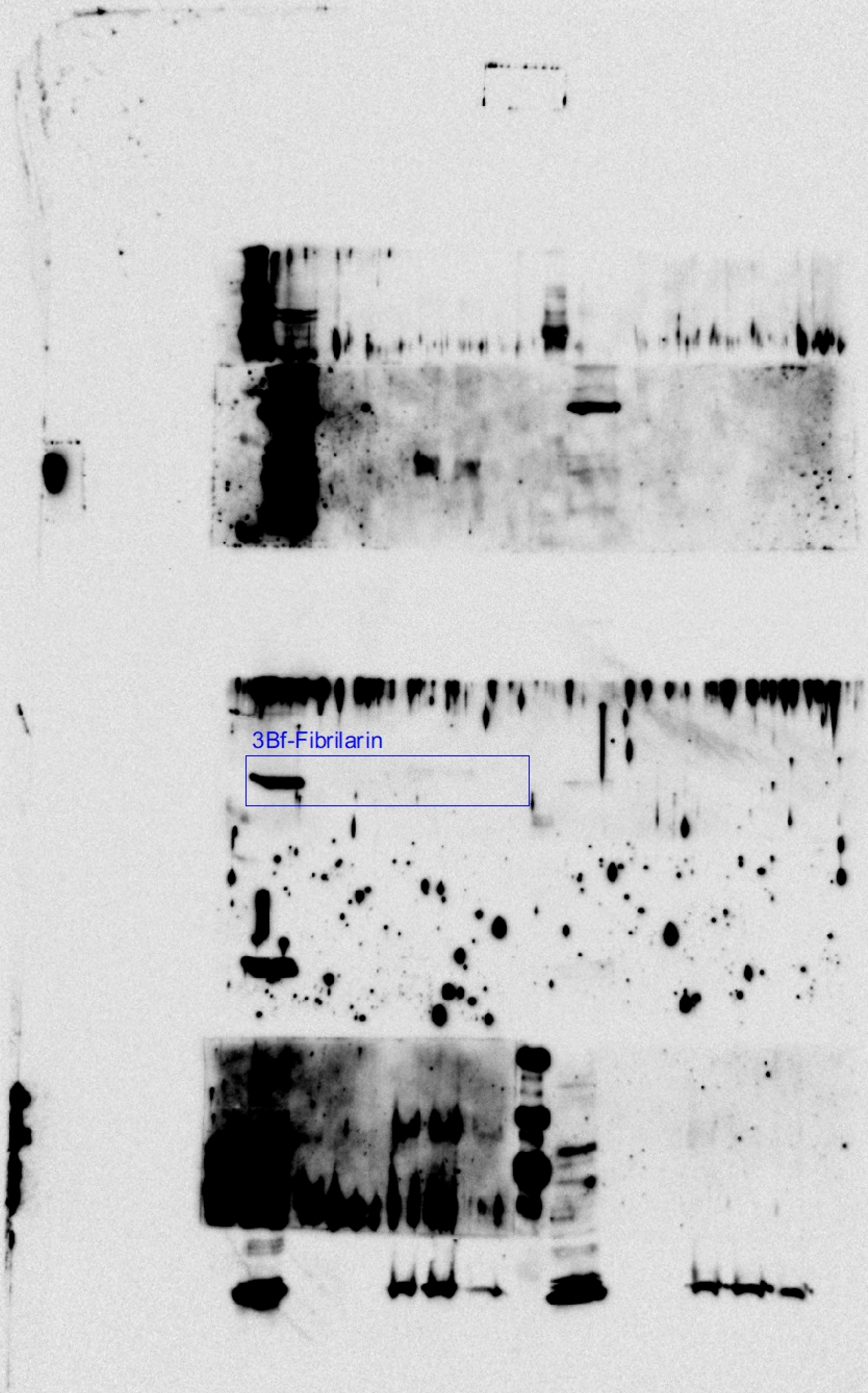
3Bf-Fibrilarin
