# Supplementary material for: Regulation of microtubule nucleation in mouse bone marrow-derived mast cells by ARF GTPase-activating protein GIT2
Source: Front Immunol. 2024 Feb 2;15:1321321. doi: 10.3389/fimmu.2024.1321321 (PMC10870779; doi:10.3389/fimmu.2024.1321321)

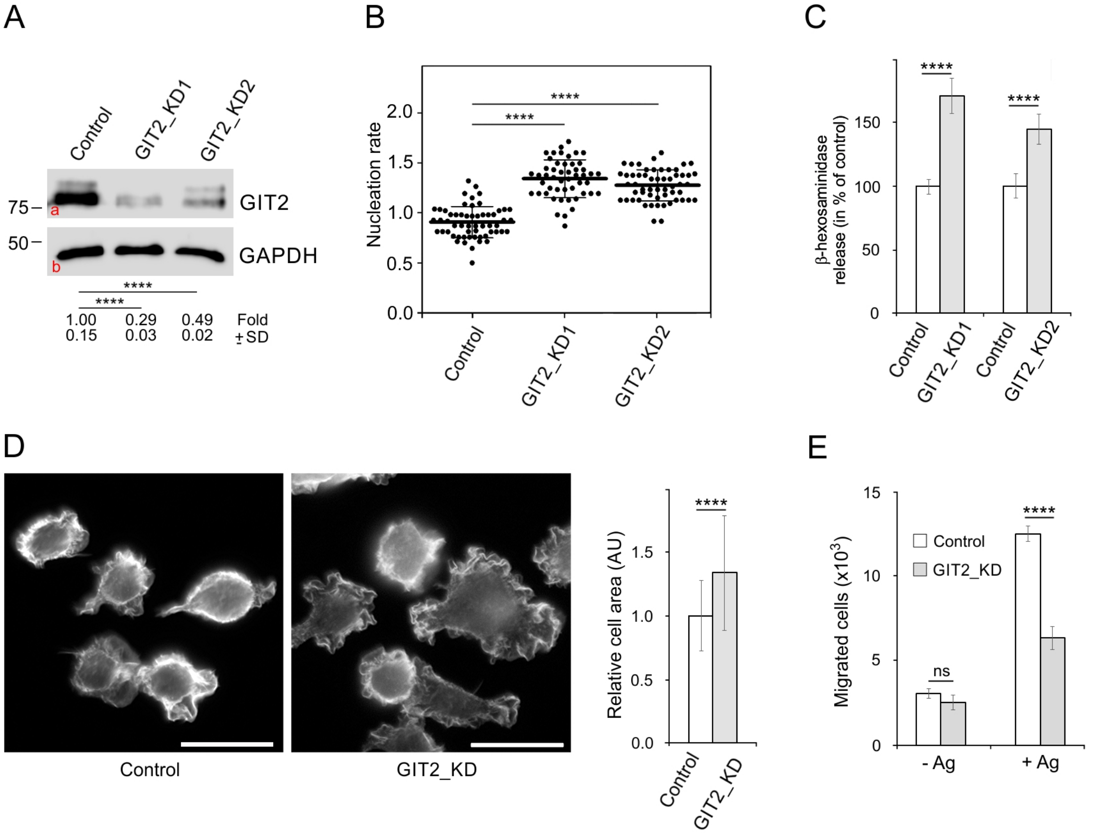

A
Control
GIT2_KD1
GIT2_KD2
75
GIT2
50
GAPDH
a
b
****
****
1.00 0.29 0.49 Fold
0.15 0.03 0.02 ± SD
B
Nucleation rate
0.0
0.5
1.0
1.5
2.0
****
****
Control
GIT2_KD1
GIT2_KD2
C
β-hexosaminidase release (in % of control)
0
50
100
150
****
****
Control
GIT2_KD1
Control
GIT2_KD2
D
Control
GIT2_KD
Relative cell area (AU)
0.0
0.5
1.0
1.5
****
Control
GIT2_KD
E
Migrated cells (x10³)
0
5
10
15
Control
GIT2_KD
ns
****
- Ag
+ Ag

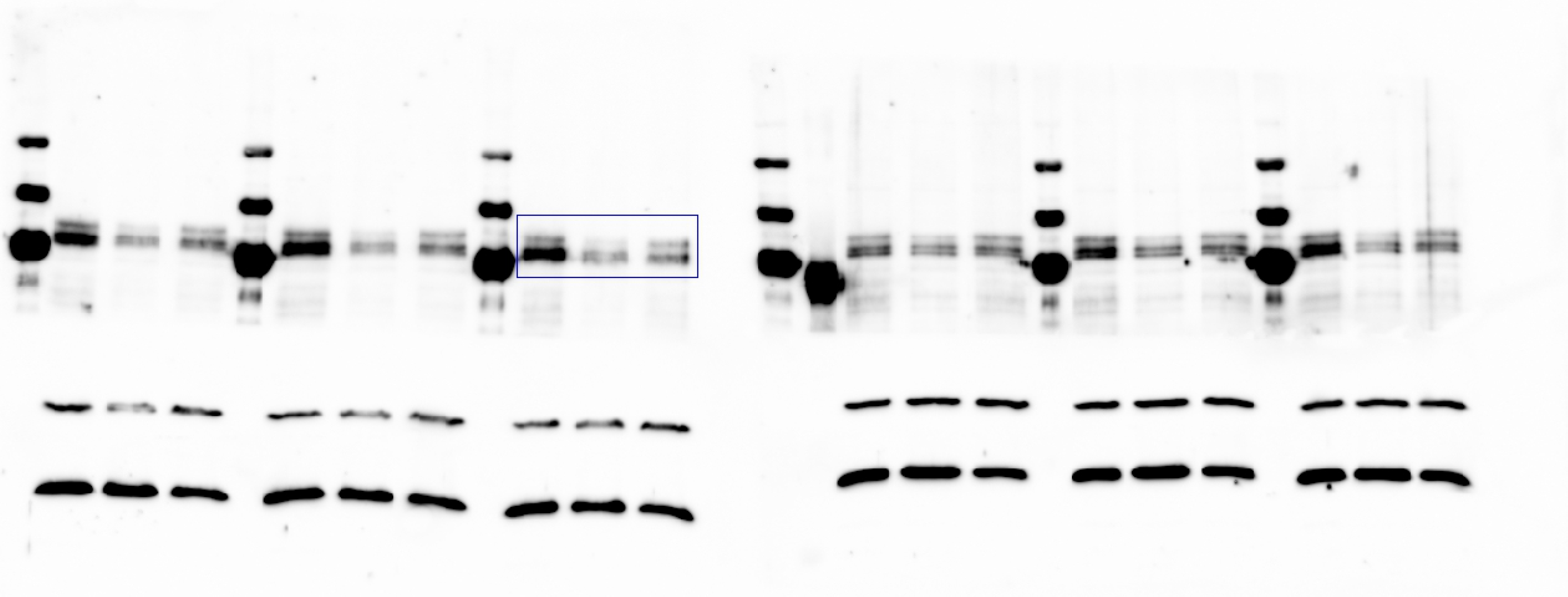

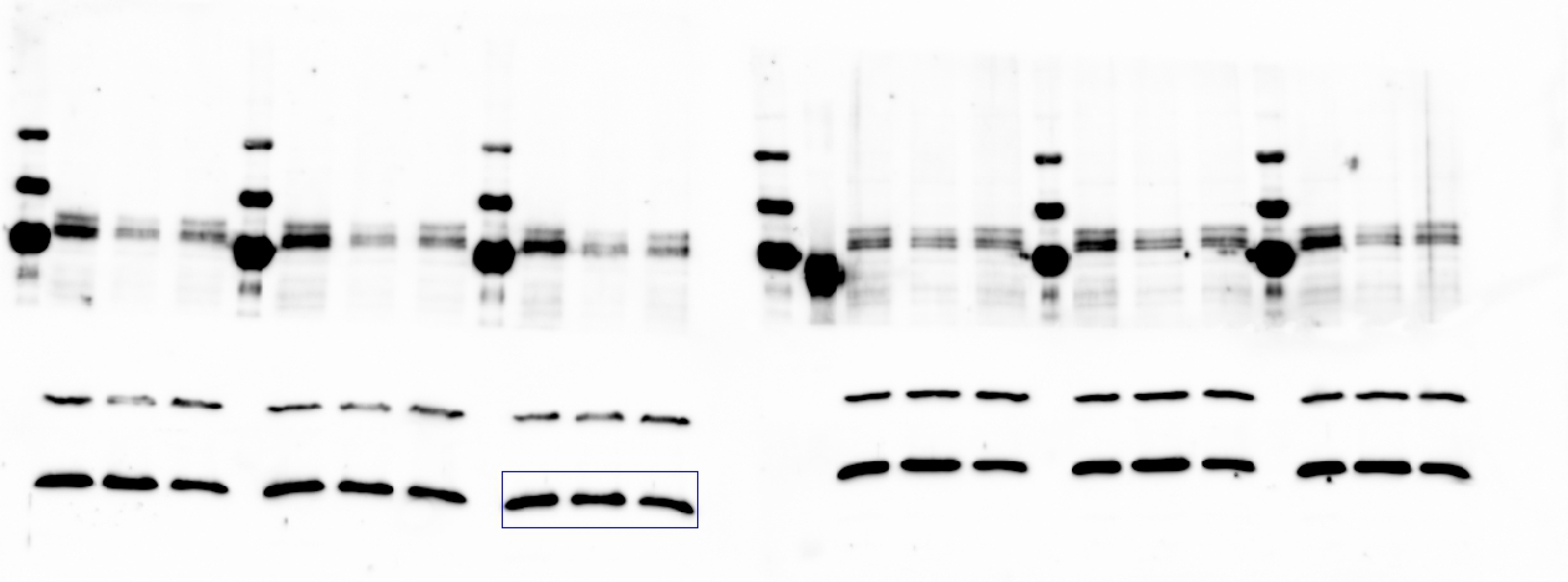

Supplement: Supplementary file 2 [file Figure 4-originals_v2.pdf › Data Sheet 7/Figure 4-originals_v2.pdf]
