# Supplementary material for: Regulation of microtubule nucleation in mouse bone marrow-derived mast cells by ARF GTPase-activating protein GIT2
Source: Front Immunol. 2024 Feb 2;15:1321321. doi: 10.3389/fimmu.2024.1321321 (PMC10870779; doi:10.3389/fimmu.2024.1321321)
Supplement: Supplementary file 2 [file Figure 5-originals.pdf › Data Sheet 7/Figure 5-originals.pdf]

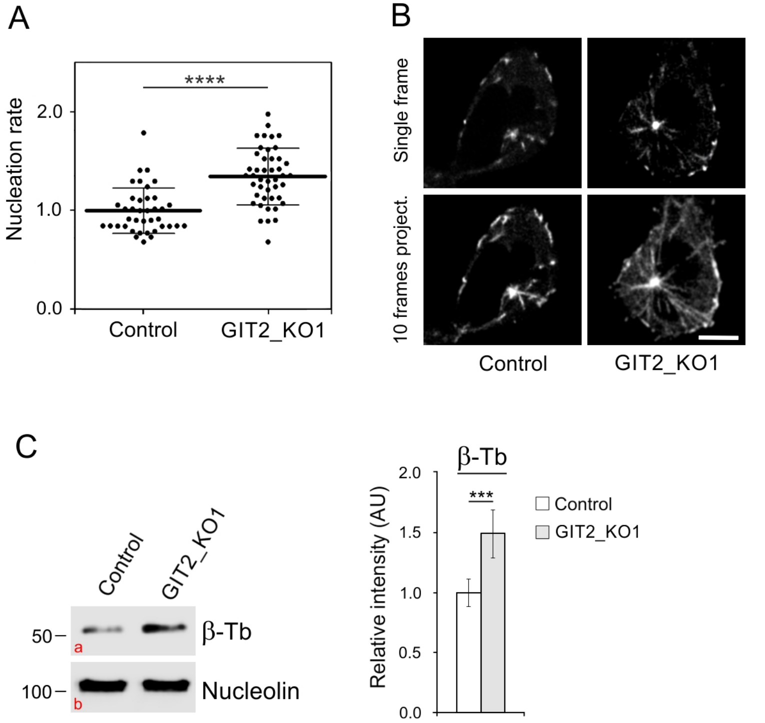
A
****
Nucleation rate
2.0
1.0
0.0
Control
GIT2_KO1
B
Single frame
10 frames project.
Control
GIT2_KO1
C
Control
GIT2_KO1
50
100
a
b
β-Tb
Nucleolin
β-Tb
***
Relative intensity (AU)
2.0
1.5
1.0
0.5
0.0
Control
GIT2_KO1

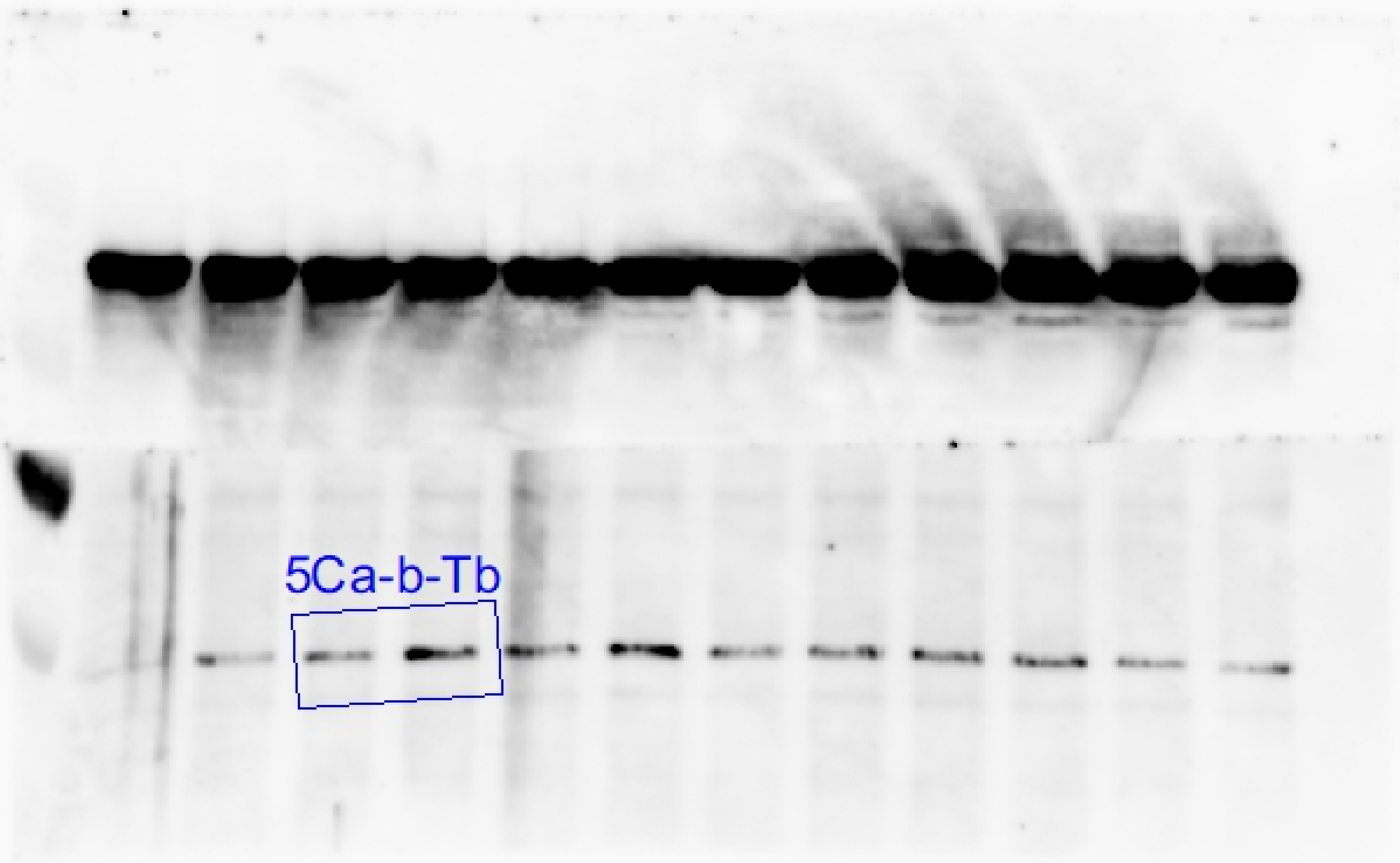
5Ca-b-Tb

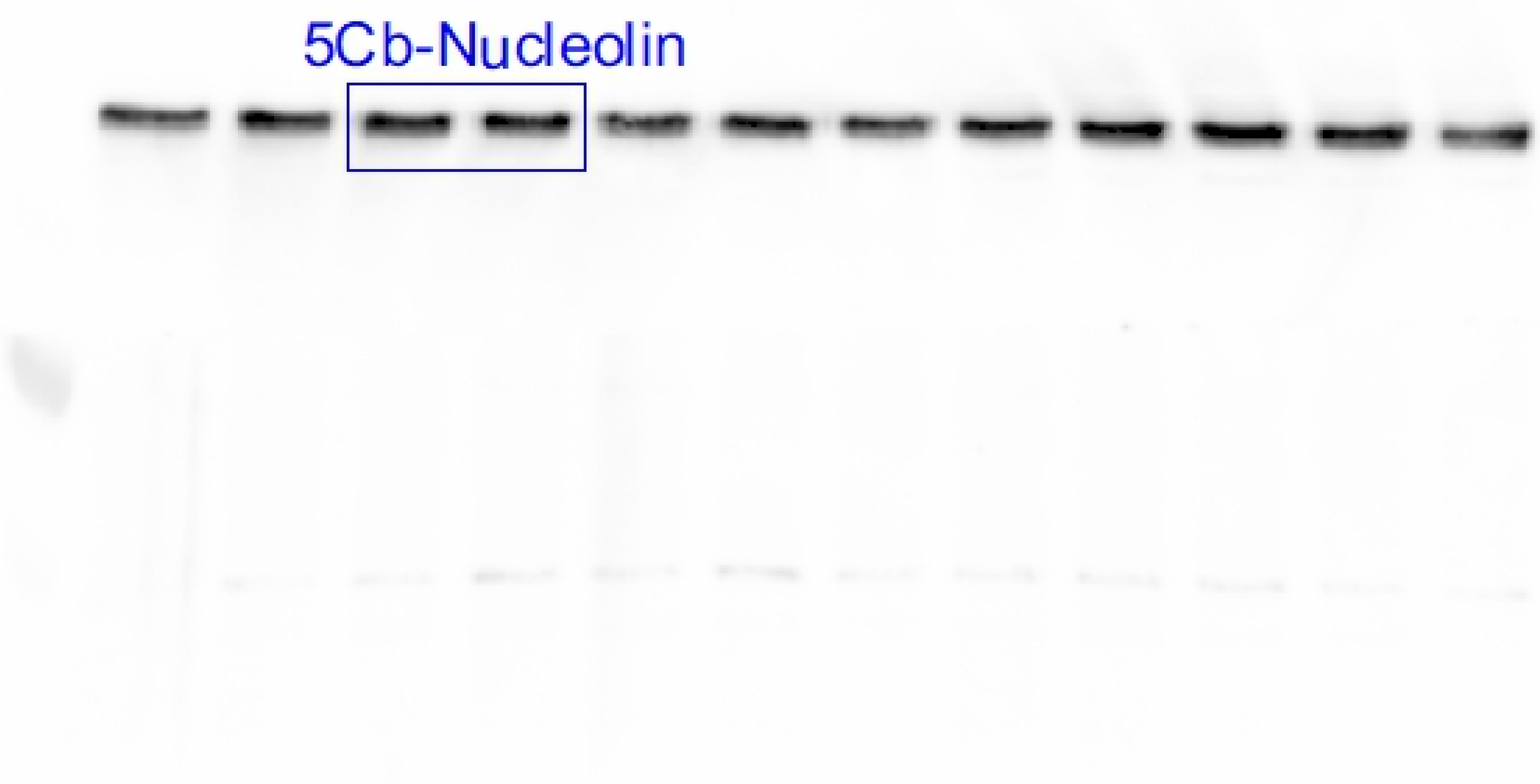
5Cb-Nucleolin
