# Supplementary material for: Regulation of microtubule nucleation in mouse bone marrow-derived mast cells by ARF GTPase-activating protein GIT2
Source: Front Immunol. 2024 Feb 2;15:1321321. doi: 10.3389/fimmu.2024.1321321 (PMC10870779; doi:10.3389/fimmu.2024.1321321)
Supplement: Supplementary file 2 [file Figure 6-originals.pdf › Data Sheet 7/Figure 6-originals.pdf]

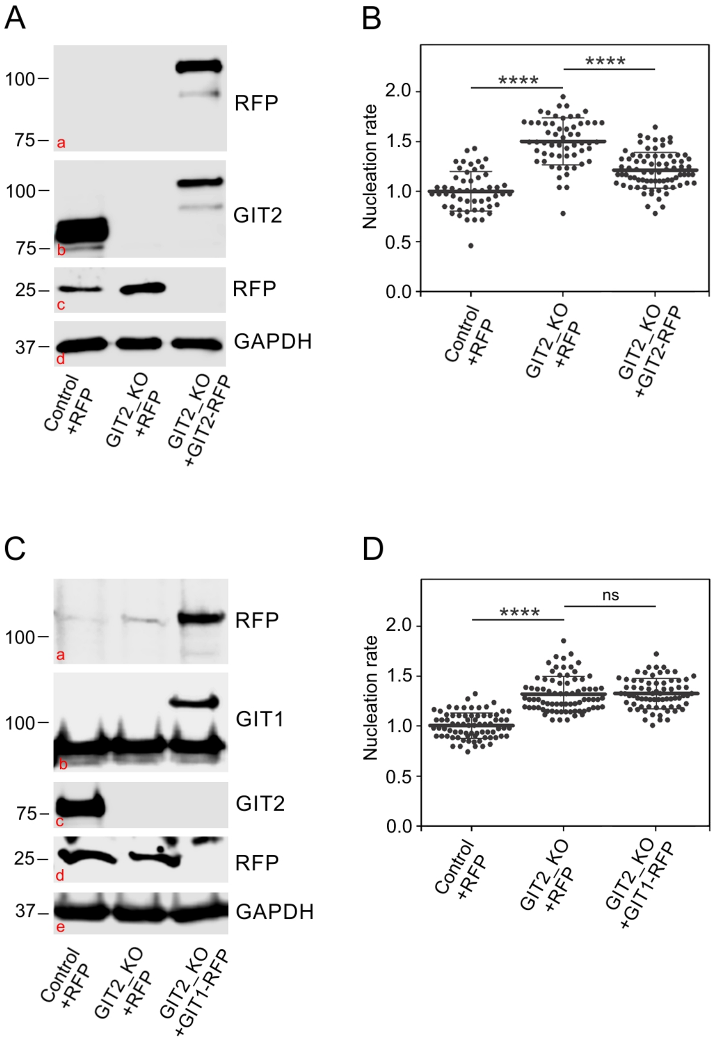
A
B
C
D
RFP
GIT2
RFP
GAPDH
GIT1
100
75
25
37
Nucleation rate
****
ns
Control +RFP
GIT2_KO +RFP
GIT2_KO +GIT2-RFP
GIT2_KO +GIT1-RFP

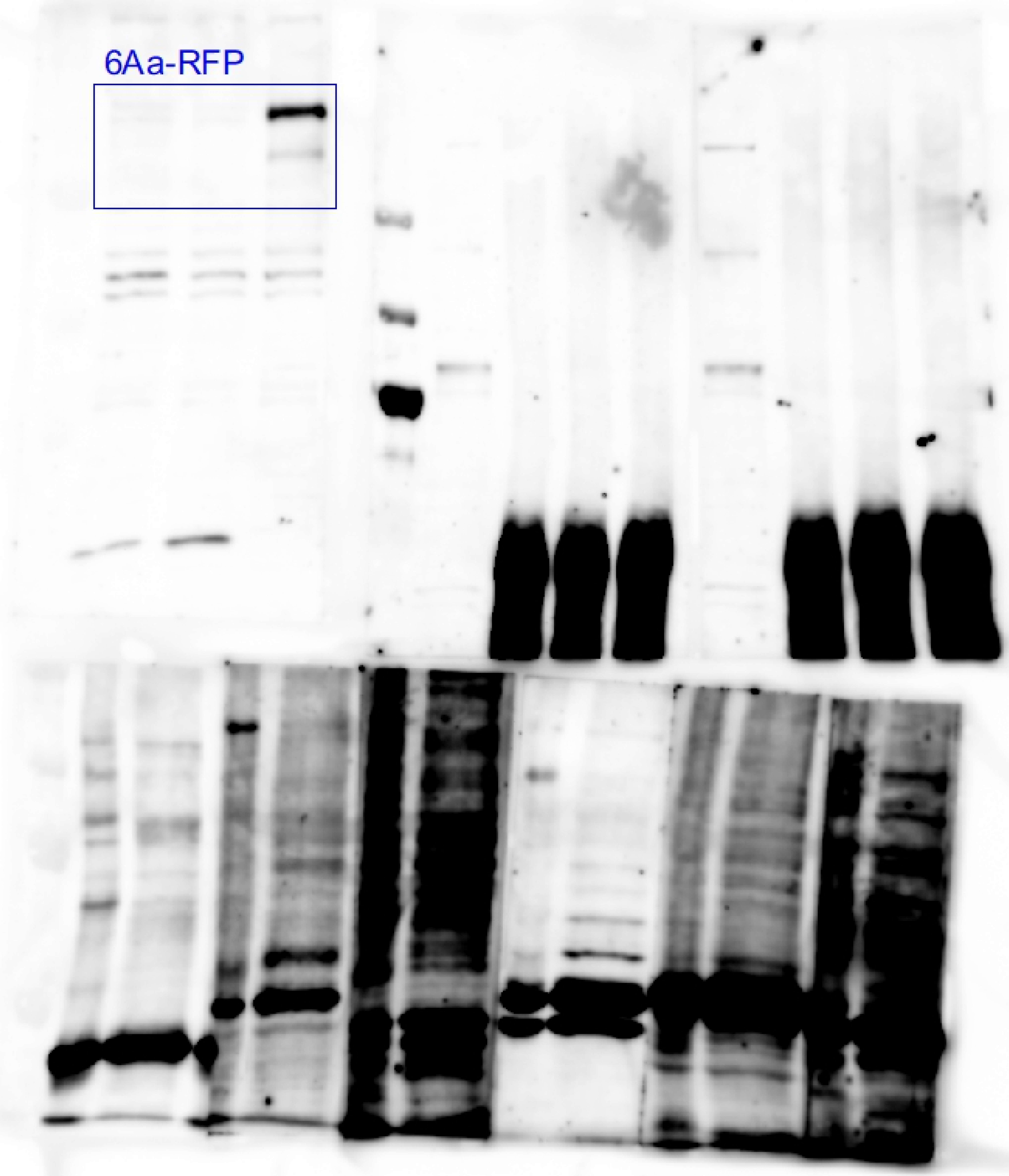
6Aa-RFP

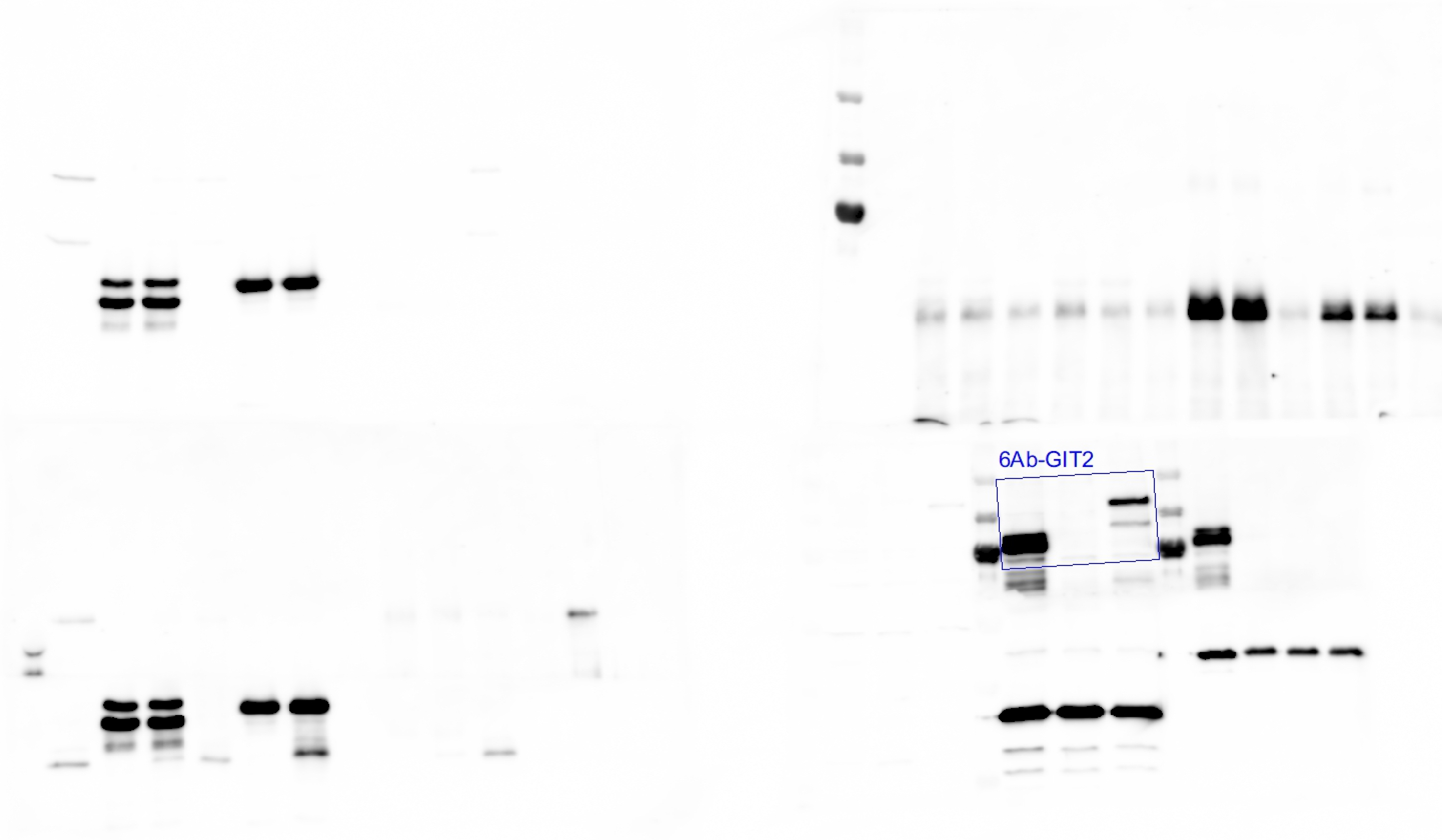
6Ab-GIT2

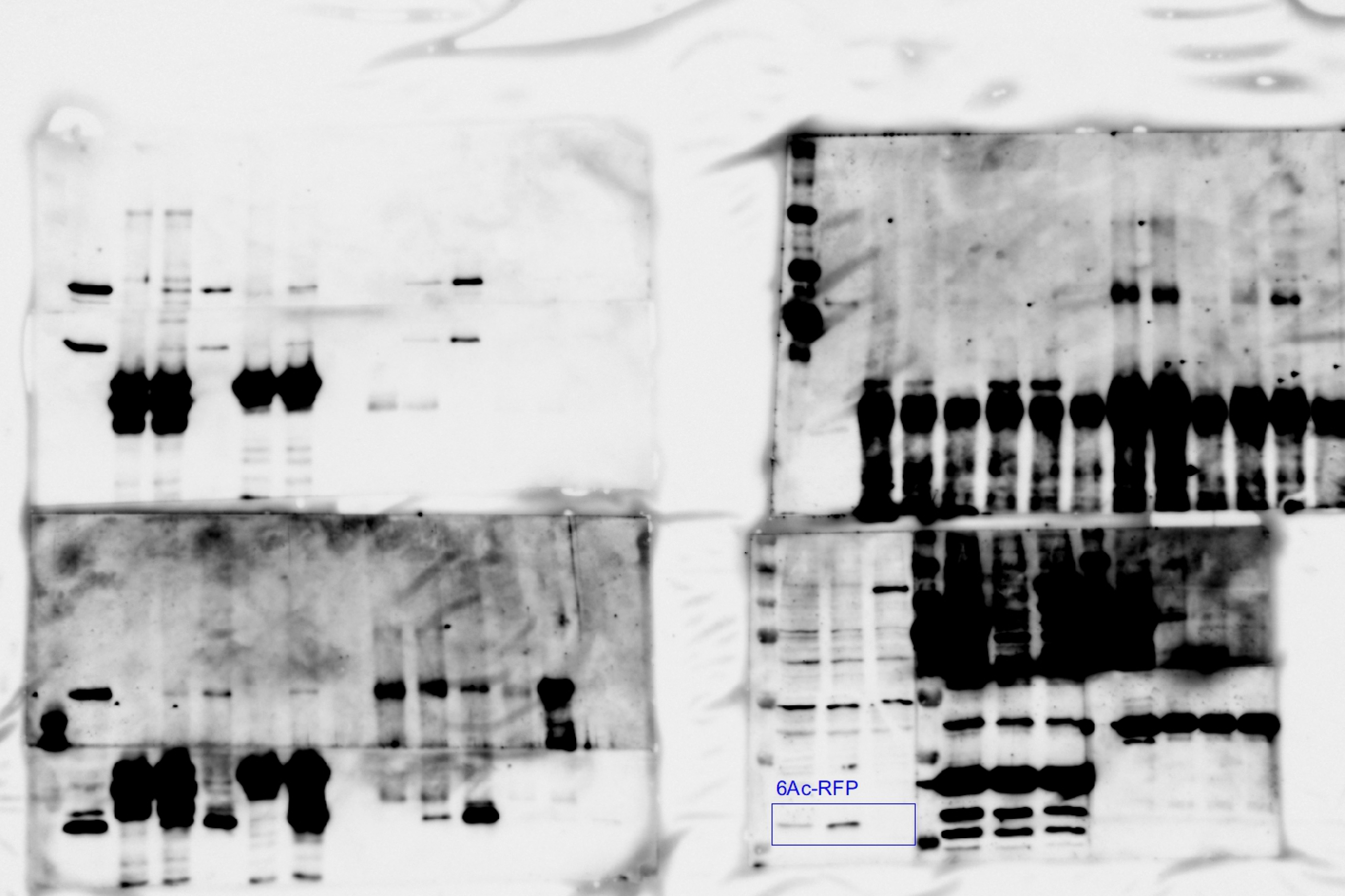
6Ac-RFP

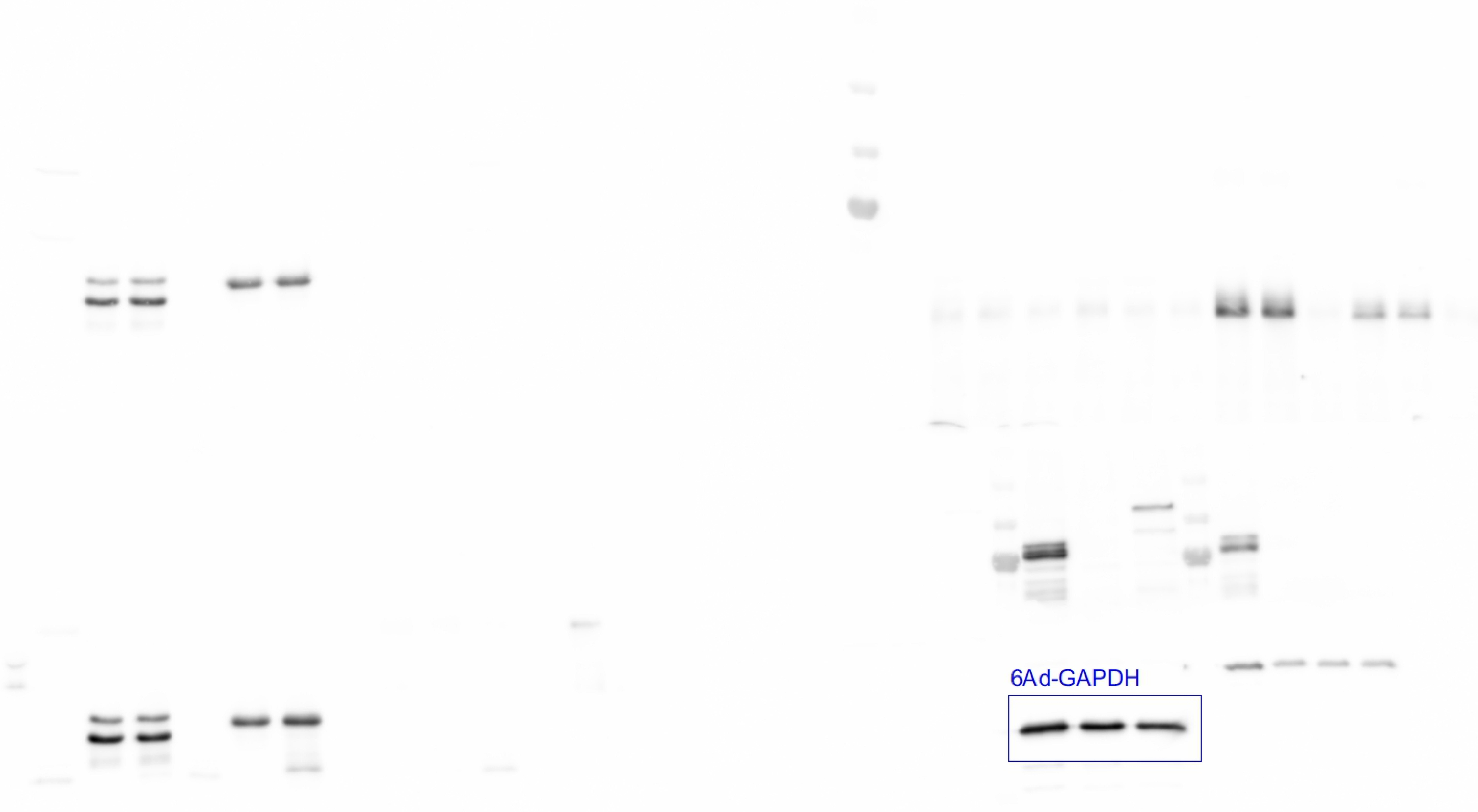
6Ad-GAPDH

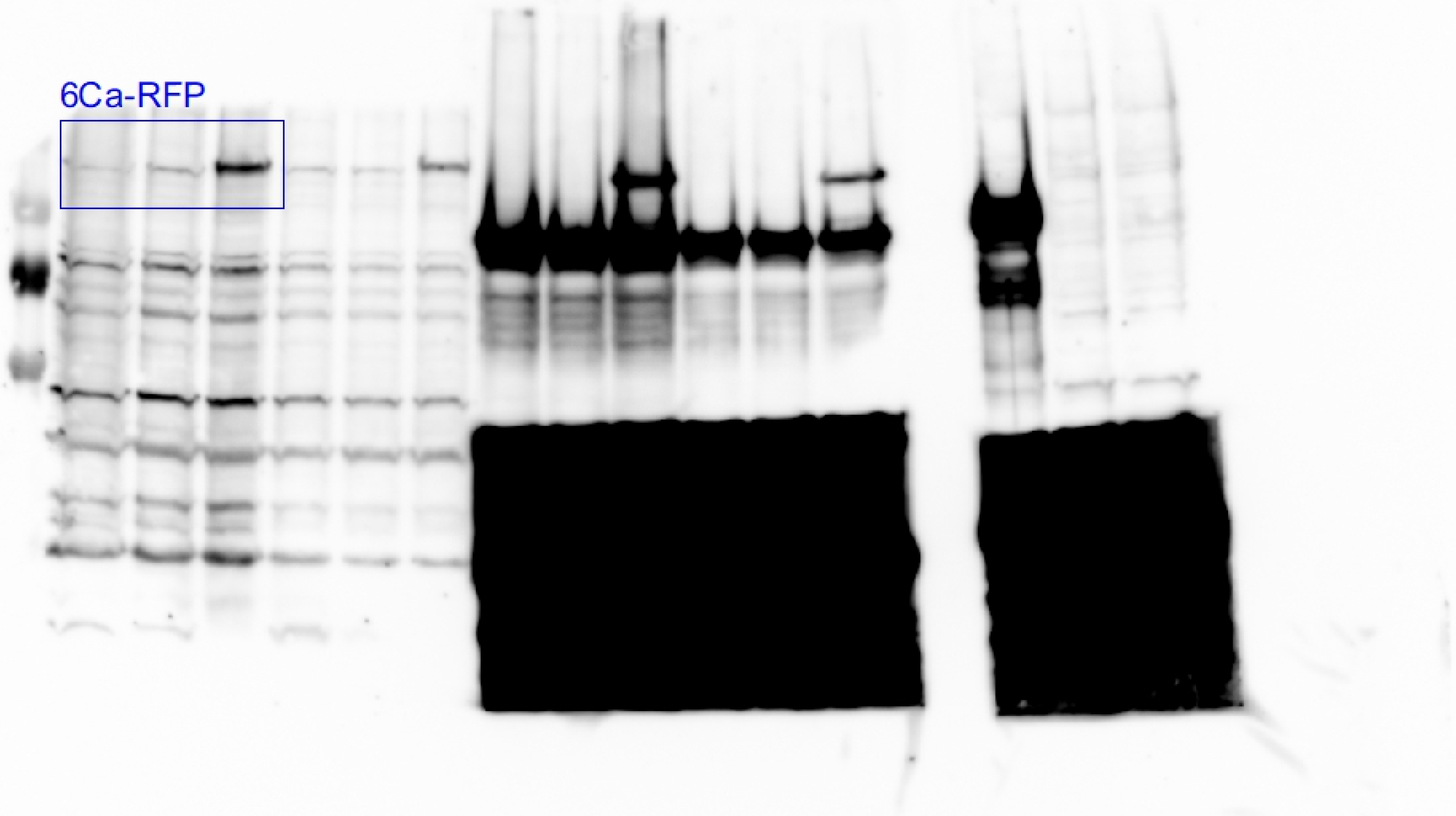

6Ca-RFP

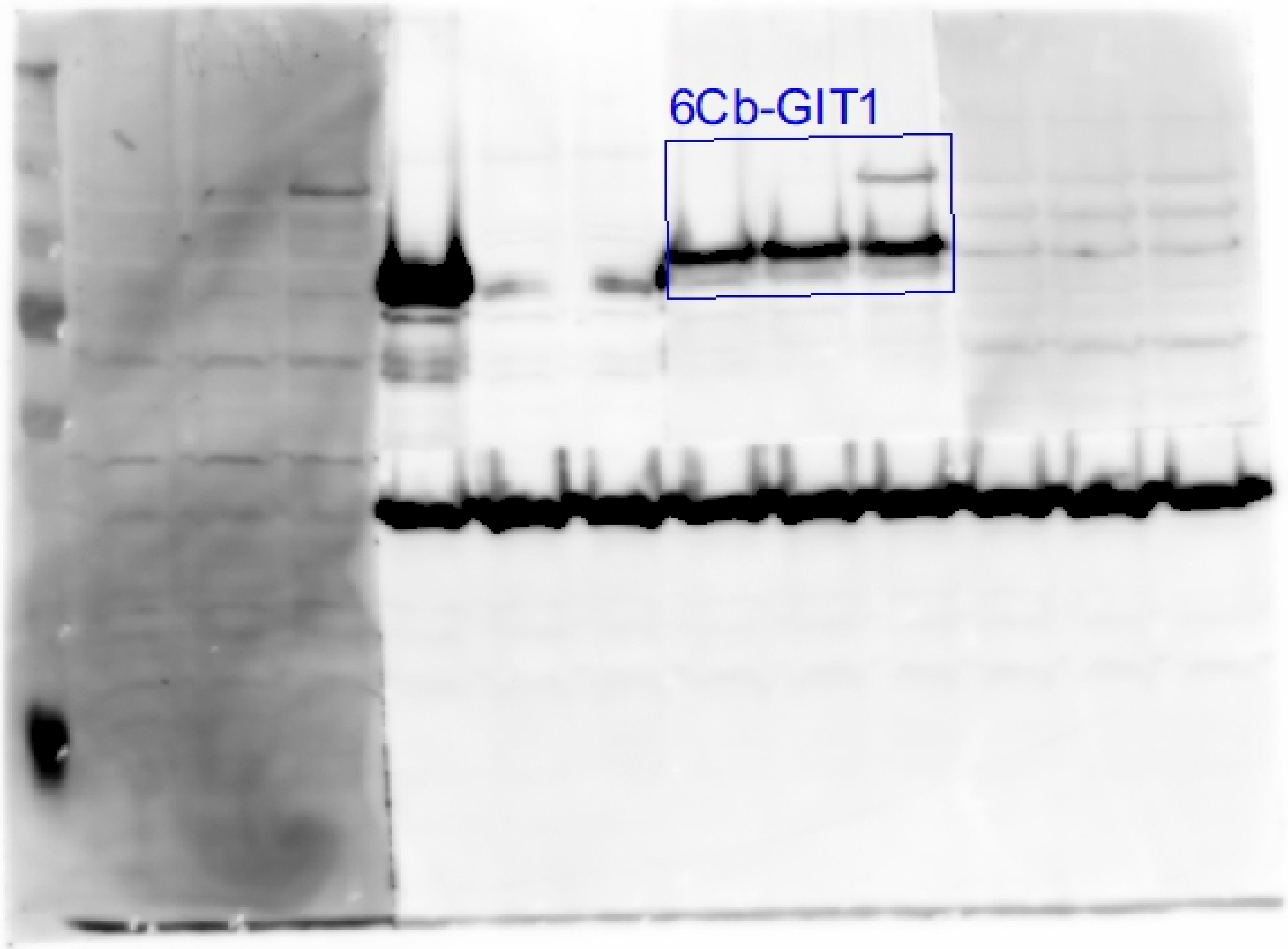

6Cb-GIT1

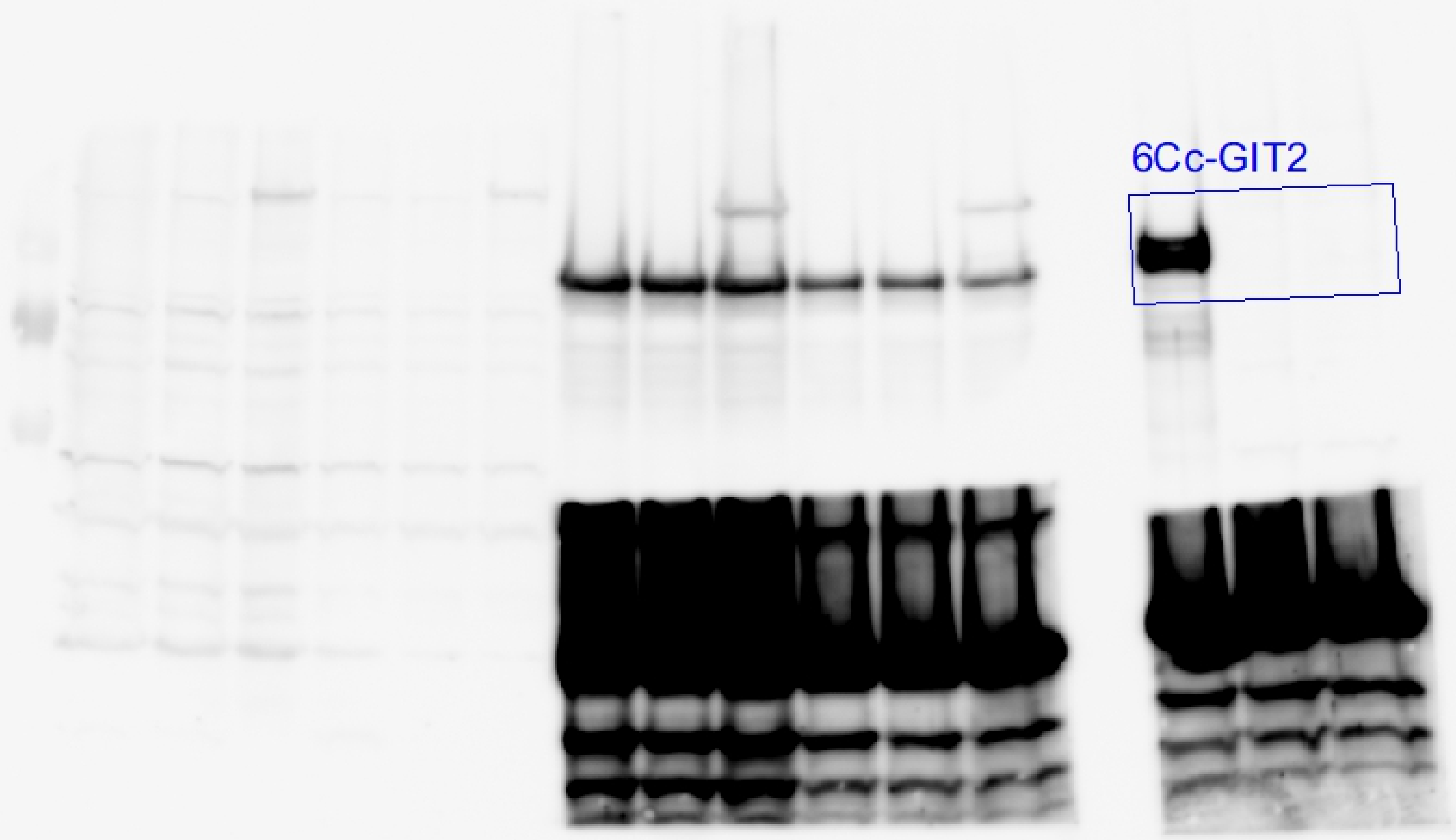
6Cc-GIT2

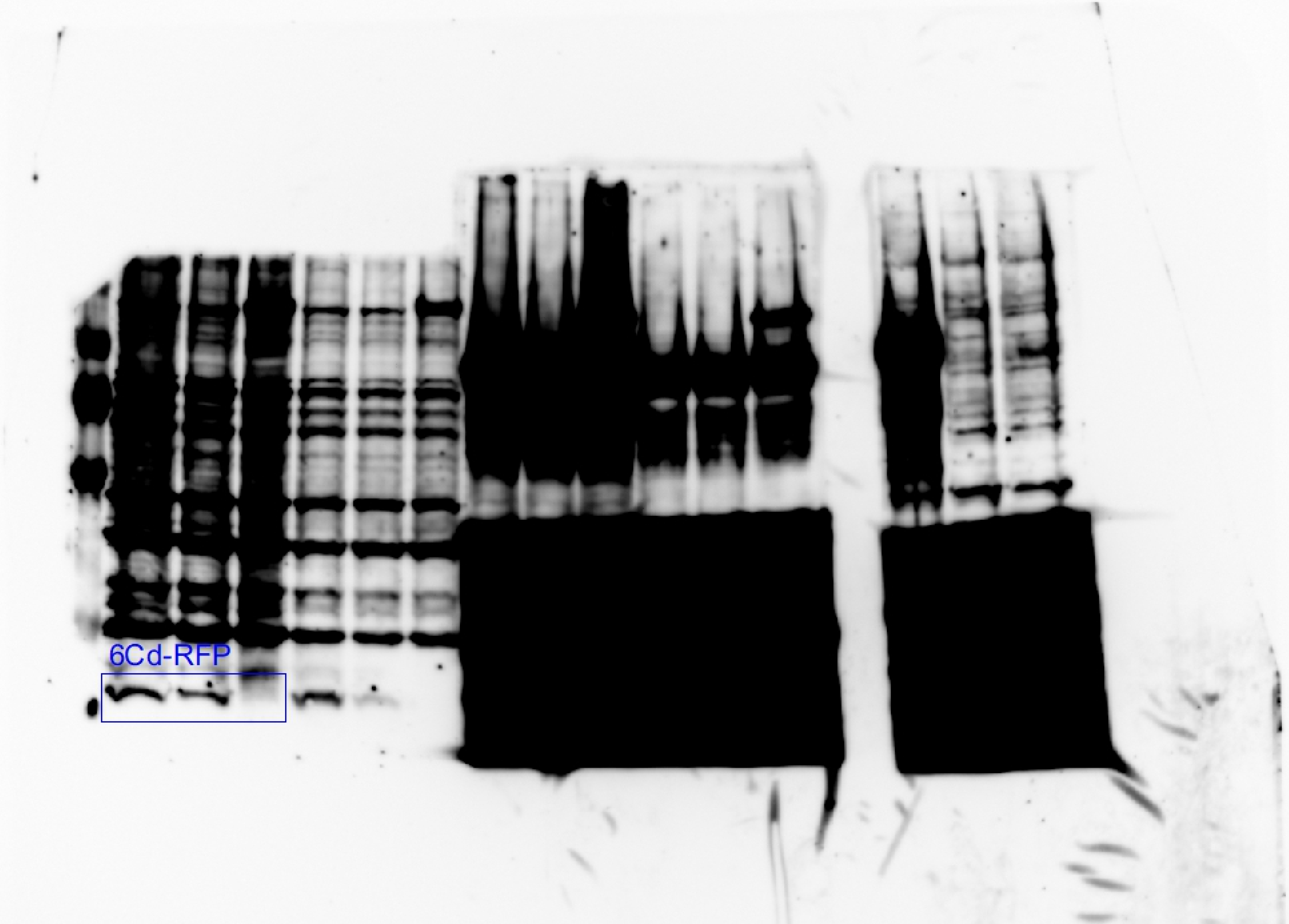
6Cd-RFP

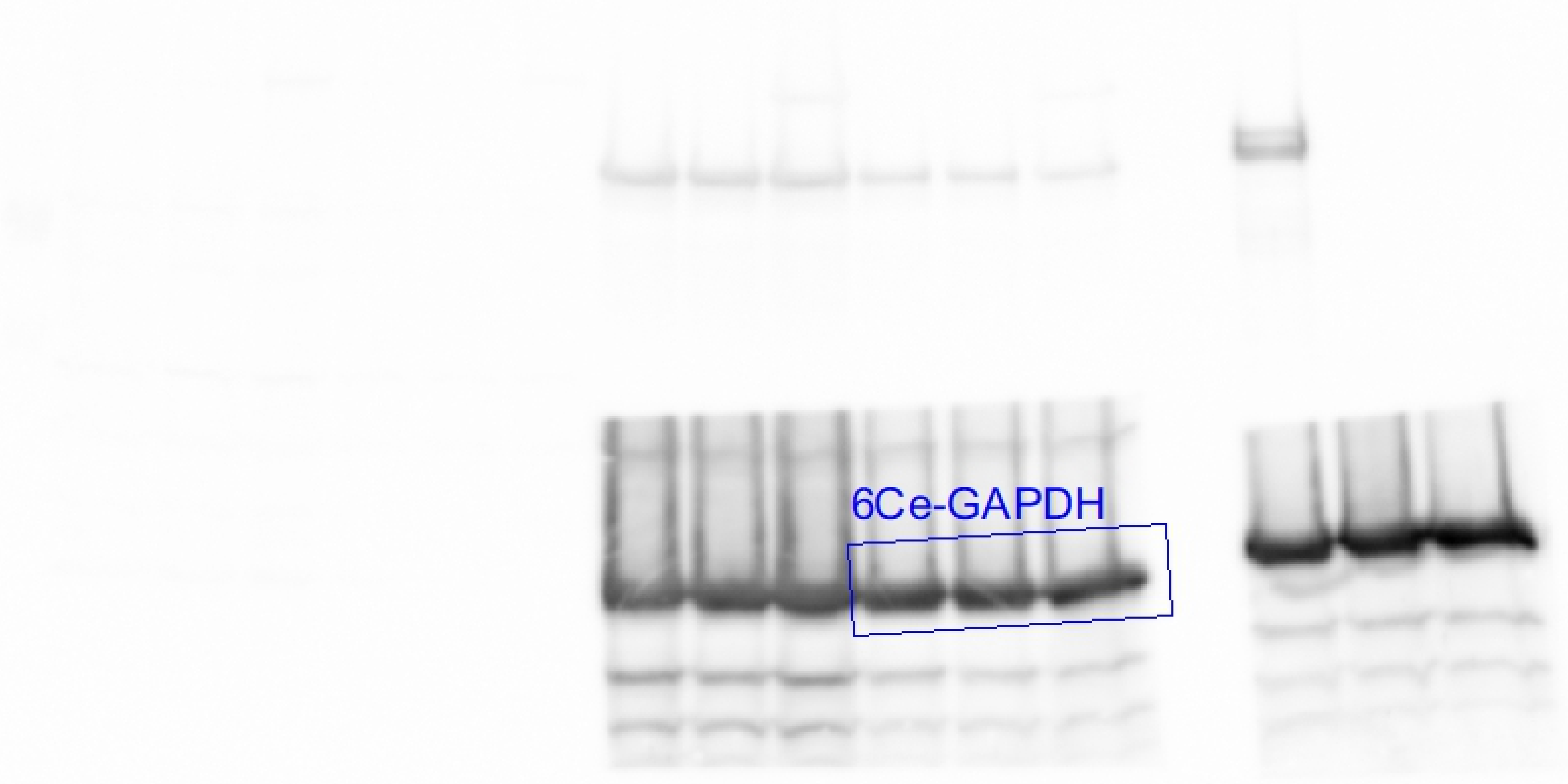
6Ce-GAPDH
