# Supplementary material for: Regulation of microtubule nucleation in mouse bone marrow-derived mast cells by ARF GTPase-activating protein GIT2
Source: Front Immunol. 2024 Feb 2;15:1321321. doi: 10.3389/fimmu.2024.1321321 (PMC10870779; doi:10.3389/fimmu.2024.1321321)
Supplement: Supplementary file 2 [file Figure 7-originals.pdf › Data Sheet 7/Figure 7-originals.pdf]

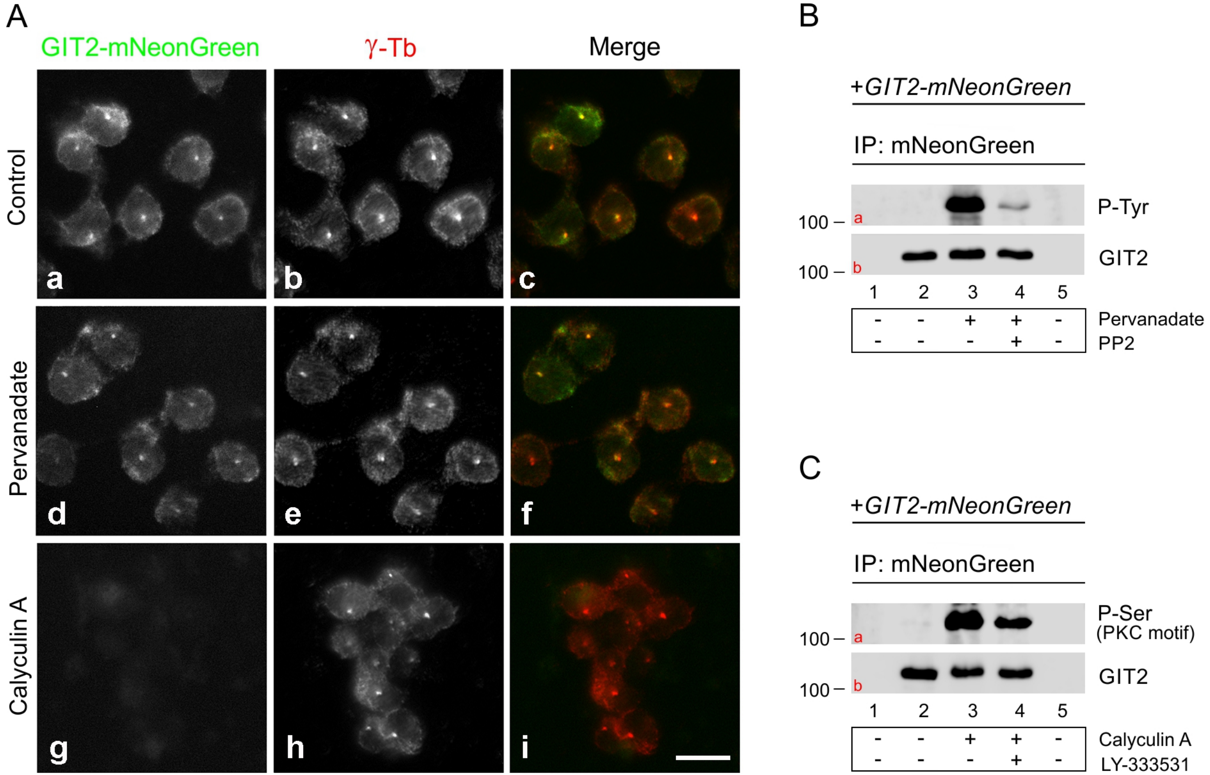

A
GIT2-mNeonGreen
γ-Tb
Merge
Control
Pervanadate
Calyculin A
a
b
c
d
e
f
g
h
i
B
+GIT2-mNeonGreen
IP: mNeonGreen
P-Tyr
GIT2
100 –
100 –
1 2 3 4 5
Pervanadate - - + + -
PP2 - - - + -
C
+GIT2-mNeonGreen
IP: mNeonGreen
P-Ser
(PKC motif)
GIT2
100 –
100 –
1 2 3 4 5
Calyculin A - - + + -
LY-333531 - - - + -

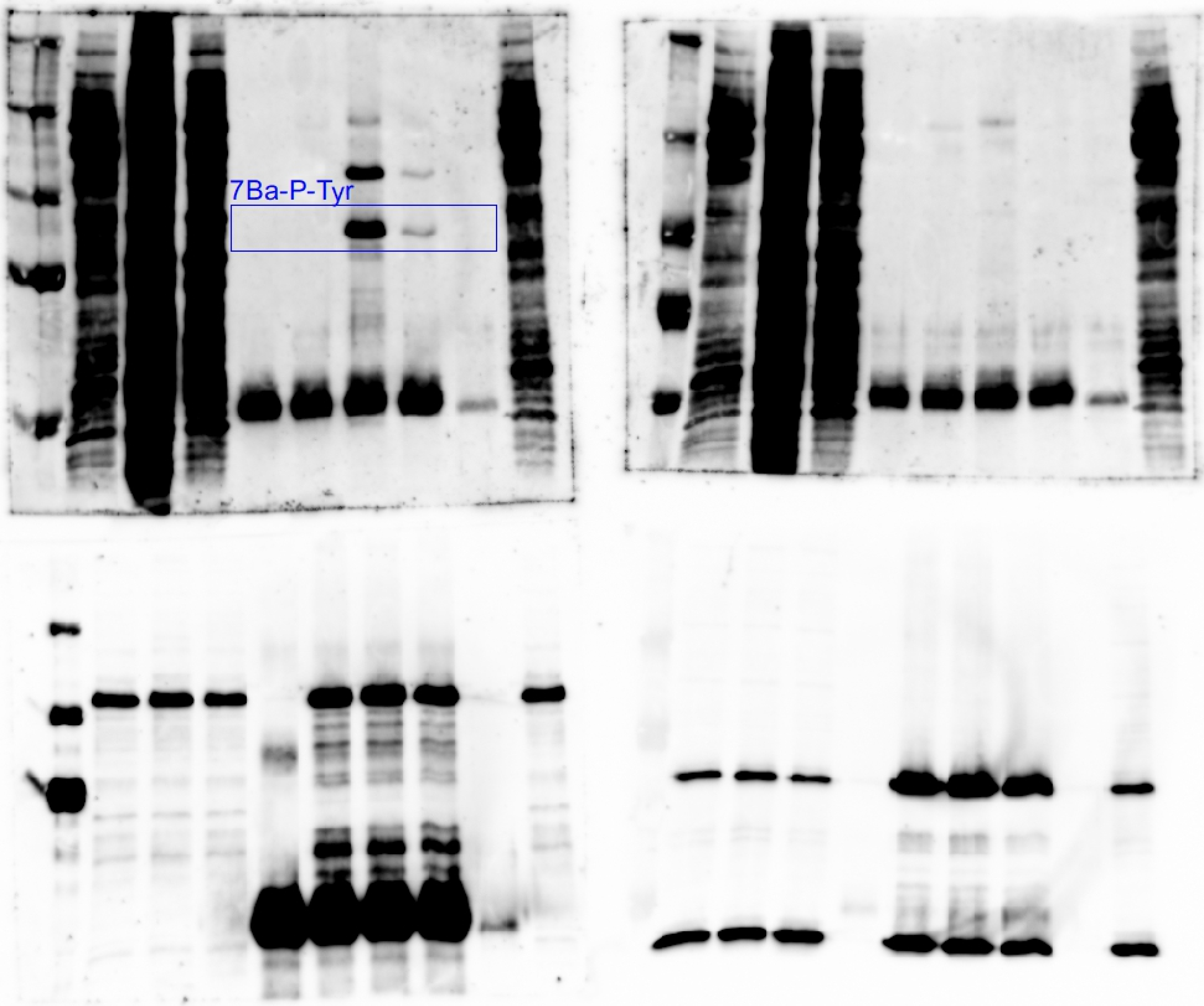
7Ba-P-Tyr

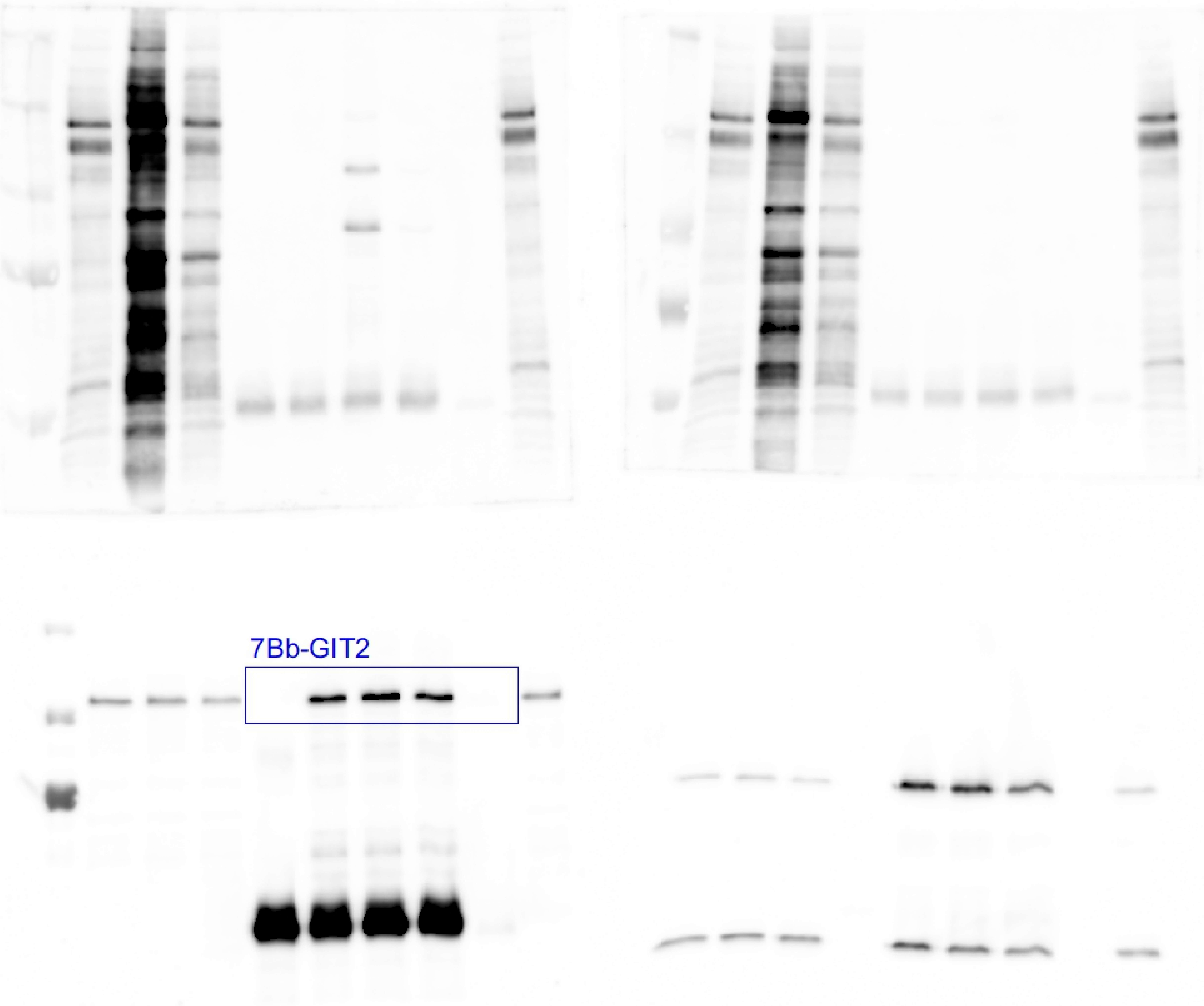
7Bb-GIT2

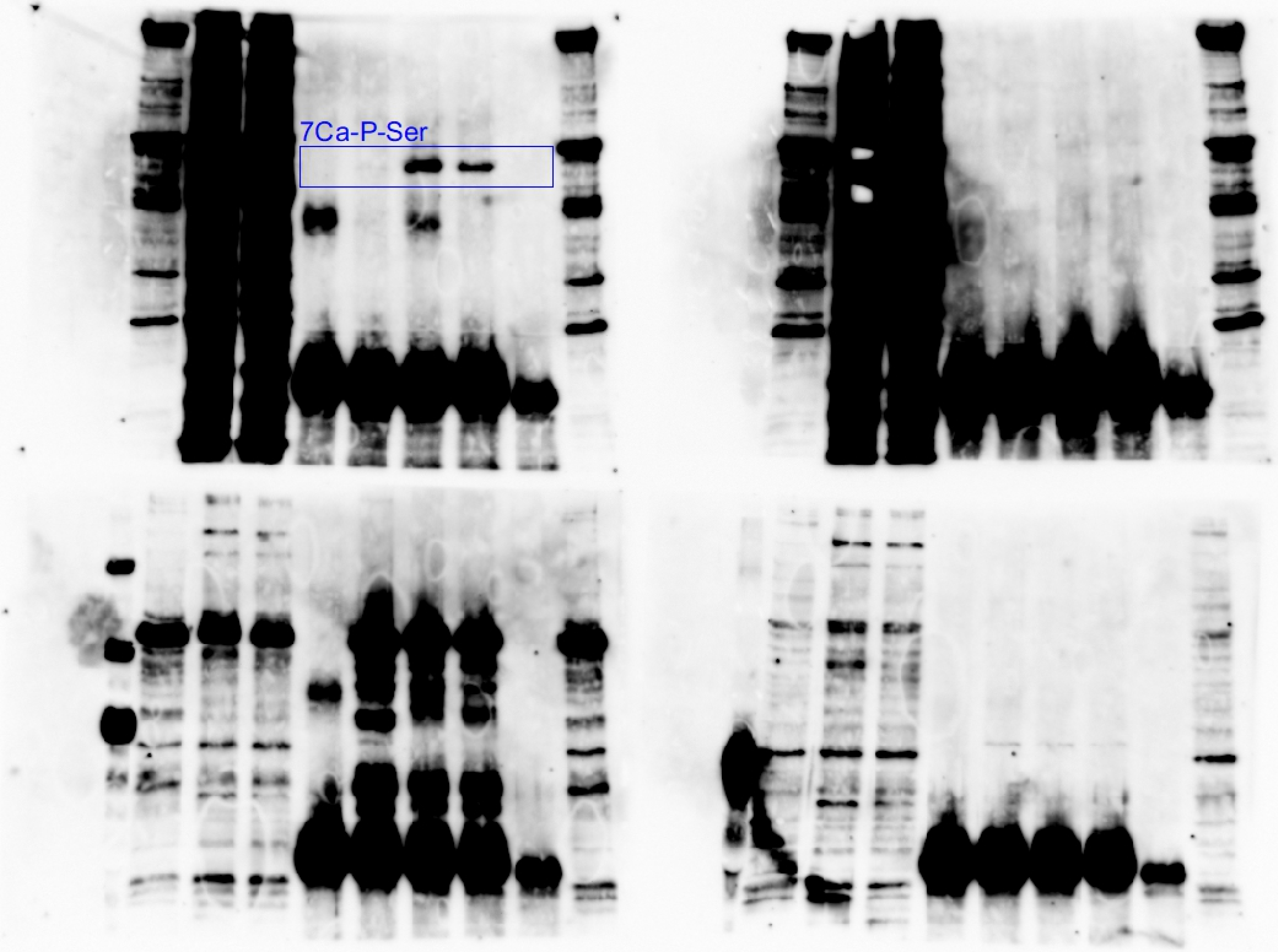
7Ca-P-Ser

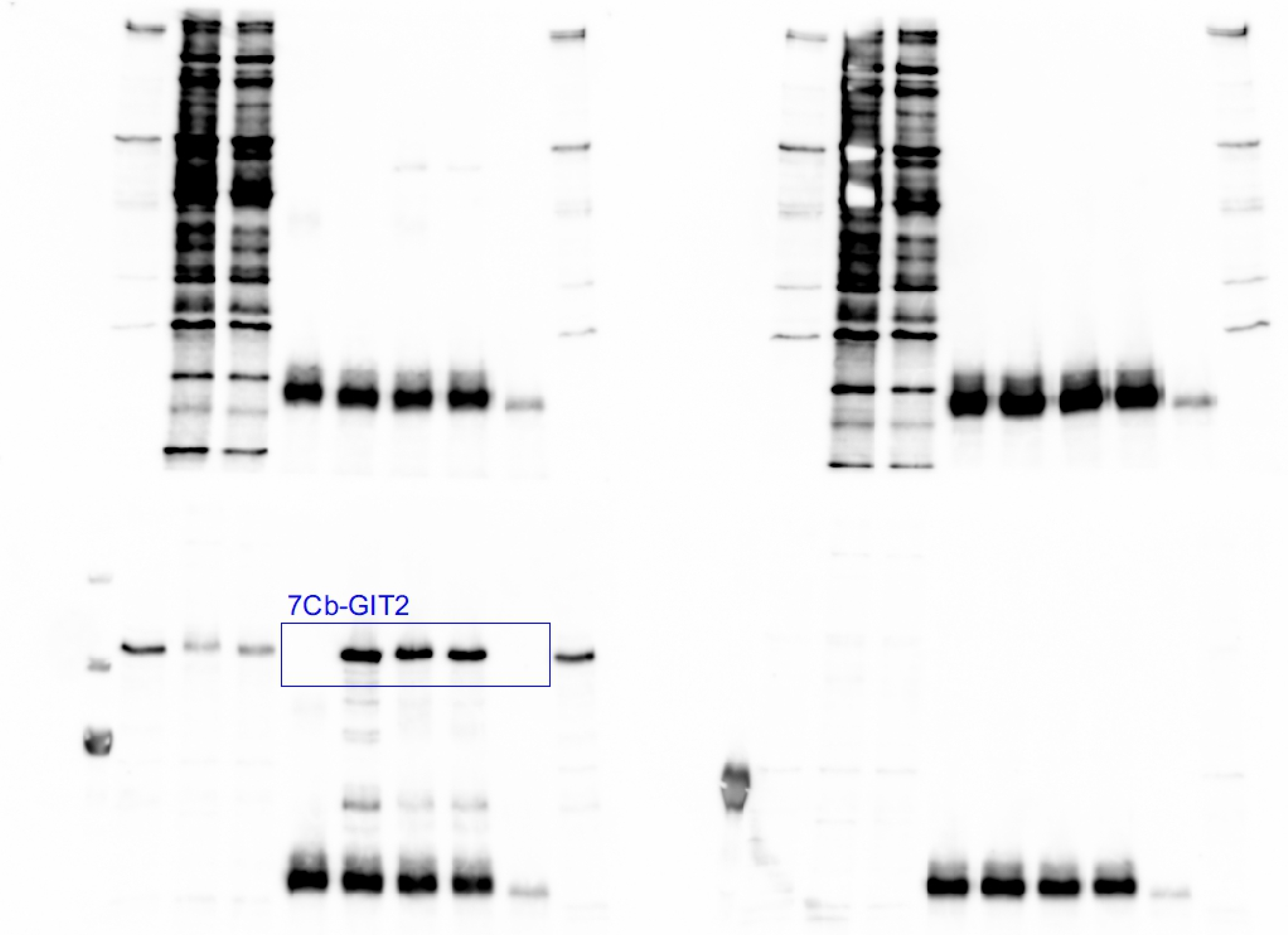
7Cb-GIT2
