# Supplementary material for: Regulation of microtubule nucleation in mouse bone marrow-derived mast cells by ARF GTPase-activating protein GIT2
Source: Front Immunol. 2024 Feb 2;15:1321321. doi: 10.3389/fimmu.2024.1321321 (PMC10870779; doi:10.3389/fimmu.2024.1321321)
Supplement: Supplementary file 2 [file Figure 8-originals.pdf › Data Sheet 7/Figure 8-originals.pdf]

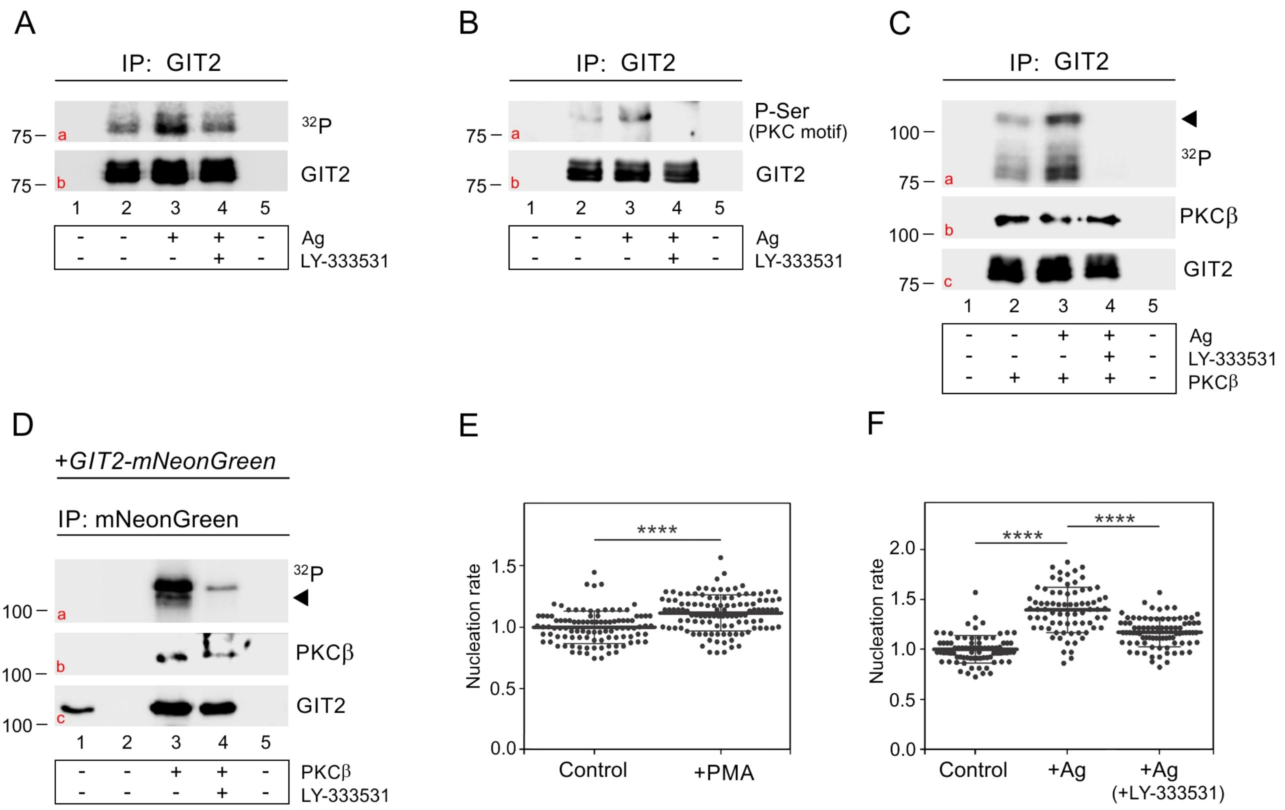

A
IP: GIT2
32P
GIT2
75
a
b
1 2 3 4 5
Ag
LY-333531
B
IP: GIT2
P-Ser
(PKC motif)
GIT2
C
IP: GIT2
100
32P
PKCβ
GIT2
c
D
+GIT2-mNeonGreen
IP: mNeonGreen
E
Nucleation rate
****
Control
+PMA
F
Control
+Ag
+Ag
(+LY-333531)

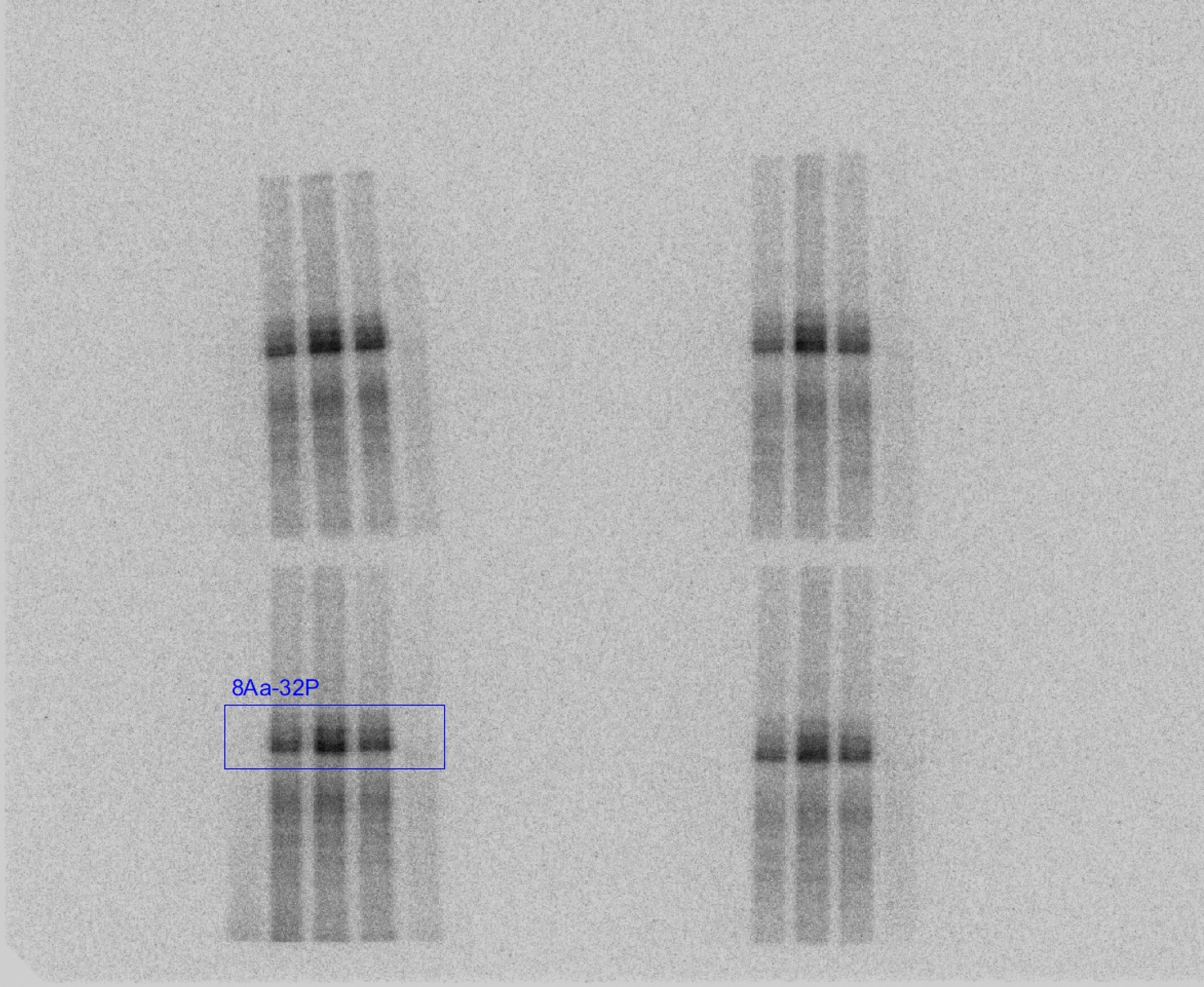
8Aa-32P

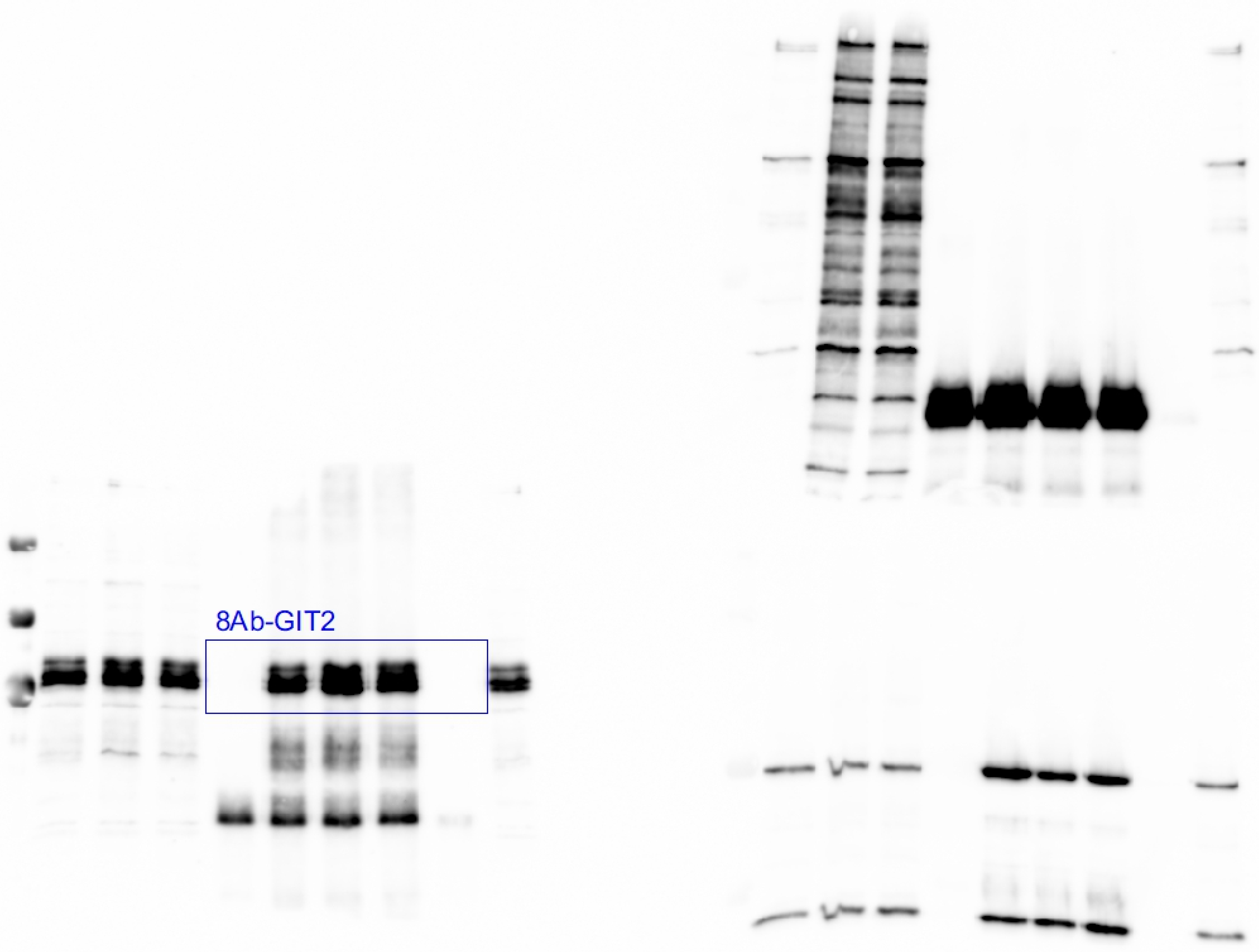
8Ab-GIT2

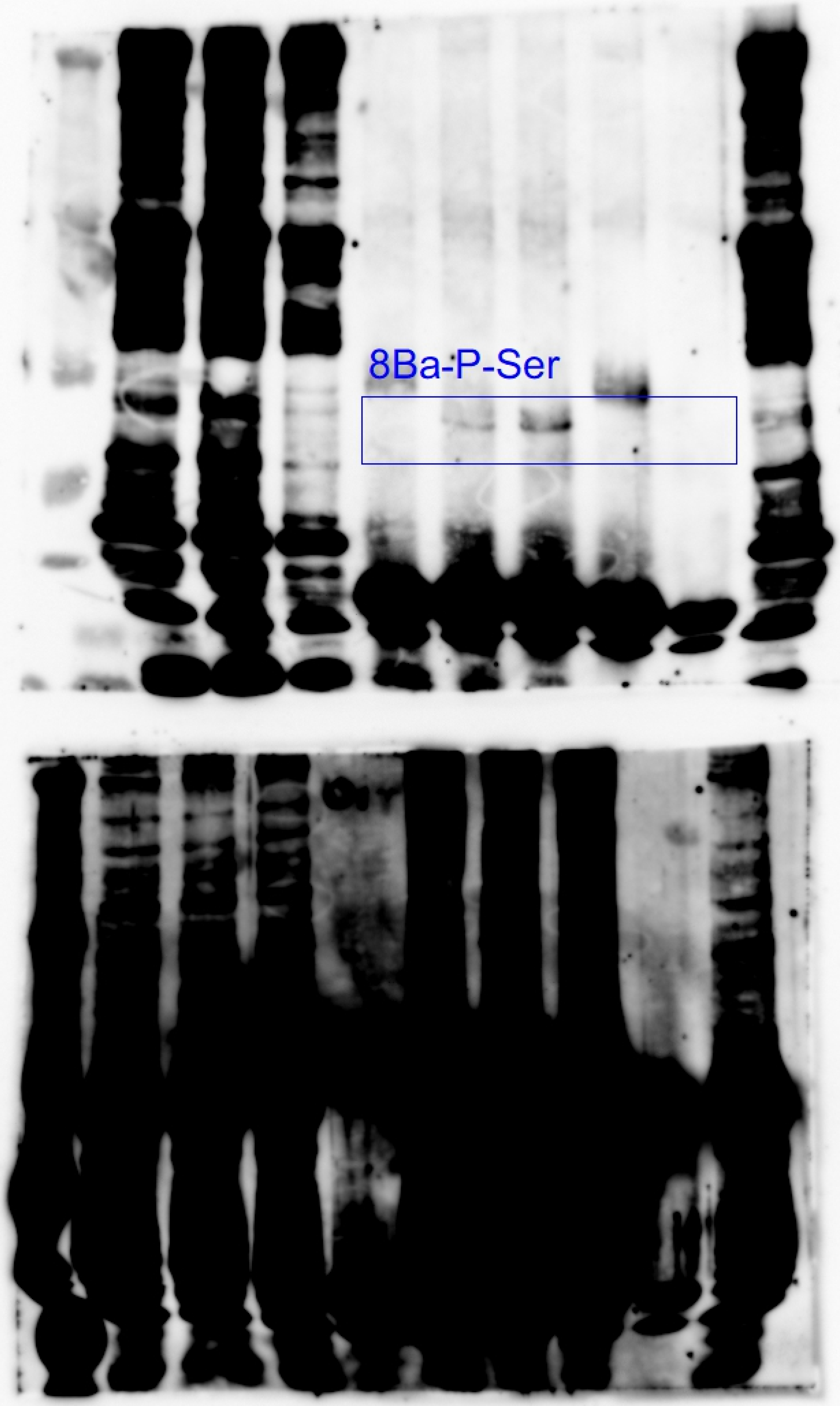
8Ba-P-Ser

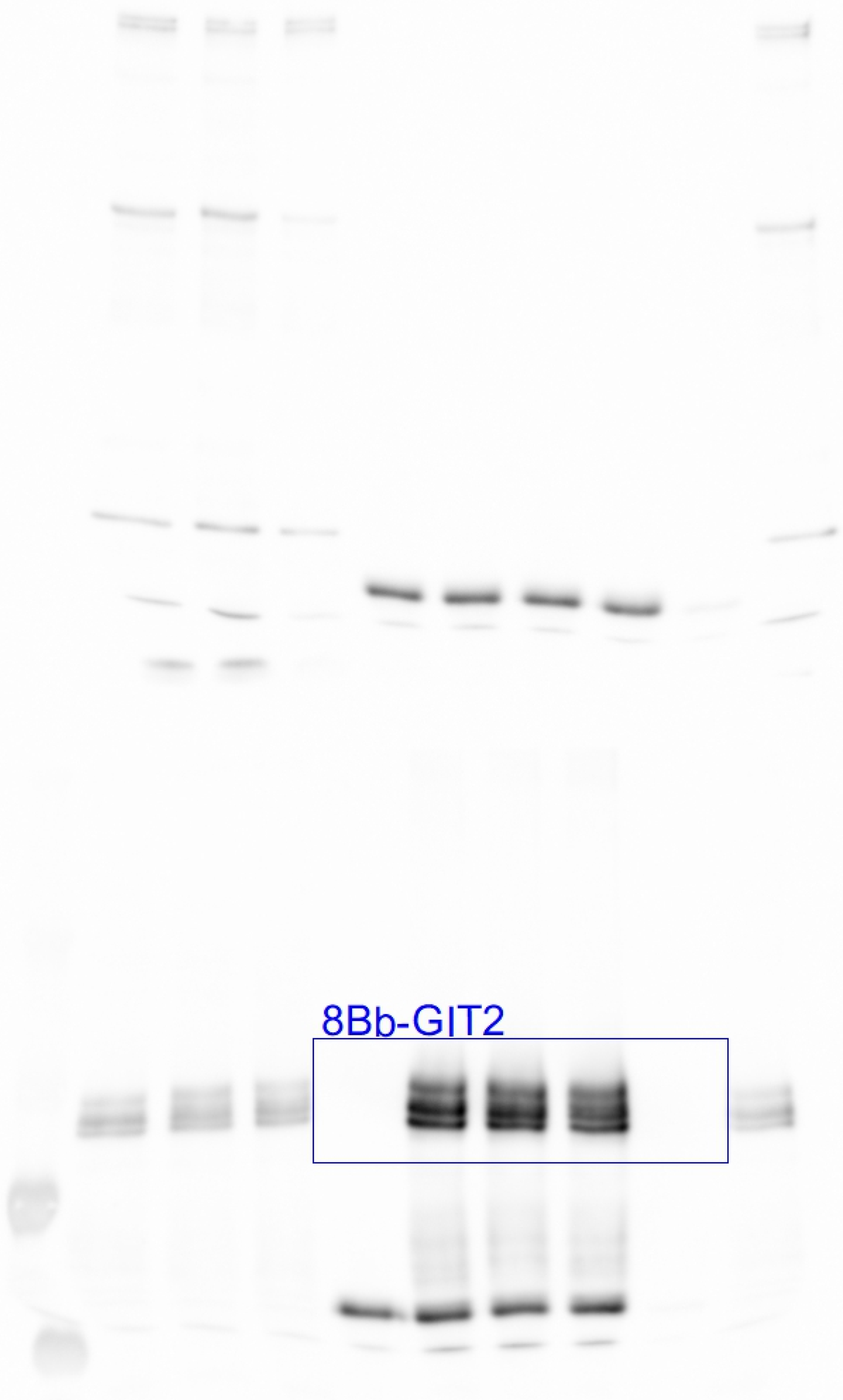
8Bb-GIT2

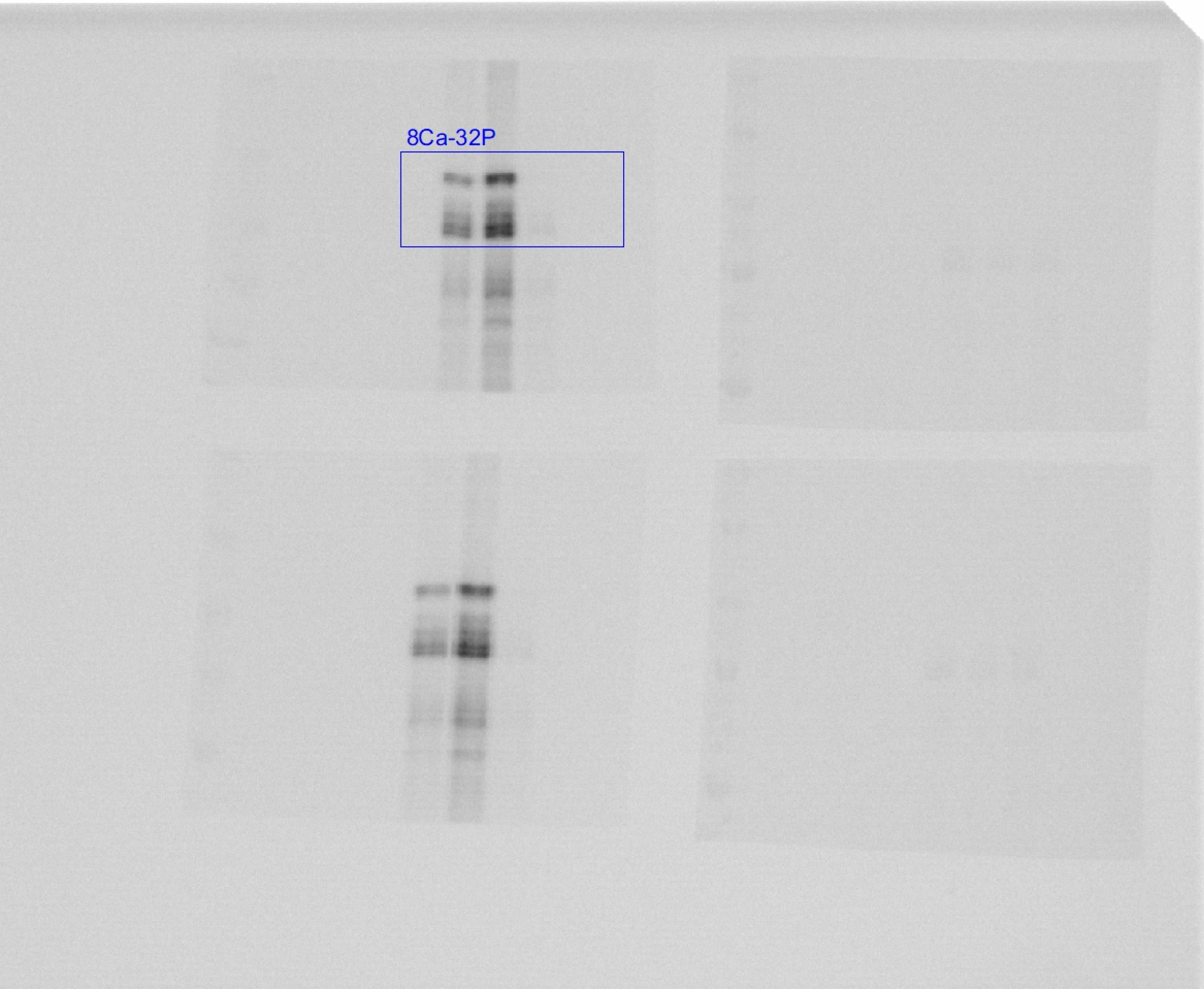
8Ca-32P

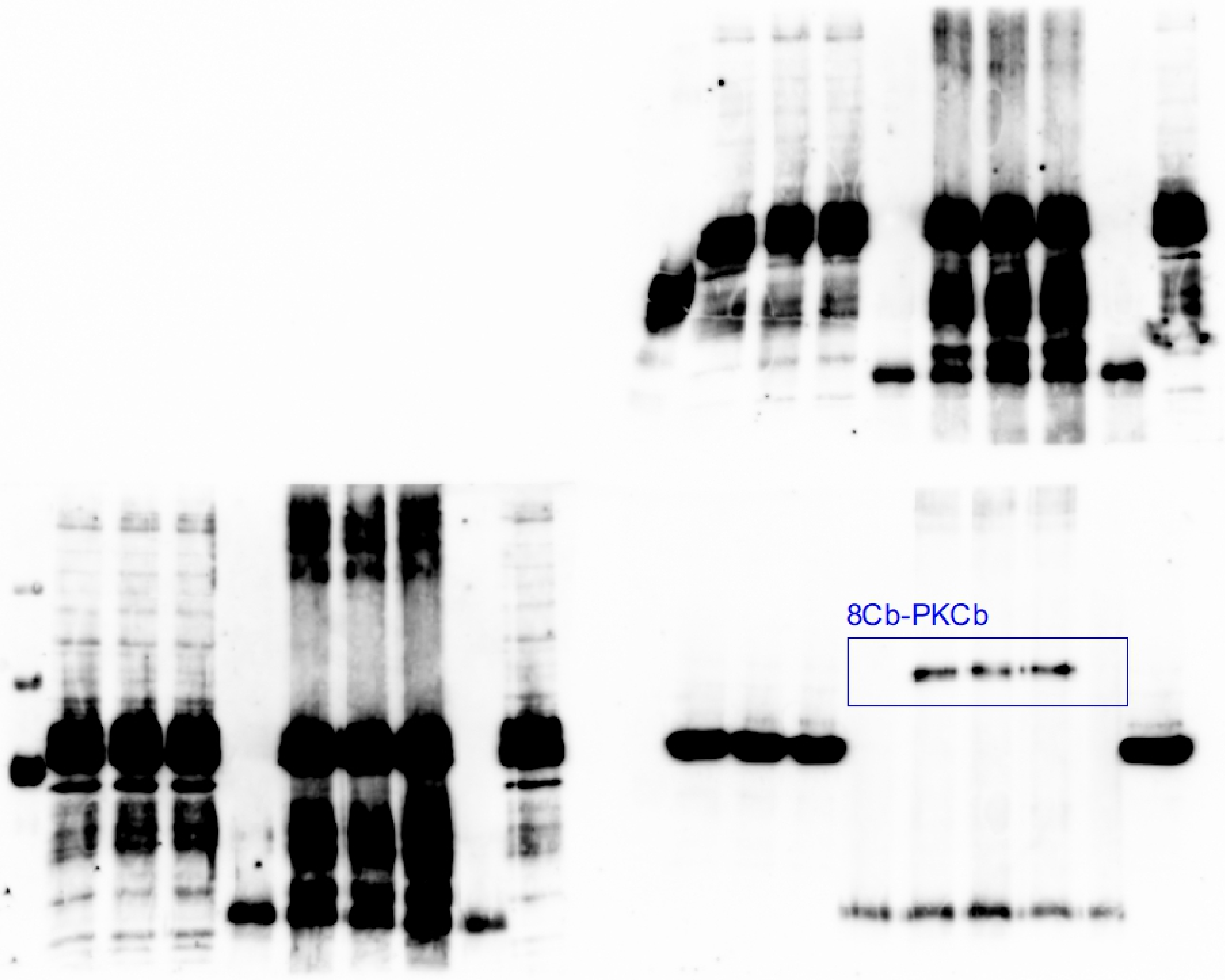
8Cb-PKCb

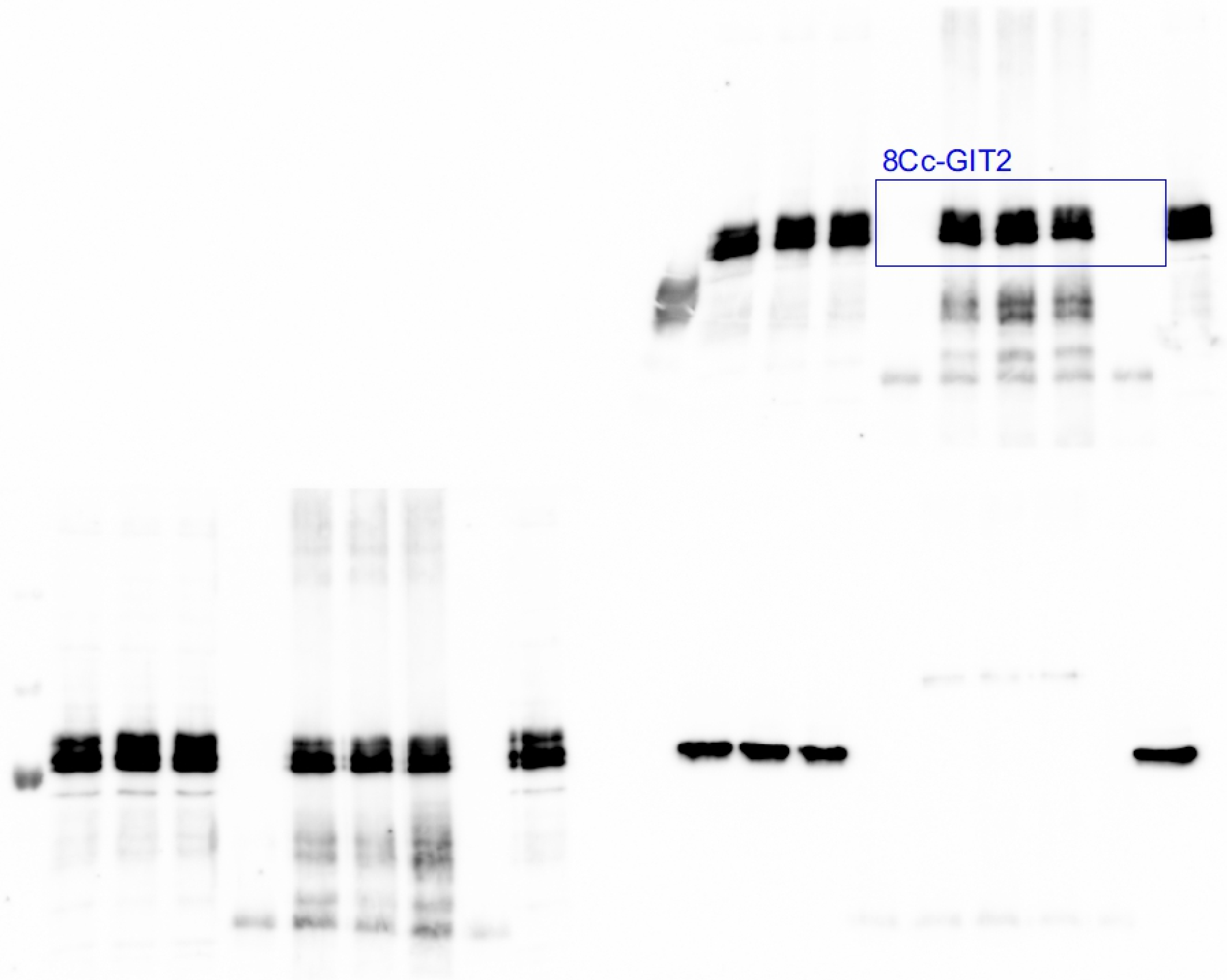
8Cc-GIT2

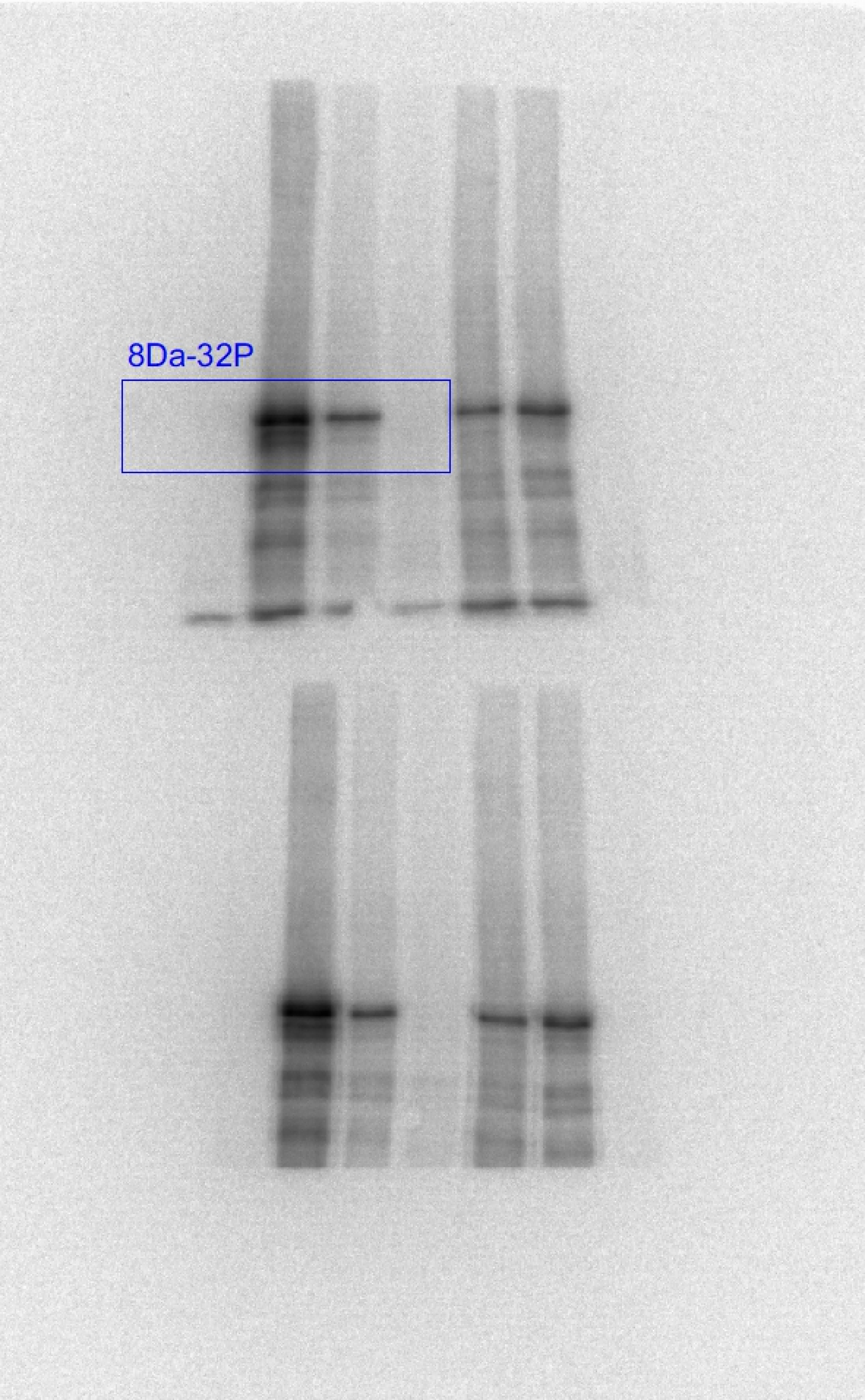
8Da-32P

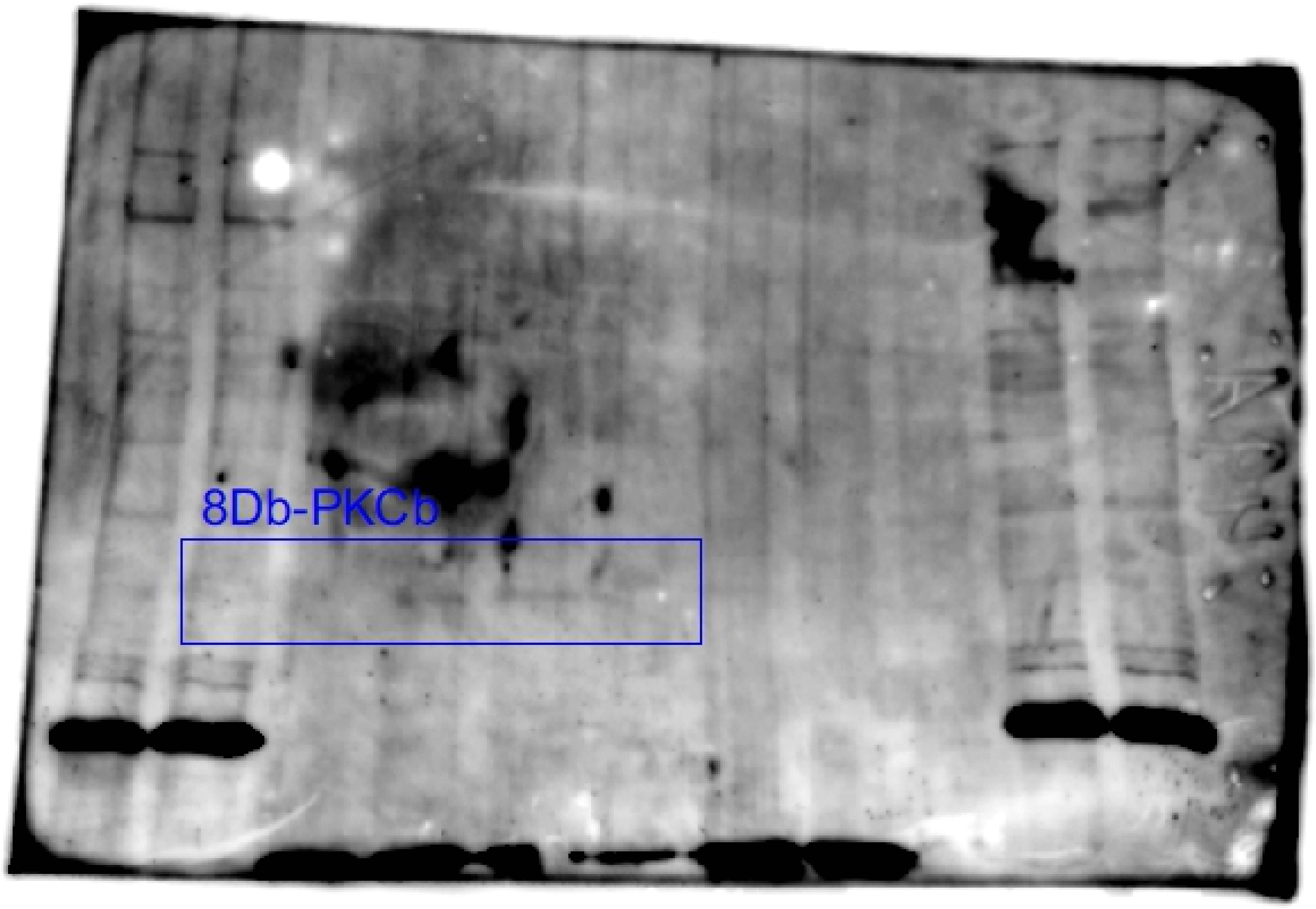
8Db-PKCb

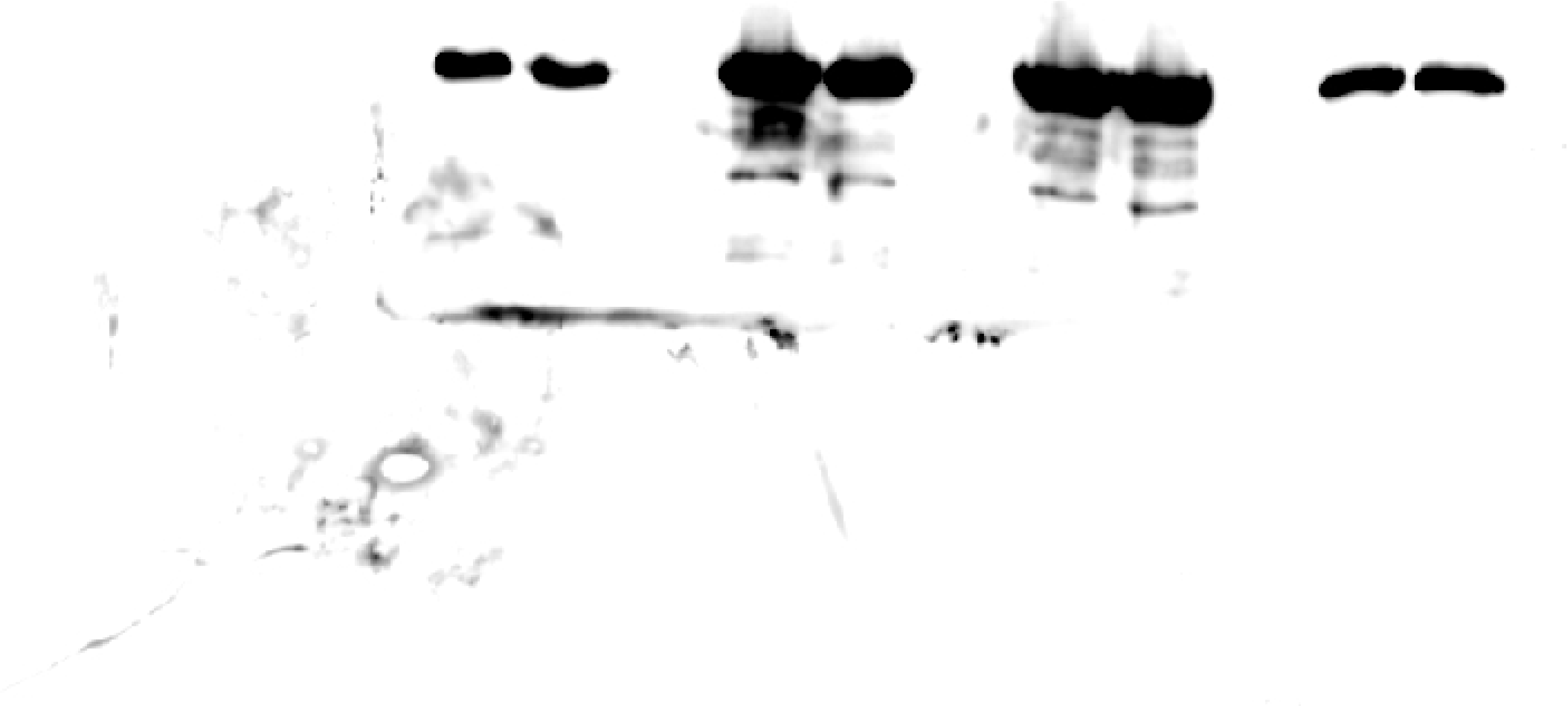

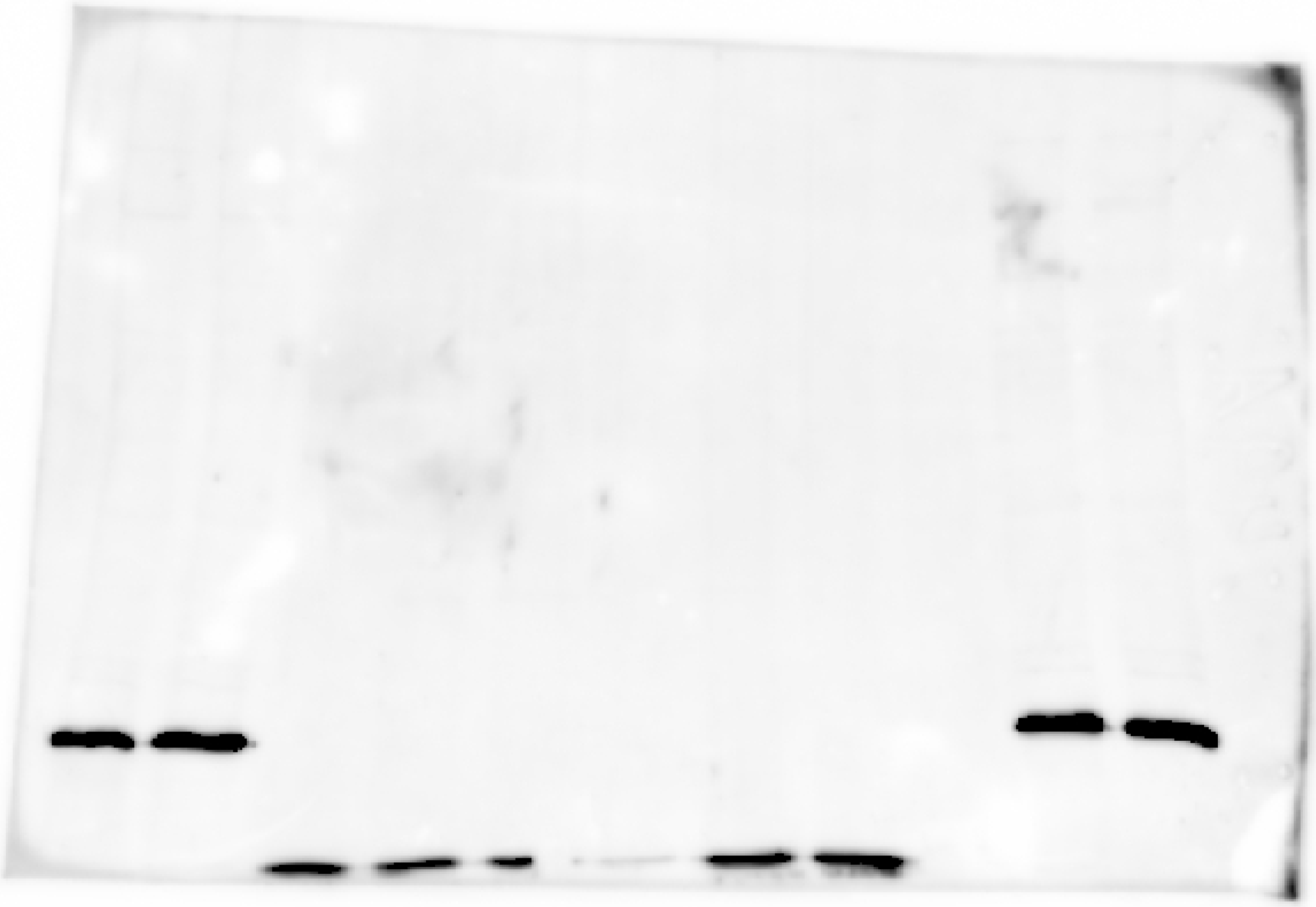

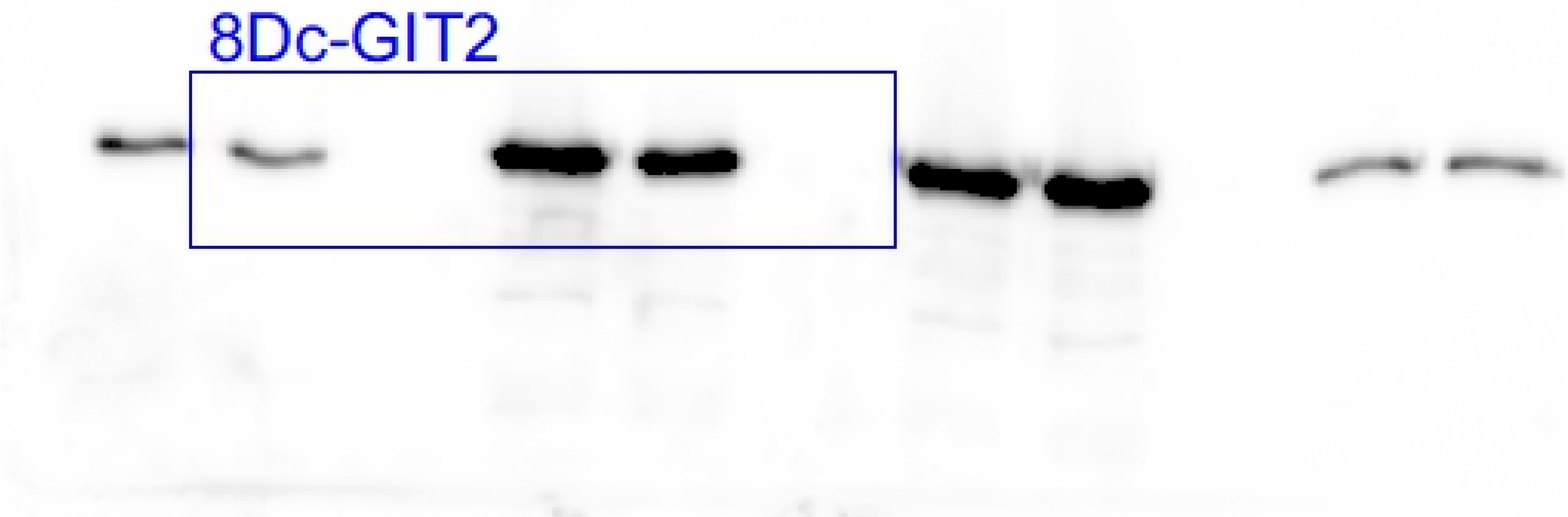
8Dc-GIT2
