# Supplementary material for: Regulation of microtubule nucleation in mouse bone marrow-derived mast cells by ARF GTPase-activating protein GIT2
Source: Front Immunol. 2024 Feb 2;15:1321321. doi: 10.3389/fimmu.2024.1321321 (PMC10870779; doi:10.3389/fimmu.2024.1321321)

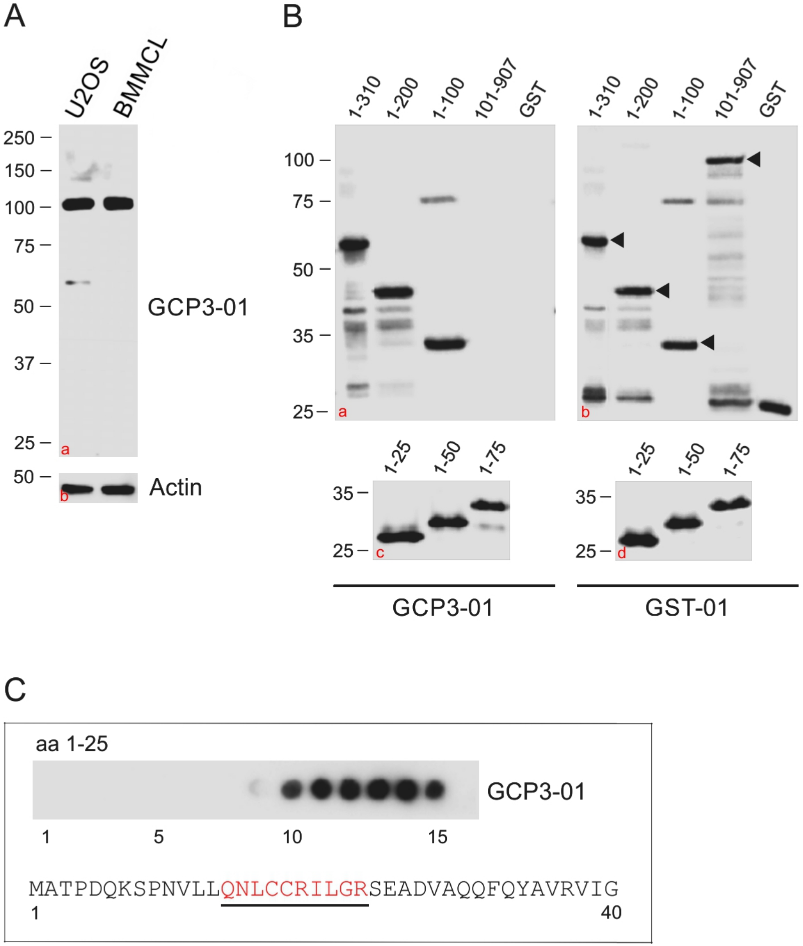
A
U2OS
BMMCL
250
150
100
75
50
37
25
GCP3-01
50
Actin
B
1-310
1-200
1-100
101-907
GST
100
75
50
35
25
1-25
1-50
1-75
35
25
GCP3-01
GST-01
C
aa 1-25
GCP3-01
1
5
10
15
MATPDQKSPNVLLQNLCCRILGRSEADVAQQFQYAVRVIG
1
40

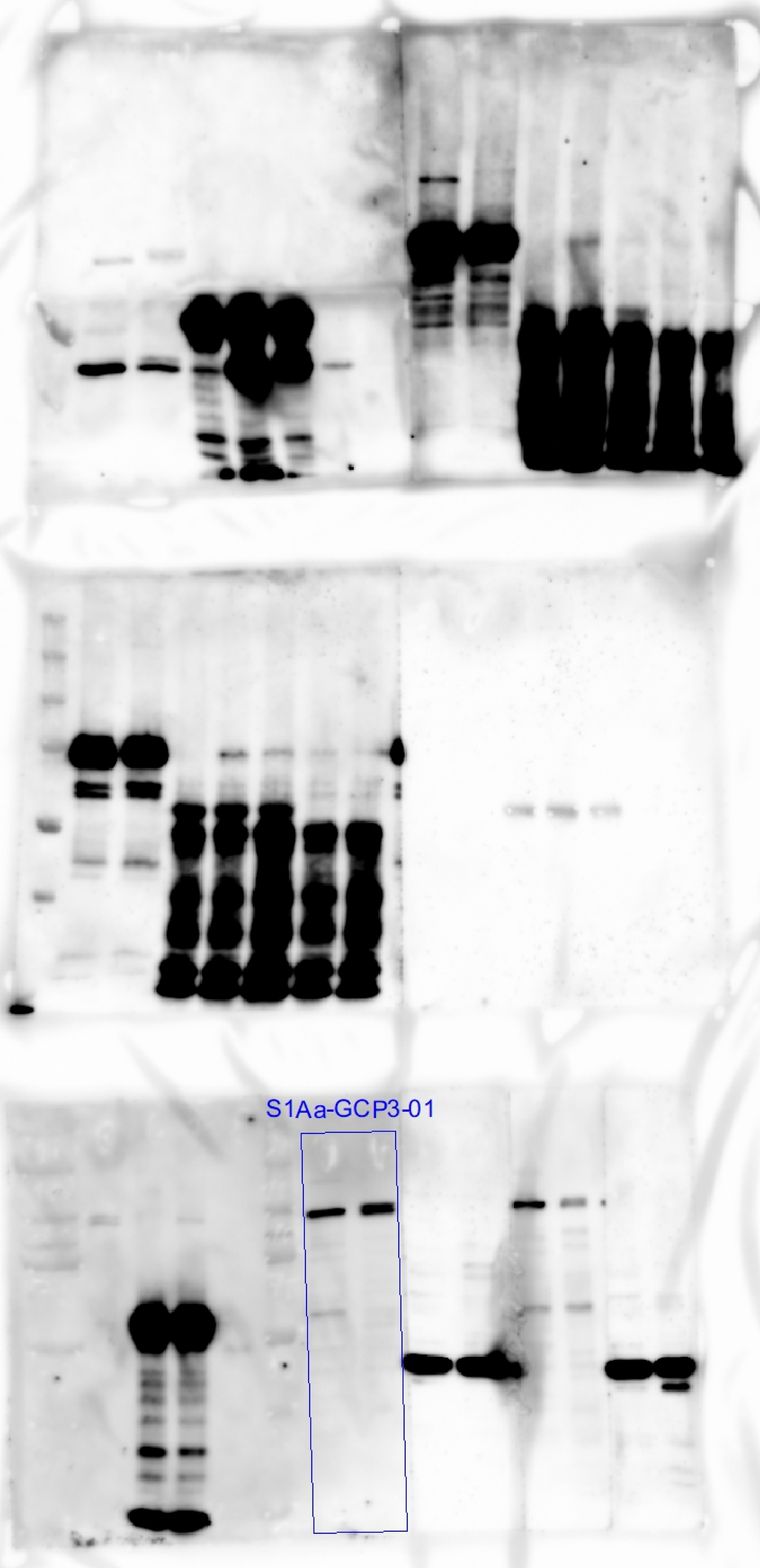
S1Aa-GCP3-01

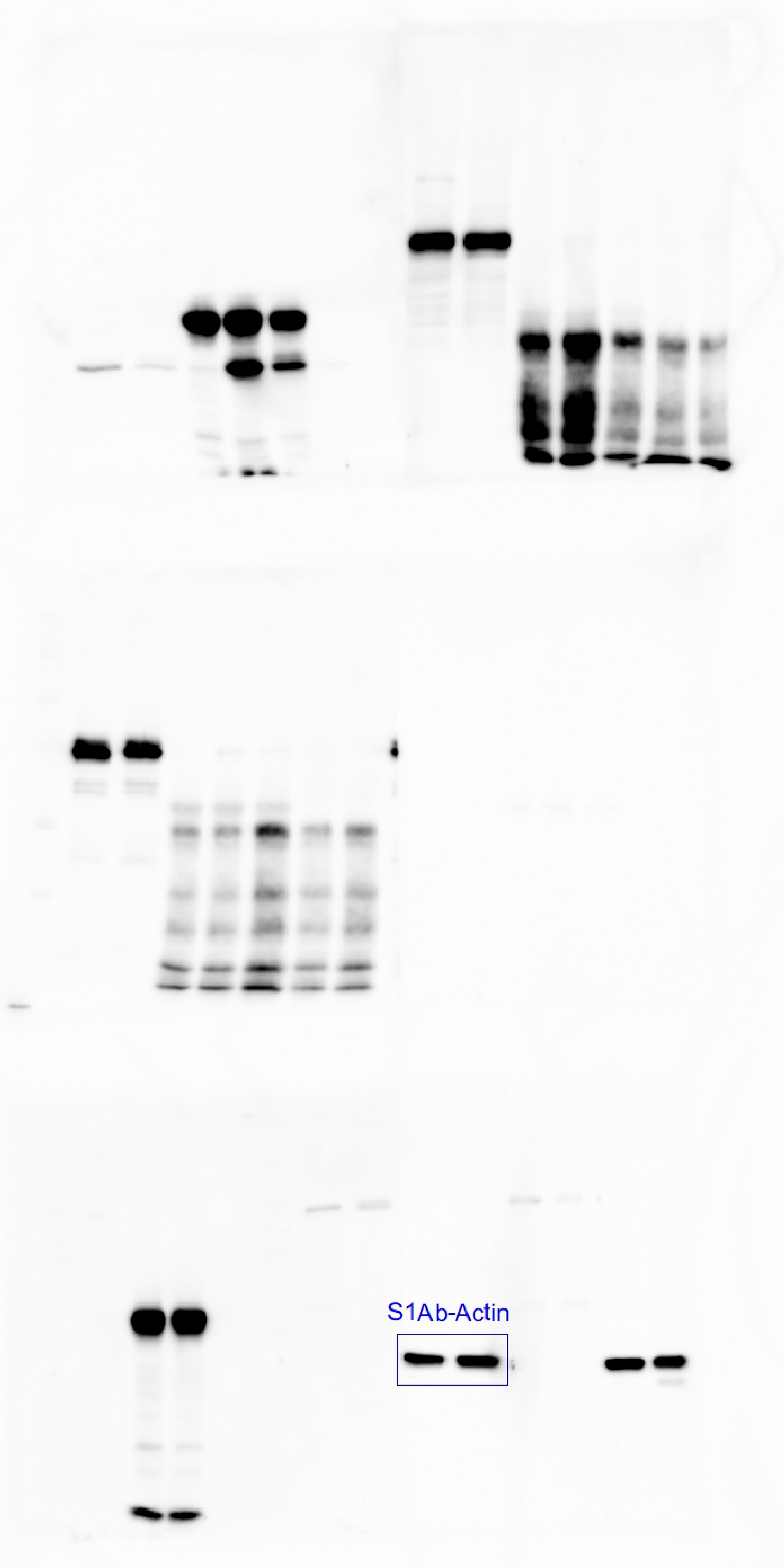
S1Ab-Actin

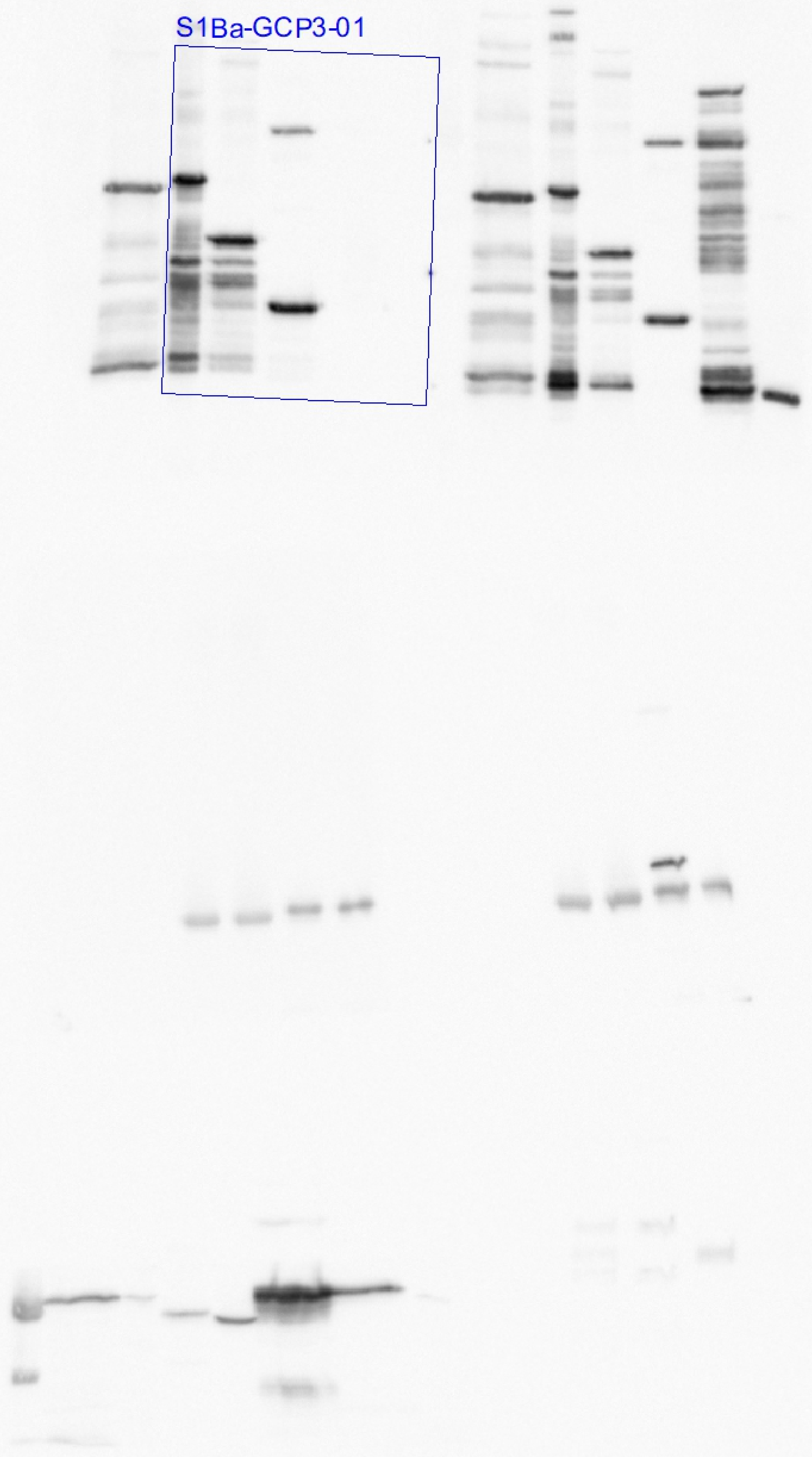
S1Ba-GCP3-01

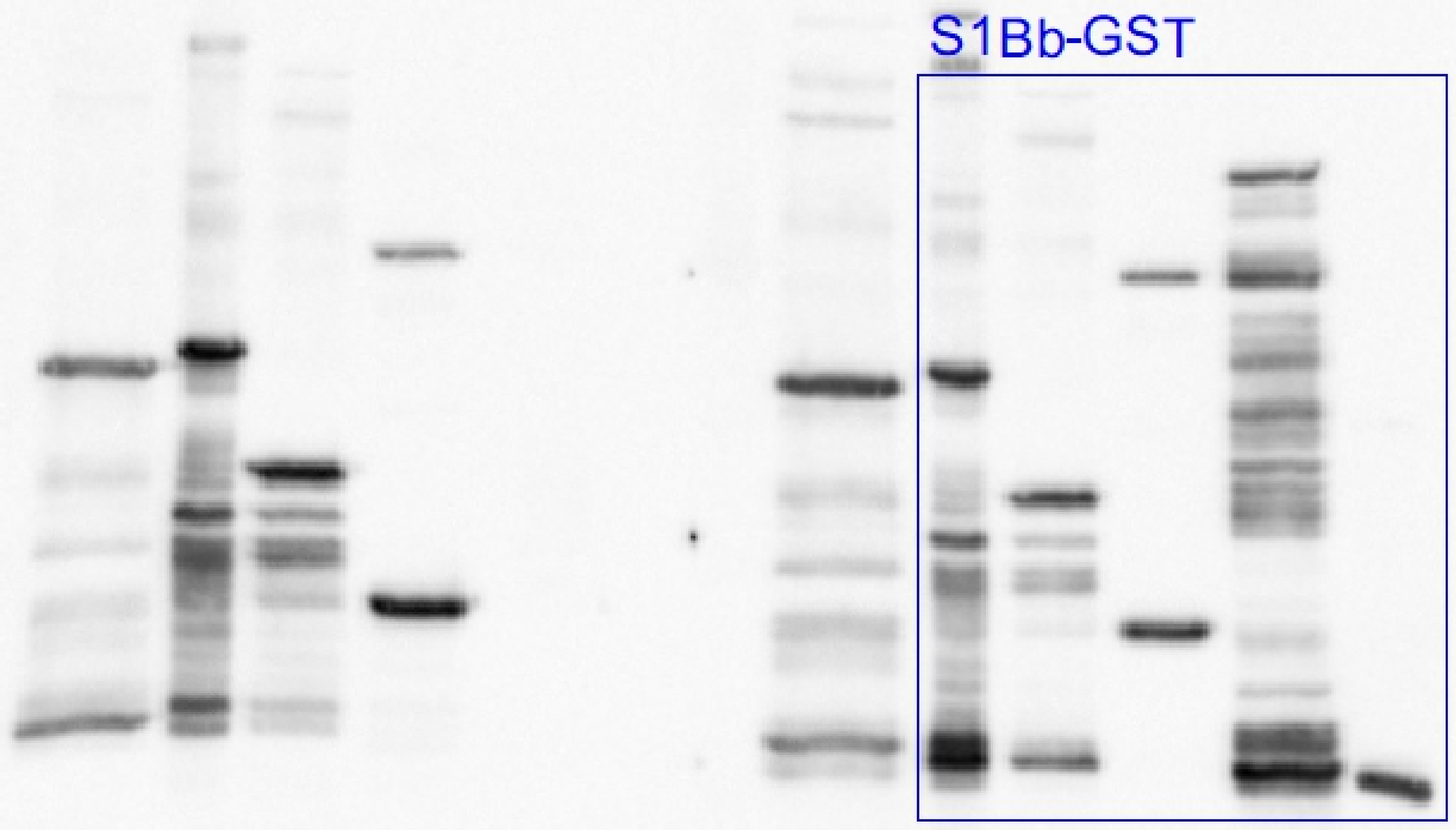
S1Bb-GST

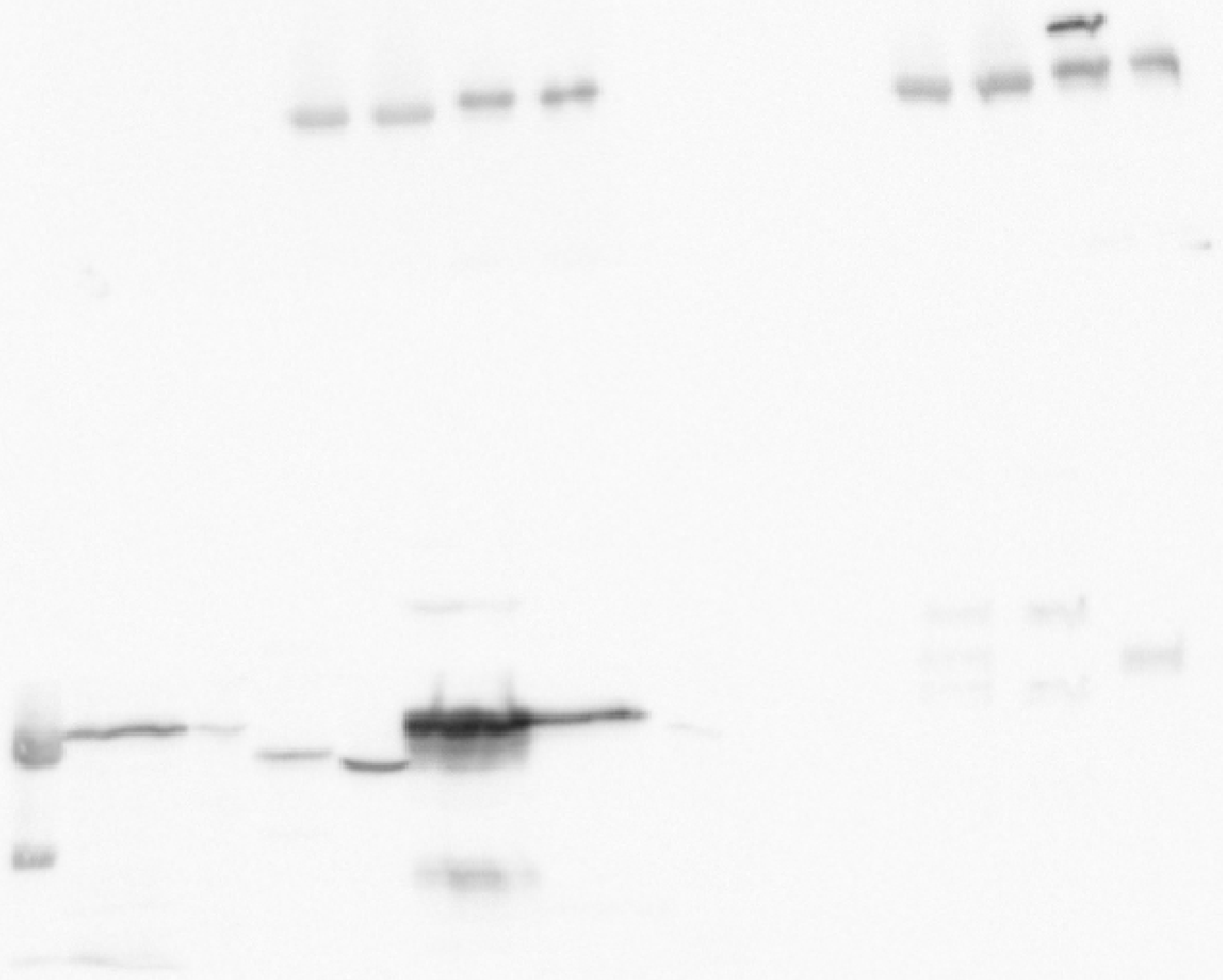

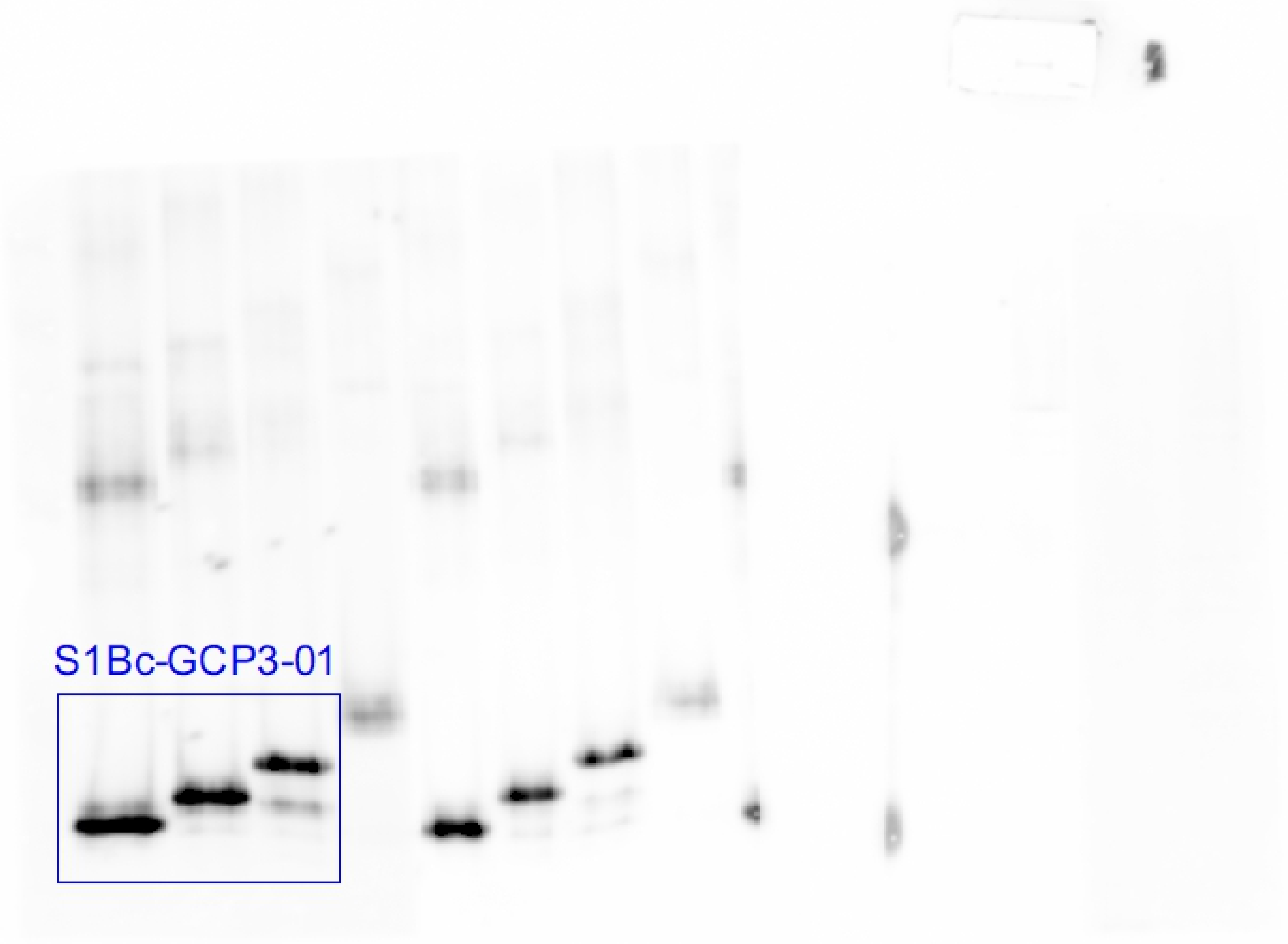
S1Bc-GCP3-01

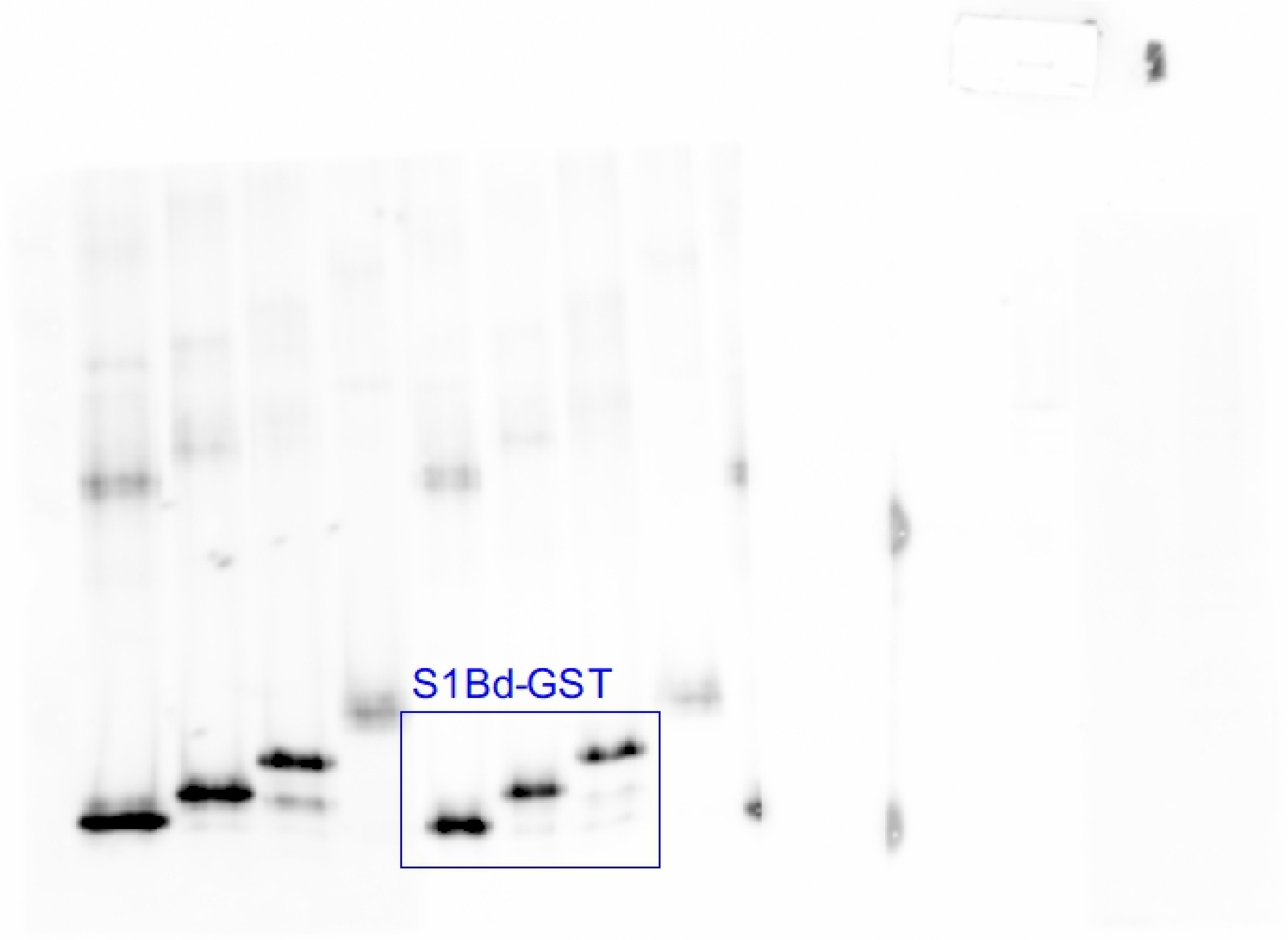
S1Bd-GST

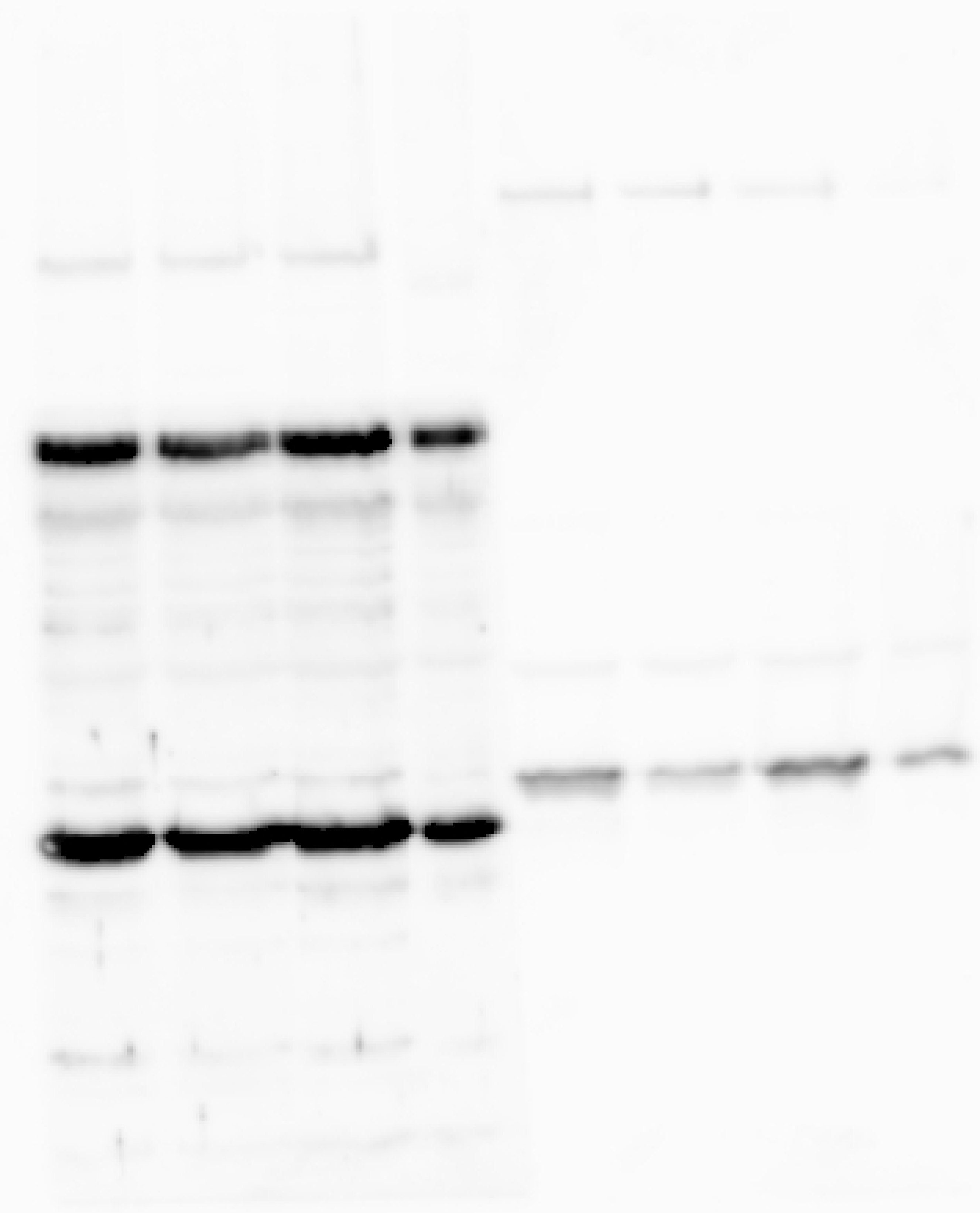

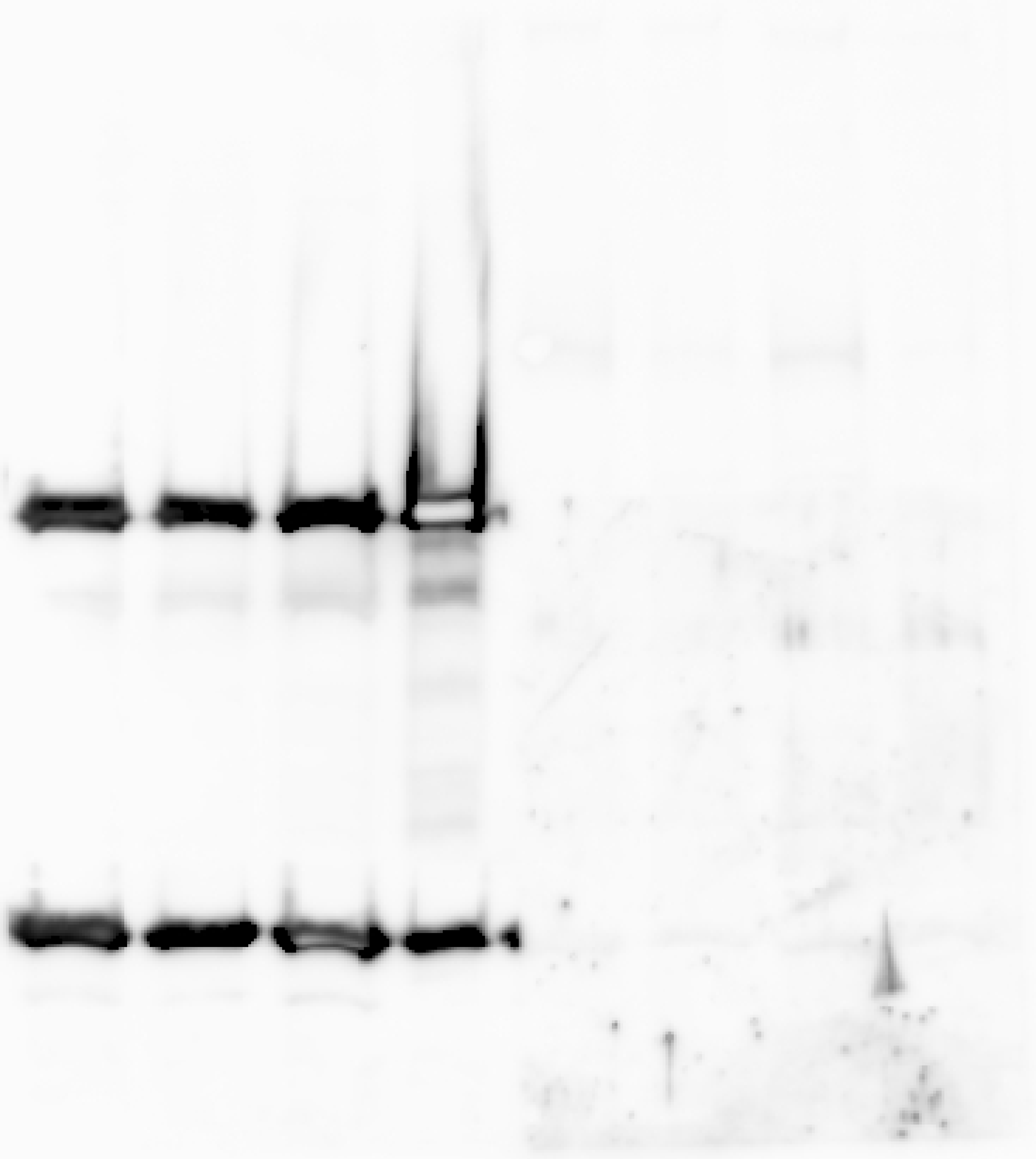

S1C-GCP3-01

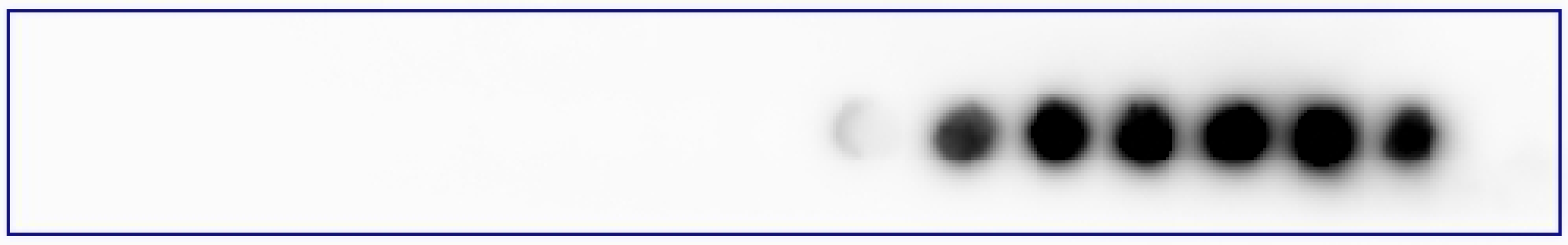

Supplement: Supplementary file 3 [file Figure S1-originals.pdf › Data Sheet 8/Figure S1-originals.pdf]
