# Supplementary material for: Regulation of microtubule nucleation in mouse bone marrow-derived mast cells by ARF GTPase-activating protein GIT2
Source: Front Immunol. 2024 Feb 2;15:1321321. doi: 10.3389/fimmu.2024.1321321 (PMC10870779; doi:10.3389/fimmu.2024.1321321)
Supplement: Supplementary file 3 [file Figure S2-originals.pdf › Data Sheet 8/Figure S2-originals.pdf]

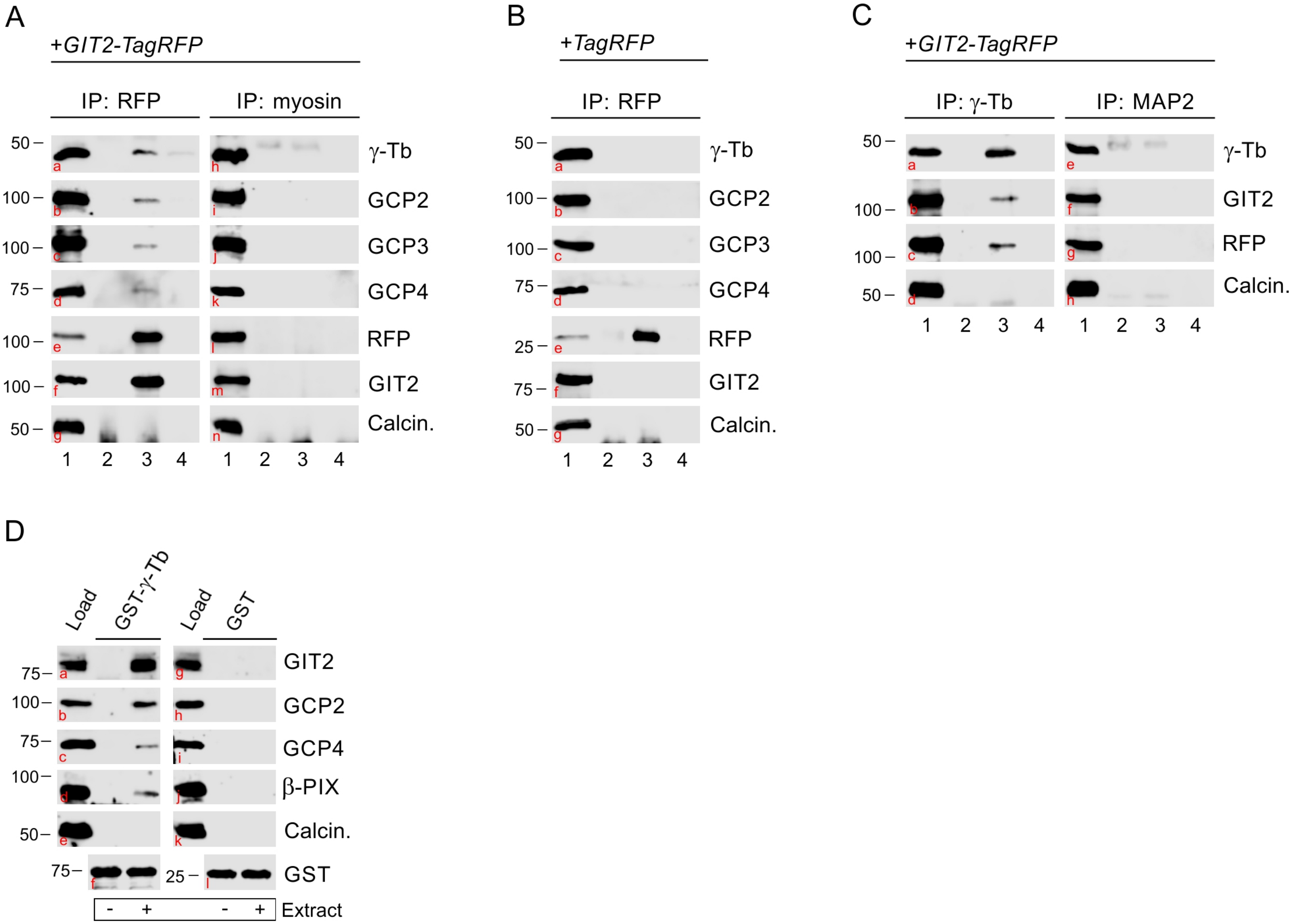

A
+GIT2-TagRFP
IP: RFP
IP: myosin
γ-Tb
GCP2
GCP3
GCP4
RFP
GIT2
Calcin.
50
100
100
75
100
100
50
1 2 3 4
1 2 3 4
B
+TagRFP
IP: RFP
γ-Tb
GCP2
GCP3
GCP4
RFP
GIT2
Calcin.
50
100
100
75
25
75
50
1 2 3 4
C
+GIT2-TagRFP
IP: γ-Tb
IP: MAP2
γ-Tb
GIT2
RFP
Calcin.
50
100
100
50
1 2 3 4
1 2 3 4
D
Load
GST-γ-Tb
Load
GST
GIT2
GCP2
GCP4
β-PIX
Calcin.
GST
75
100
75
100
50
75
25
- + - + Extract

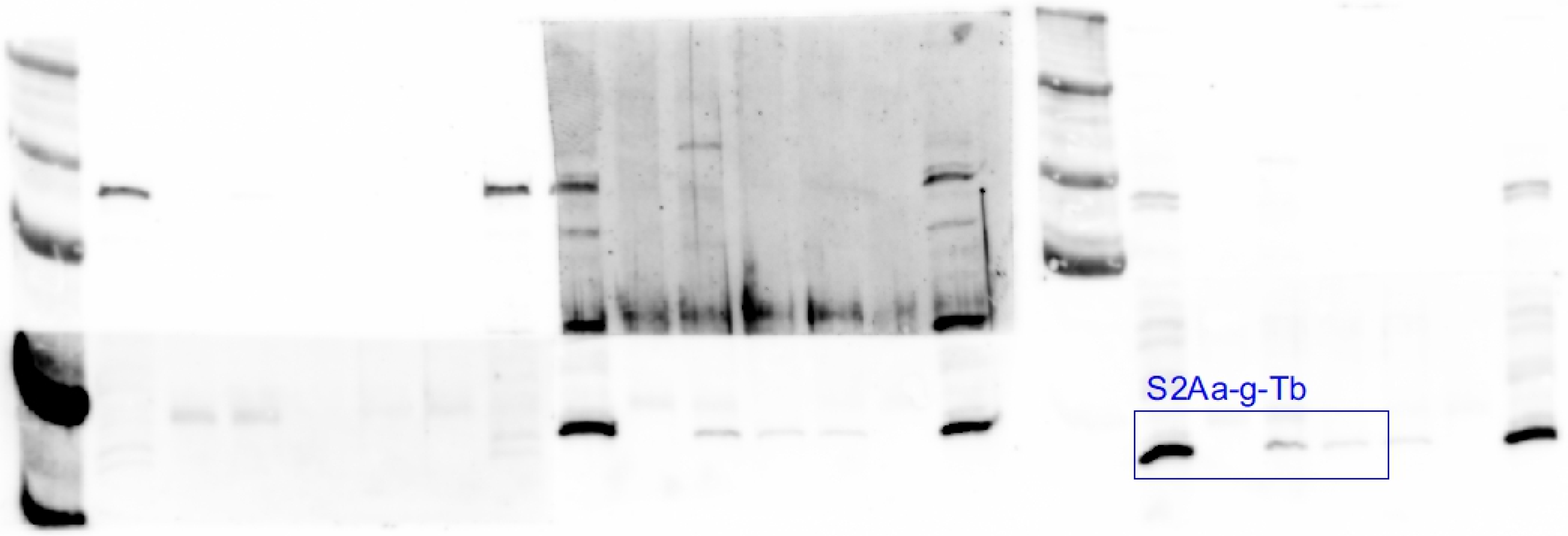
S2Aa-g-Tb

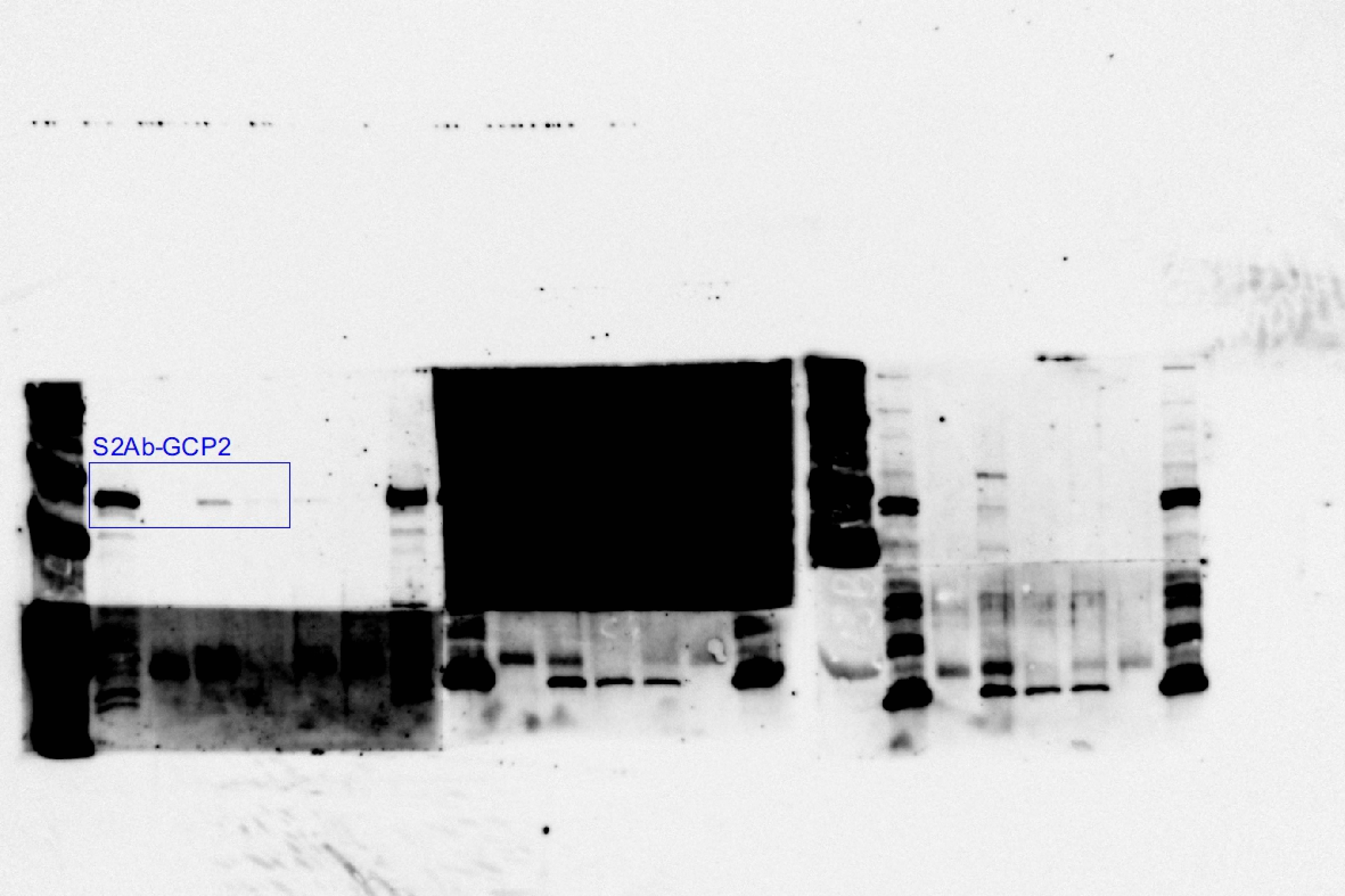
S2Ab-GCP2

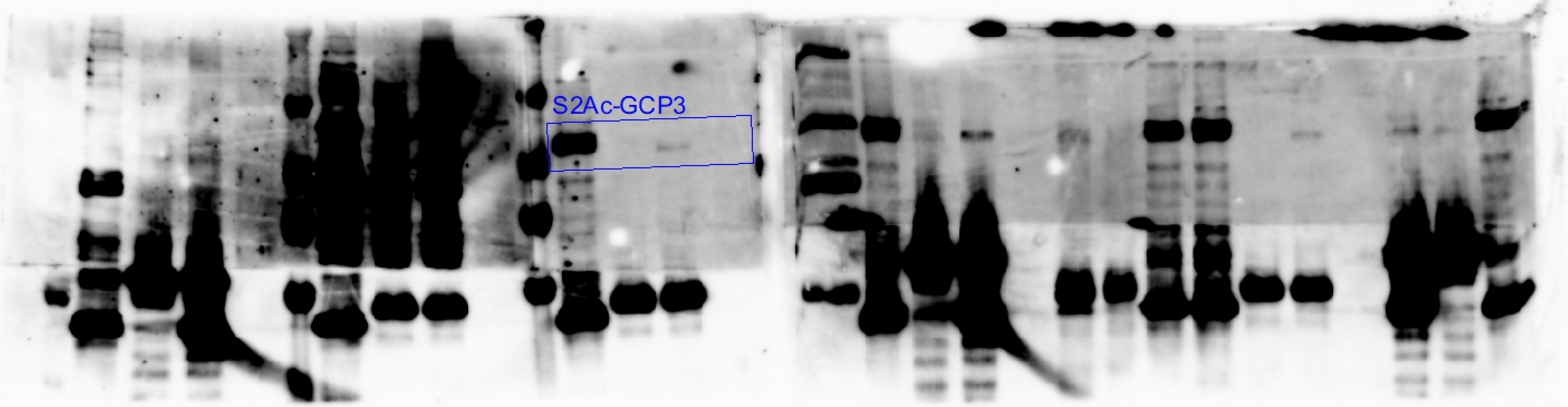
S2Ac-GCP3

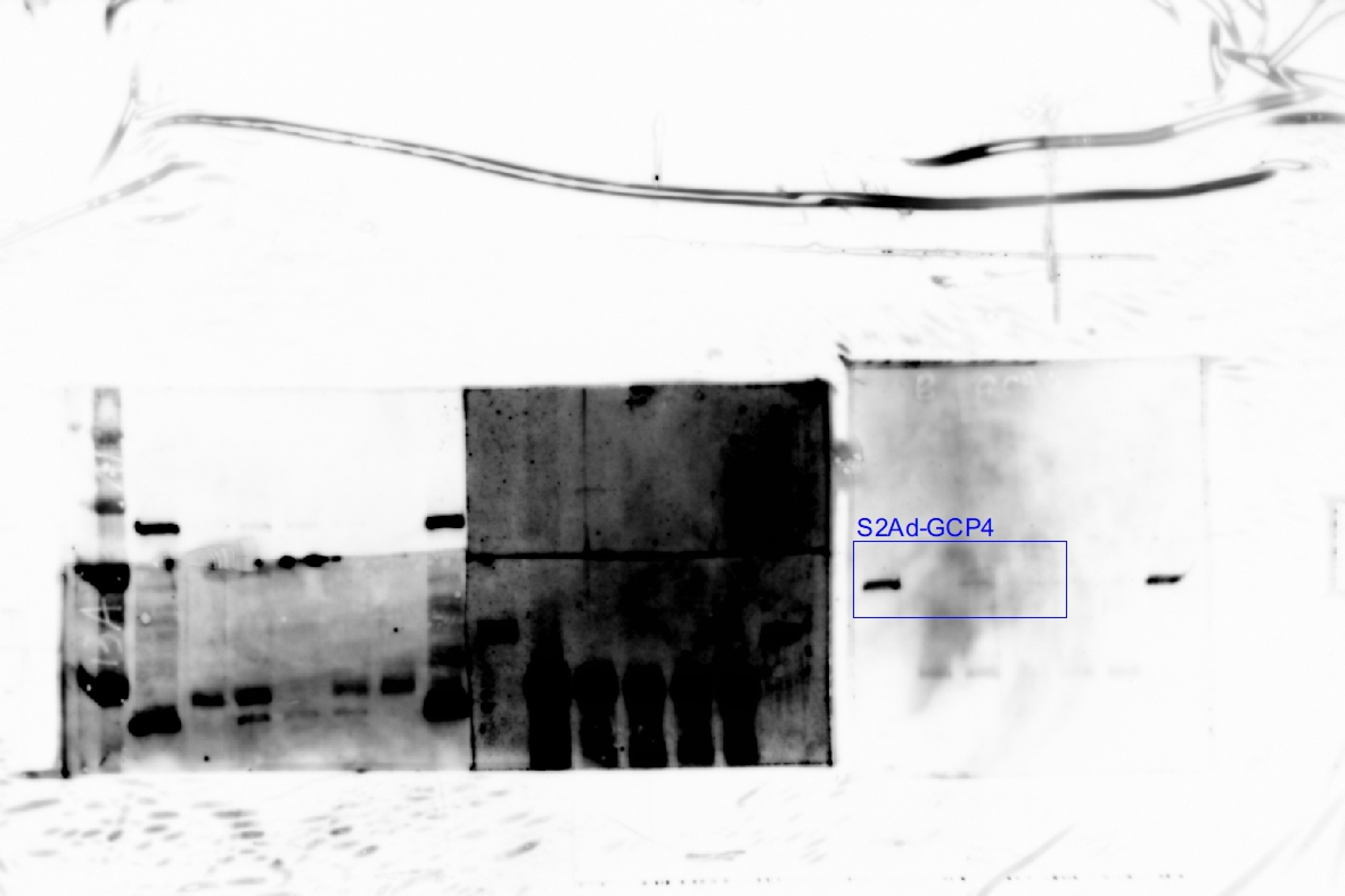
S2Ad-GCP4

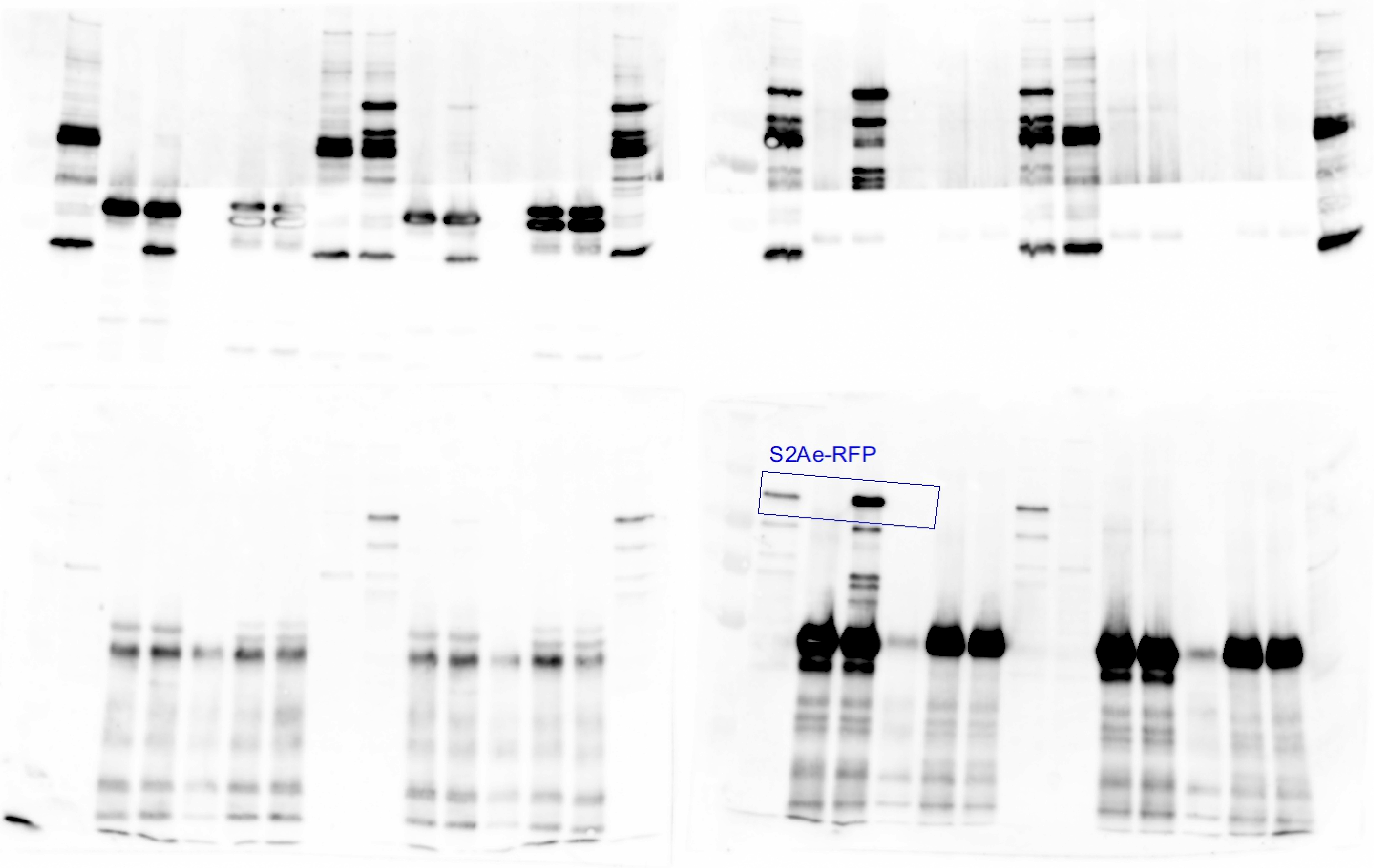
S2Ae-RFP

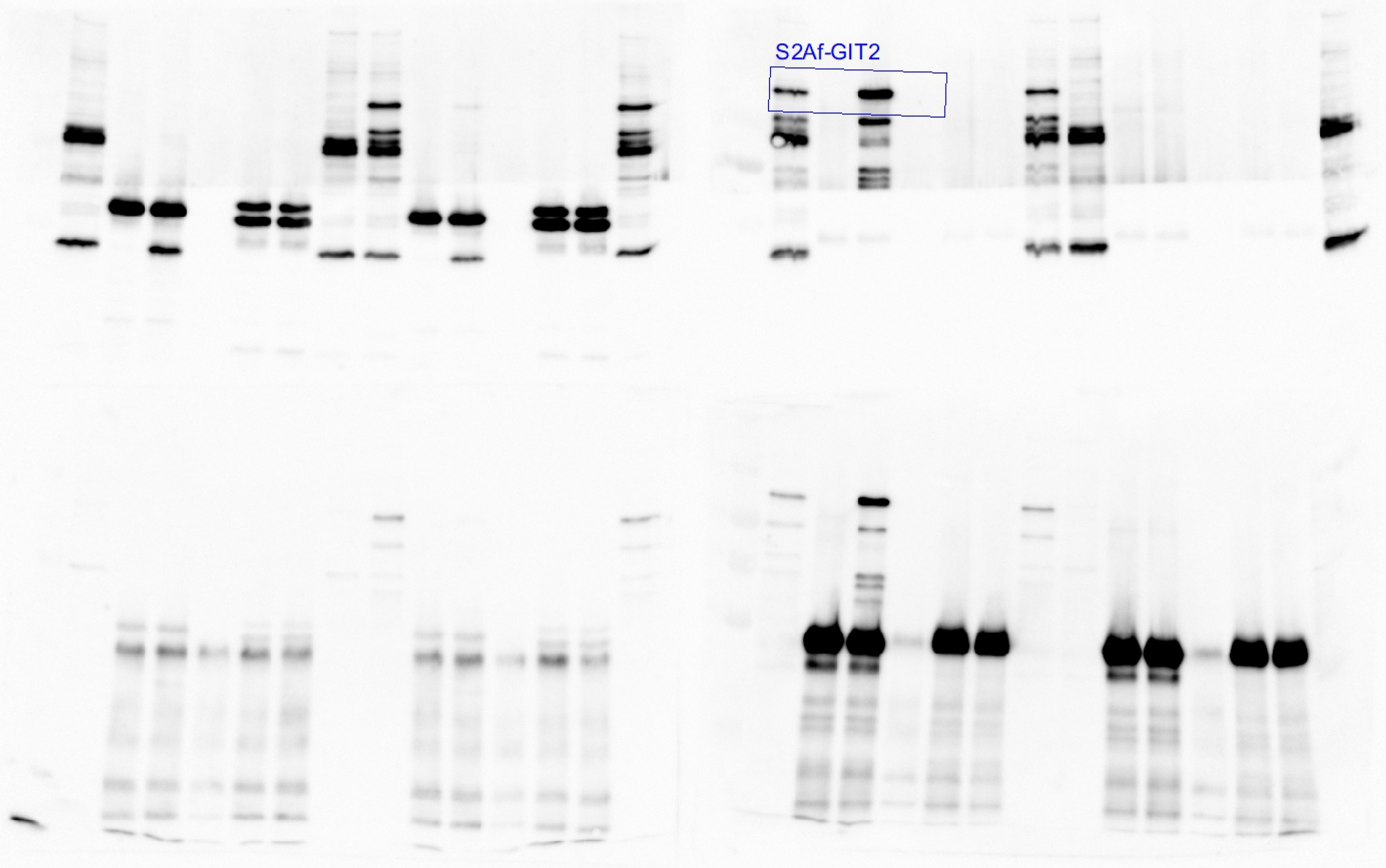
S2Af-GIT2

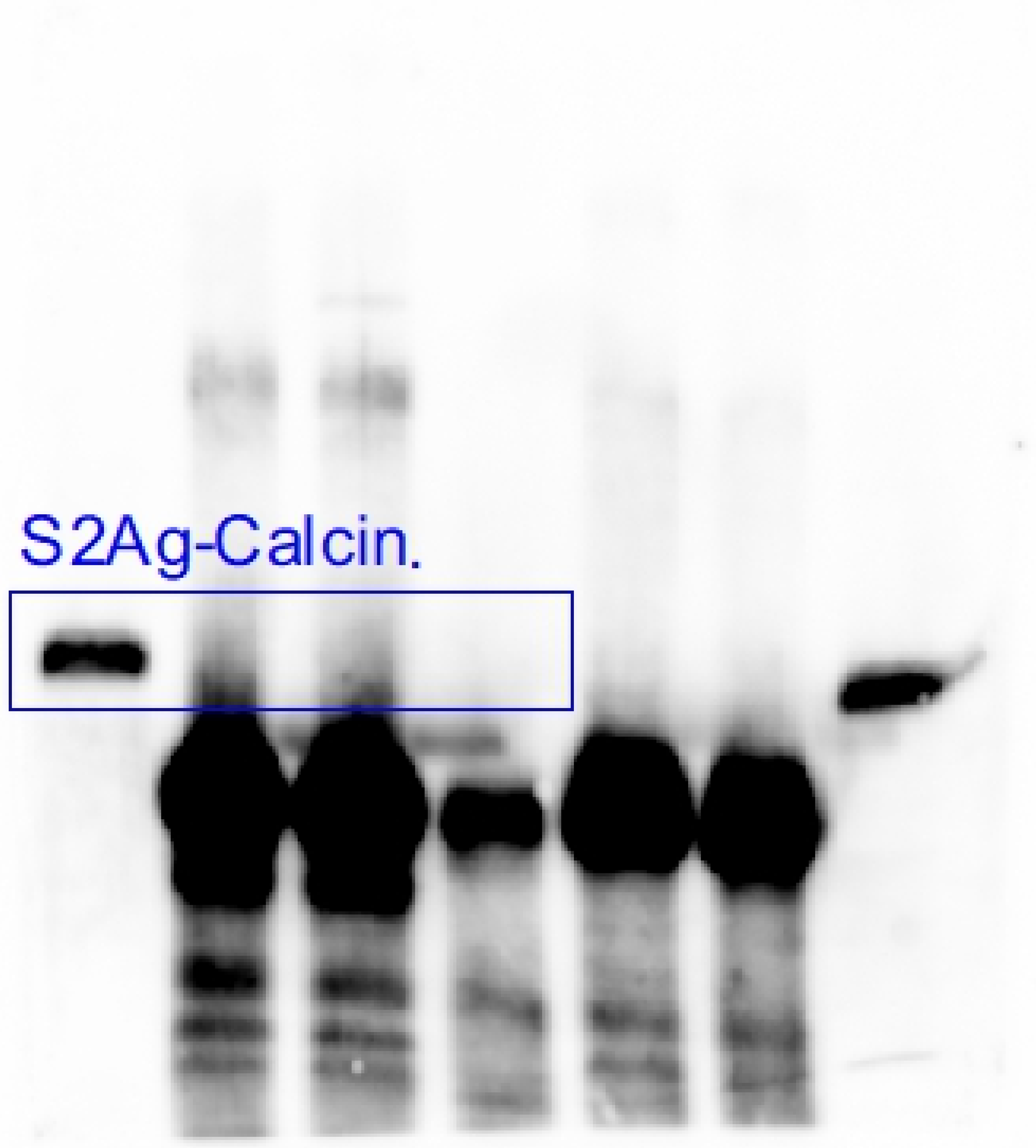
S2Ag-Calcin.

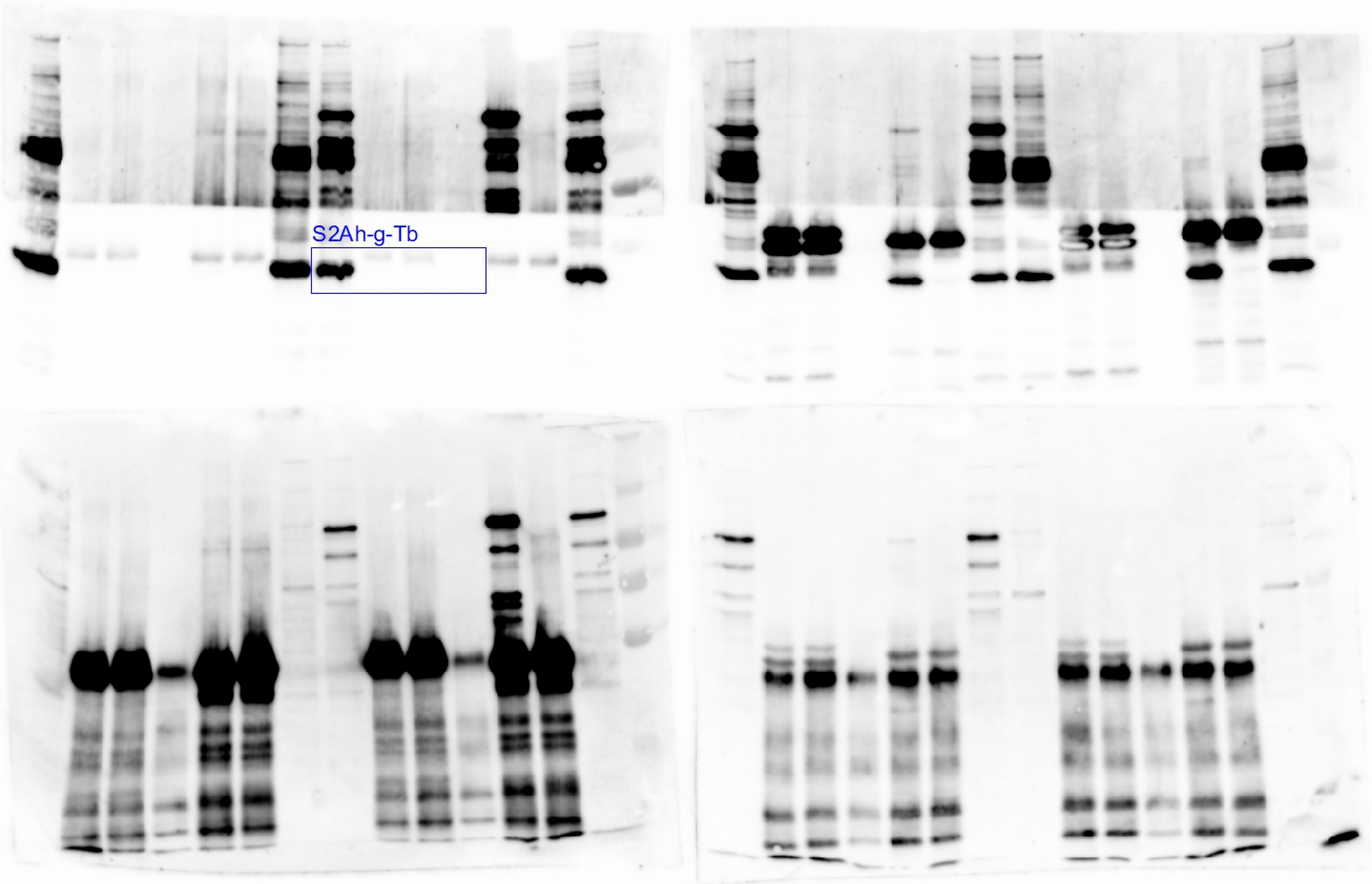
S2Ah-g-Tb

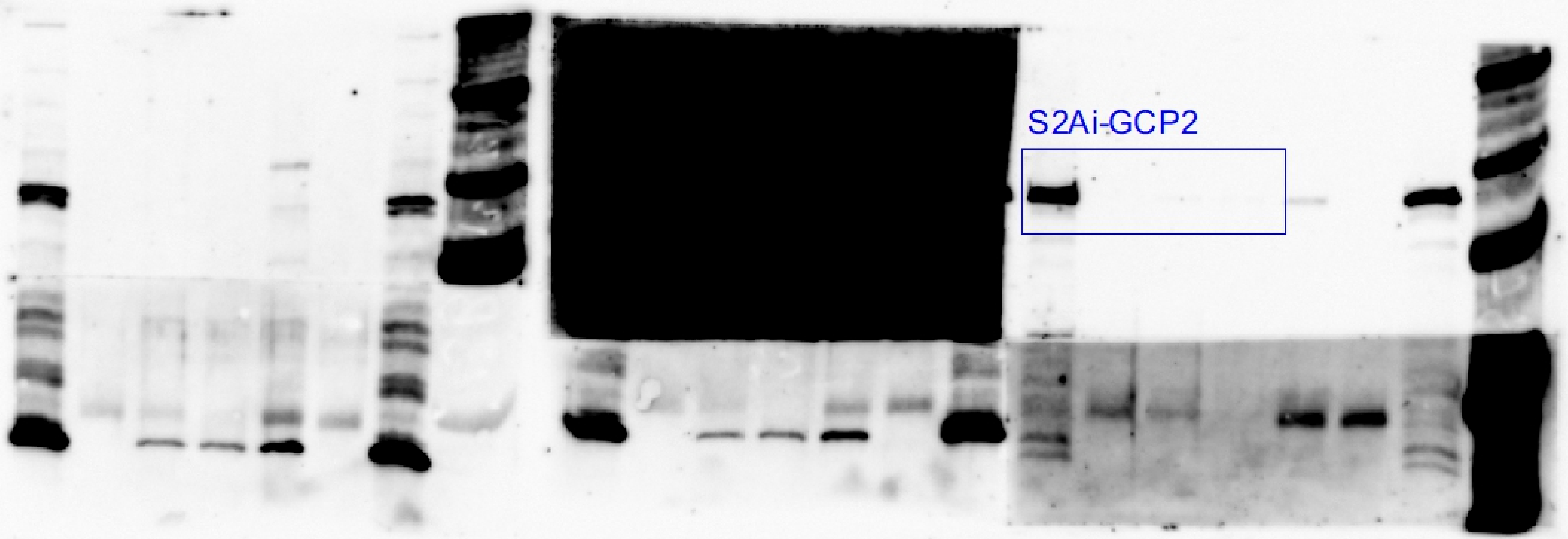
S2Ai-GCP2

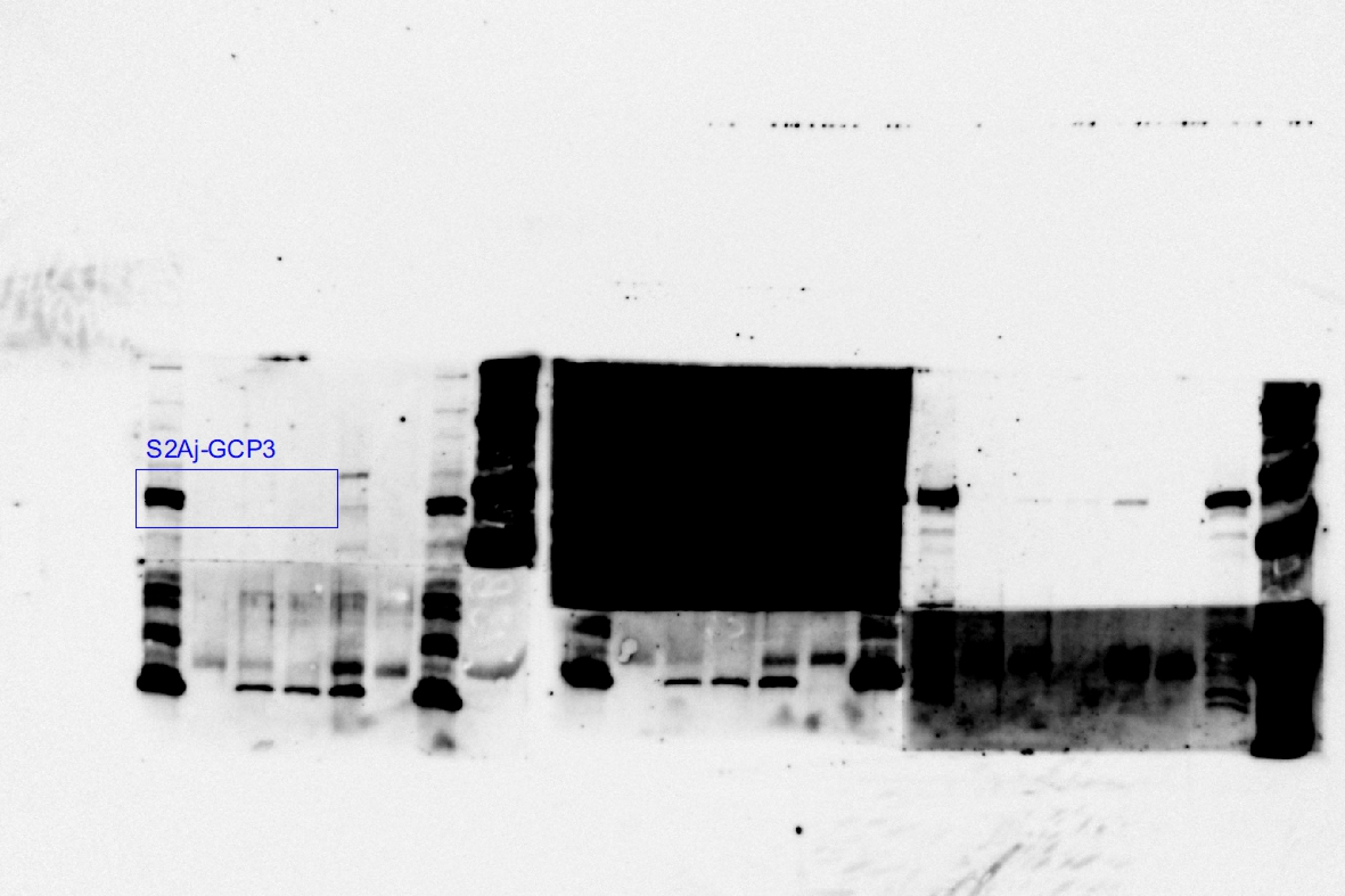
S2Aj-GCP3

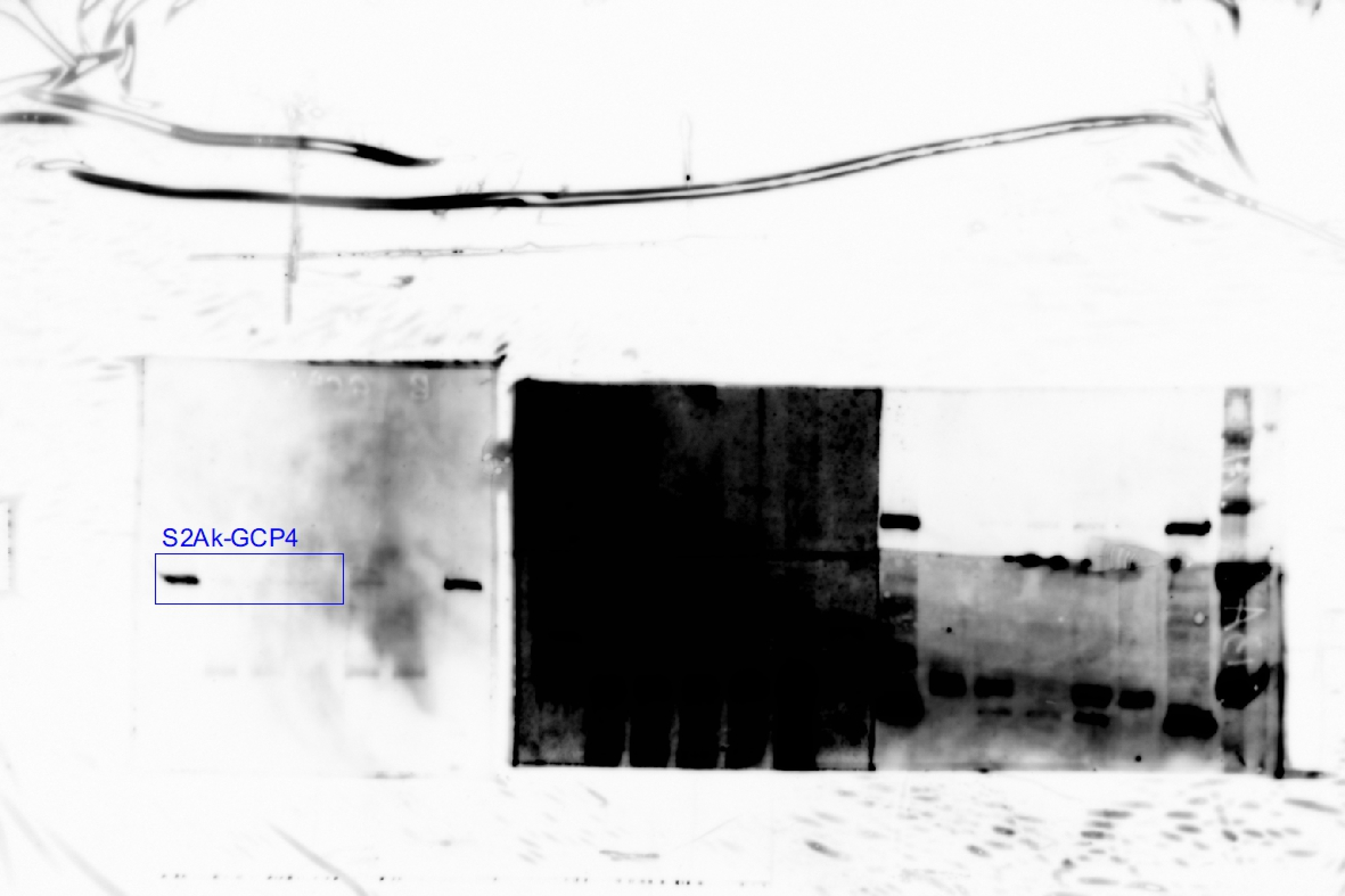
S2Ak-GCP4

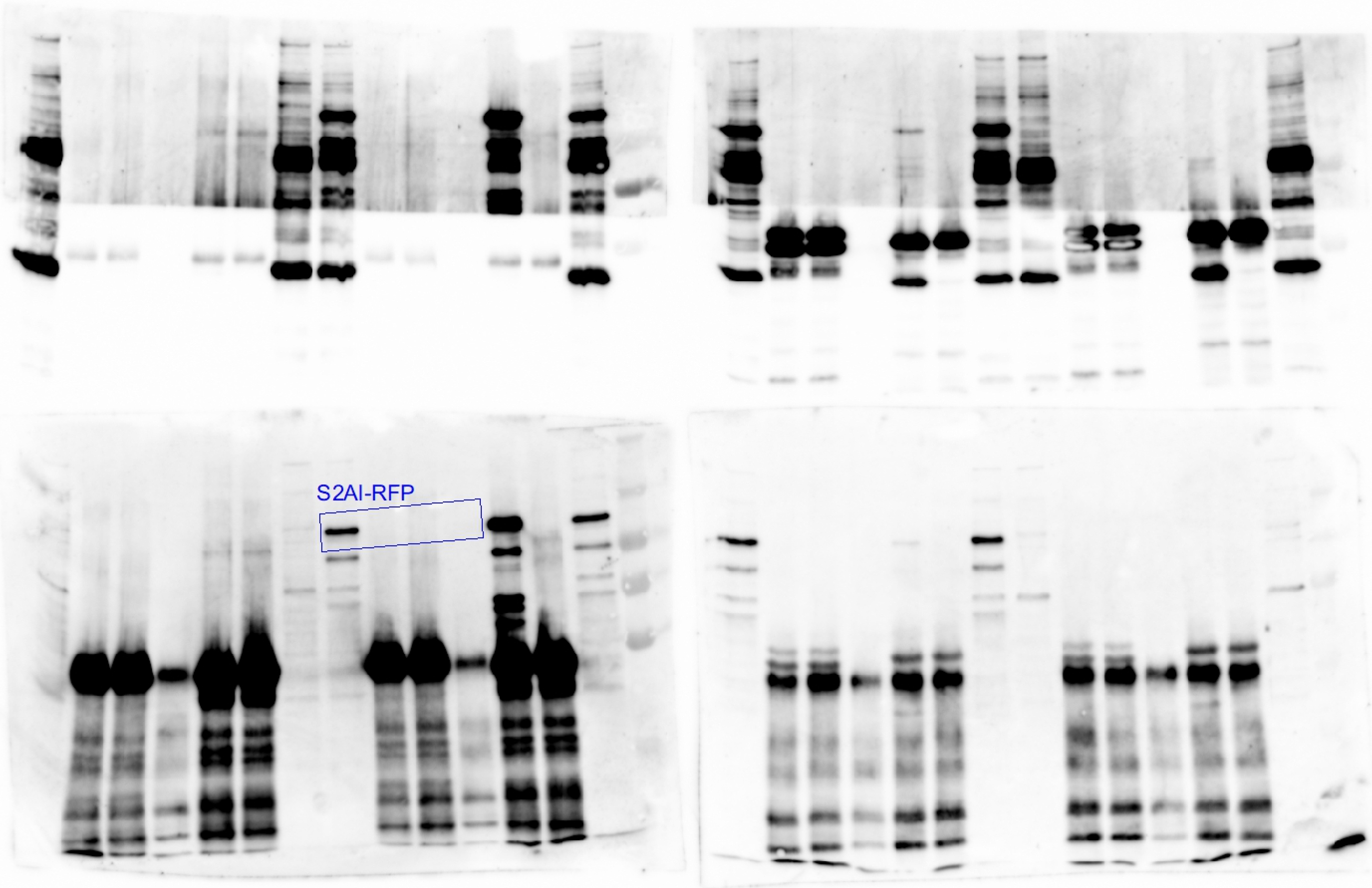
S2AI-RFP

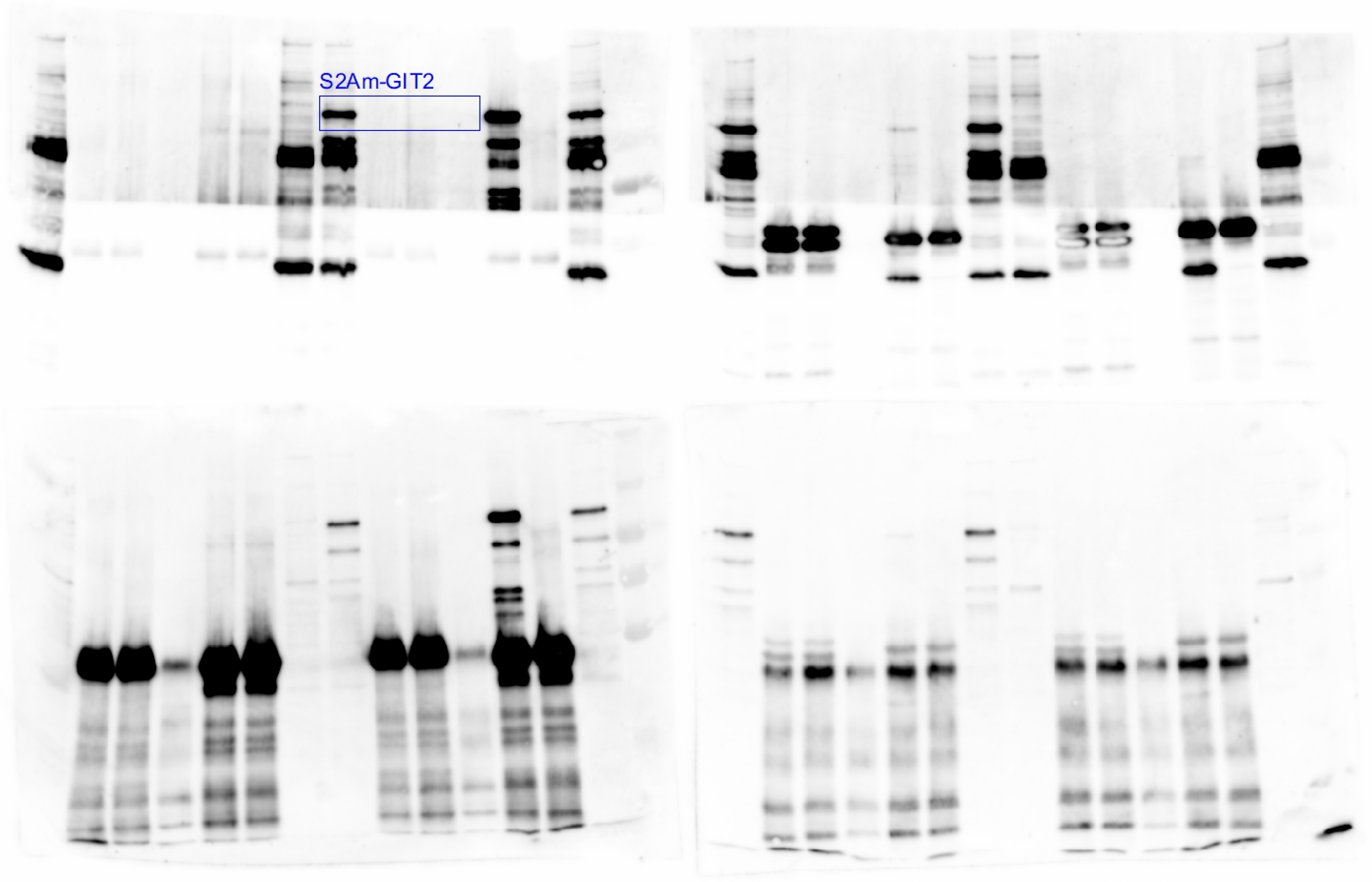
S2Am-GIT2

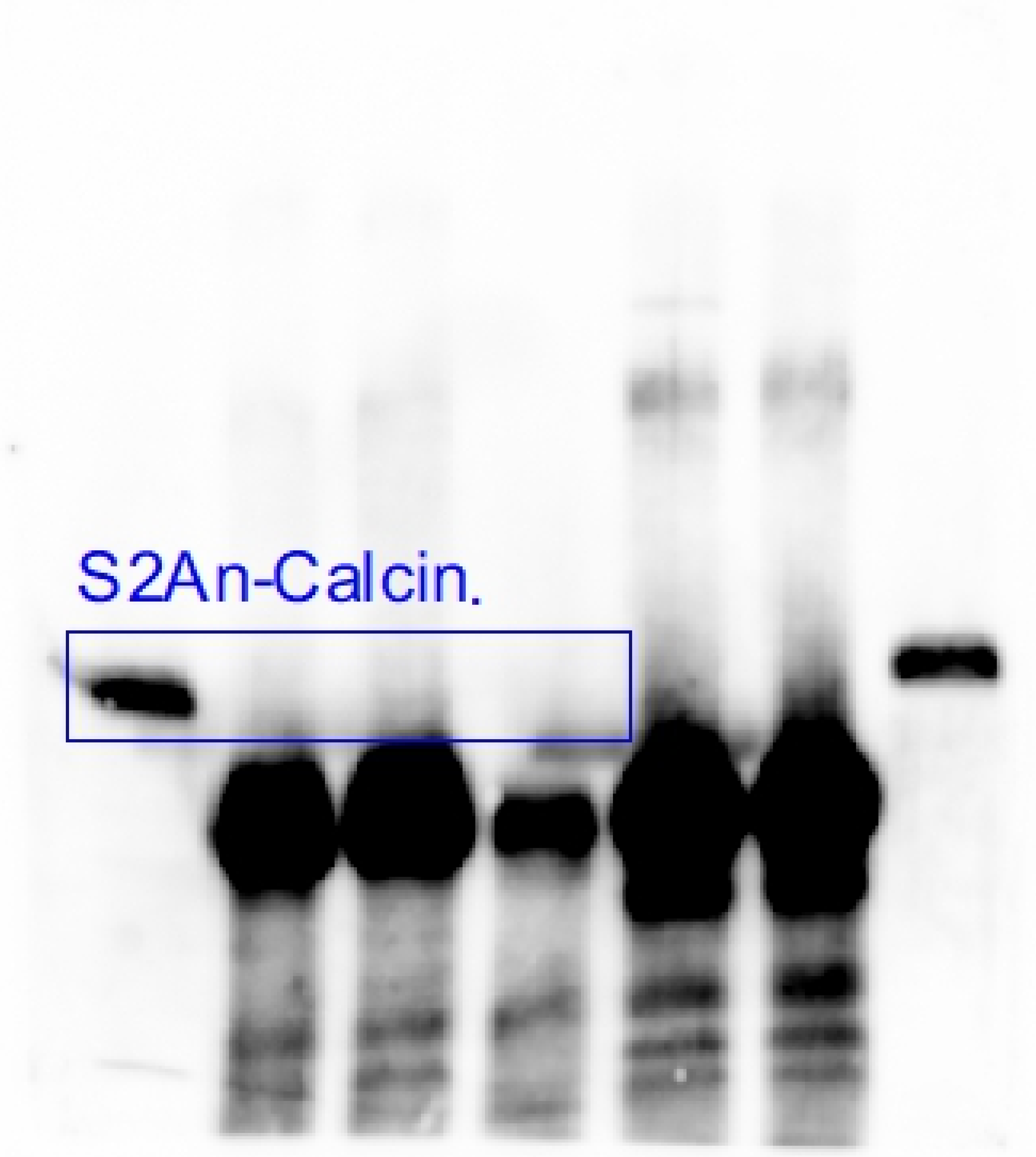
S2An-Calcin.

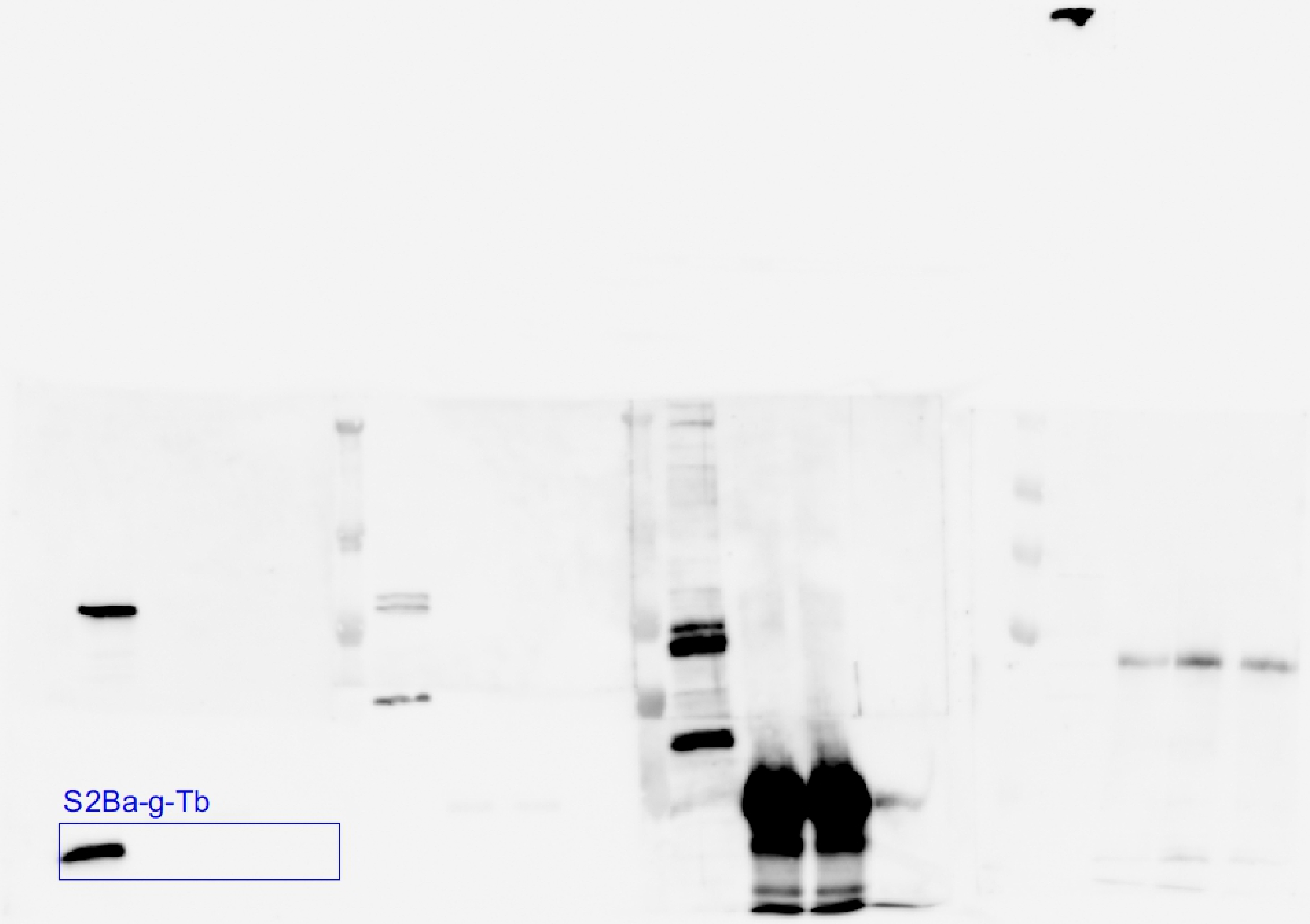
S2Ba-g-Tb

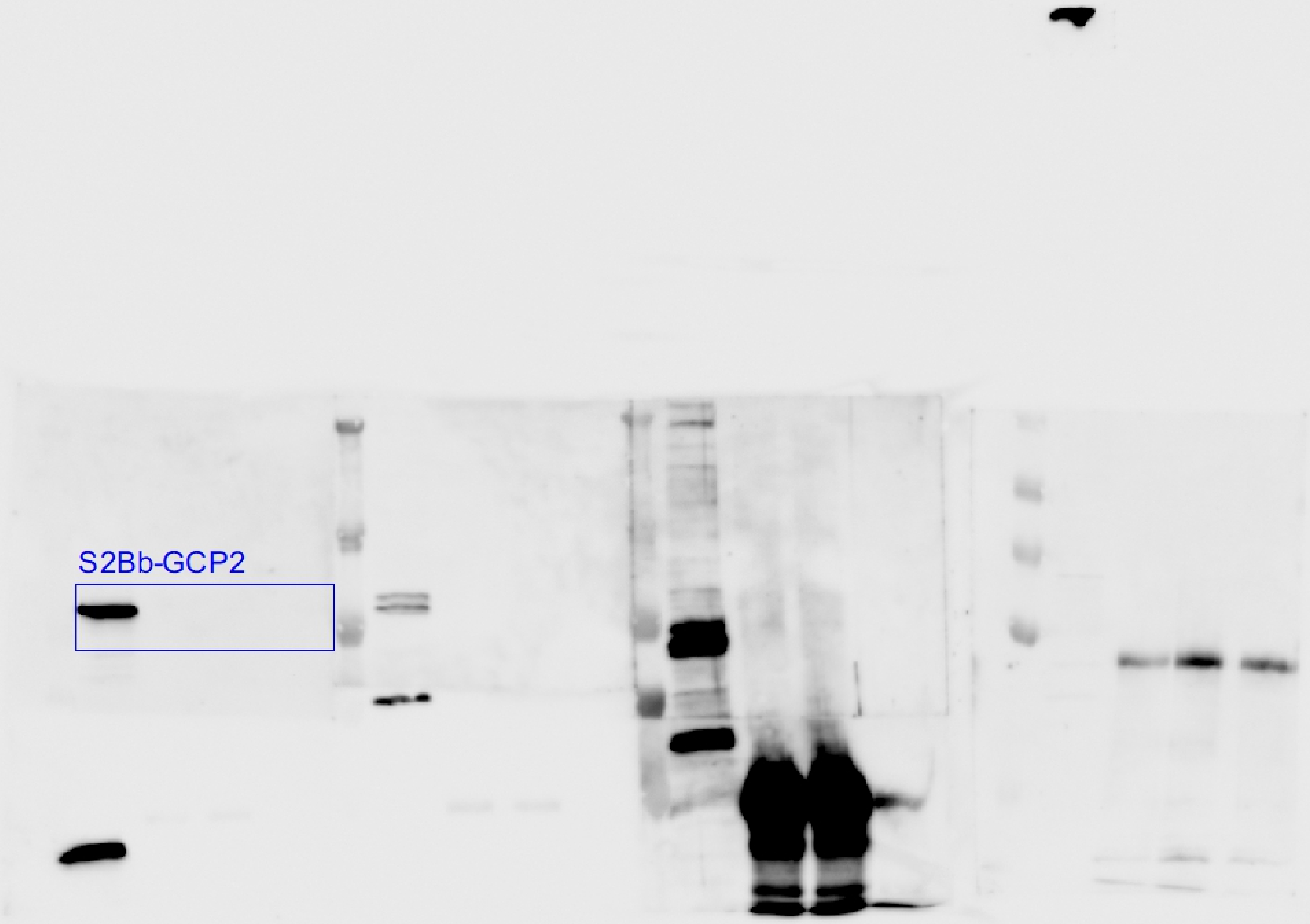
S2Bb-GCP2

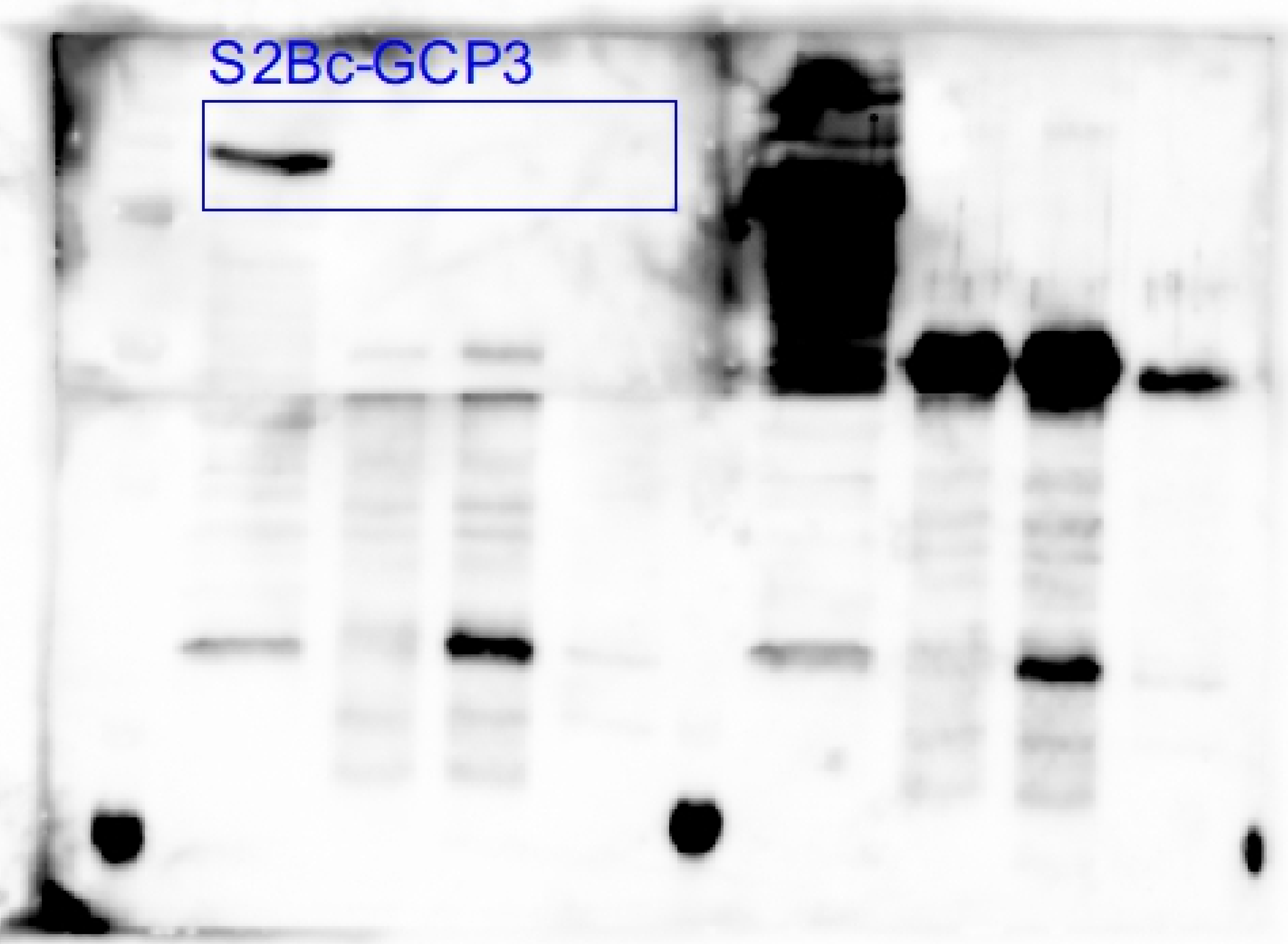
S2Bc-GCP3

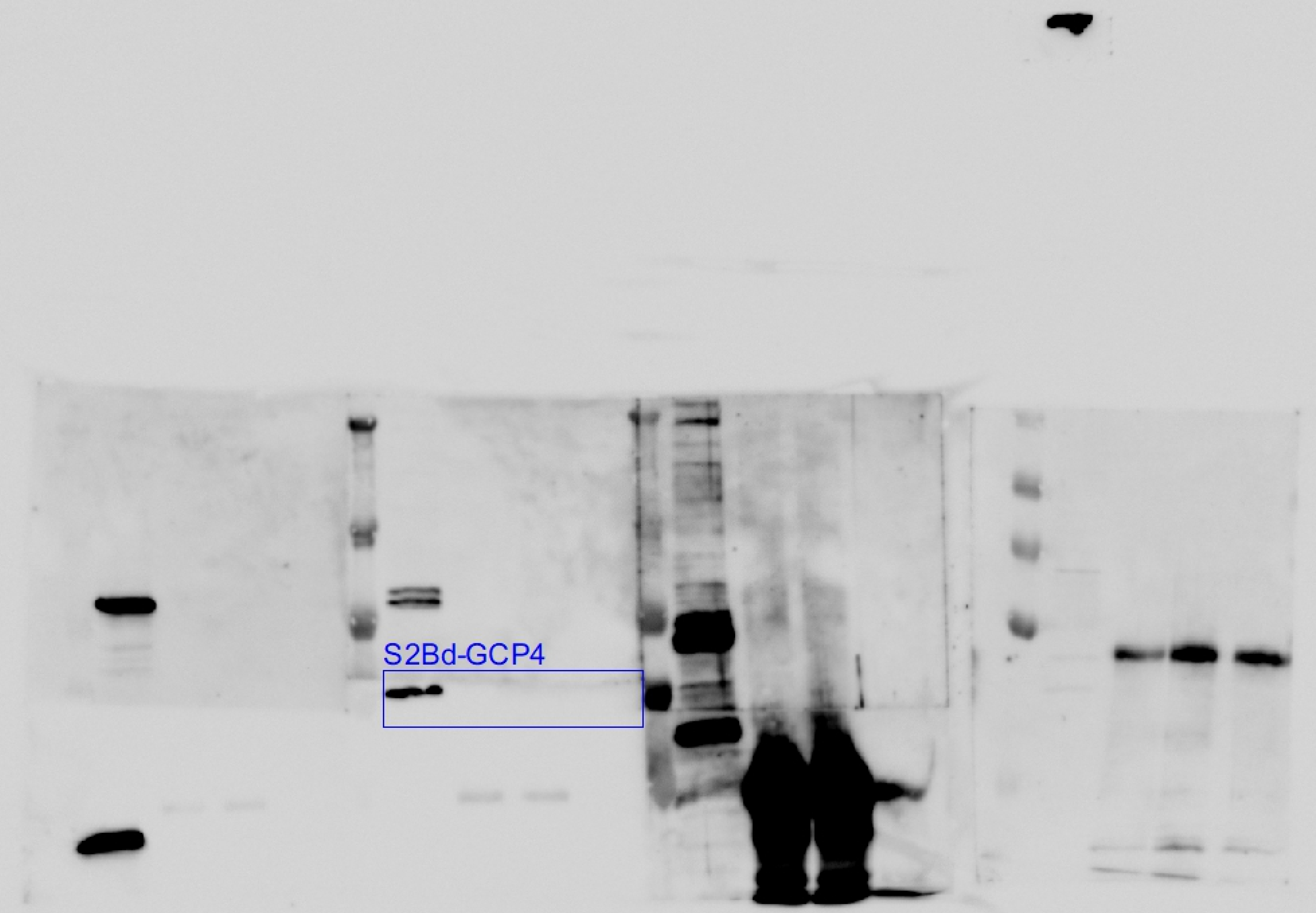
S2Bd-GCP4

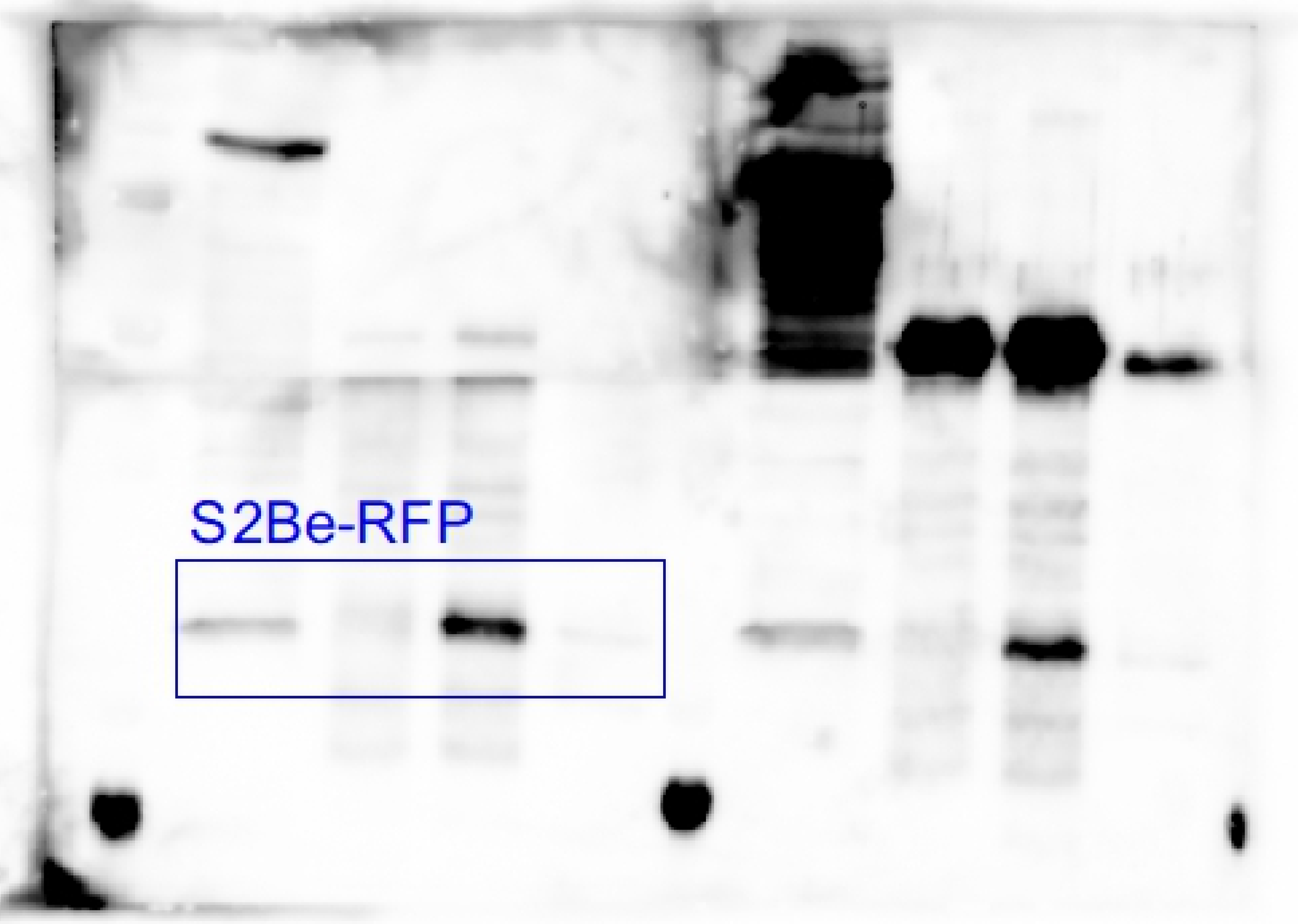
S2Be-RFP

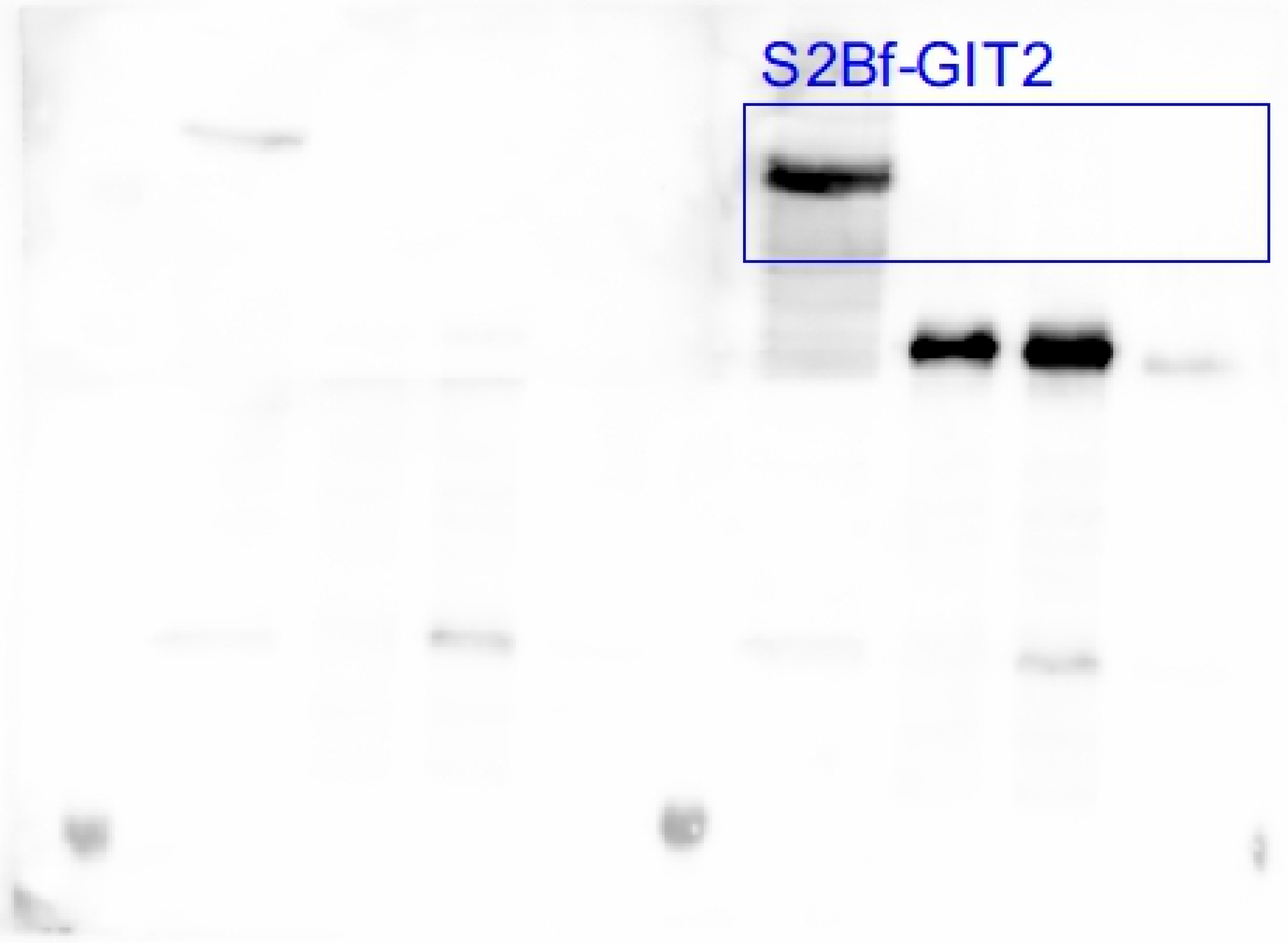
S2Bf-GIT2

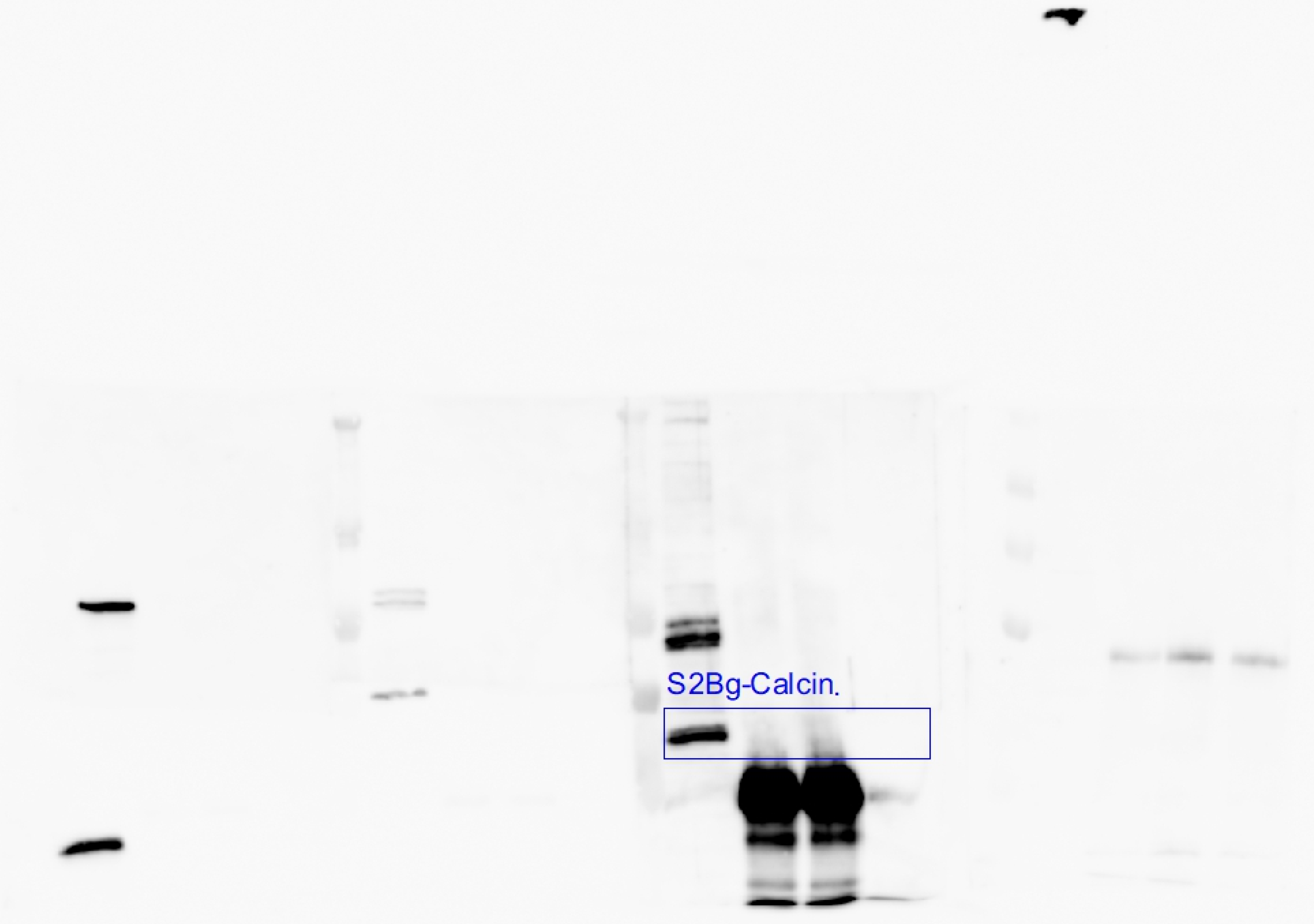
S2Bg-Calcin.

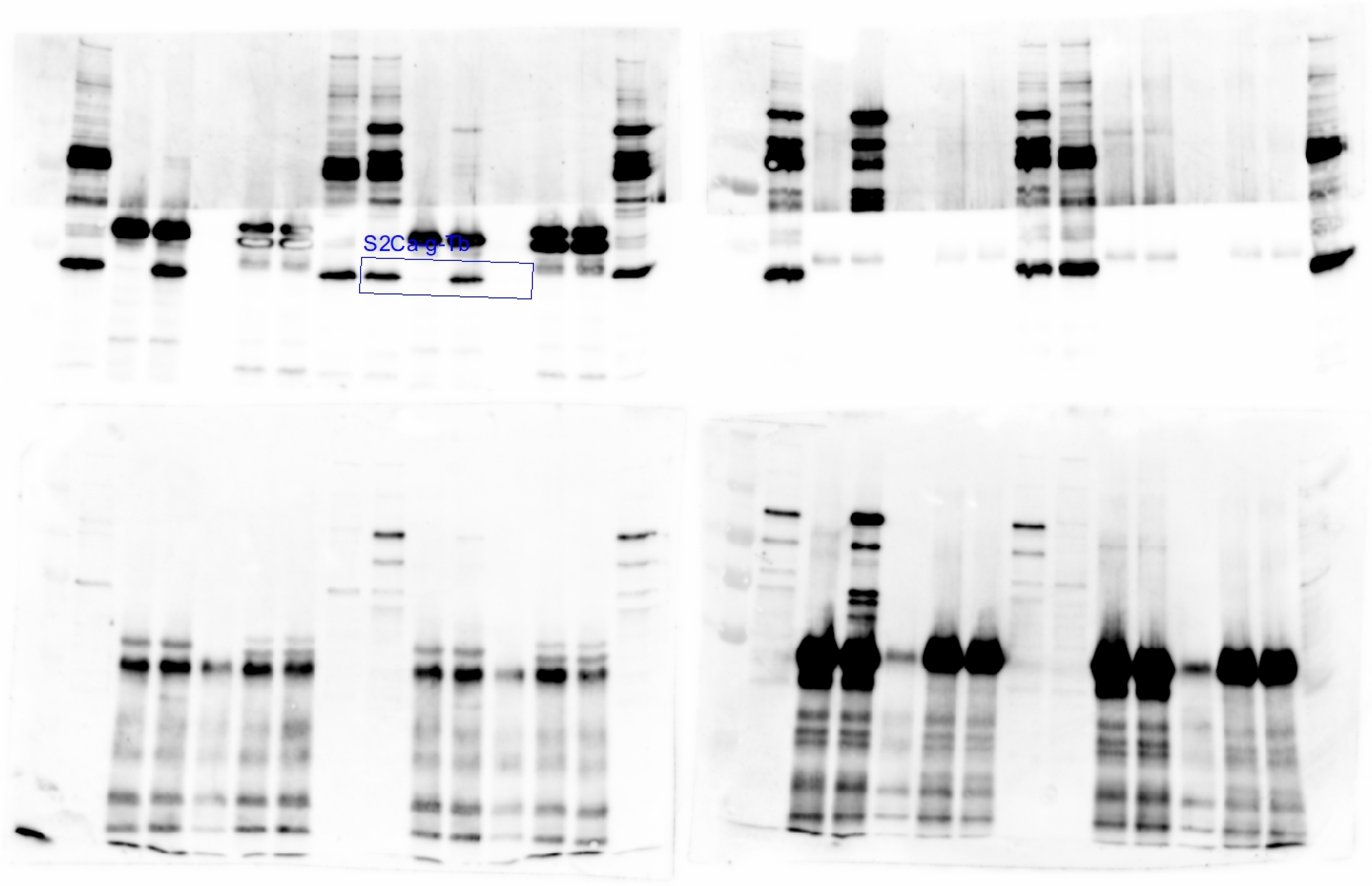
S2Ca-g-Tb

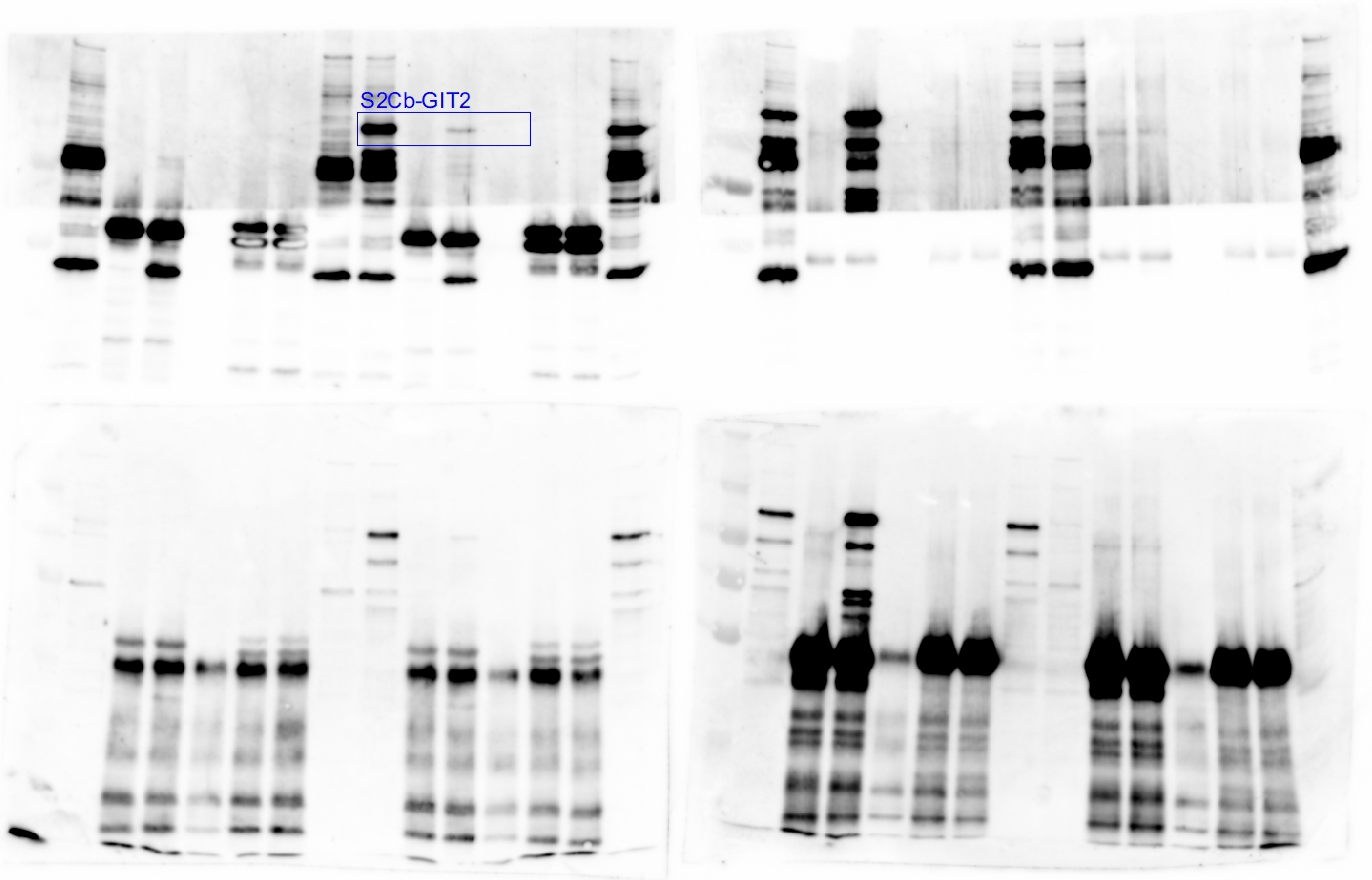
S2Cb-GIT2

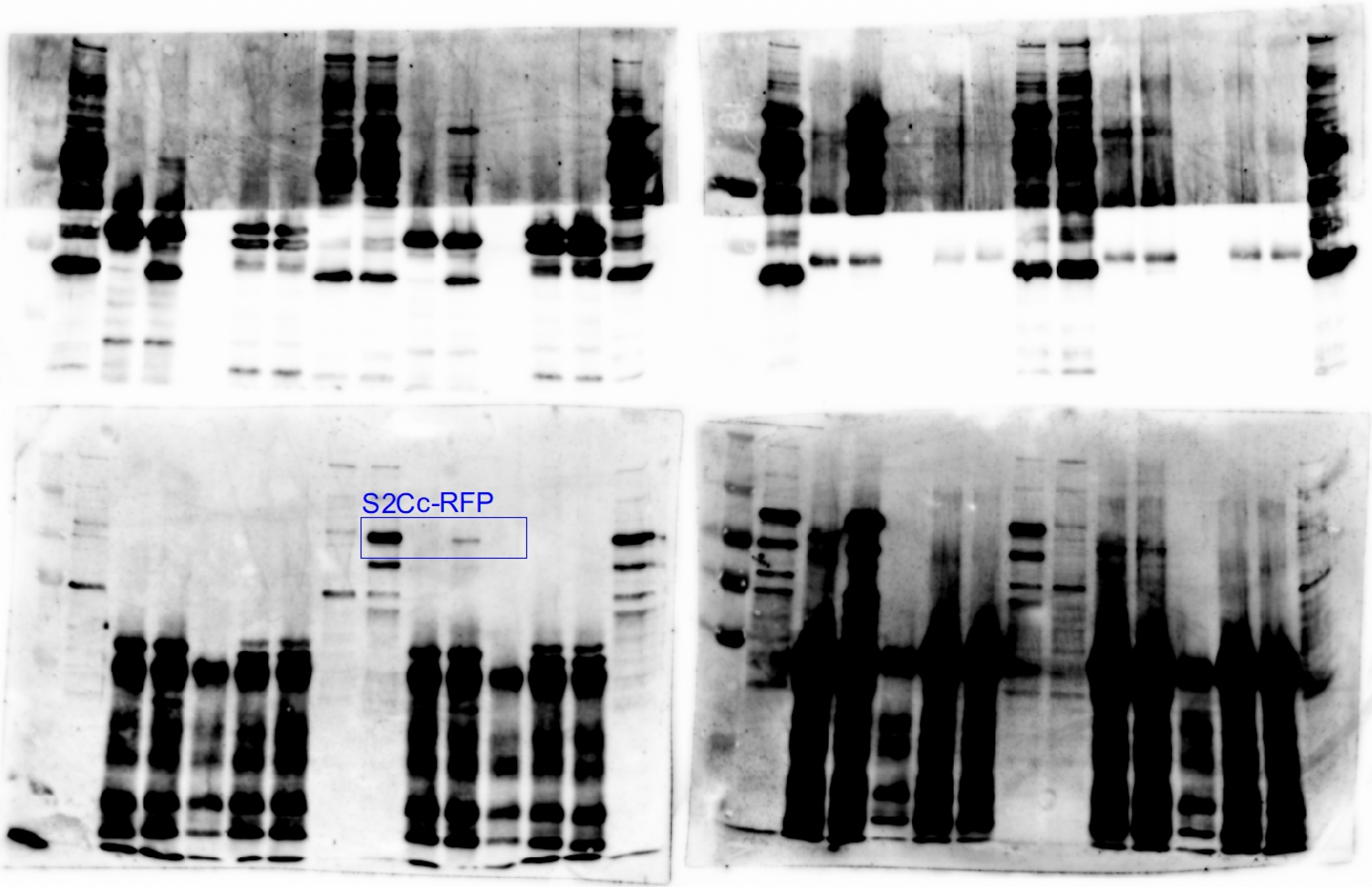
S2Cc-RFP

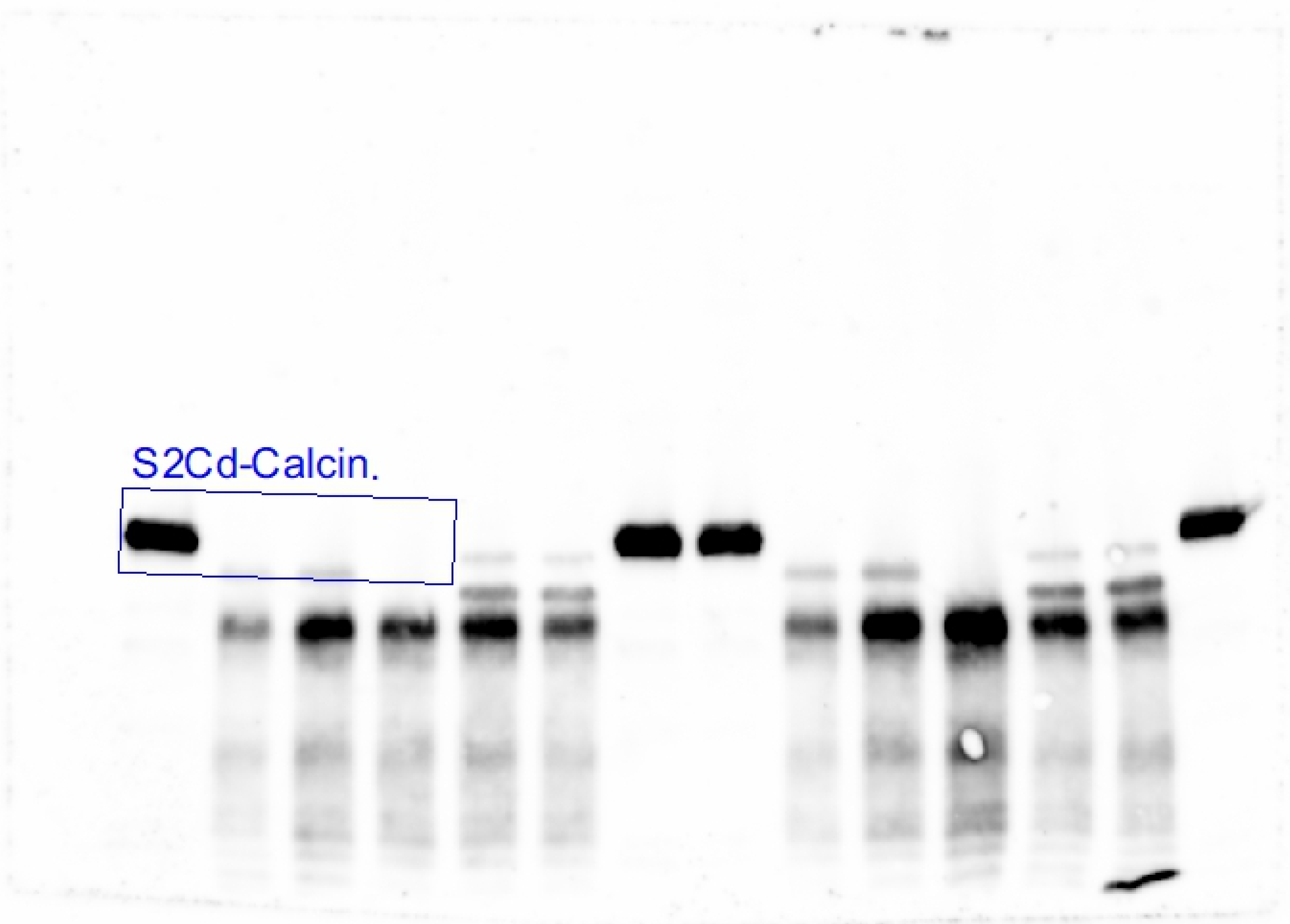
S2Cd-Calcin.

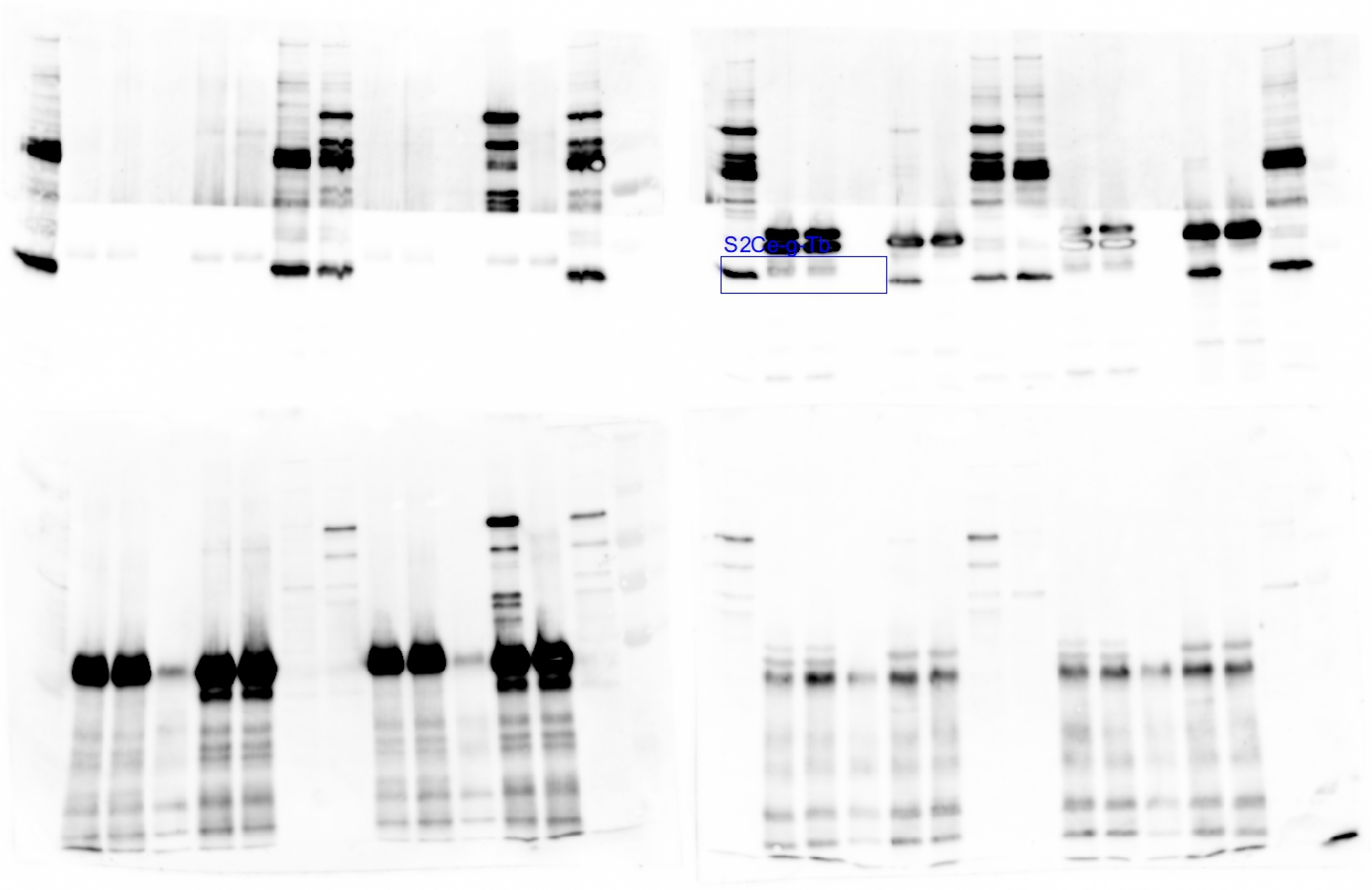

S2Ce-g-Tb

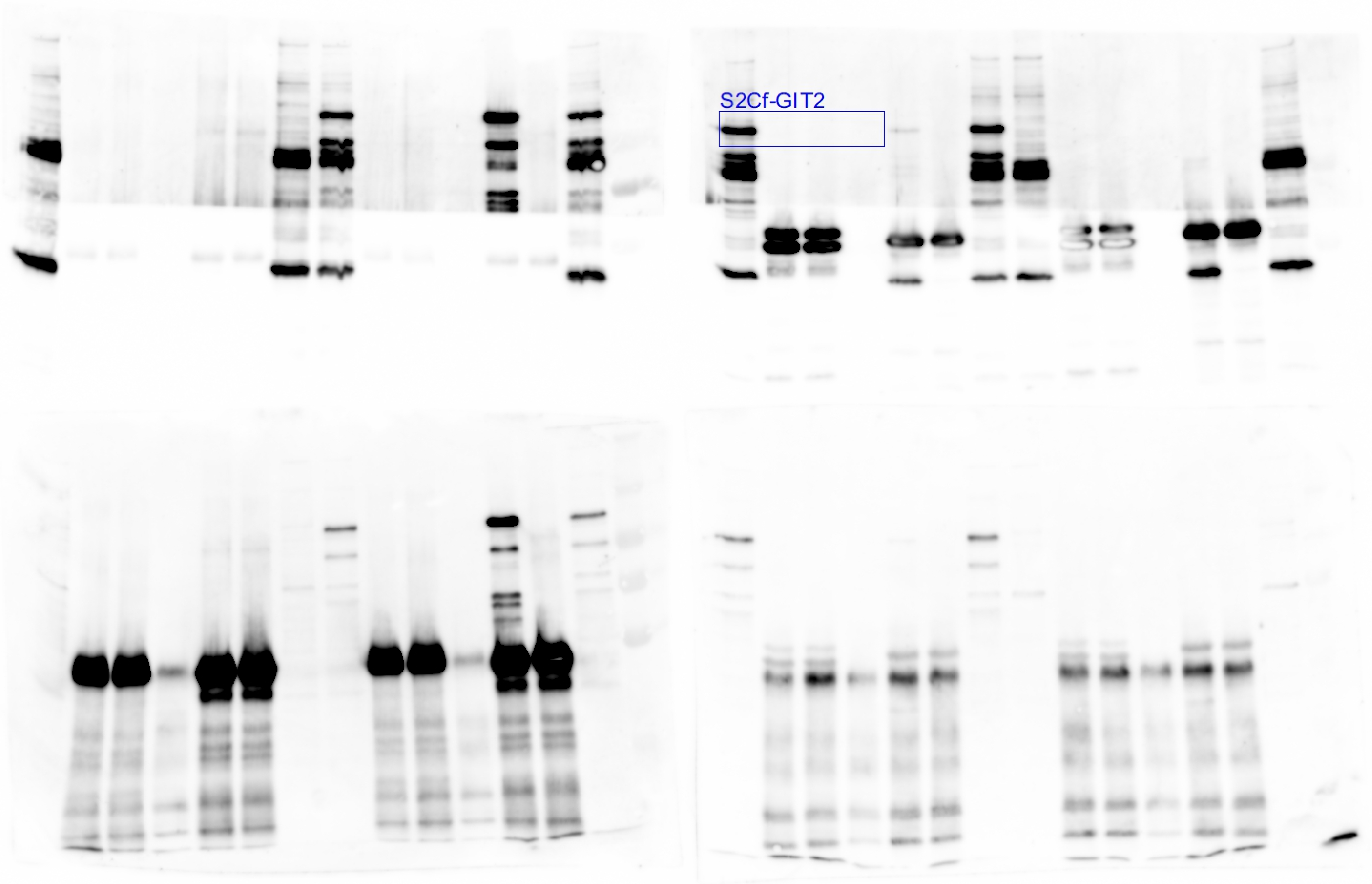
S2Cf-GIT2

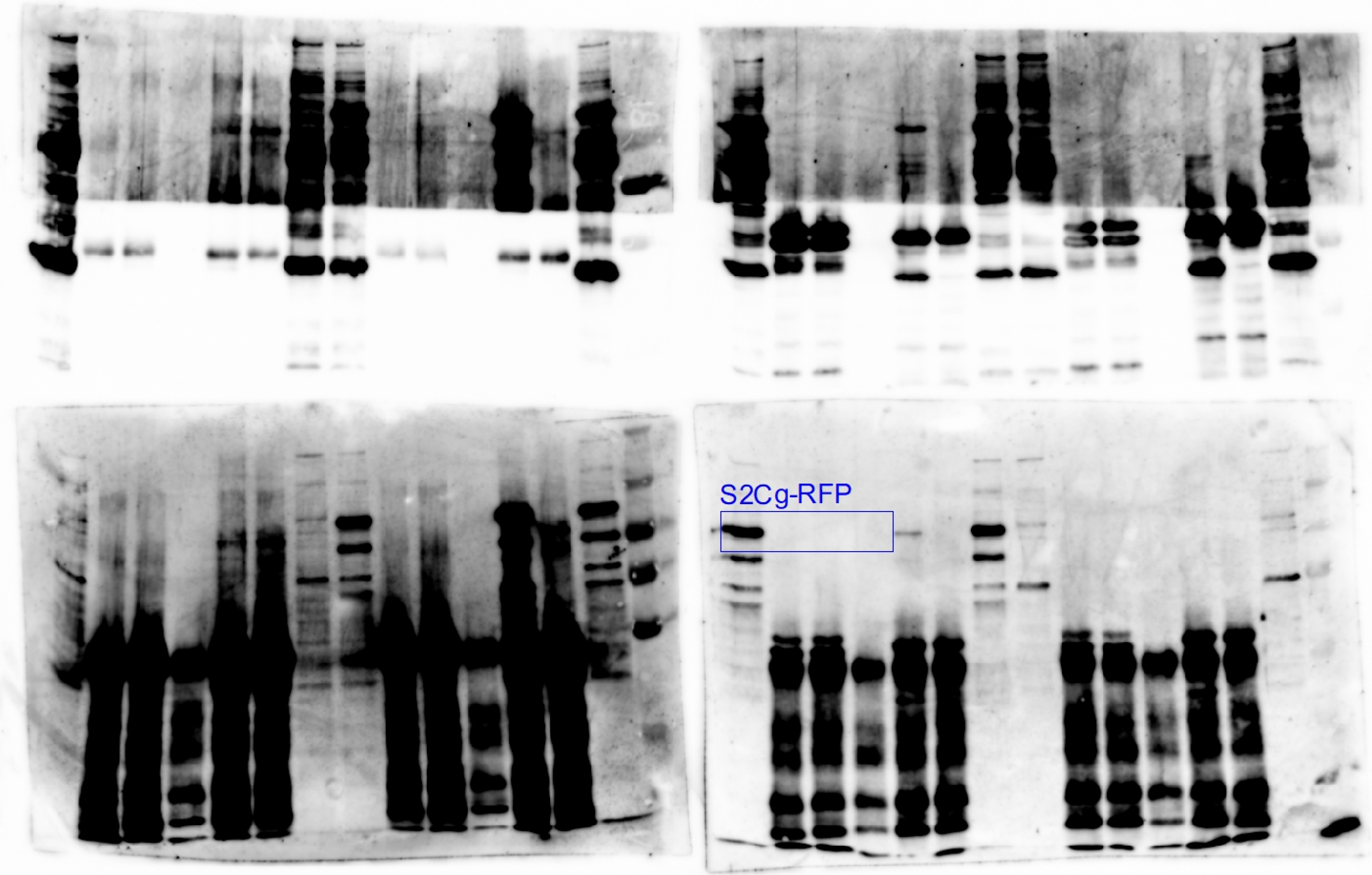
S2Cg-RFP

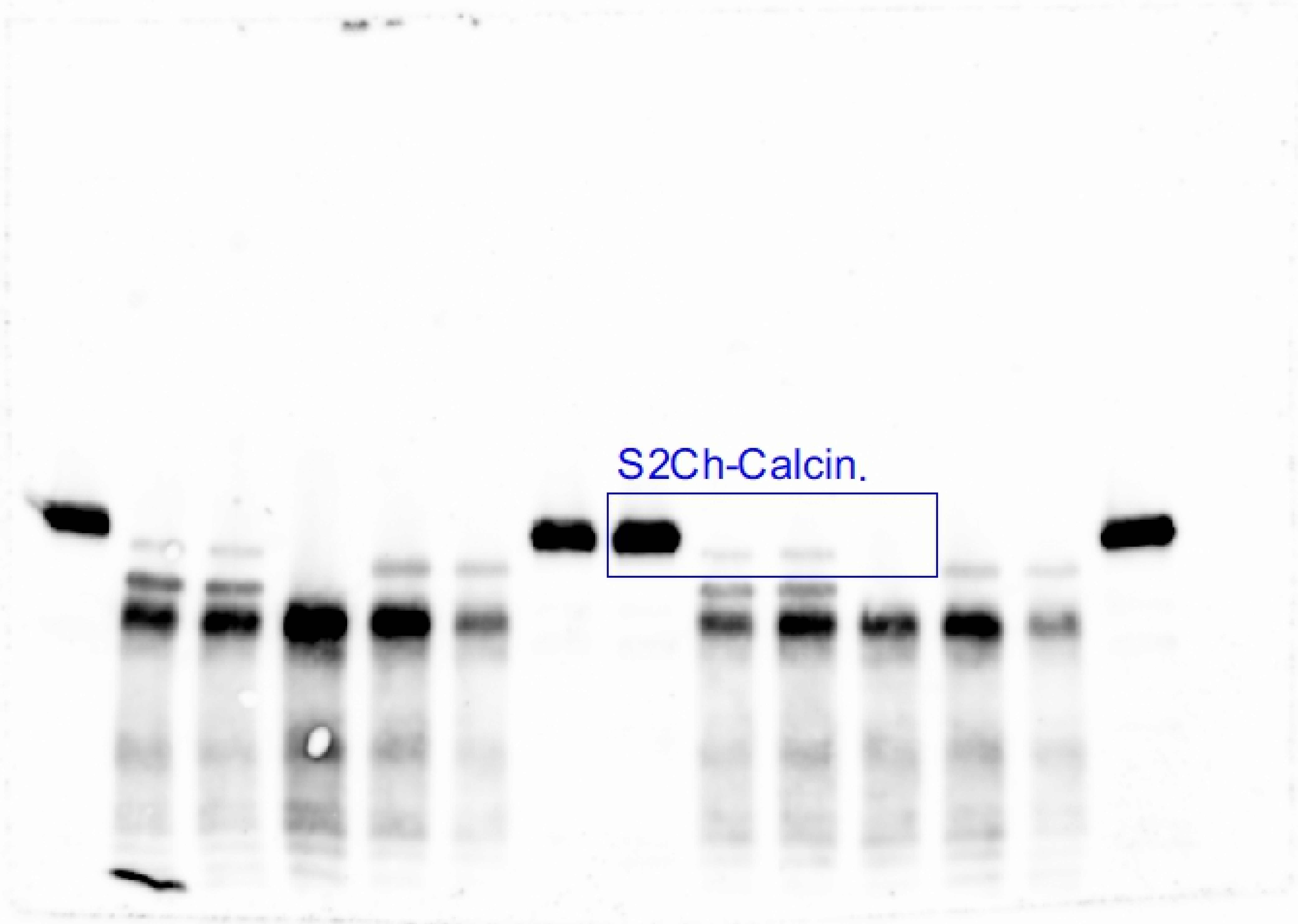
S2Ch-Calcin.

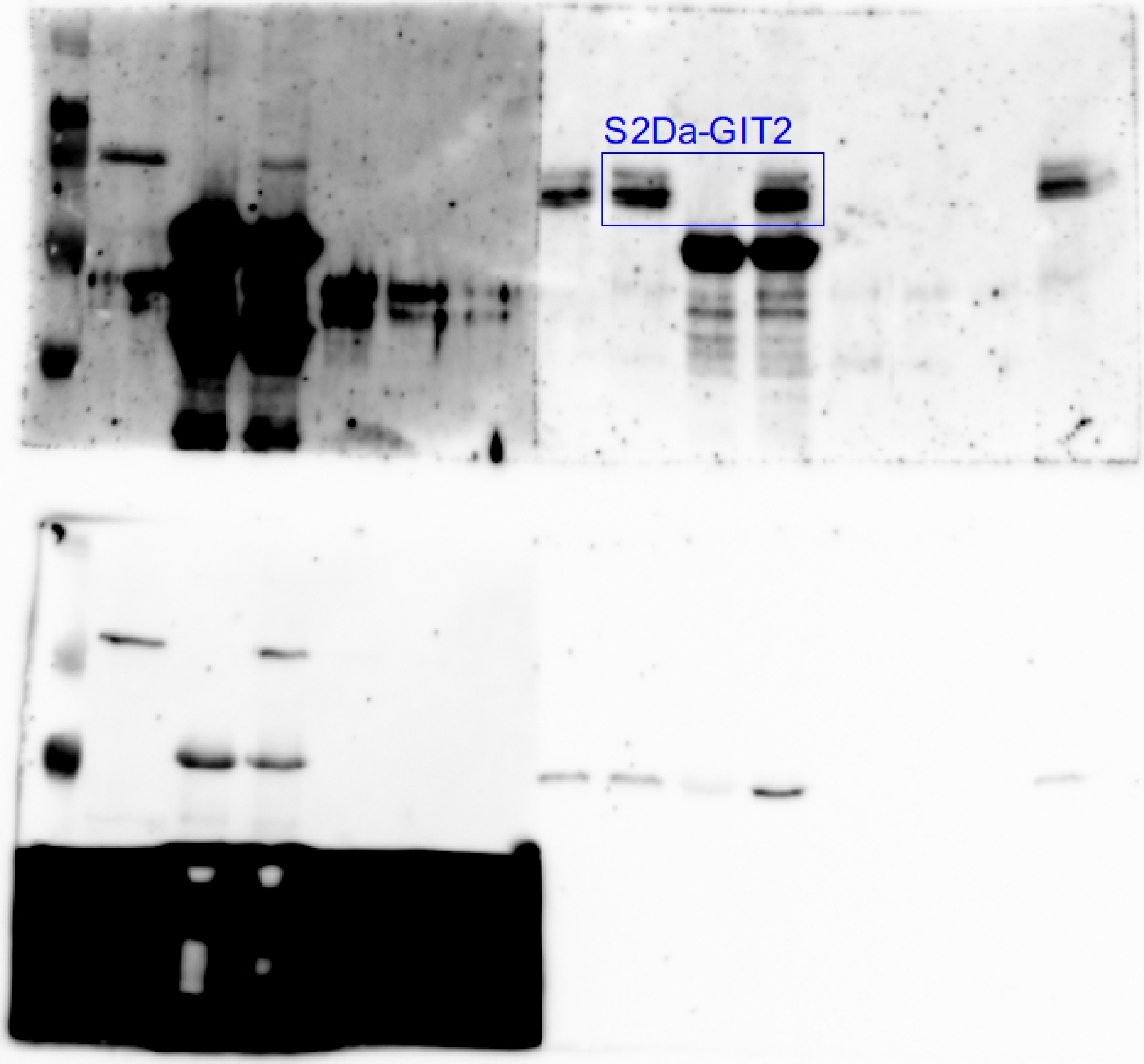
S2Da-GIT2

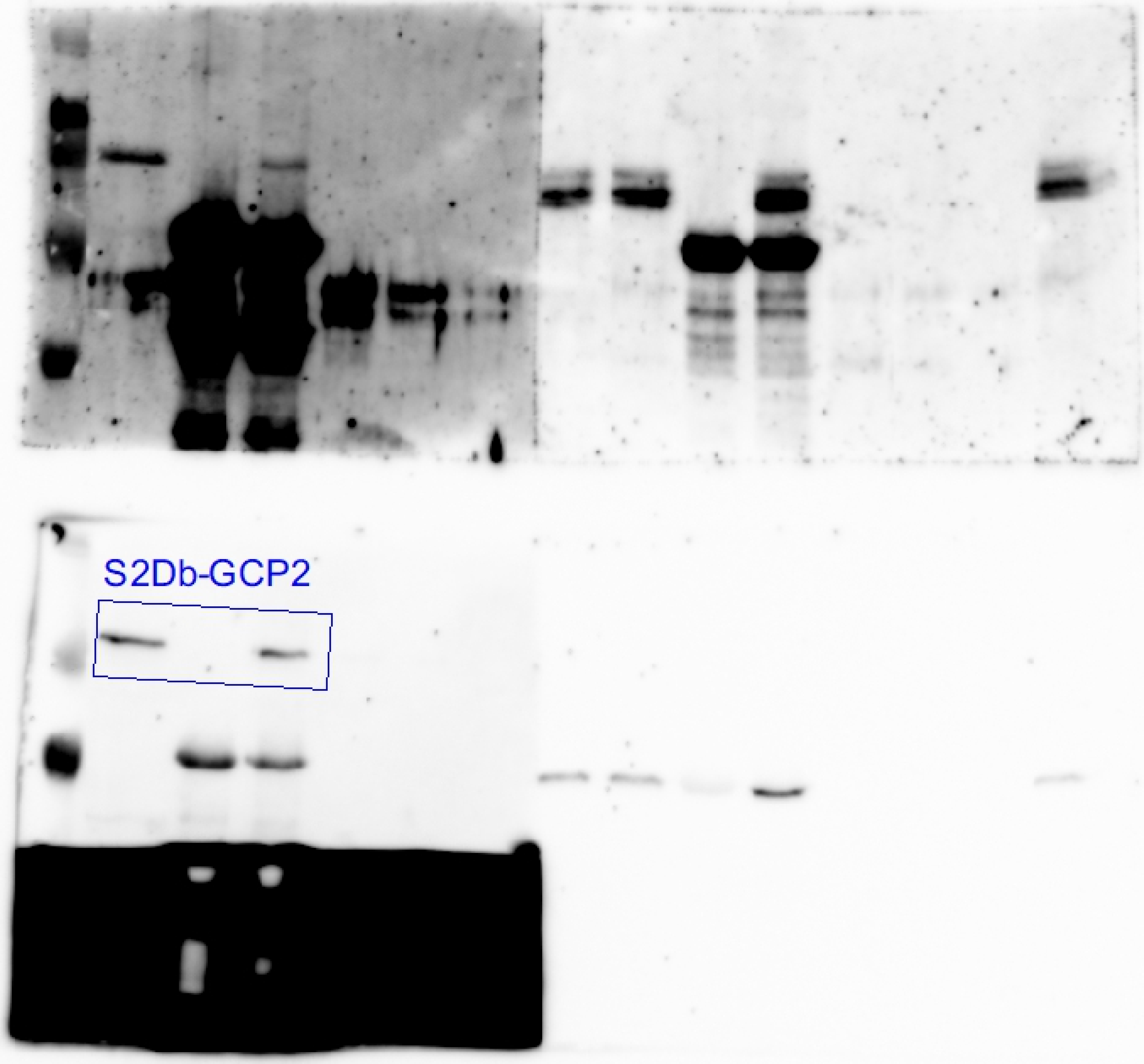
S2Db-GCP2

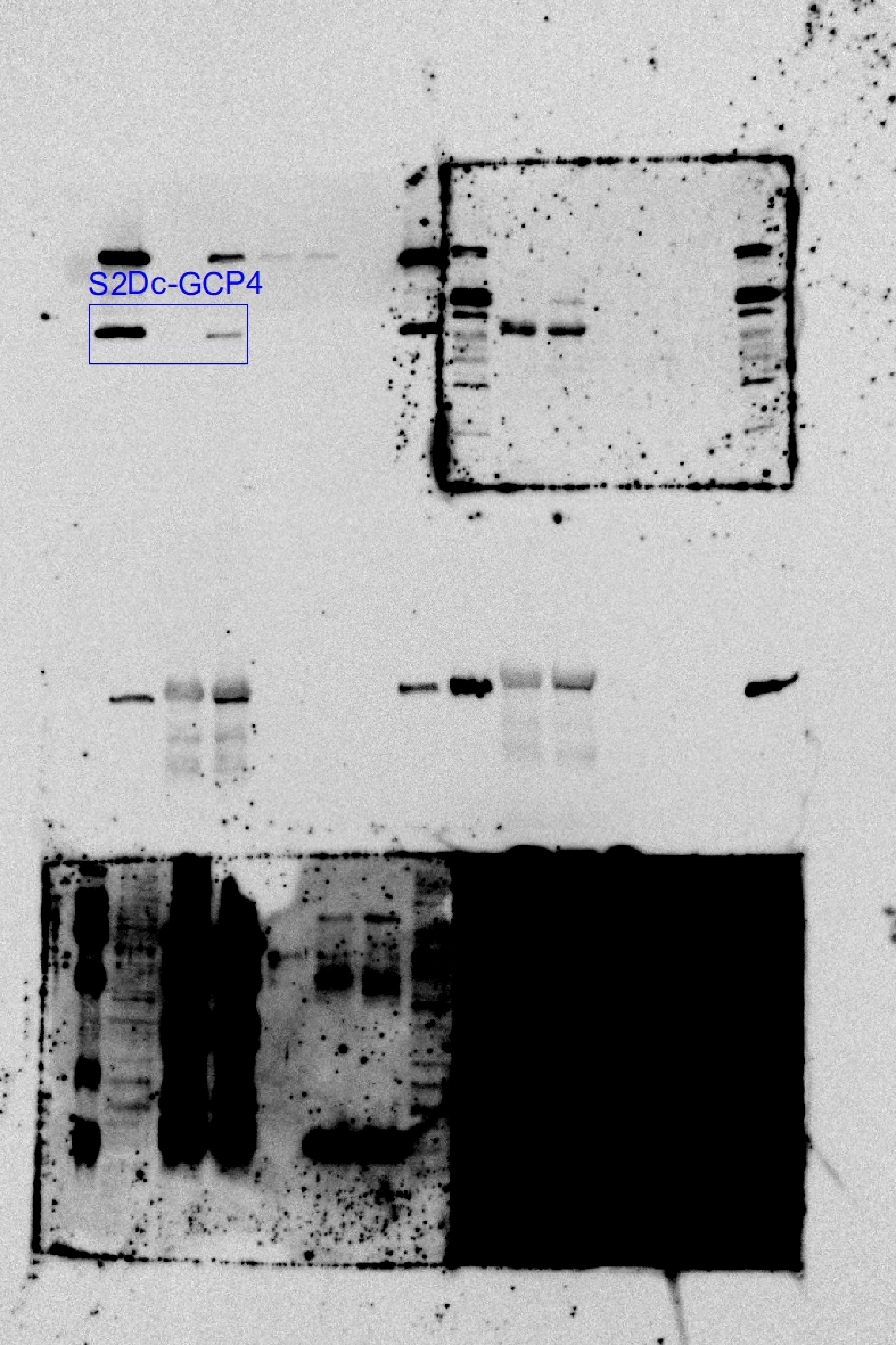
S2Dc-GCP4

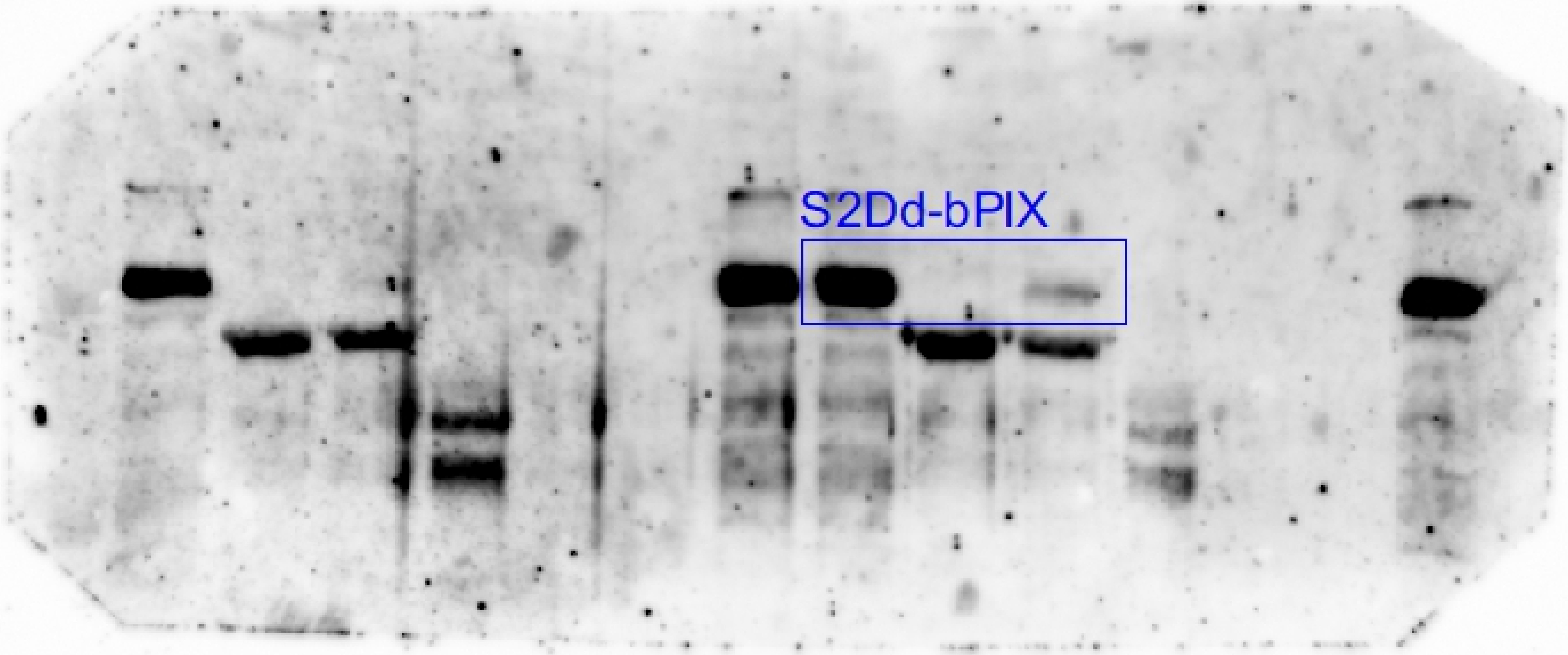
S2Dd-bPIX

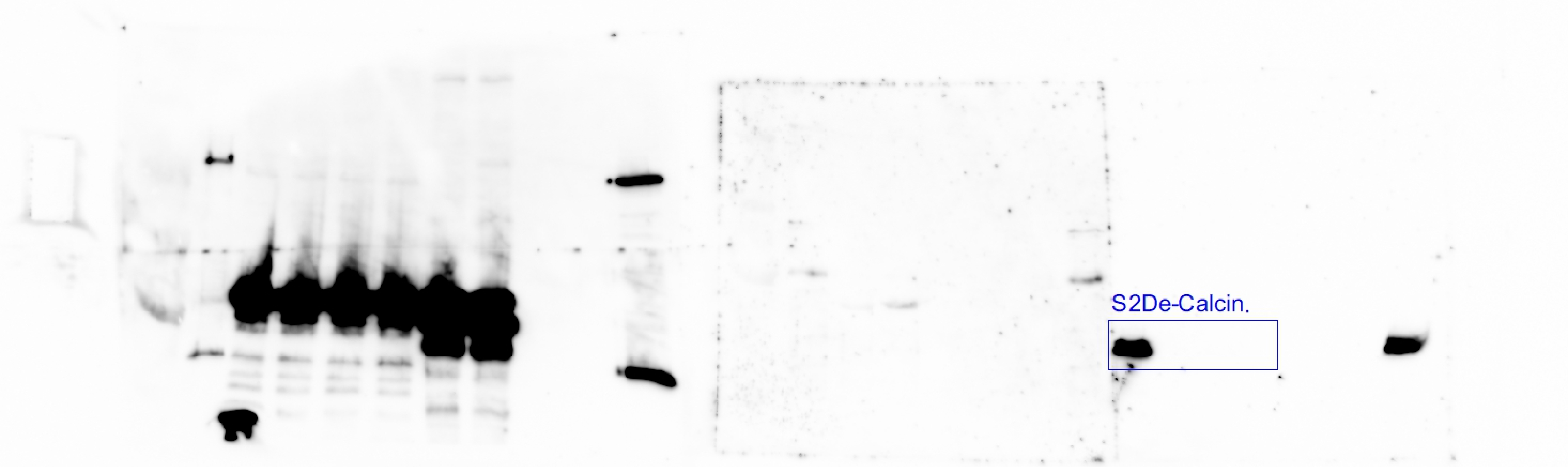
S2De-Calcin.

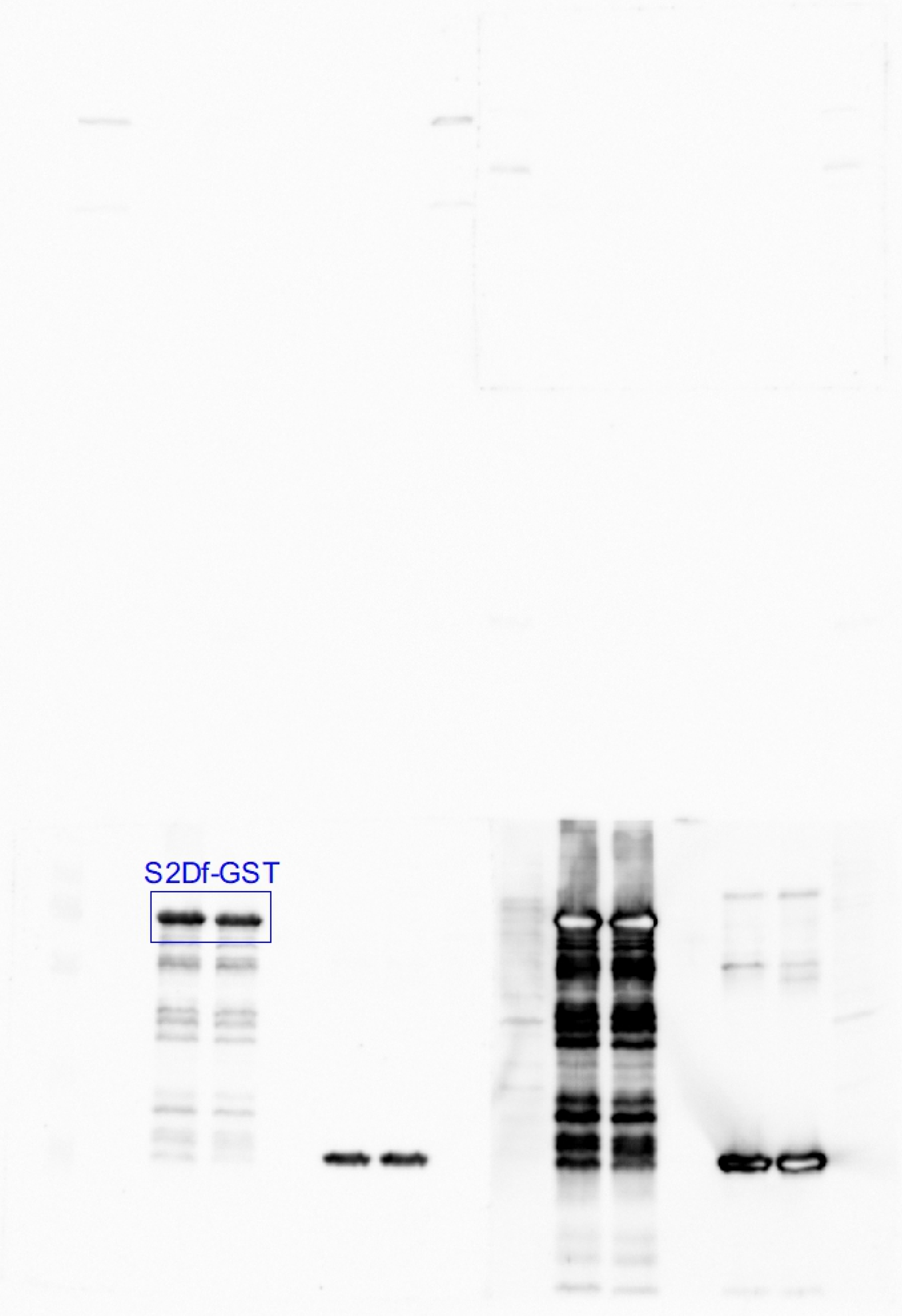
S2Df-GST

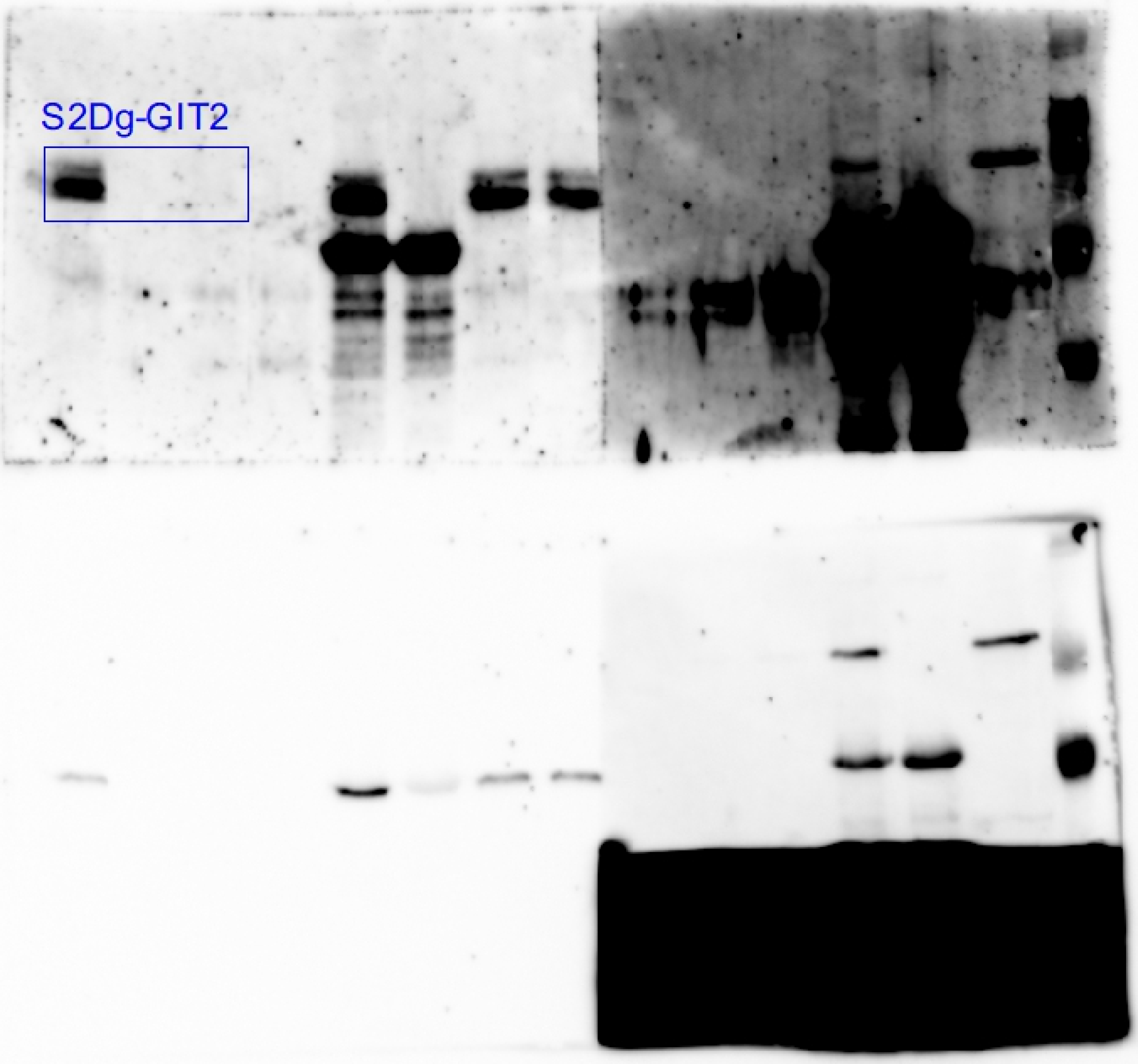
S2Dg-GIT2

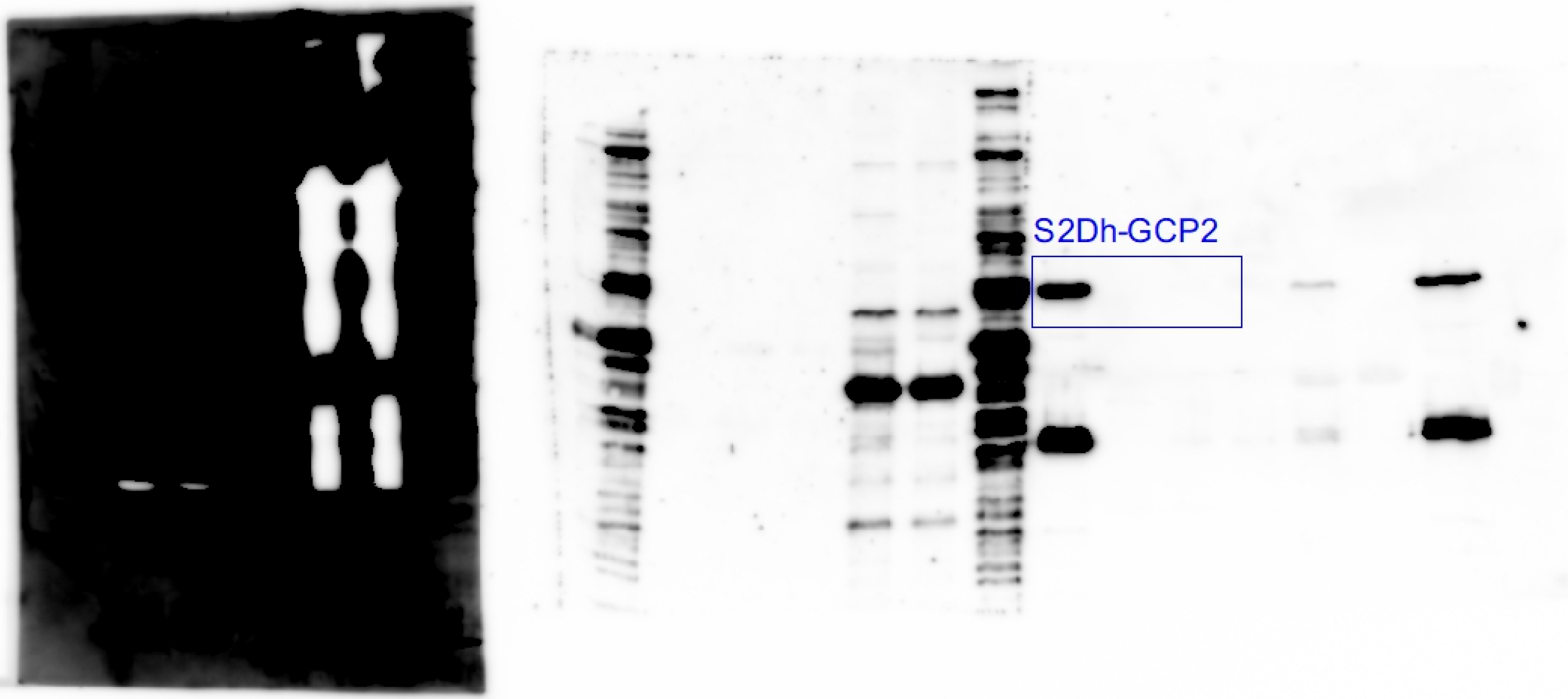
S2Dh-GCP2

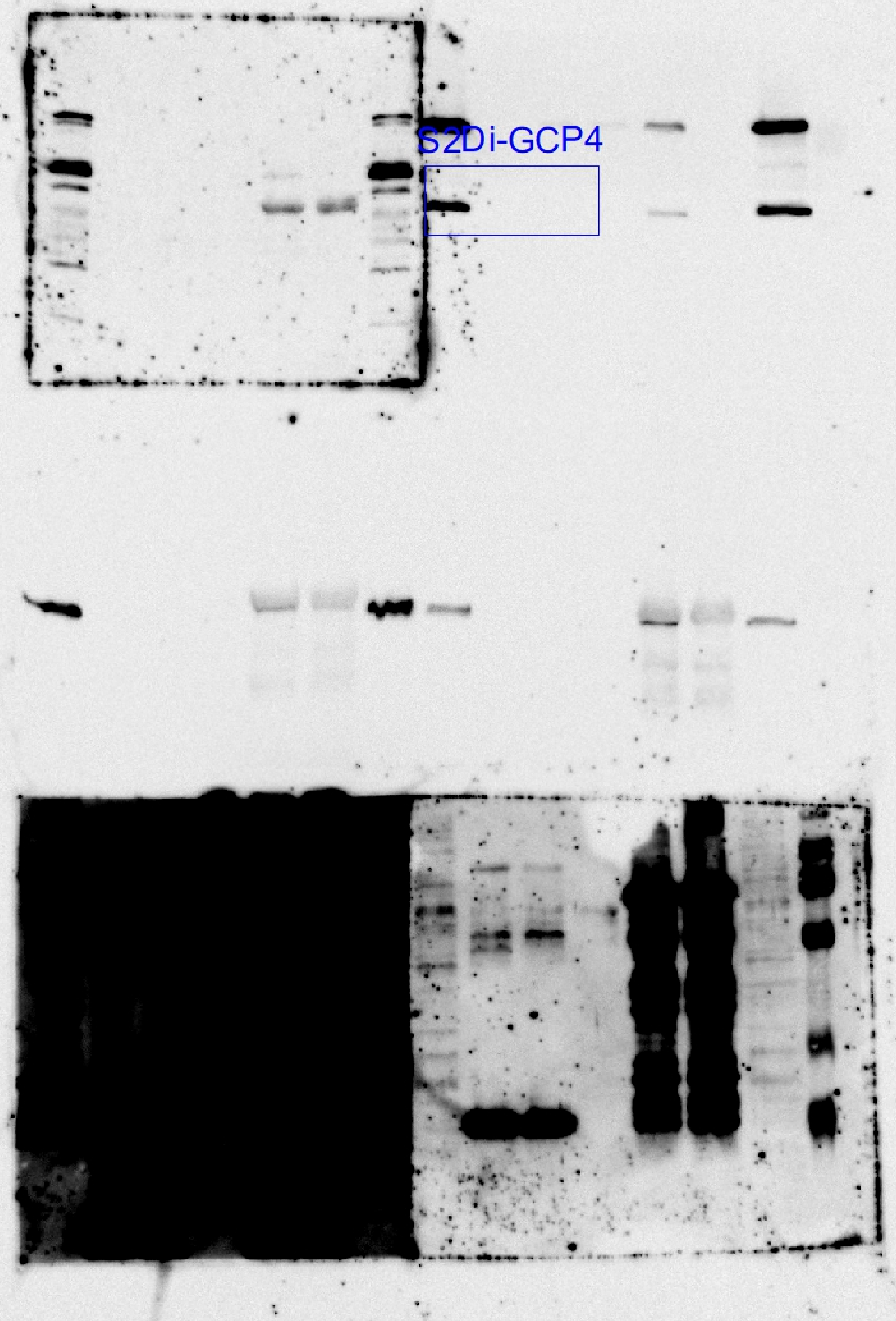
S2Di-GCP4

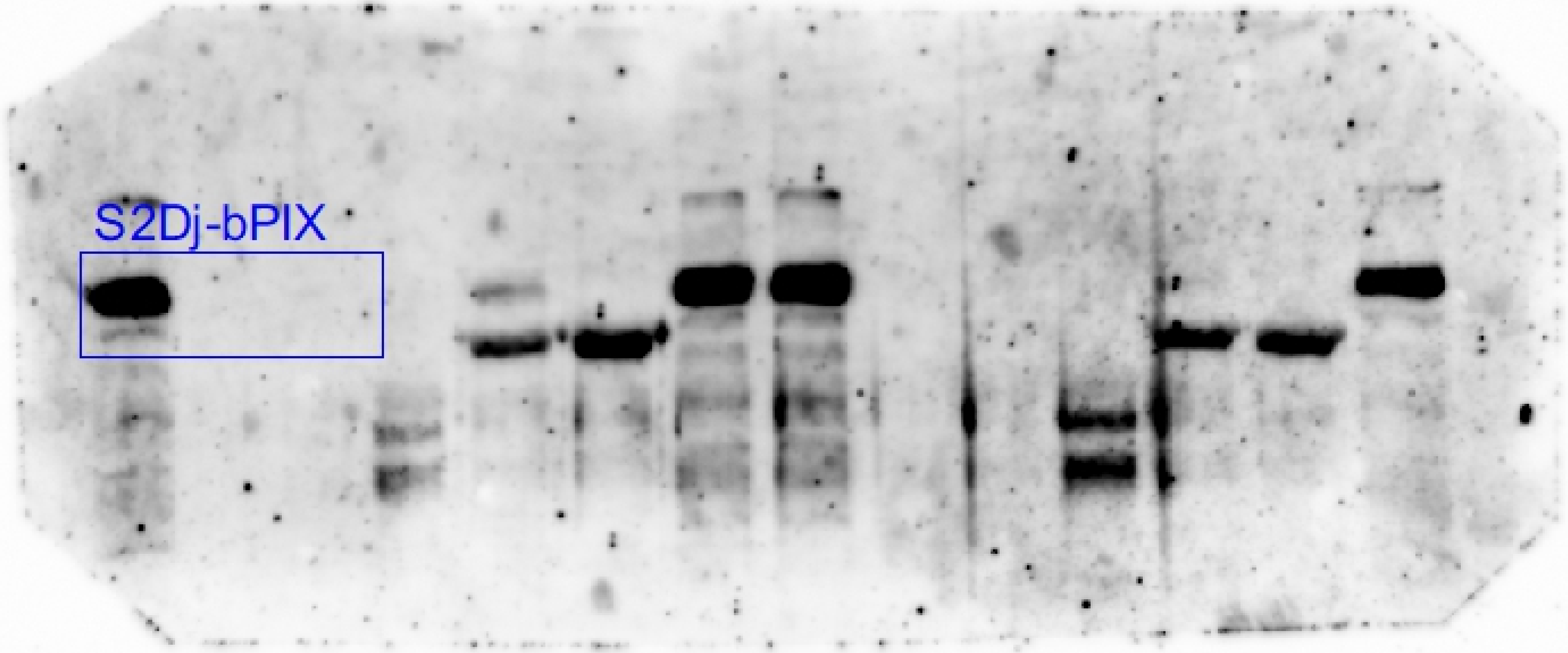
S2Dj-bPIX

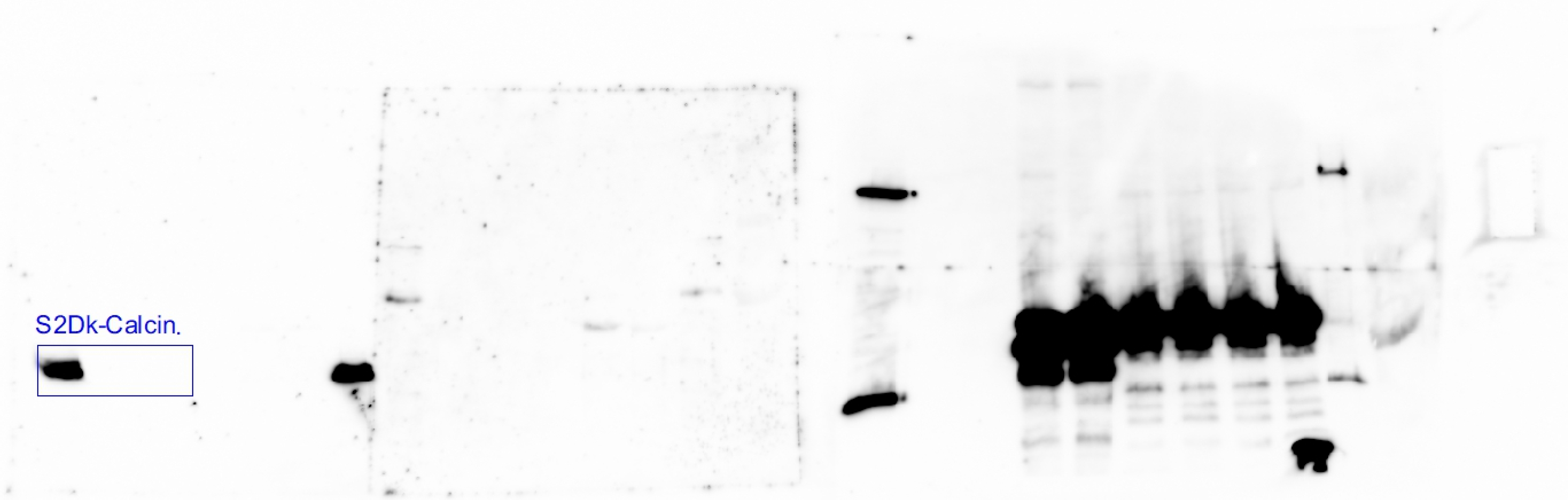
S2Dk-Calcin.

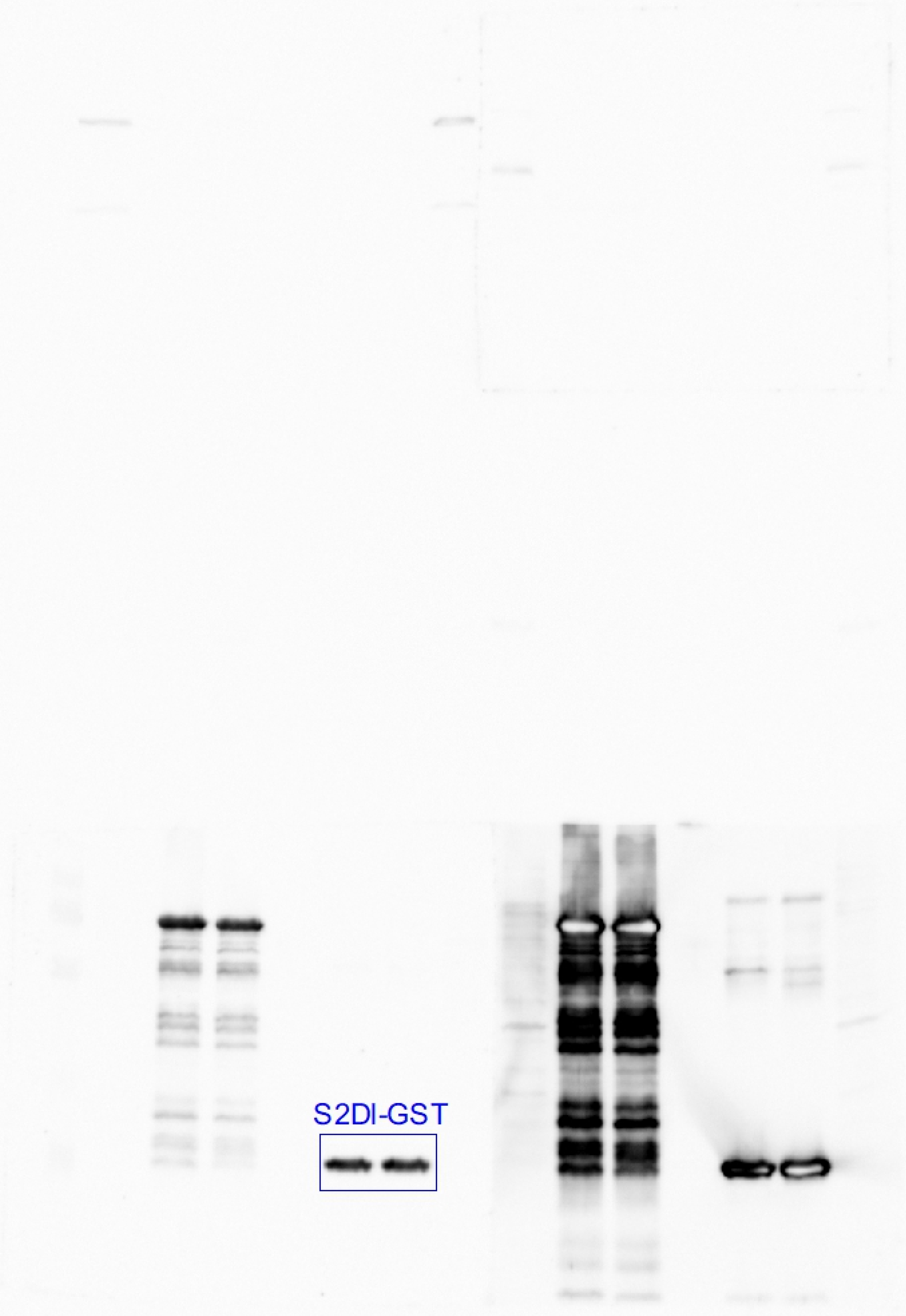
S2DI-GST
