# Supplementary material for: Regulation of microtubule nucleation in mouse bone marrow-derived mast cells by ARF GTPase-activating protein GIT2
Source: Front Immunol. 2024 Feb 2;15:1321321. doi: 10.3389/fimmu.2024.1321321 (PMC10870779; doi:10.3389/fimmu.2024.1321321)
Supplement: Supplementary file 3 [file Figure S3-originals.pdf › Data Sheet 8/Figure S3-originals.pdf]

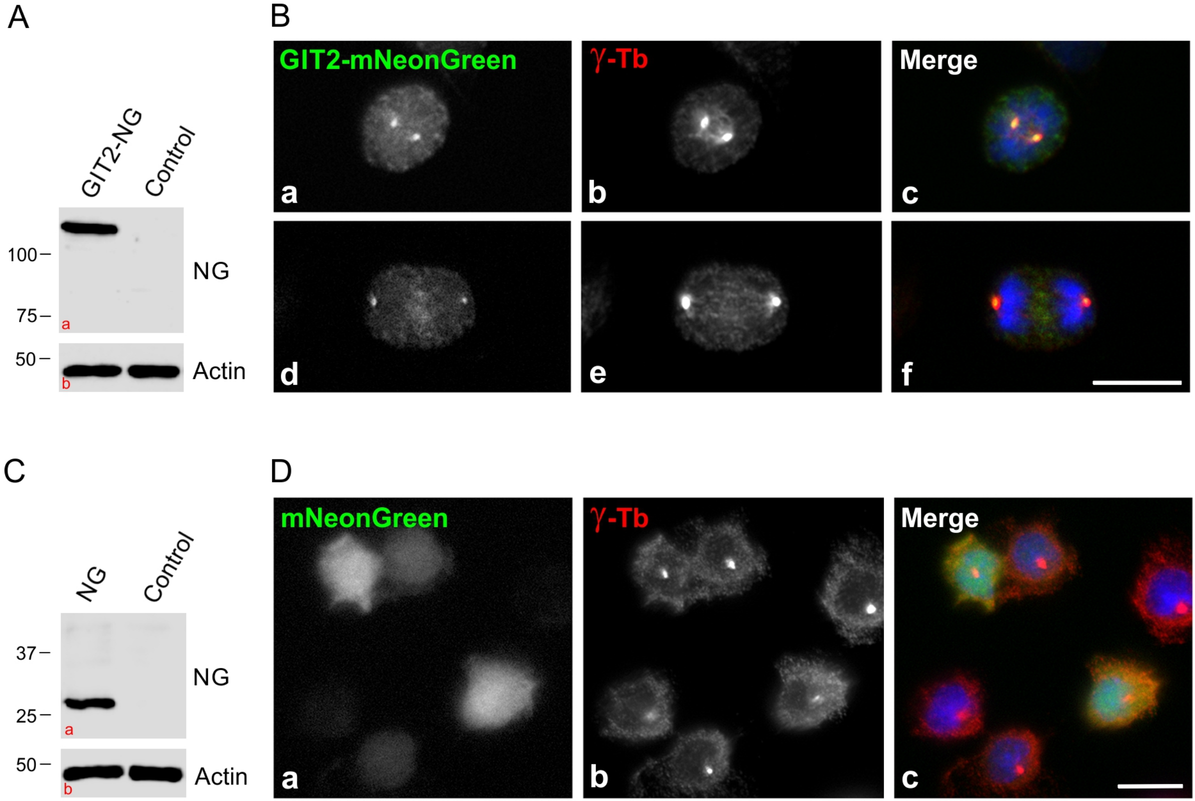

A
GIT2-NG
Control
100
75
50
NG
Actin
a
b
B
GIT2-mNeonGreen
γ-Tb
Merge
a
b
c
d
e
f
C
NG
Control
37
25
50
NG
Actin
a
b
D
mNeonGreen
γ-Tb
Merge
a
b
c

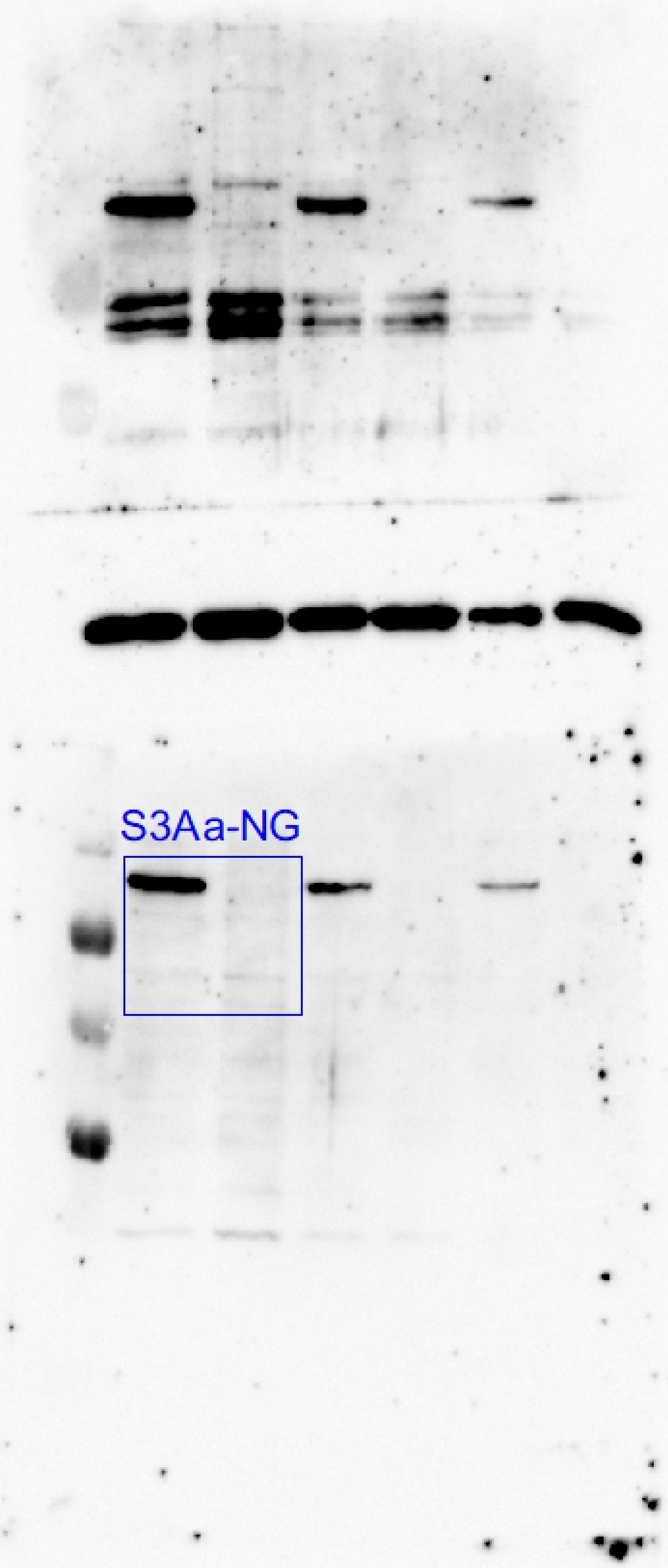

S3Aa-NG

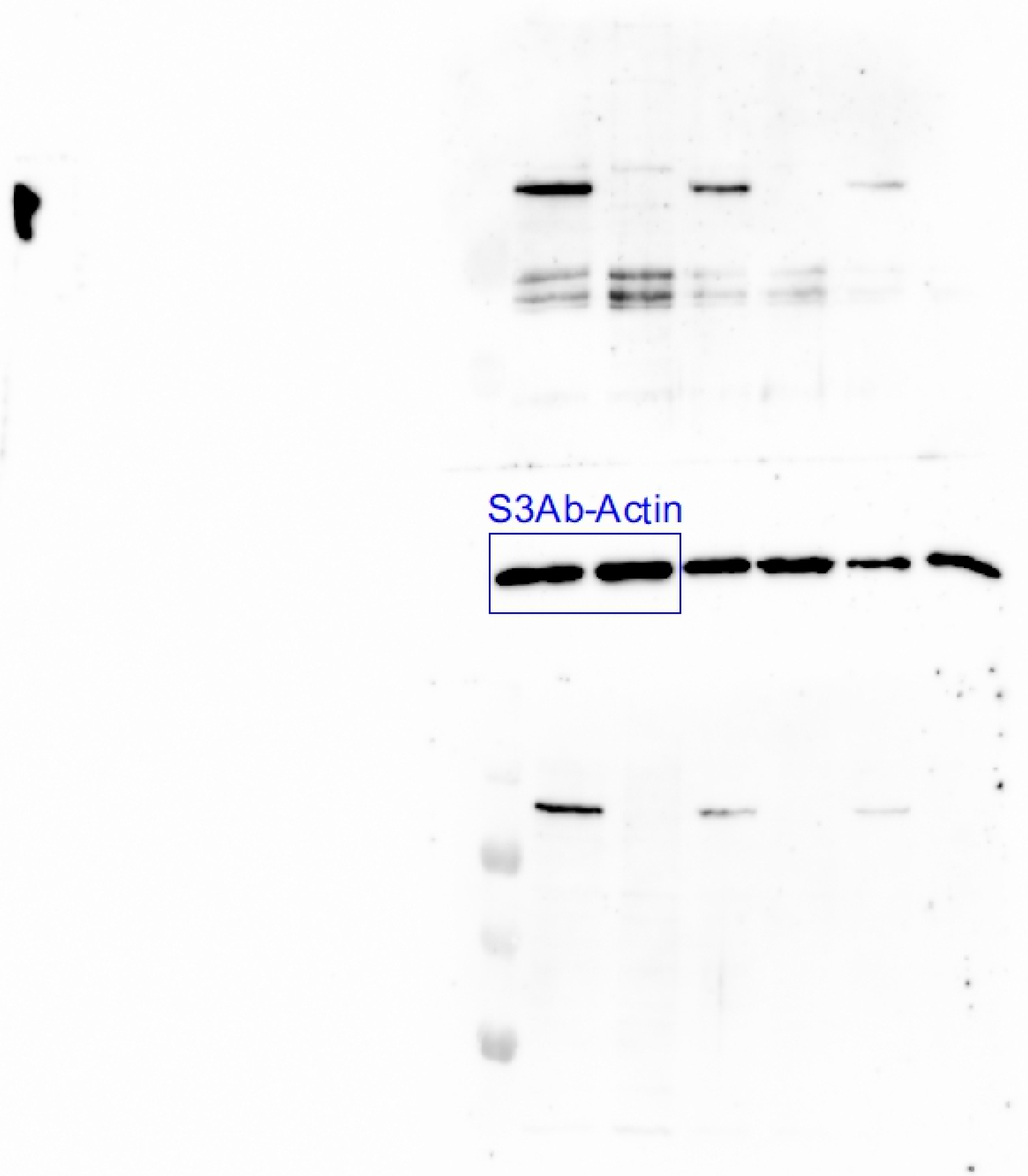
S3Ab-Actin

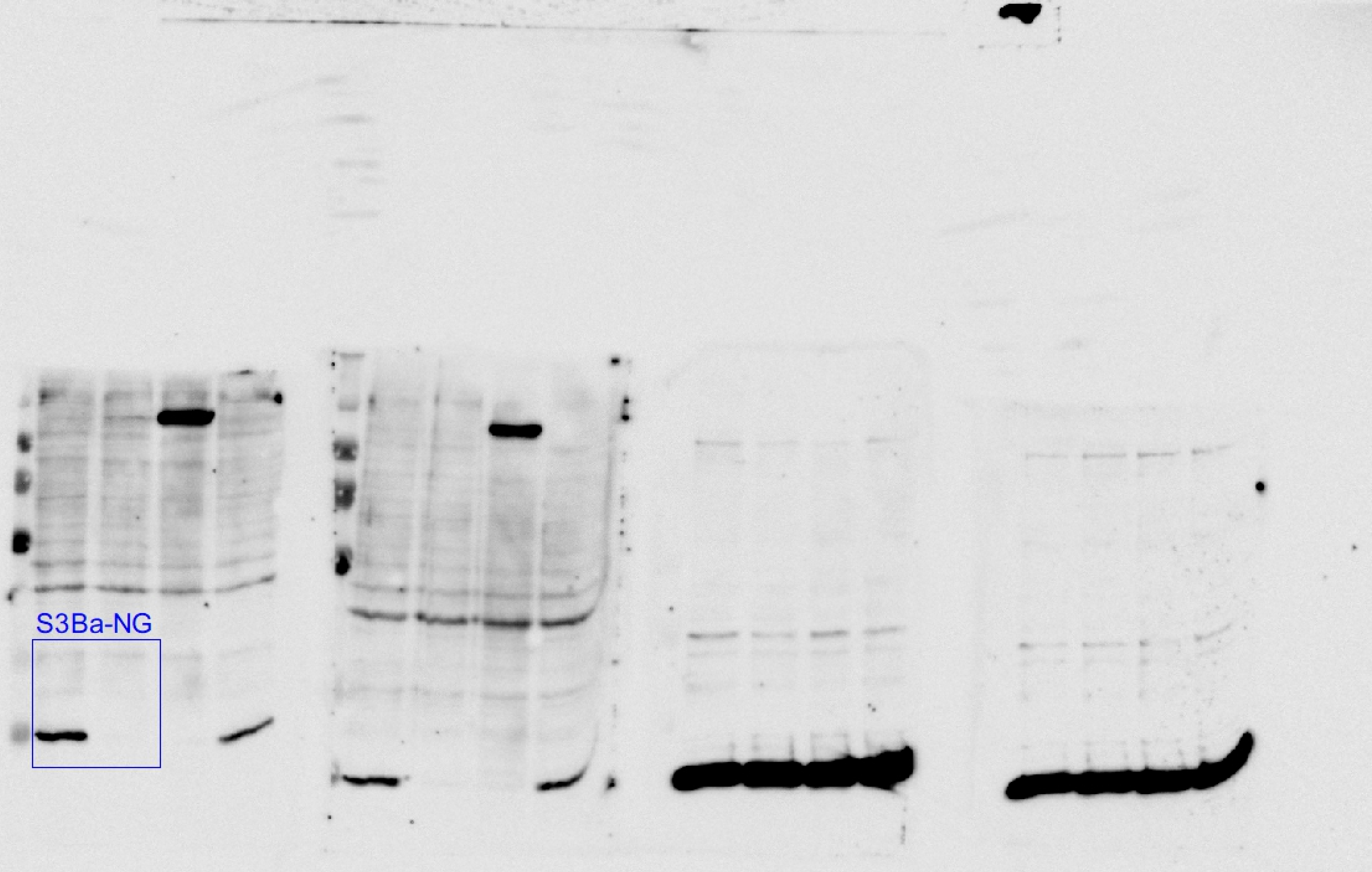
S3Ba-NG

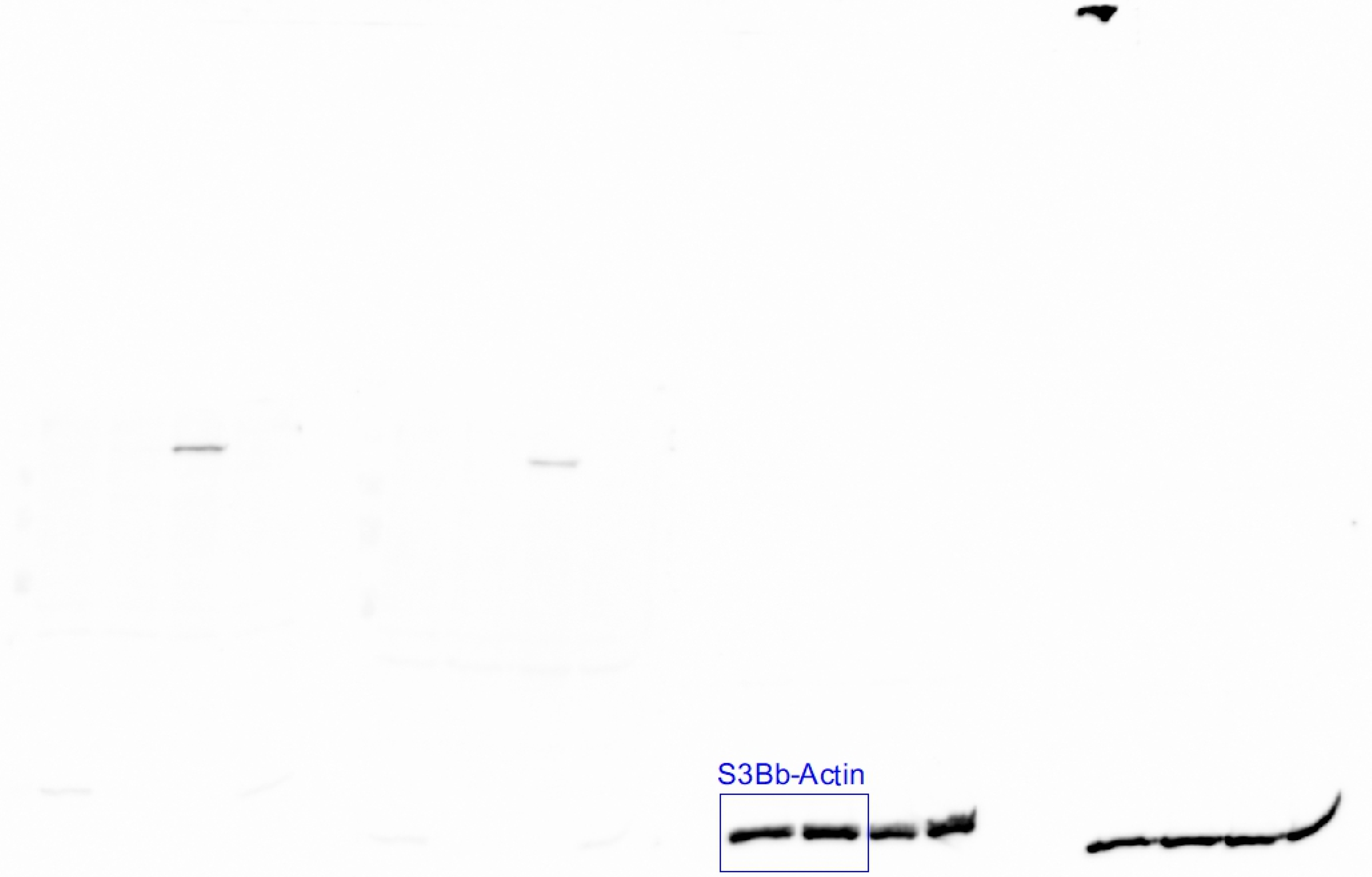
S3Bb-Actin
