# Supplementary material for: Regulation of microtubule nucleation in mouse bone marrow-derived mast cells by ARF GTPase-activating protein GIT2
Source: Front Immunol. 2024 Feb 2;15:1321321. doi: 10.3389/fimmu.2024.1321321 (PMC10870779; doi:10.3389/fimmu.2024.1321321)
Supplement: Supplementary file 3 [file Figure S5-originals.pdf › Data Sheet 8/Figure S5-originals.pdf]

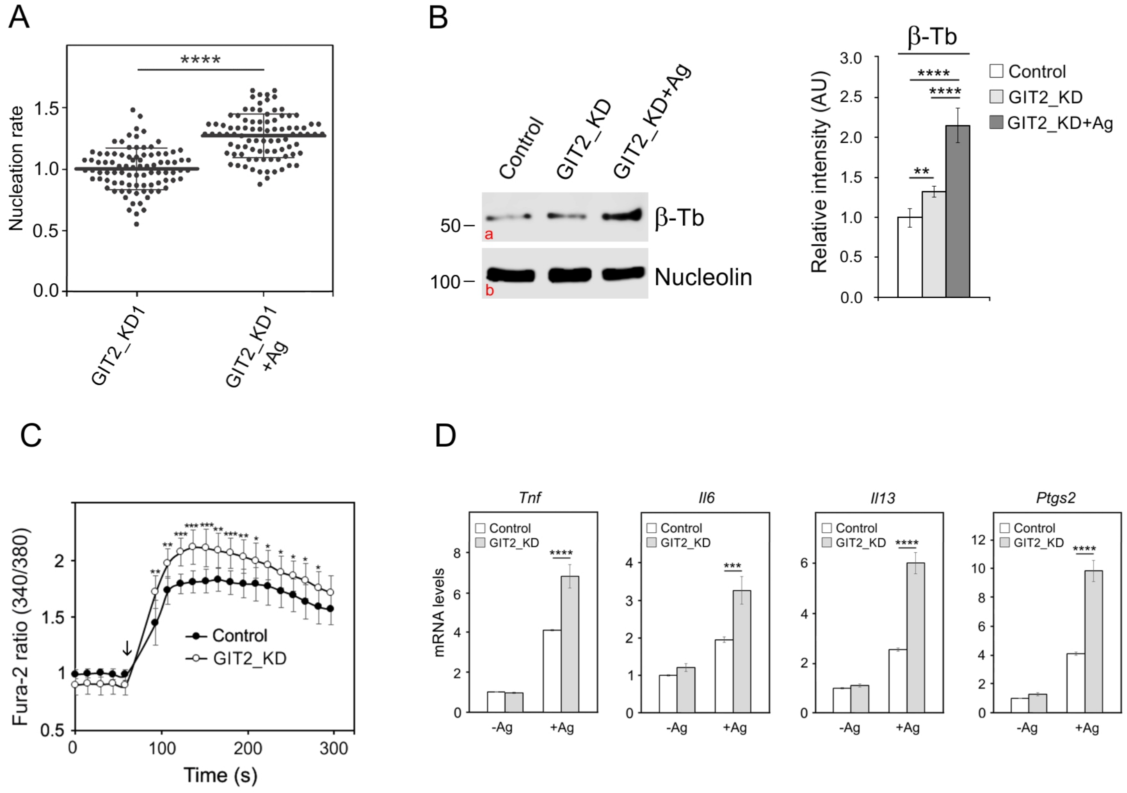

A
****
Nucleation rate
1.5
1.0
0.5
0.0
GIT2_KD1
GIT2_KD1 +Ag
B
Control
GIT2_KD
GIT2_KD+Ag
50 —
100 —
a
b
β-Tb
Nucleolin
β-Tb
Relative intensity (AU)
3.0
2.5
2.0
1.5
1.0
0.5
0.0
****
****
**
Control
GIT2_KD
GIT2_KD+Ag
C
Fura-2 ratio (340/380)
2
1.5
1
0.5
0
100
200
300
Time (s)
Control
GIT2_KD
D
Tnf
Il6
Il13
Ptgs2
mRNA levels
Control
GIT2_KD
-Ag
+Ag
****
***
****
****

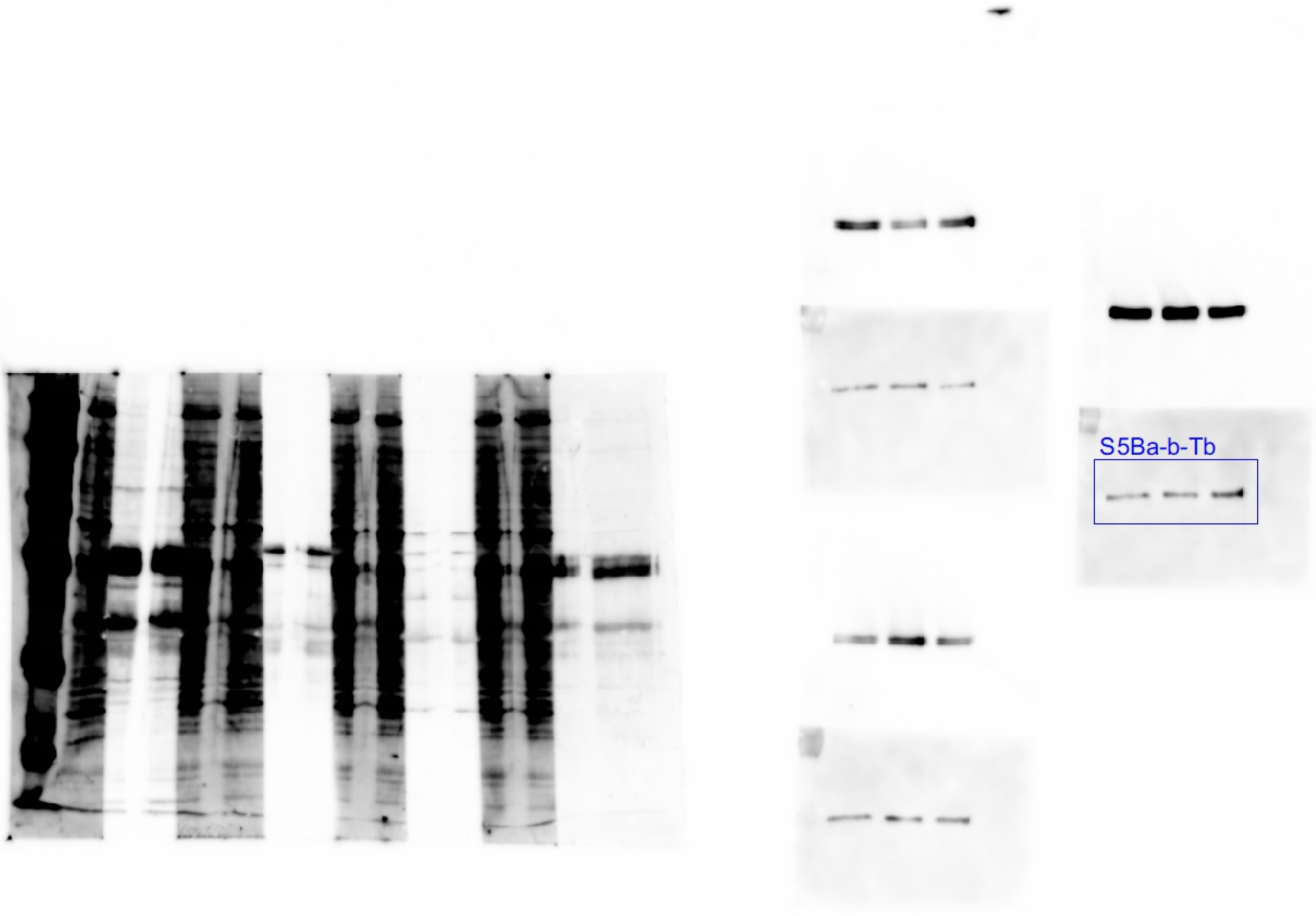
S5Ba-b-Tb

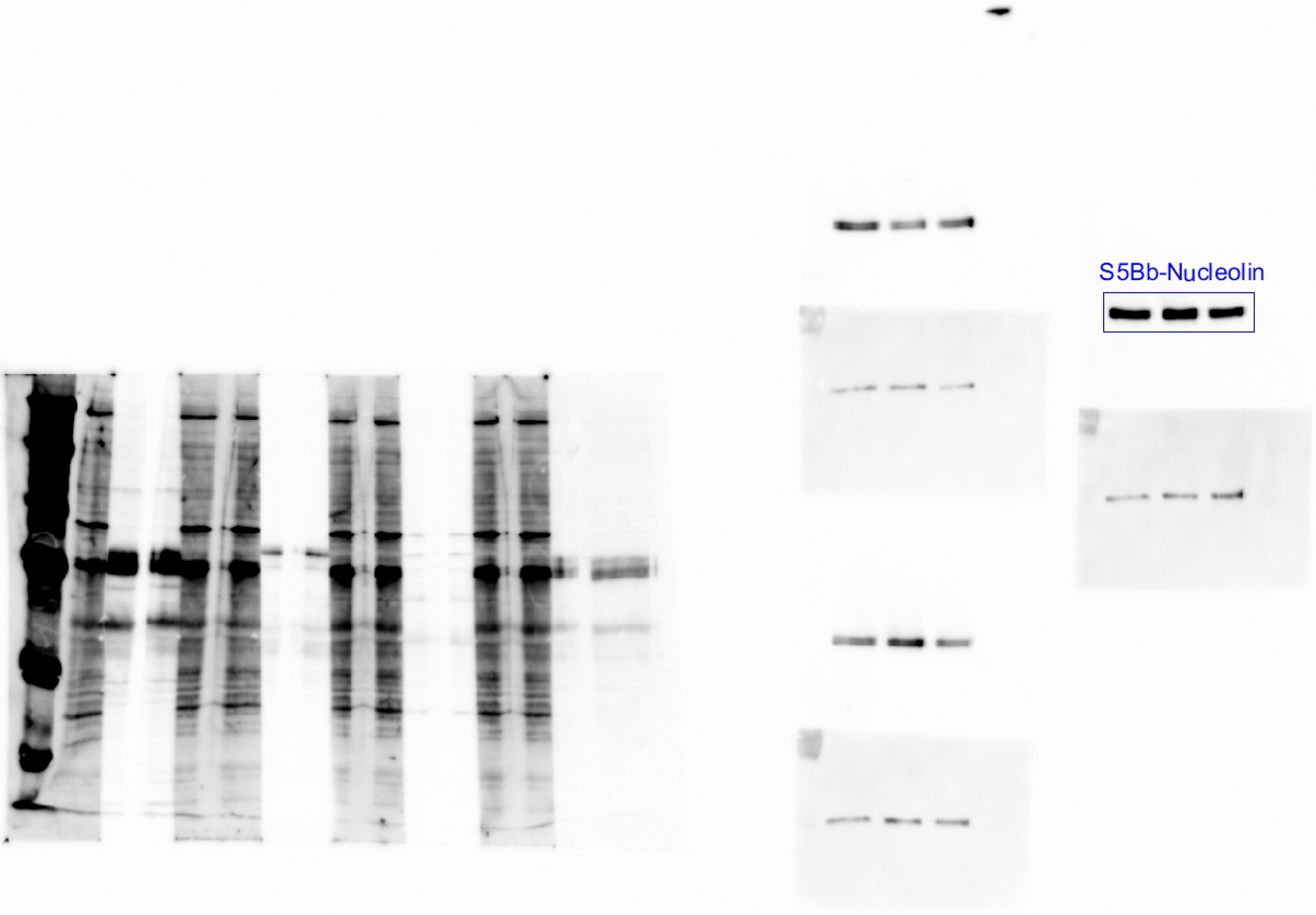
S5Bb-Nucleolin
