# Supplementary material for: Regulation of microtubule nucleation in mouse bone marrow-derived mast cells by ARF GTPase-activating protein GIT2
Source: Front Immunol. 2024 Feb 2;15:1321321. doi: 10.3389/fimmu.2024.1321321 (PMC10870779; doi:10.3389/fimmu.2024.1321321)

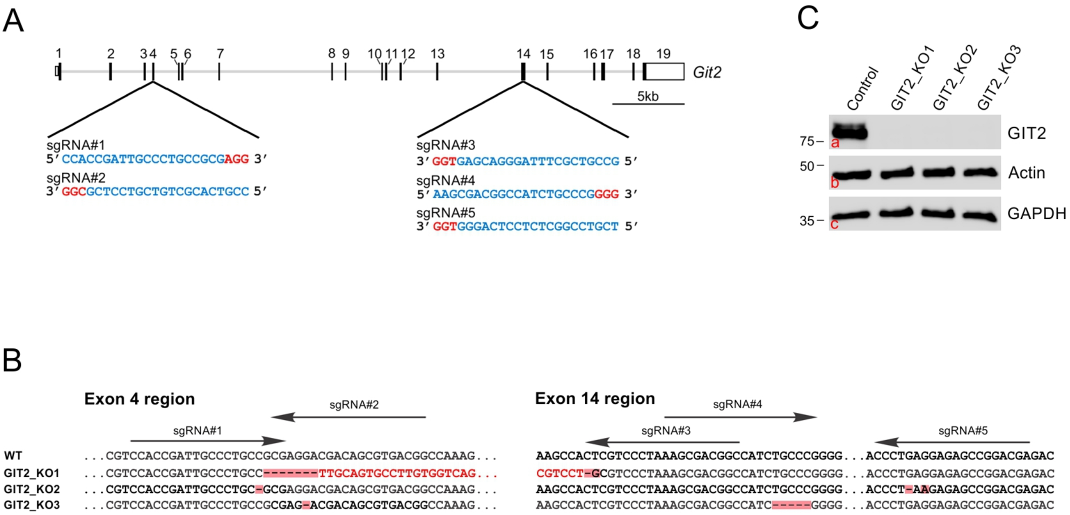
A
1 2 3 4 5 6 7 8 9 10 11 12 13 14 15 16 17 18 19
Git2
5kb
sgRNA#1
5′ CCACCGATTGCCCTGCCGCGAGG 3′
sgRNA#2
3′ GGCGCTCCTGCTGTCGCACTGCC 5′
sgRNA#3
3′ GGTGAGCAGGGATTTCGCTGCCG 5′
sgRNA#4
5′ AAGCGACGGCCATCTGCCCGGGG 3′
sgRNA#5
3′ GGTGGGACTCCTCTCGGCCTGCT 5′
C
Control
GIT2_KO1
GIT2_KO2
GIT2_KO3
75
50
35
a
b
c
GIT2
Actin
GAPDH
B
Exon 4 region
sgRNA#2
sgRNA#1
WT ...CGTCCACCGATTGCCCTGCCGCGAGGACGACAGCGTGACGGCCAAAG...
GIT2_KO1 ...CGTCCACCGATTGCCCTGCC-------TTGCAGTGCCTTGTGGTCAG...
GIT2_KO2 ...CGTCCACCGATTGCCCTGC-GCGAGGACGACAGCGTGACGGCCAAAG...
GIT2_KO3 ...CGTCCACCGATTGCCCTGCCGCGAG-ACGACAGCGTGACGGCCAAAG...
Exon 14 region
sgRNA#4
sgRNA#3
sgRNA#5
AAGCCACTCGTCCCTAAAGCGACGGCCATCTGCCCGGGG...ACCCTGAGGAGAGCCGGACGAGAC
CGTCCT-GCGTCCCTAAAGCGACGGCCATCTGCCCGGGG...ACCCTGAGGAGAGCCGGACGAGAC
AAGCCACTCGTCCCTAAAGCGACGGCCATCTGCCCGGGG...ACCCT-AAGAGAGCCGGACGAGAC
AAGCCACTCGTCCCTAAAGCGACGGCCATC-----GGGG...ACCCTGAGGAGAGCCGGACGAGAC

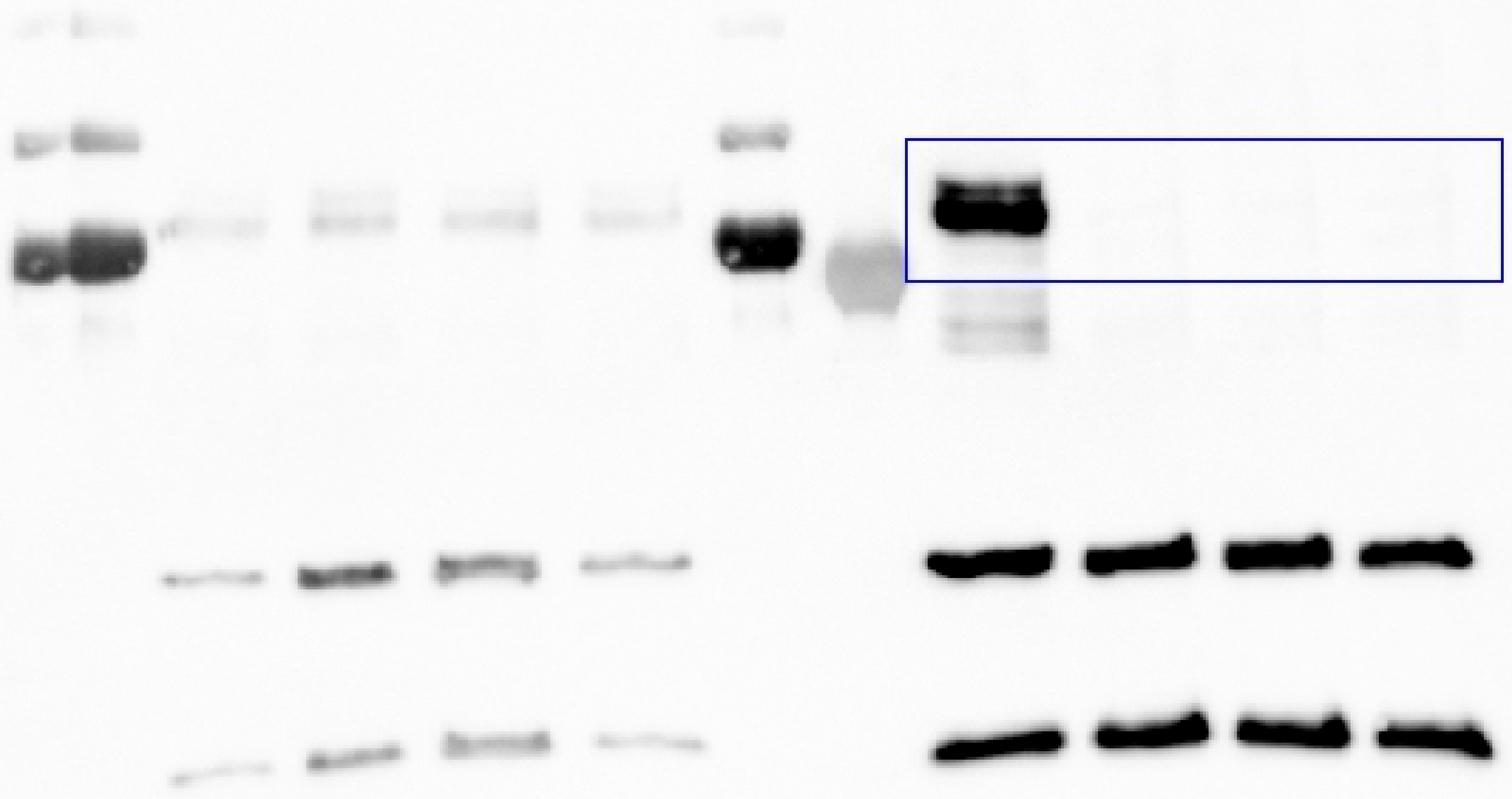

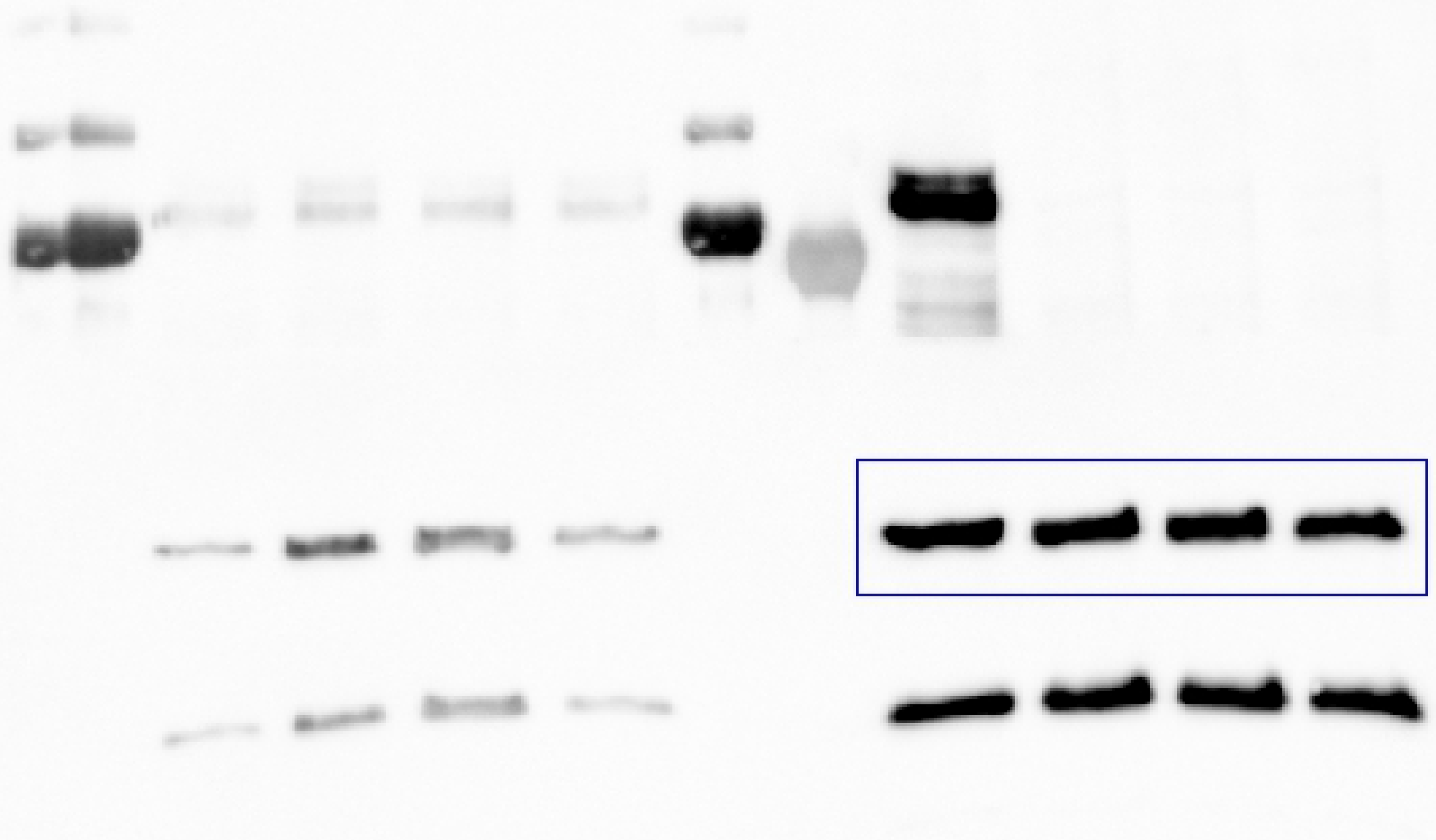

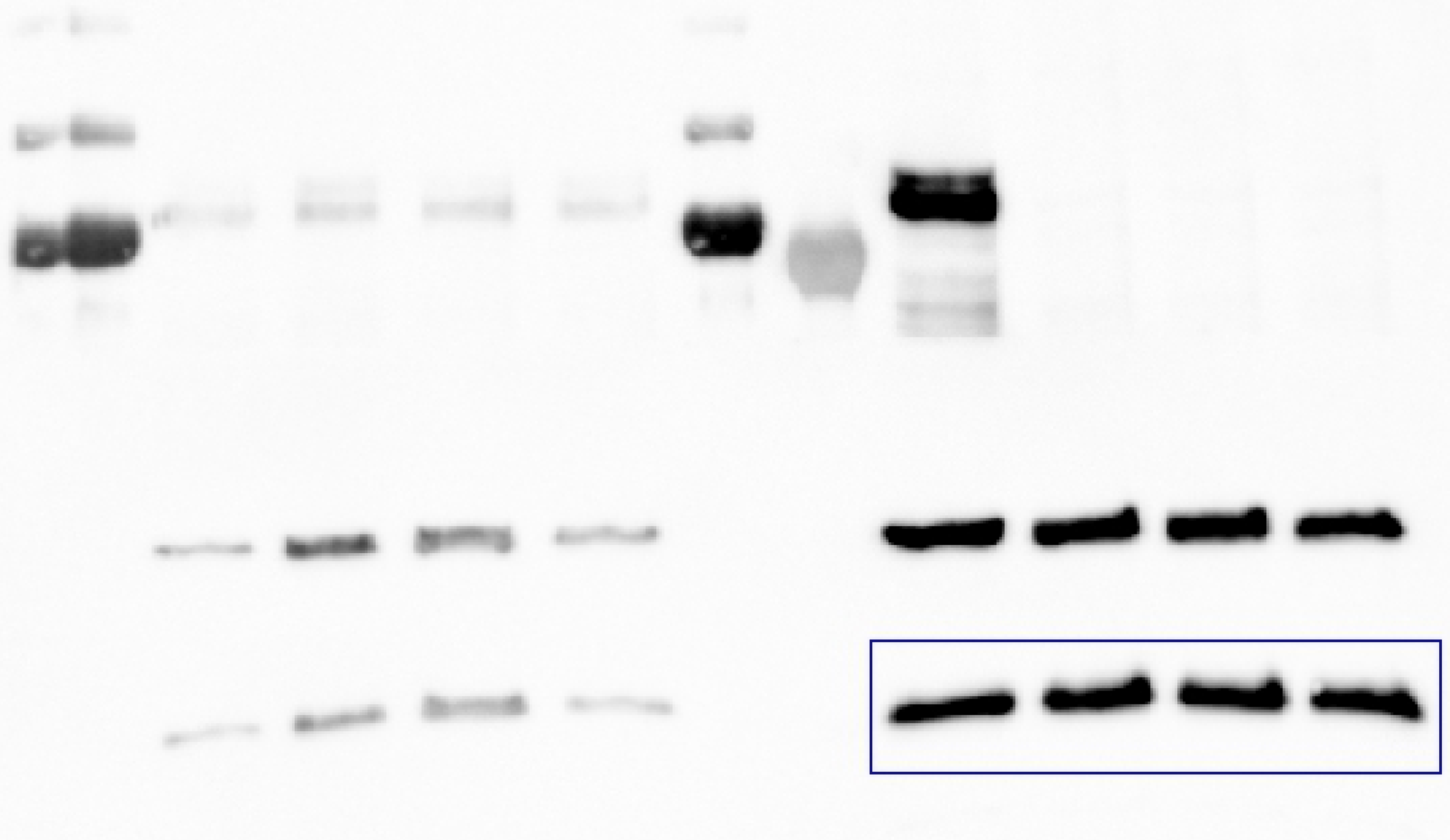

Supplement: Supplementary file 3 [file Figure S6-originals_v2.pdf › Data Sheet 8/Figure S6-originals_v2.pdf]
