# Supplementary material for: Regulation of microtubule nucleation in mouse bone marrow-derived mast cells by ARF GTPase-activating protein GIT2
Source: Front Immunol. 2024 Feb 2;15:1321321. doi: 10.3389/fimmu.2024.1321321 (PMC10870779; doi:10.3389/fimmu.2024.1321321)
Supplement: Supplementary file 3 [file Figure S7-originals.pdf › Data Sheet 8/Figure S7-originals.pdf]

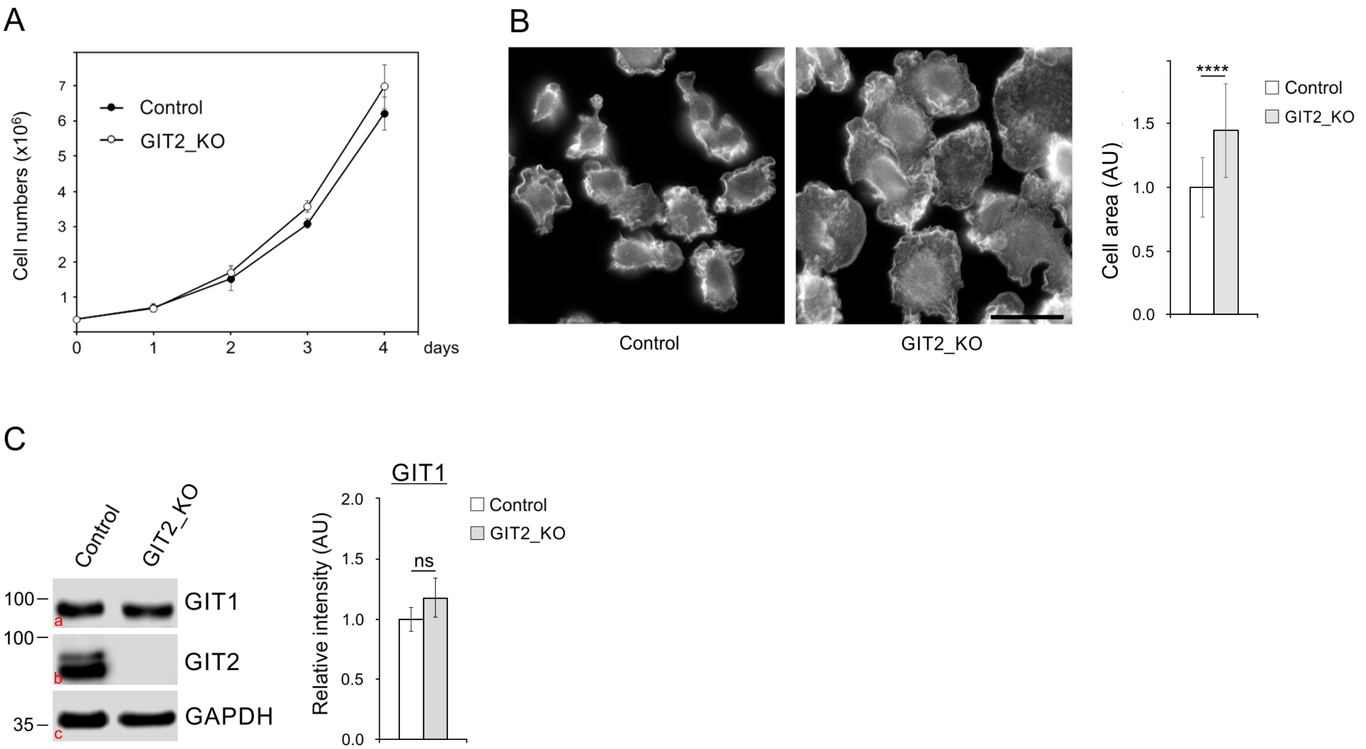

A
Cell numbers (x10^6)
0
1
2
3
4
5
6
7
days
Control
GIT2_KO
B
Control
GIT2_KO
Cell area (AU)
0.0
0.5
1.0
1.5
****
Control
GIT2_KO
C
Control
GIT2_KO
100
100
35
GIT1
GIT2
GAPDH
a
b
c
GIT1
Relative intensity (AU)
0.0
0.5
1.0
1.5
2.0
ns
Control
GIT2_KO

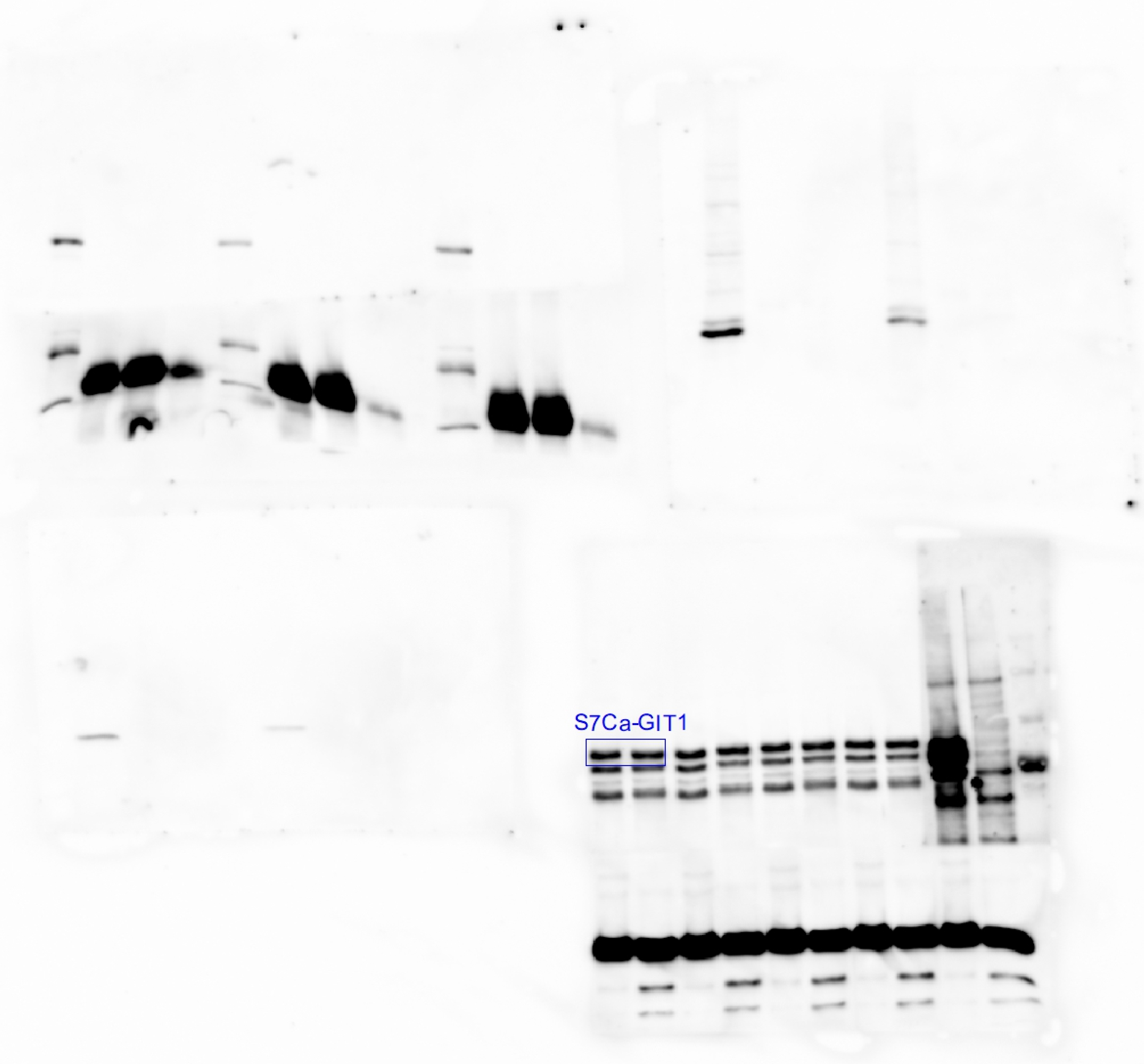
S7Ca-GIT1

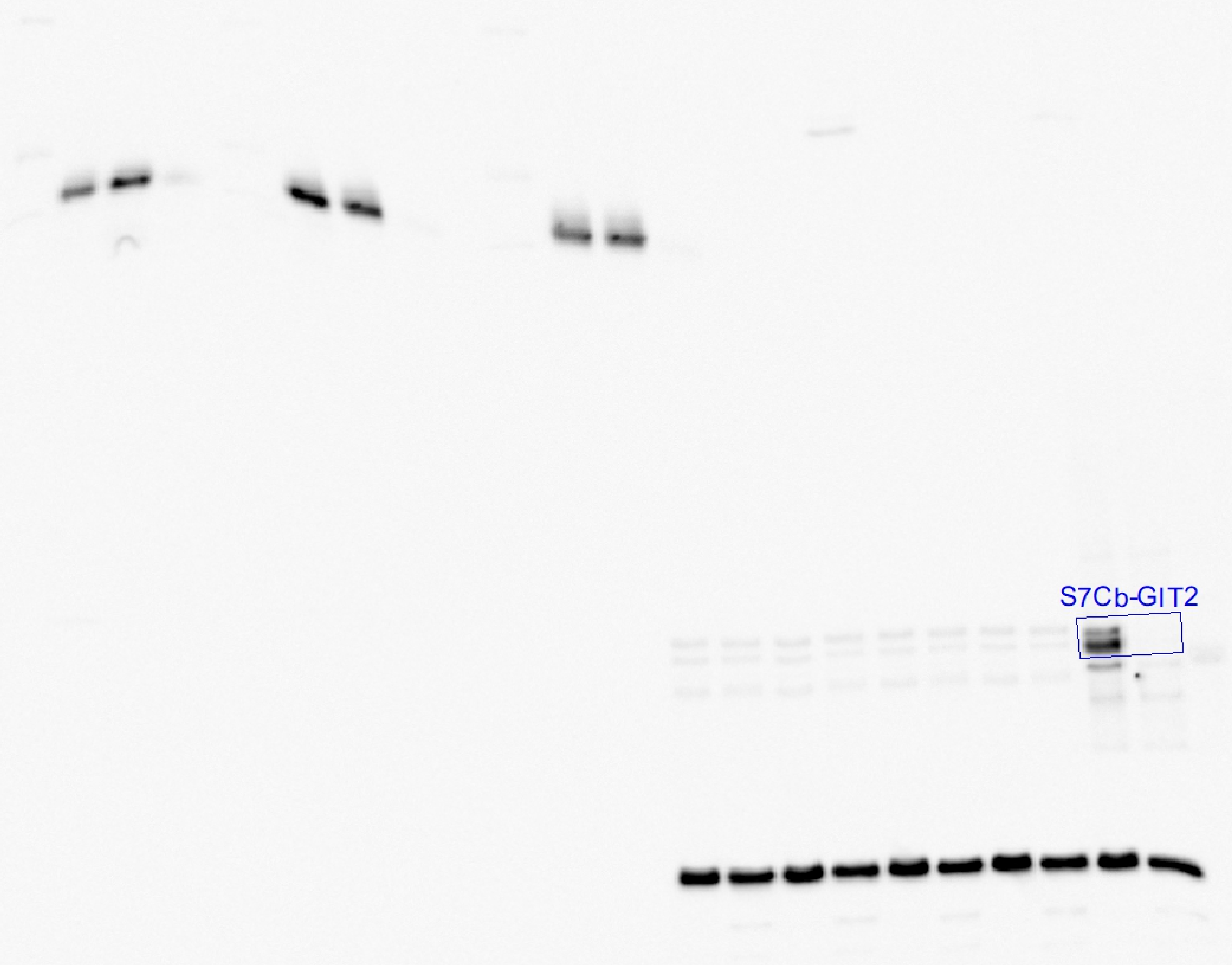
S7Cb-GIT2

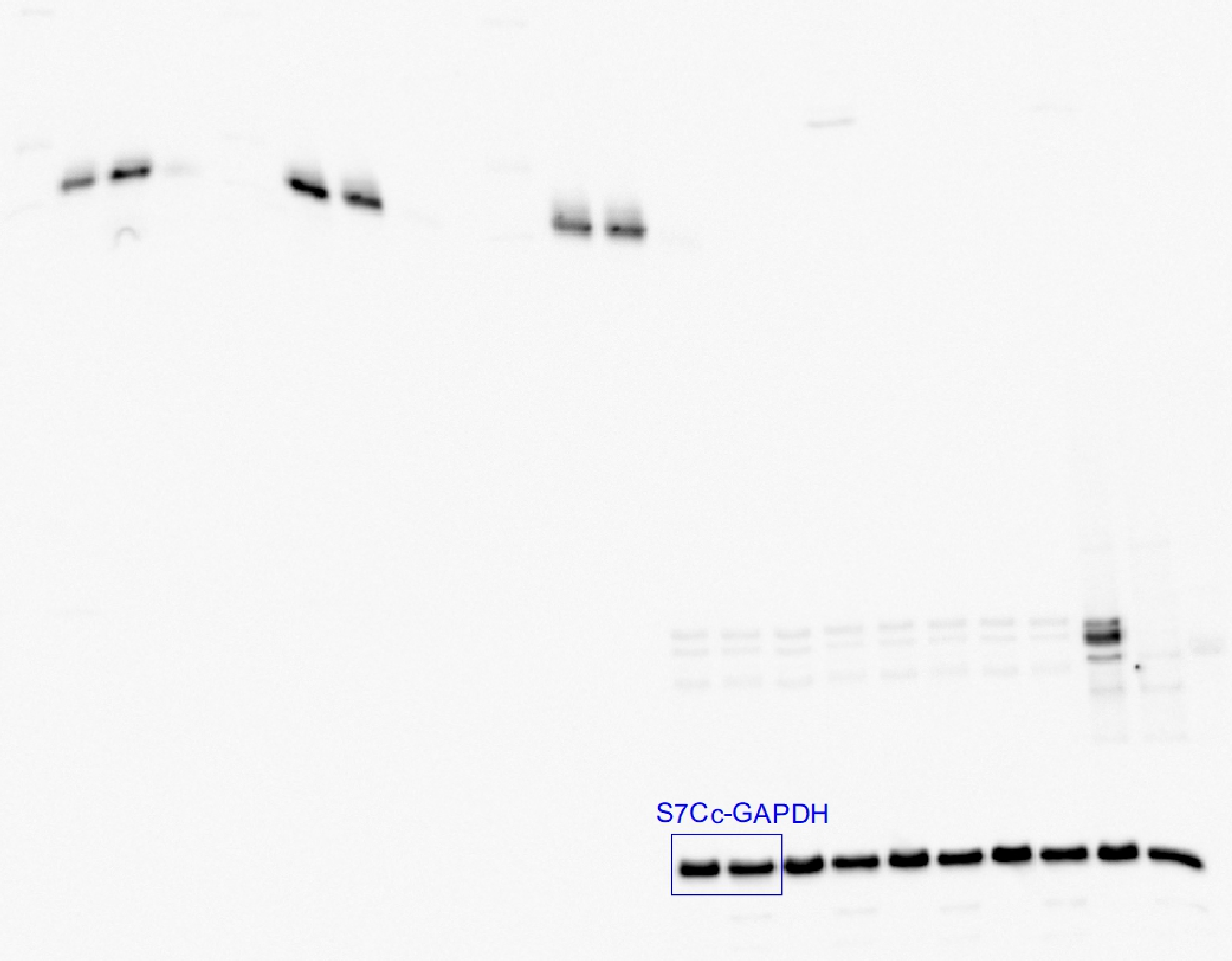
S7Cc-GAPDH
