# Supplementary material for: Regulation of microtubule nucleation in mouse bone marrow-derived mast cells by ARF GTPase-activating protein GIT2
Source: Front Immunol. 2024 Feb 2;15:1321321. doi: 10.3389/fimmu.2024.1321321 (PMC10870779; doi:10.3389/fimmu.2024.1321321)
Supplement: Supplementary file 3 [file Figure S8-originals.pdf › Data Sheet 8/Figure S8-originals.pdf]

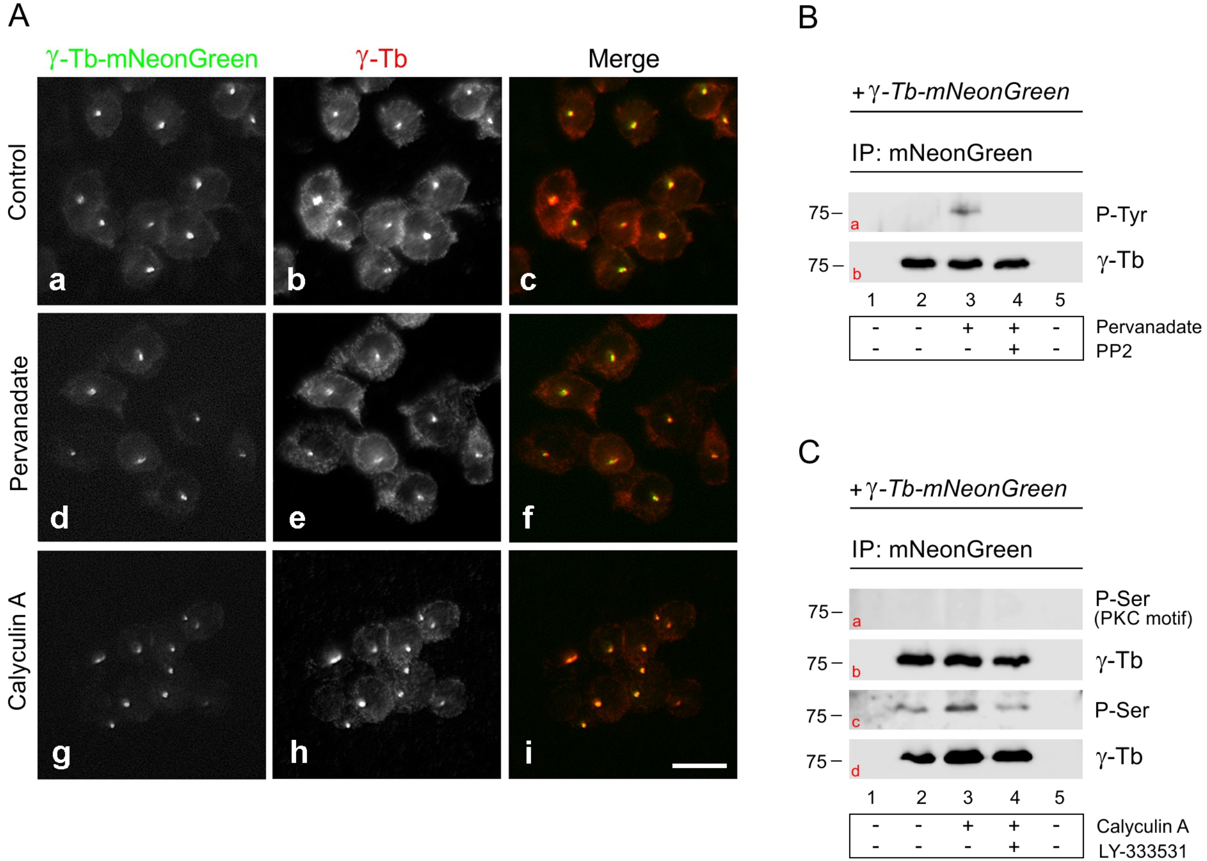
A
γ-Tb-mNeonGreen
γ-Tb
Merge
Control
Pervanadate
Calyculin A
a
b
c
d
e
f
g
h
i
B
+γ-Tb-mNeonGreen
IP: mNeonGreen
75
P-Tyr
75
γ-Tb
1 2 3 4 5
Pervanadate - - + + -
PP2 - - - + -
C
+γ-Tb-mNeonGreen
IP: mNeonGreen
75
P-Ser (PKC motif)
75
γ-Tb
75
P-Ser
75
γ-Tb
1 2 3 4 5
Calyculin A - - + + -
LY-333531 - - - + -

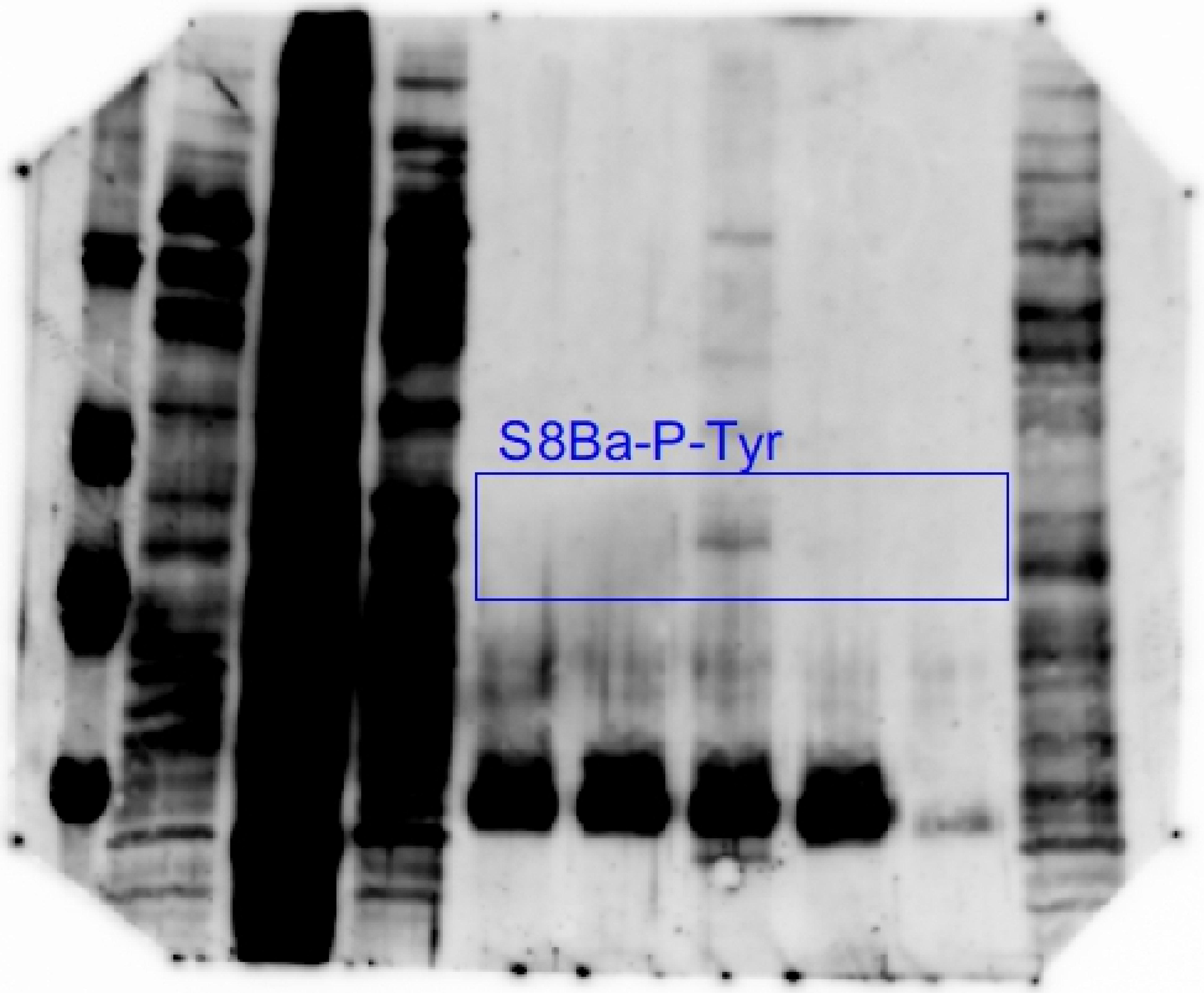
S8Ba-P-Tyr

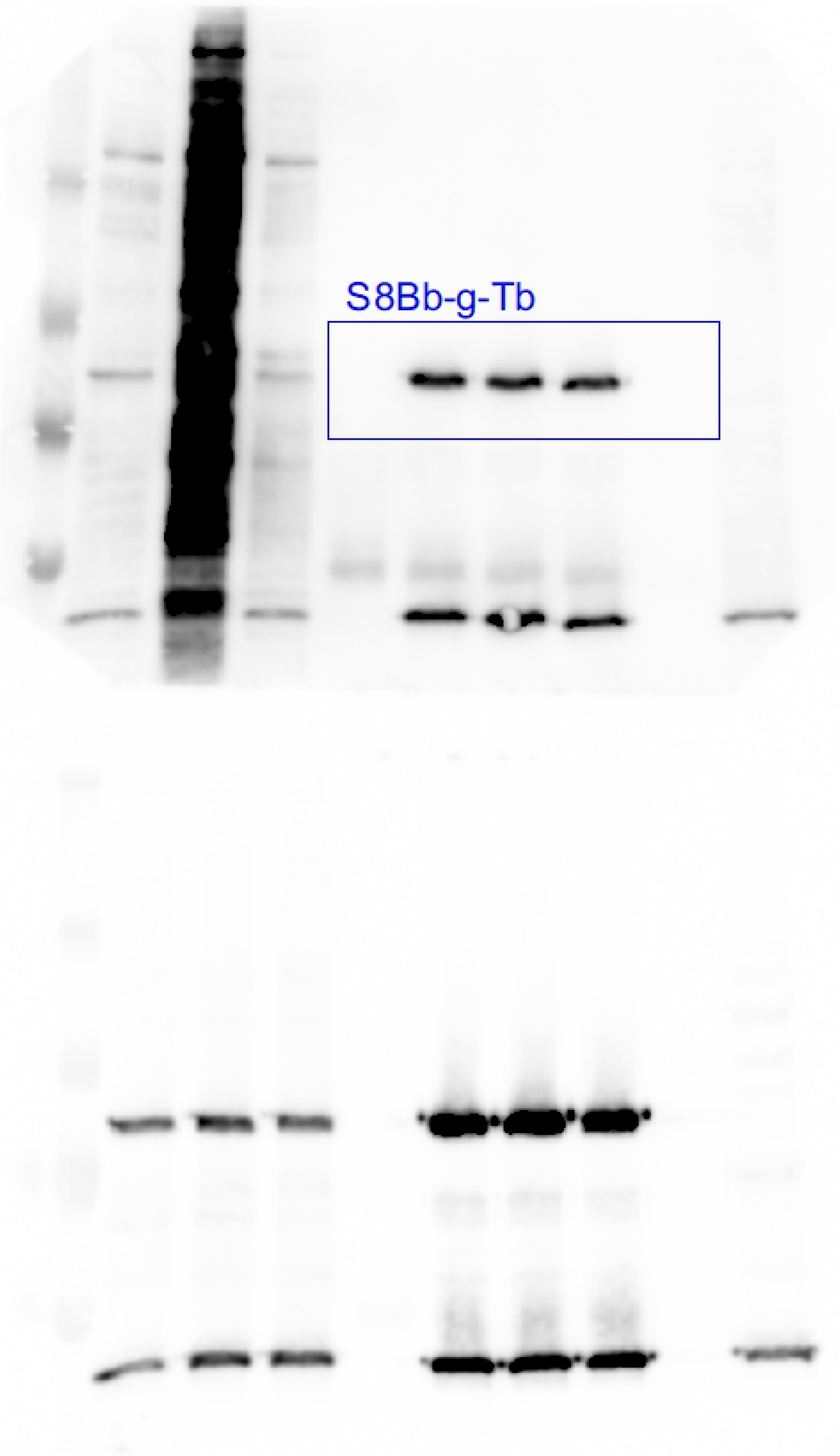
S8Bb-g-Tb

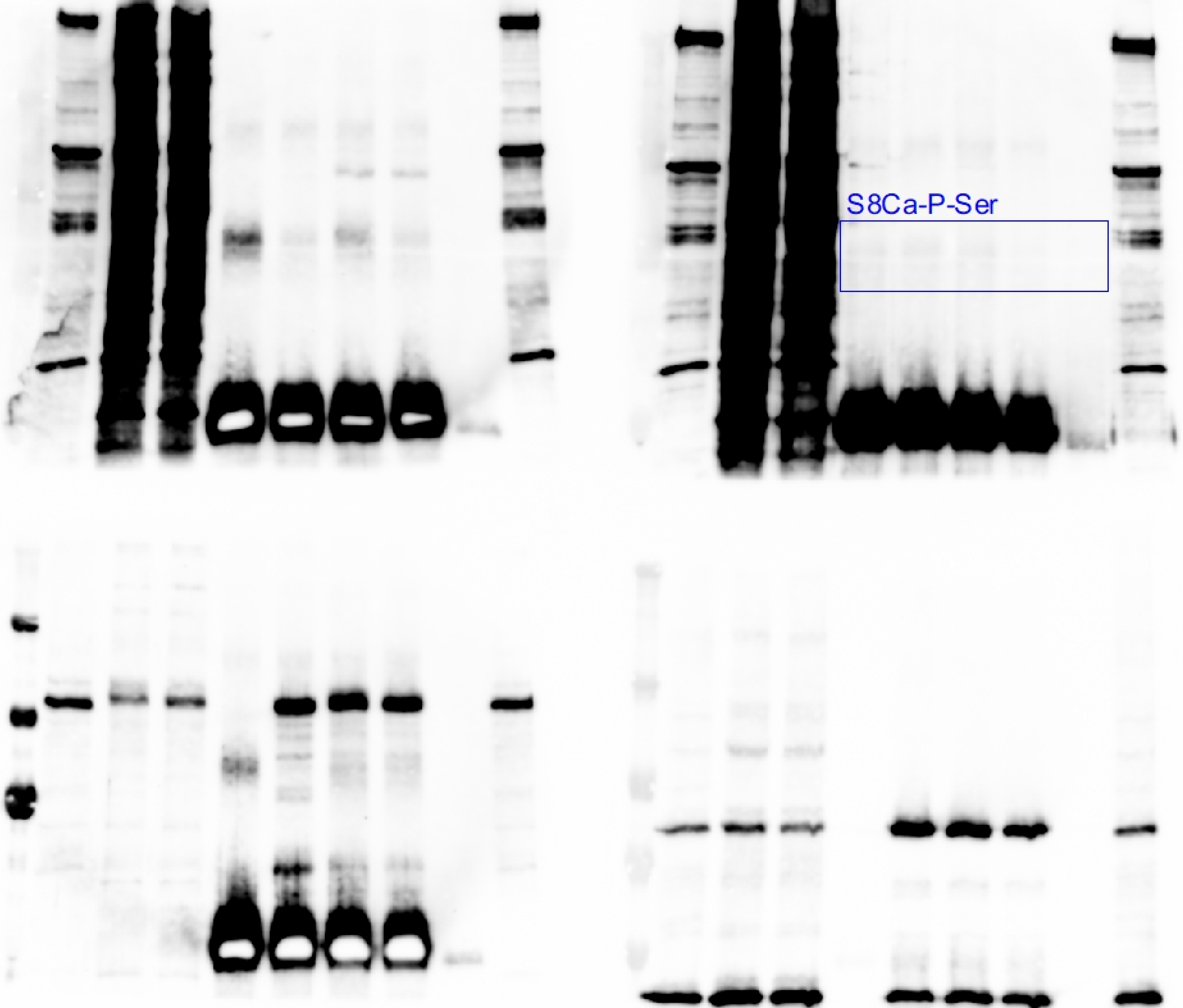
S8Ca-P-Ser

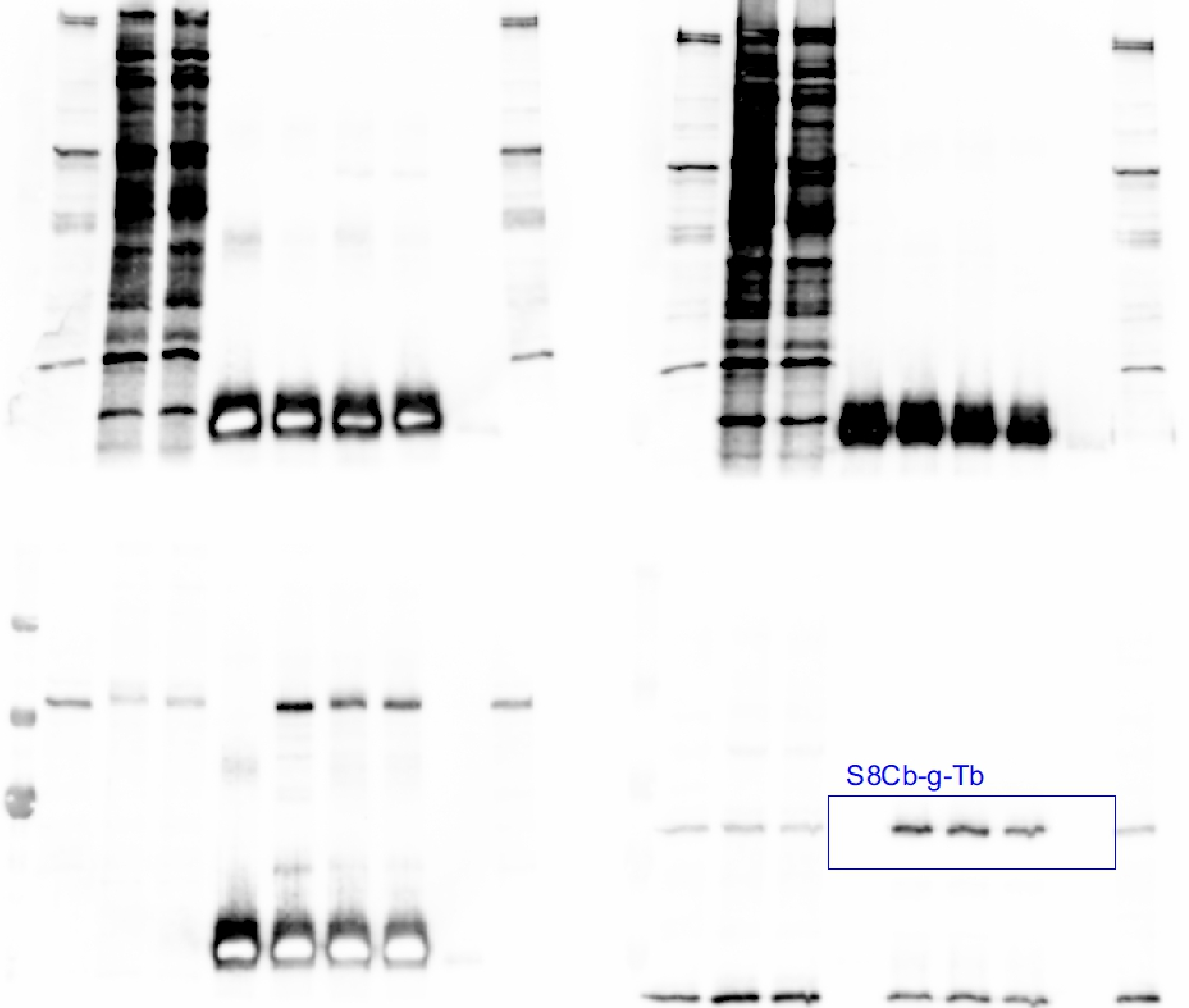
S8Cb-g-Tb

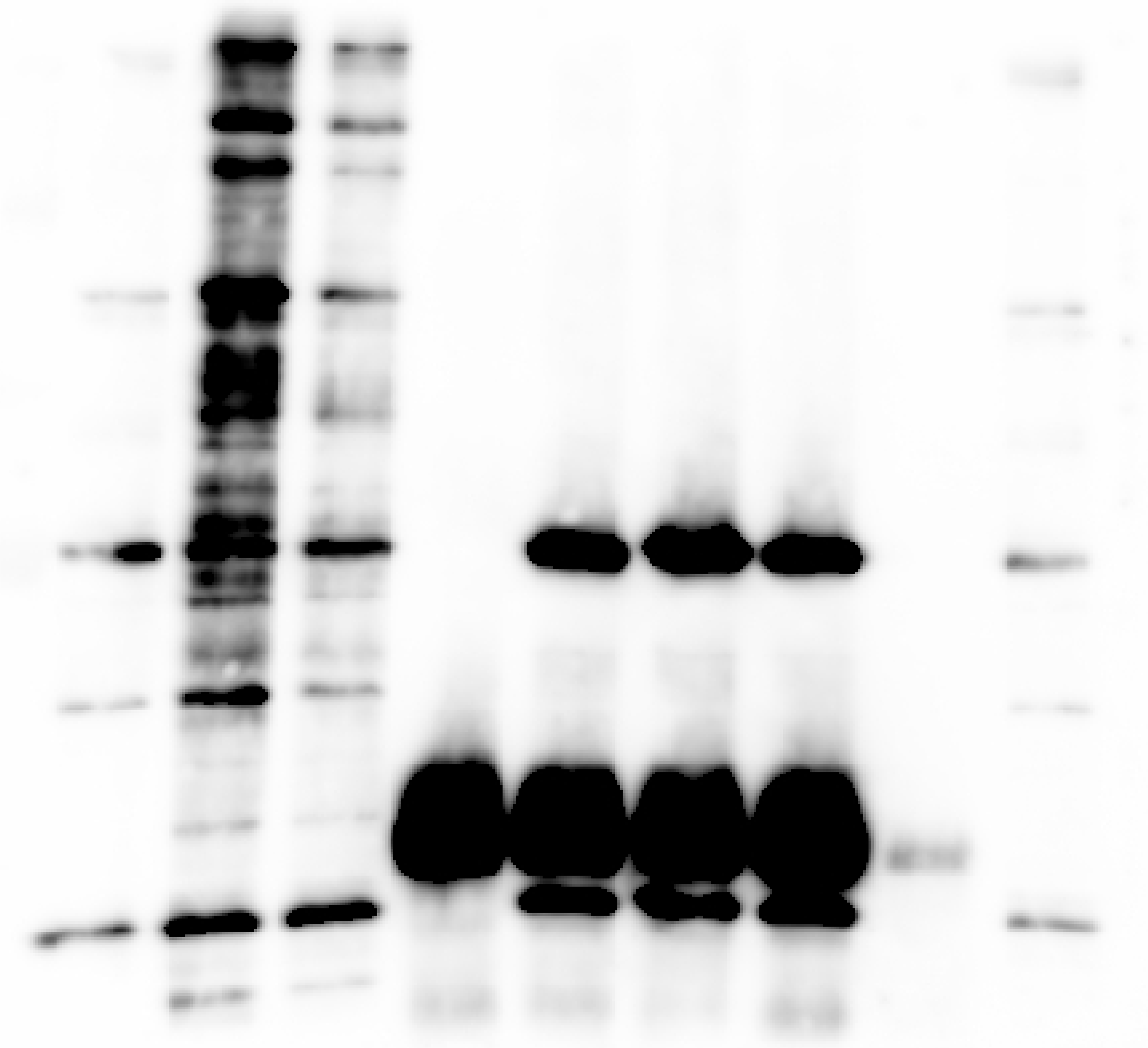

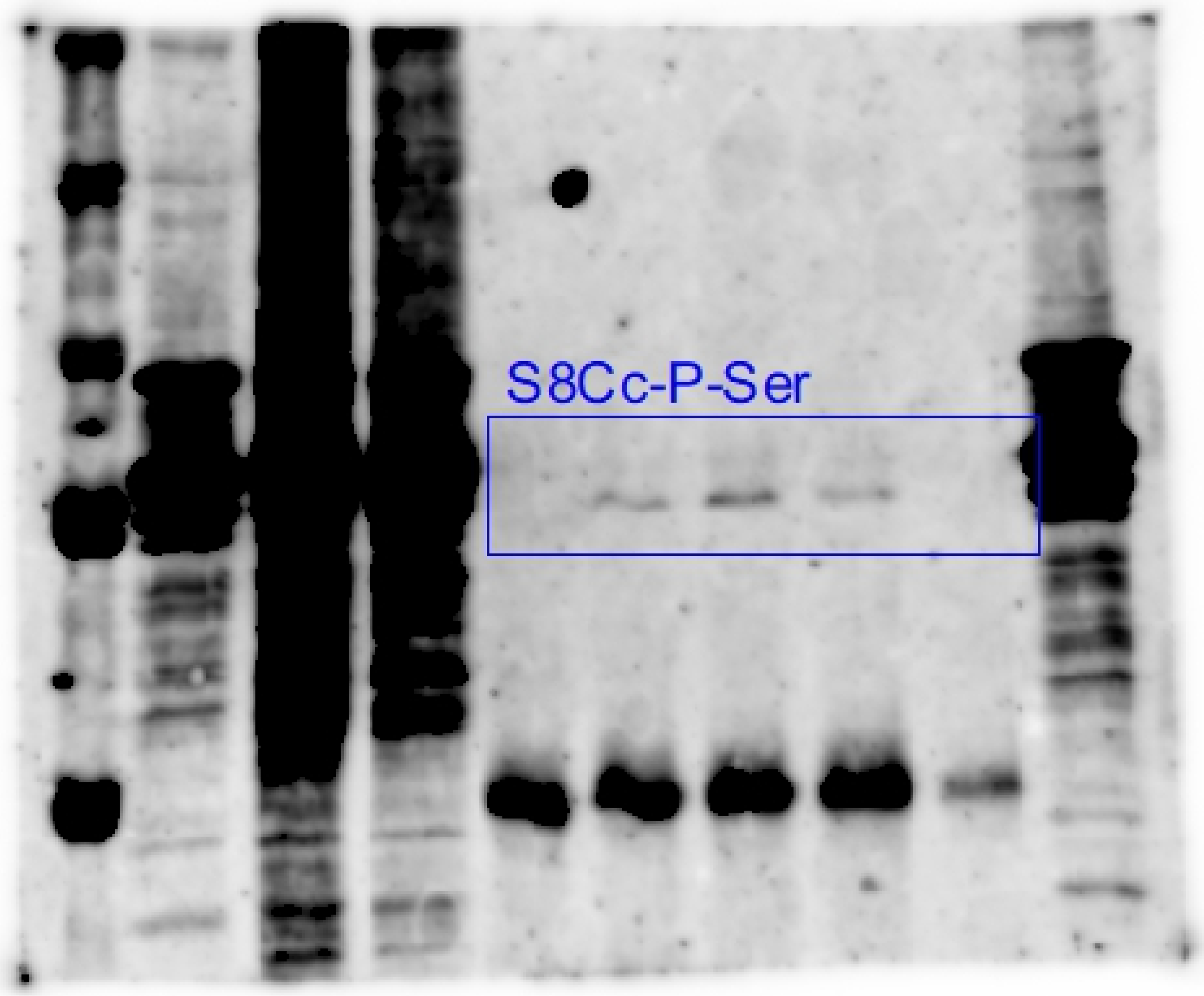
S8Cc-P-Ser

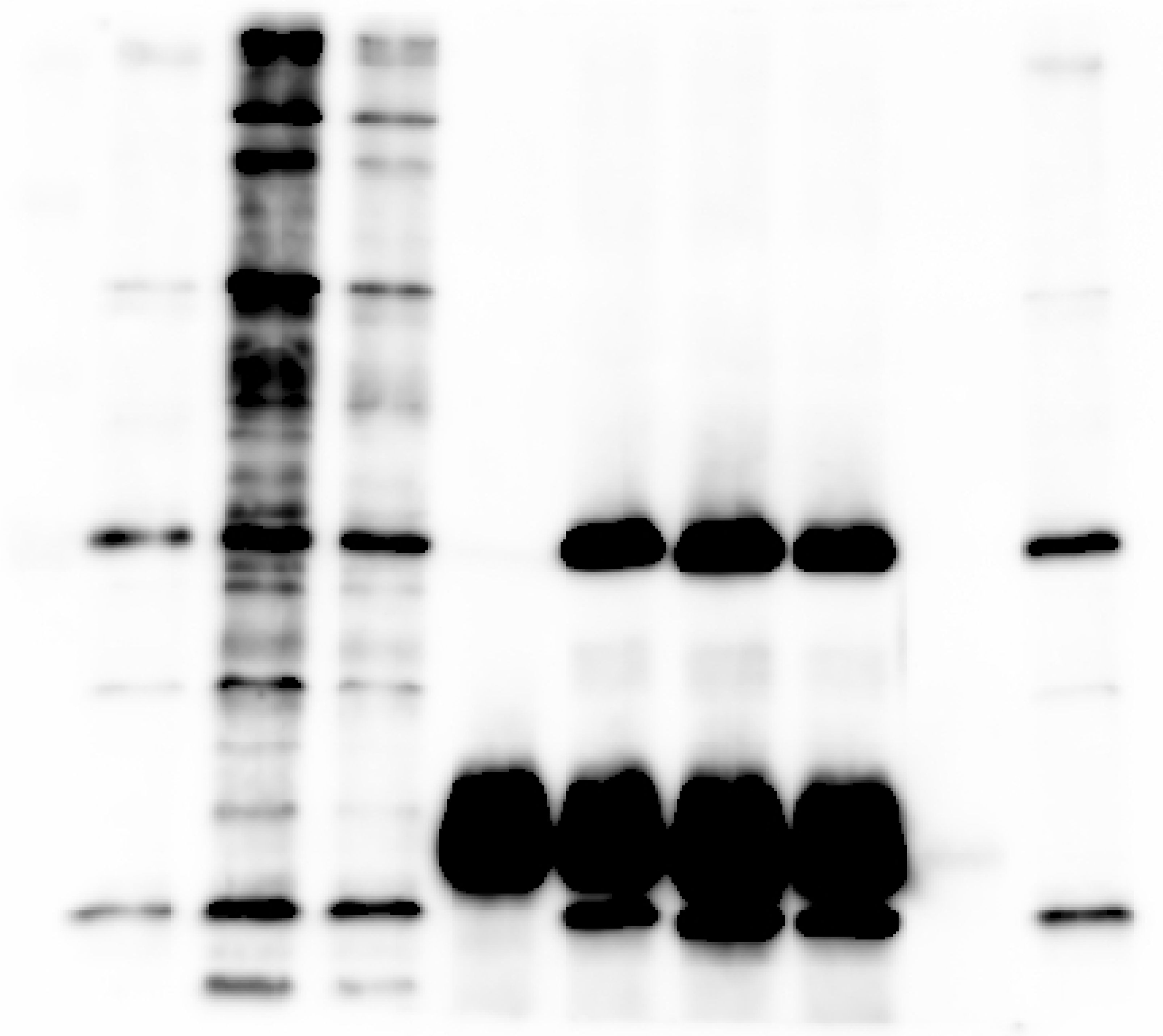

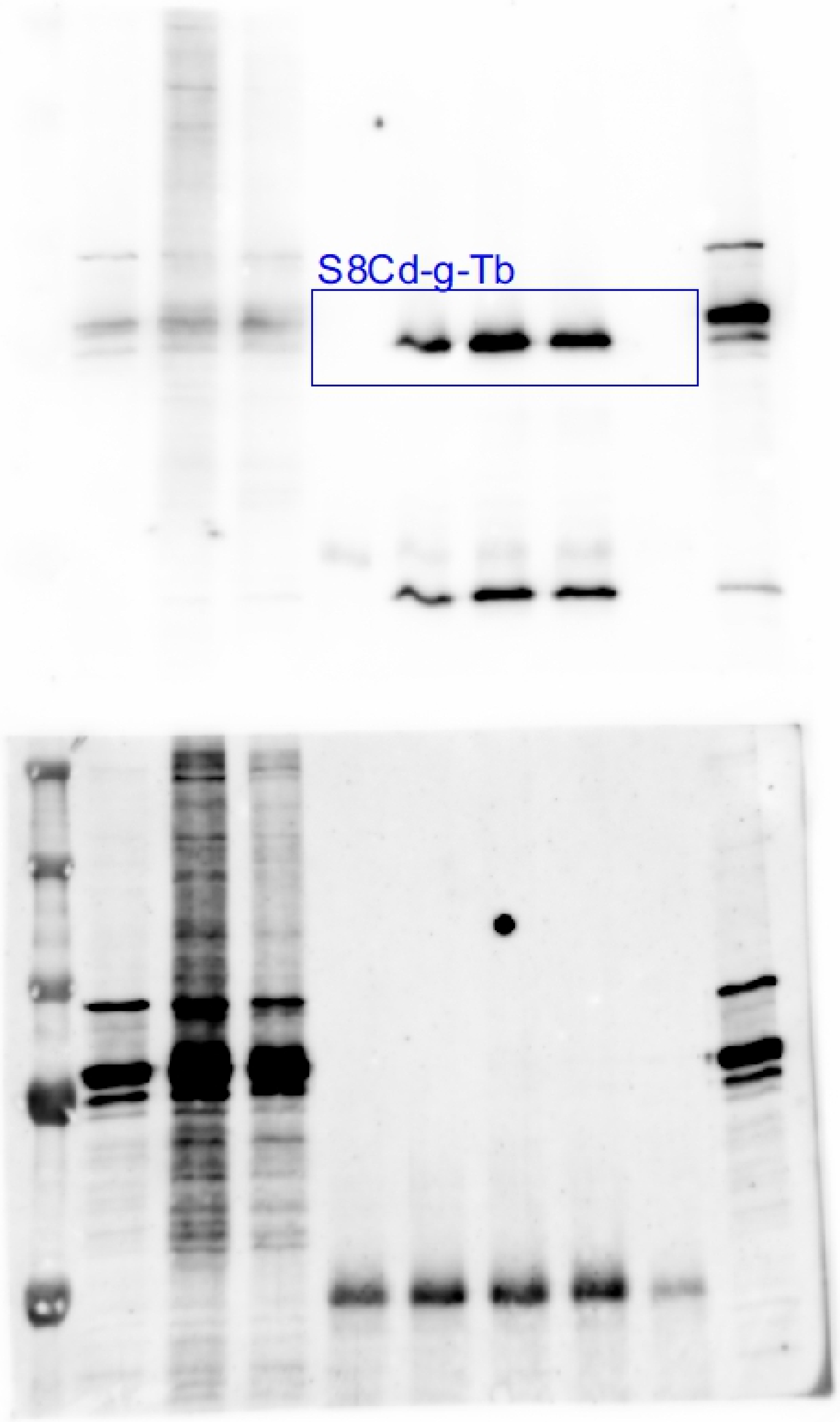
S8Cd-g-Tb
